# *N*^4^-Acetylcytidine enhances synthetic mRNA translation yield and fidelity

**DOI:** 10.1038/s41586-026-10729-8

**Published:** 2026-07-01

**Authors:** Sarah Schiffers, Blake W. Nelson, Maria Prigge, Shriya Krishna, Leslie Watkins, Yining Zhu, Nishu Tyagi, Hamid Beiki, Sudipto Das, Ayush Raman, Jingyao Ma, Thorkell Andresson, Hai-Quan Mao, Bin Wu, Shalini Oberdoerffer

**Affiliations:** 1 Laboratory of Receptor Biology and Gene Expression, Center for Cancer Research, National Cancer Institute, NIH, Bethesda, MD USA; 2 Department of Biophysics and Biophysical Chemistry, Johns Hopkins University School of Medicine, Baltimore, MD USA; 3 Center for Cell Dynamics, Johns Hopkins University School of Medicine, Baltimore, MD USA; 4 Department of Biomedical Engineering, Johns Hopkins University School of Medicine, Baltimore, MD USA; 5 Institute for NanoBioTechnology, Johns Hopkins University, Baltimore, MD USA; 6 Translational Tissue Engineering Center, Johns Hopkins University School of Medicine, Baltimore, MD USA; 7 Protein Characterization Laboratory, Frederick National Laboratory for Cancer Research, Leidos Biomedical Research, Frederick, MD USA; 8 Department of Materials Science and Engineering, Johns Hopkins University, Baltimore, MD USA; 9 Solomon H. Snyder Department of Neuroscience, Johns Hopkins University School of Medicine, Baltimore, MD USA

**Keywords:** Nucleic-acid therapeutics, RNA modification, Innate immune cells, RNA, Ribosome

## Abstract

Synthetic mRNA therapeutics offer a versatile platform for treating diverse conditions, including cancer and infectious diseases. For delivery into cells, these mRNAs are encapsulated in lipid nanoparticles and commonly incorporate modified ribonucleotides to improve stability, enhance translation and mitigate immune recognition^1^. *N*^1^-Methylpseudouridine (m^1^Ψ) has become the industry standard for synthetic mRNAs owing to its effectiveness in promoting translation and reducing immunogenicity^2^. However, recent studies have shown that m^1^Ψ can compromise translational fidelity, leading to errors such as premature termination and ribosomal frameshifting^3–5^. Here we reveal *N*^4^-acetylcytidine (ac^4^C) as a functionally distinct alternative to m^1^Ψ. Across cultured cell lines, primary human monocyte-derived dendritic cells and mouse liver, ac^4^C suppressed inflammatory responses as effectively as m^1^Ψ while driving higher protein yields. Single-molecule imaging of translation revealed broadly similar ribosome densities per mRNA for ac^4^C-modified and m^1^Ψ-modified transcripts. However, translation elongation with m^1^Ψ-modified mRNA was nearly twofold slower than with ac^4^C, which resulted in reduced protein output and increased ribosome collisions that further limited protein production through the engagement of quality-control pathways and +1 frameshifting. These findings underscore the importance of context in designing therapeutic mRNAs and position the translation elongation rate as a key determinant of the efficacy of modified ribonucleotides.

## Abstract (web summary)

Different RNA modifications elicit different translation elongation rates for in vitro transcribed mRNAs that result in disparate translation outputs and fidelity, as shown here for *N*^4^-acetylcytidine versus the industry standard for synthetic mRNAs, *N*^1^-methylpseudouridine.

## Main

In vitro transcribed (IVT) mRNAs have matured into a versatile therapeutic platform, as exemplified by mRNA-based COVID-19 vaccines that program cells to express immunogenic viral proteins^1,6^. To enhance stability and translation, synthetic mRNAs are 5′ capped with *N*^7^-methylguanosine and 3′ polyadenylated^7^. Unlike endogenous transcripts, which are exported directly to the cytosol, IVT mRNAs enter cells through endosomal pathways that engage innate immune sensors. This process induces type I interferon responses and eIF2α phosphorylation (p-eIF2α), which promotes RNA degradation and global translational repression^6^. Uniform incorporation of modified nucleoside triphosphates (NTPs) during in vitro transcription mitigates immune detection and enhances mRNA stability and protein output^1,6,8^. However, recent findings have indicated that the current industry standard m^1^Ψ compromises translational fidelity and thereby generates unintended neoantigens^3,9^. These observations highlight the need for alternative or complementary strategies to optimize the safety and efficacy of mRNA therapeutics.

Of the approximately 170 known RNA modifications, only a small subset has been evaluated for mRNA therapeutic applications^7,10^. We previously demonstrated that ac^4^C, a naturally occurring modification, potently enhances IVT mRNA translation in HeLa cells^11^. Mechanistically, ac^4^C stabilizes Watson–Crick base pairing with guanosine, which strengthens interactions with cognate tRNAs and promotes RNA secondary structure formation^12^. The net effect is a position-dependent modulation of translation. In coding sequences (CDSs), ac^4^C promotes translation elongation by improving tRNA–mRNA decoding efficiency^11^. Conversely, in 5′ untranslated regions (UTRs), ac^4^C inhibits translation initiation by forming structures that hinder ribosome scanning^11,13^. Notably, as a cytidine modification, ac^4^C is inherently excluded from start and stop codons, sites where nucleotide modifications may disrupt initiation and termination^13,14^.

Given these properties, we examined the effects of incorporating ac^4^C into IVT mRNAs alongside the industry standard m^1^Ψ. Furthermore, we evaluated pseudouridine (Ψ) and 5-methylcytidine (m^5^C), which have also been explored in mRNA therapeutics^2,8^.

## ac^4^C enhances IVT mRNA translation

**Fig. 1:**
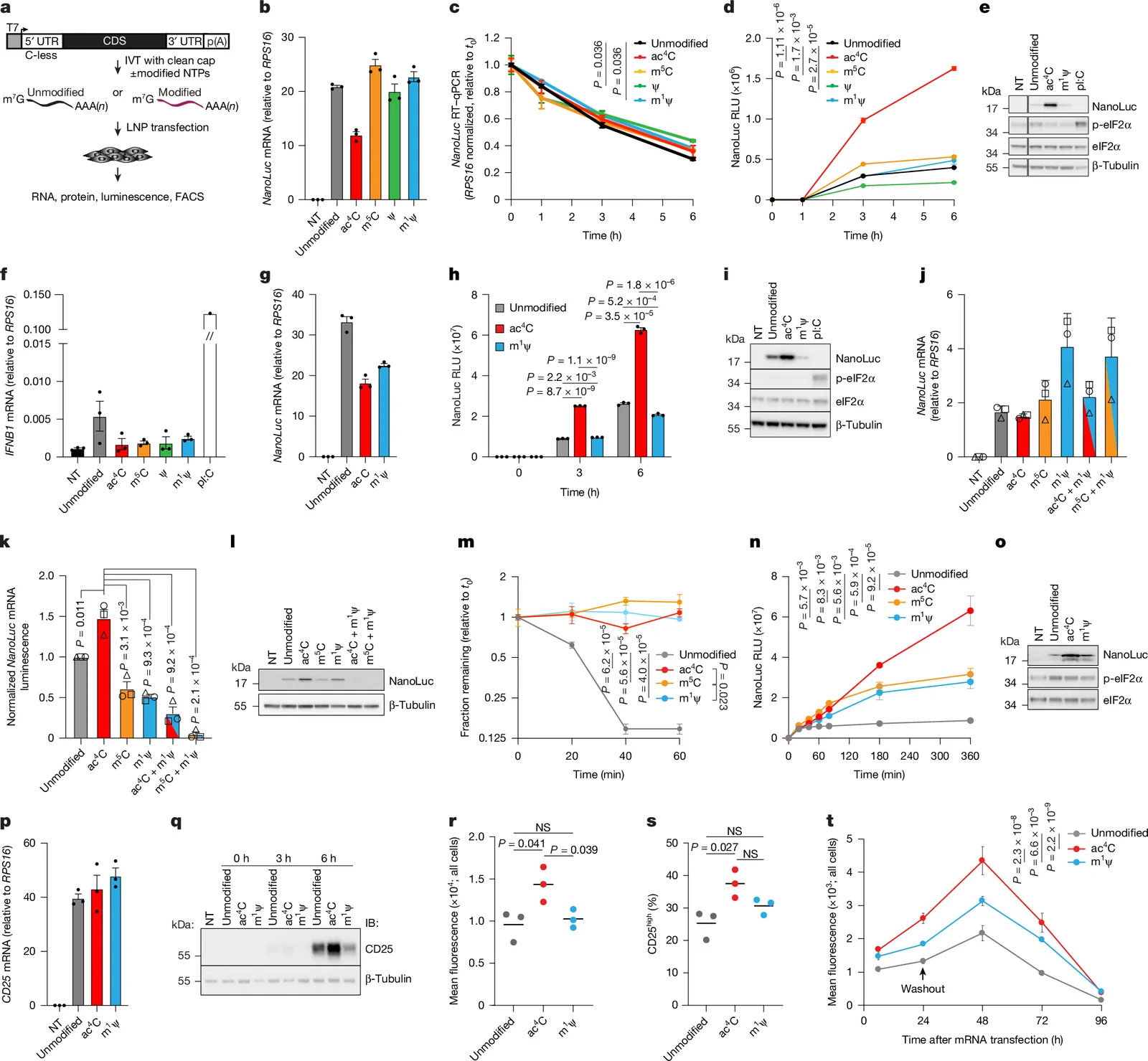
ac^4^C enhances IVT mRNA translation. **a**, Schematic of IVT mRNA production and transfection. **b**–**f**, *NanoLuc* translation following transfection into HeLa cells. **b**,* NanoLuc* mRNA levels at *t *= 0 (5 min), normalized to *RPS16*. NT, non-transfected. **c**, *NanoLuc* mRNA decay at indicated times after transfection.** d**, NanoLuc luminescence at indicated times. RLU, relative light units.** e**, Representative immunoblot of indicated proteins at 6 h after transfection, with β-tubulin as the loading control. **f**, *IFNB1* mRNA levels at 6 h after transfection, with polyI:C (pI:C) as a positive control. *n* = 3; two-tailed unpaired Student’s *t*-test on the area under the curve (AUC) compared with unmodified mRNA (**c**,**d**). **g**–**i**, *NanoLuc* translation in HeLa cells following transfection with G47A+884G mutant T7-derived IVT mRNA. **g**, mRNA levels after 5 min of transfection and washout. **h**, Luminescence at the indicated times. **i**, Western blot at 6 h after transfection. *n* = 3; two-tailed unpaired Student’s *t*-test (**h**). **j**–**l**, *NanoLuc* translation in HeLa cells following electroporation of IVT mRNA. **j**, mRNA levels immediately after electroporation.** k**, Luminescence at 6 h. **l**, Immunoblot at 6 h after transfection. *n* = 3; two-tailed unpaired Student’s *t*-test compared with ac^4^C-modified mRNA (**k**). **m**–**o**, *NanoLuc* translation in RRLs. **m**, mRNA levels normalized to time zero.** n**, Luminescence at the indicated times. **o**, Representative immunoblot of proteins at 80 min. *n* = 3; two-tailed unpaired Student’s* t*-test on the AUC compared with unmodified mRNA (**n**). **p**–**t**, *CD25* translation in HeLa cells following transfection of IVT mRNA. **p**, mRNA levels after 5 min of transfection and washout. **q**, Immunoblot (IB) at indicated times after transfection. **r**,**s**, Surface expression of all cells (**r**) and CD25^high^ fraction (**s**) by flow cytometry at 6 h. **t**, Surface expression over time after extended transfection. *n* = 3; two-tailed unpaired Student’s *t*-test. NS, not significant. Data are the mean ± s.e.m.; only significant values are shown unless otherwise indicated; *n* indicates independent biological replicates.

**Extended Data Fig. 1:**
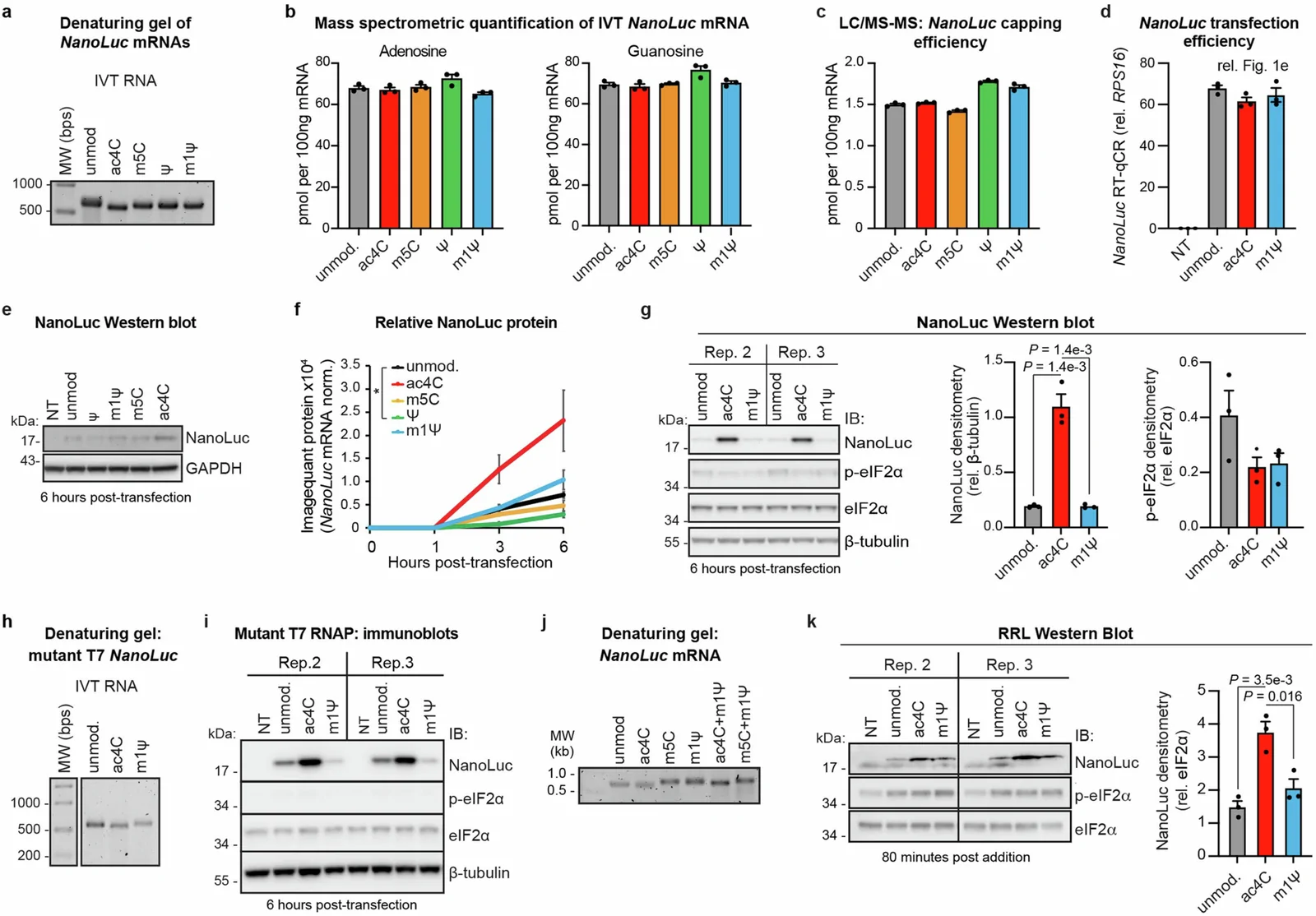
ac^4^C enhances *NanoLuc* translation in HeLa cells and in vitro. **a**, Denaturing agarose gel electrophoresis of *NanoLuc* IVT mRNA synthesized with the indicated modified NTPs. **b**, Mass spectrometric quantification of adenosine and guanosine in 100 ng IVT mRNA containing the indicated modified NTPs (n = 3). **c**, Mass spectrometric quantification of 2′-O-methyladenosine to assess clean-capping efficiency in 100 ng IVT mRNA containing the indicated modified NTPs (n = 3). **d**, *NanoLuc* mRNA levels at t = 0 (5 min), normalized to *RPS16*; NT, non-transfected (n = 3). **e**, Western blot of NanoLuc 6 h post-transfection; GAPDH, loading control. Representative of three independent experiments. **f**, Quantification of NanoLuc protein levels by densitometry of immunoblots at the indicated time points, normalized to transfected mRNA levels (n = 3; *P = 0.013 versus unmodified *NanoLuc*; two-tailed unpaired Student’s t-test at 6 h post-transfection). **g**, Western blot of additional replicates related to Fig. 1e following transfection with *NanoLuc* IVT mRNA, with corresponding densitometry of NanoLuc and relative p-eIF2α (n = 3; two-tailed unpaired Student’s t-test). **h**, Denaturing agarose gel electrophoresis of *NanoLuc* IVT mRNA synthesized using G47A+884G mutant T7 RNA polymerase. **i**, Western blot of additional replicates related to Fig. 1i following transfection with *NanoLuc* mRNA synthesized using G47A+884G mutant T7 RNA polymerase. **j**, Denaturing agarose gel electrophoresis of *NanoLuc* IVT mRNA synthesized with an expanded set of modified NTPs, including m^5^C- and double-modified mRNAs. **k**, Western blot of additional replicates related to Fig. 1o following programming of rabbit reticulocyte lysate (RRL) with *NanoLuc* IVT mRNA, with corresponding protein densitometry (n = 3; two-tailed unpaired Student’s t-test). Data are mean ± s.e.m.; n = independent technical replicates (b,c) or independent biological replicates (d-k).

A DNA template encoding optimized UTRs flanking the CDS of bioluminescent NanoLuciferase (NanoLuc) was IVT with ac^4^C or m^5^C substituted for cytidine or with Ψ or m^1^Ψ substituted for uridine (Fig. 1a and Supplementary Table 1). All mRNAs were co-transcriptionally capped and polyadenylated. To avoid inhibitory effects of ac^4^C on translation initiation, IVT templates were designed with C-less 5′ UTRs that preserved the Kozak sequence, which restricts ac^4^C to the CDS^13^. In vitro transcription with canonical NTPs served as an unmodified control. RNA integrity, yield and capping efficiency were validated (Extended Data Fig. 1a–c).

Synthetic mRNAs were encapsulated into lipid nanoparticles (LNPs) and transfected into HeLa cells, with complexes removed after 5 min to synchronize uptake. Quantitative PCR with reverse transcription (RT–qPCR) confirmed efficient delivery across conditions (Fig. 1b). Chemically modified mRNAs exhibited prolonged half-lives relative to unmodified mRNA, although overall decay kinetics were similar (Fig. 1c). Despite comparable or reduced RNA levels, ac^4^C-modified *NanoLuc* led to increased protein synthesis relative to all other conditions, including m^1^Ψ, as measured by luminescence and immunoblotting experiments (Fig. 1d,e and Extended Data Fig. 1d–g).

The enhanced translation of modified mRNAs is often attributed to reduced innate immune activation^6^. However, immune signalling was minimal in HeLa cells, with all IVT mRNAs inducing little to no *IFNB1* or p-eIF2α expression (Fig. 1e,f). Furthermore, the translational advantage of ac^4^C persisted when mRNAs were synthesized using a G47A+884G mutant T7 polymerase, which suppresses immunostimulatory double-stranded RNA (dsRNA) by-products^15^. NanoLuc protein levels remained highest for ac^4^C-modified mRNA, even as p-eIF2α was further reduced relative to wild-type (WT) T7 (Fig. 1g–i and Extended Data Fig. 1h,i).

Endosomal escape is inefficient, and total cellular RNA may not reflect the translation-competent pool^16^. Accordingly, we quantified translation after electroporation, which delivers IVT mRNAs directly to the cytoplasm and minimizes the confounding effects of immune responses^17^. m^5^C served as a control cytidine modification, whereas ac^4^C–m^1^Ψ and m^5^C–m^1^Ψ double-modified mRNAs were used to assess combinatorial effects (Extended Data Fig. 1j). Across replicates, m^1^Ψ-modified mRNA showed higher electroporation efficiency (Fig. 1j). However, ac^4^C modestly increased NanoLuc protein output and further exceeded that mediated by m^1^Ψ after normalization to mRNA levels (Fig. 1k). This effect was specific to ac^4^C, as m^5^C reduced NanoLuc levels and double-modified mRNAs showed minimal translation (Fig. 1l).

To further isolate the direct effects of nucleotide modifications on translation, we used rabbit reticulocyte lysate (RRL), a cell-free system that retains the core translational machinery but lacks key innate immune pathways, and translation efficacy is independent of delivery^18^. RRLs were programmed with equivalent amounts of each transcript, and RNA and protein levels were monitored over time. Unmodified *NanoLuc* mRNA degraded rapidly, whereas modified transcripts remained stable over the sampling window (Fig. 1m). Correspondingly, luminescence from unmodified mRNA plateaued within 30 min, whereas luminescence from m^5^C-modified and m^1^Ψ-modified mRNAs accumulated for longer periods before plateauing. By contrast, ac^4^C-modified mRNA supported sustained and increased protein synthesis throughout the time course, without a clear plateau (Fig. 1n). Immunoblotting confirmed the increase in NanoLuc protein from ac^4^C-modified mRNA over time without detectable p-eIF2α (Fig. 1o and Extended Data Fig. 1k). Thus, ac^4^C enhances protein synthesis through an intrinsic effect on translation, independent of immune signalling or the delivery method.

**Extended Data Fig. 2:**
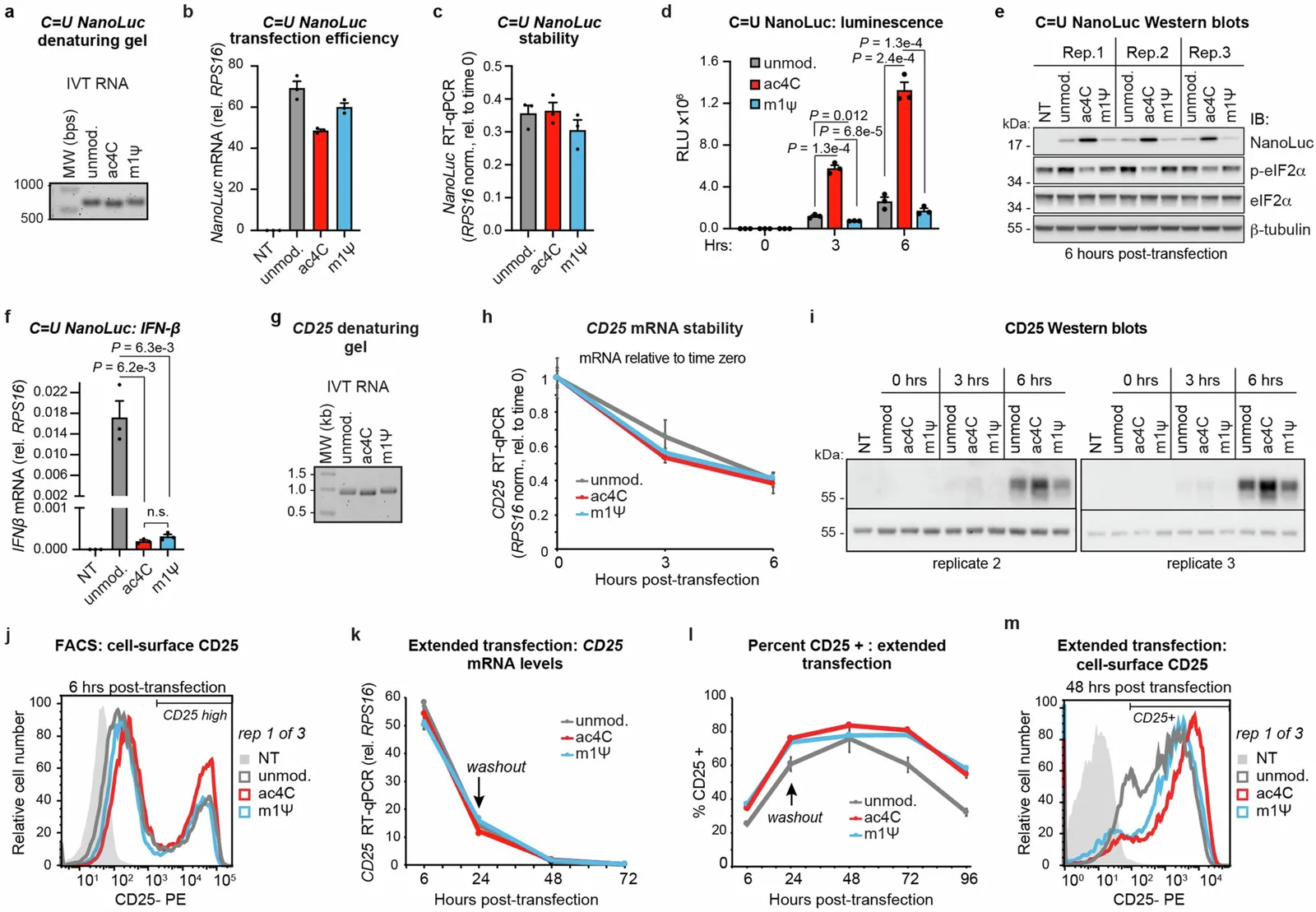
ac^4^C-stimulated translation extends beyond NanoLuc in HeLa cells. **a**, Denaturing agarose gel electrophoresis of codon-optimized *C* = *U NanoLuc* IVT mRNA synthesized with the indicated modified NTPs. **b**, *C* = *U NanoLuc* mRNA levels at t = 0 (5 min), normalized to *RPS16*; NT, non-transfected (n = 3). **c**, Fraction of *C* = *U NanoLuc* mRNA remaining 6 h post-transfection in HeLa, from b, normalized to *RPS16* and time zero (n = 3). **d**, NanoLuc luminescence at the indicated times after transfection with *C* = *U NanoLuc* mRNA (n = 3; two-tailed unpaired Student’s t-test). **e**, Western blot of NanoLuc following transfection with *C* = *U NanoLuc* mRNA. **f**, *IFNβ1* mRNA levels 6 h post-transfection with *C* = *U NanoLuc* mRNA, normalized to *RPS16* (n = 3; two-tailed unpaired Student’s t-test). **g**, Denaturing agarose gel electrophoresis of *CD25* IVT mRNA synthesized with the indicated modified NTPs. **h**, Fraction of *CD25* mRNA remaining at the indicated times post-transfection in HeLa, normalized to *RPS16* and time zero, from Fig. 1p (n = 3). **i**, Western blot of additional replicates related to Fig. 1q following transfection with *CD25* mRNA. **j**, Representative histogram of cell-surface CD25 staining, related to Fig. 1r,s (n = 3). **k**, *CD25* mRNA levels following 24-h transfection with *CD25*–LNP complexes and washout, measured by RT-qPCR at the indicated times from initiation of transfection and normalized to *RPS16* (n = 3). **l**, FACS analysis of the percentage of CD25^+^ cells at the indicated times post-transfection, from Fig. 1t (n = 3). **m**, Representative histogram of cell-surface CD25 staining 48 h after extended transfection (n = 3). Data are mean ± s.e.m.; n = independent biological replicates.

To test whether the translational advantage of ac^4^C persists under optimized decoding conditions, the *NanoLuc* open-reading frame was codon-optimized to maximize theoretical mRNA stability in human cells^19^. This redesign increased wobble uridine by around 30%, which enriched the nucleotide position most responsive to m^1^Ψ-mediated enhancement of translation^9^ while balancing the overall cytidine and uridine content to enable direct comparison of ac^4^C and m^1^Ψ (Extended Data Fig. 2a and Supplementary Table 1). Under these conditions, ac^4^C-modified mRNA continued to suppress immune activation and to produce higher protein levels than m^1^Ψ and unmodified controls, without altering mRNA stability (Extended Data Fig. 2b–f).

Finally, to assess whether the translational advantage of ac^4^C extends to membrane proteins, we analysed CD25. This protein is a therapeutically relevant transmembrane receptor, and its deficiency is associated with severe immune dysregulation^20^ *CD25* IVT mRNAs were synthesized, validated and transfected into CD25-negative HeLa cells (Extended Data Fig. 2g and Supplementary Table 1). RT–qPCR confirmed that transfection efficiencies and mRNA stability were comparable across conditions after 5 min of transfection and LNP washout. By contrast, ac^4^C-modified mRNA produced higher CD25 protein levels (as measured by immunoblotting) than m^1^Ψ-modified and unmodified transcripts (Fig. 1p,q and Extended Data Fig. 2h,i).

As a cell surface protein, CD25 can be quantified by flow cytometry. At 6 h after transfection, the mean fluorescence intensity and the fraction of CD25^high^ cells were increased for ac^4^C-modified relative to m^1^Ψ-modified and unmodified mRNAs (Fig. 1r,s and Extended Data Fig. 2j). To better approximate in vivo exposure, LNP incubation was extended to 24 h and CD25 expression was monitored over 96 h. mRNA uptake remained comparable across modifications, with transcripts becoming undetectable by 72 h (Extended Data Fig. 2k). The fraction of CD25-positive cells remained similar for ac^4^C-modified and m^1^Ψ-modified mRNAs, whereas unmodified mRNA showed reduced expression (Extended Data Fig. 2l). By contrast, the mean fluorescence intensity, which reflects protein levels per cell, was consistently higher for ac^4^C-modified mRNA throughout the time course of the experiment (Fig. 1t and Extended Data Fig. 2m). Thus, ac^4^C supports higher protein output per cell even when delivery levels are comparable.

## ac^4^C reduces immune responses to IVT mRNA

The above results established that ac^4^C improves translation in low-immune settings. Next, we evaluated its performance in therapeutically relevant contexts, including immune cells, primary fibroblasts and liver in vivo. Monocyte-derived macrophages and dendritic cells were prioritized given their high expression of pattern recognition receptors and their central role in mRNA vaccine responses^6^.

**Fig. 2:**
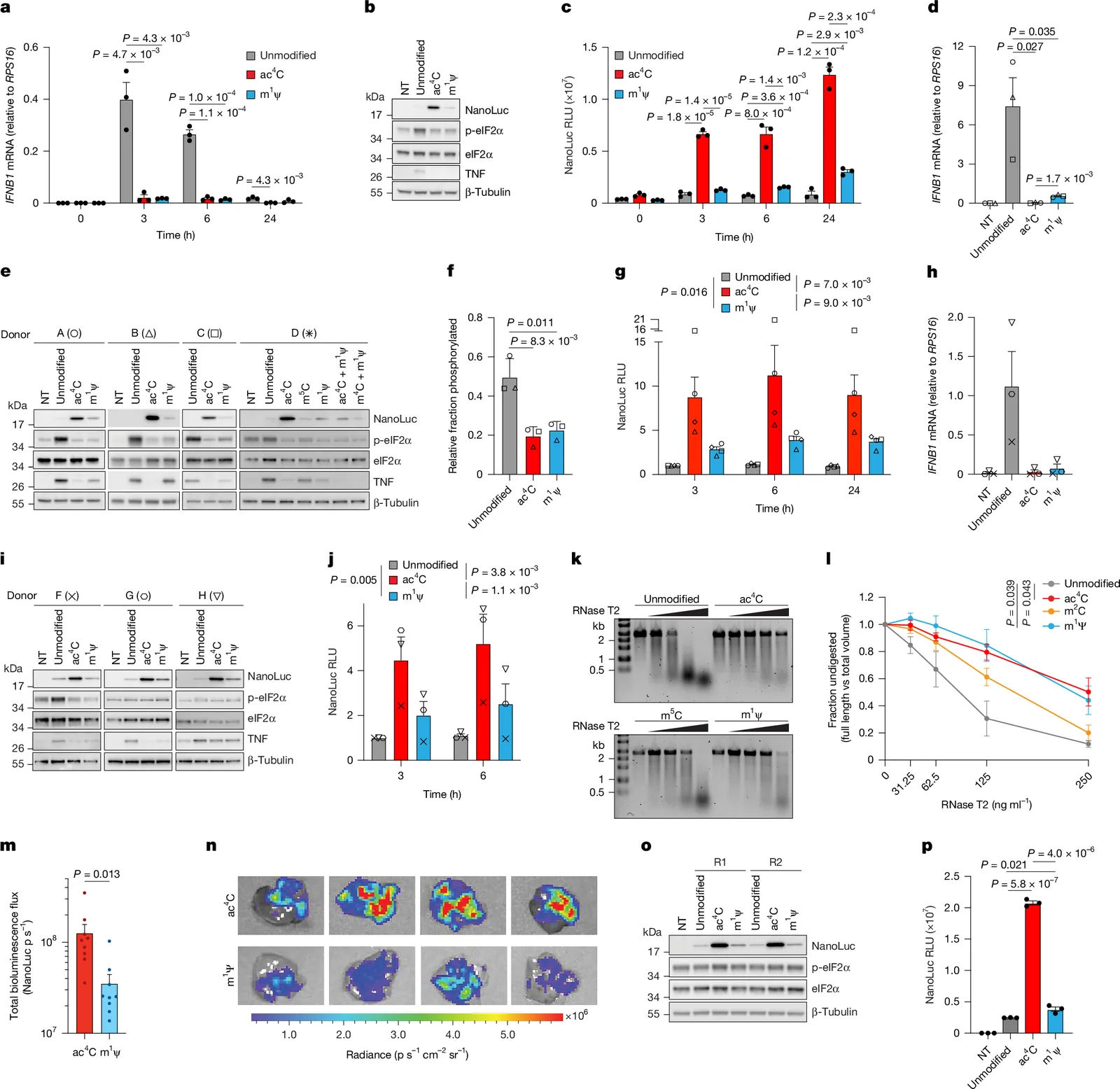
ac^4^C enhances translation independently of innate immune activation. **a**–**c**, *NanoLuc* translation in THP-1-derived M0 macrophages. **a**, *IFNB1* mRNA levels at indicated times. **b**, Immunoblot at 6 h after transfection. **c**, Luminescence at indicated times. *n* = 3; two-tailed unpaired Student’s *t*-test (**a**,**c**). **d**–**g**, *NanoLuc* translation in primary human MoDCs from independent donors (A–D). **d**, *IFNB1* mRNA levels at 6 h. **e**, Immunoblot at 6 h after transfection. **f**, Densitometry of p-eIF2α/total eIF2α from **e**. **g**, Luminescence at indicated times, normalized to each donor’s unmodified mRNA signal at 3 h. *n* = 3; two-tailed unpaired Student’s *t*-test (**d**,**f**), or two-tailed paired Student’s *t*-test (**g**). **h**–**j**, *NanoLuc* translation in MoDCs from donors (F–H) following transfection with G47A+884G mutant T7-derived IVT mRNA. **h**, *IFNB1* mRNA levels at 6 h. **i**, Immunoblot at 6 h after transfection. **j**, Luminescence at the indicated times, normalized to each donor’s unmodified mRNA signal at 3 h. *n* = 3; two-tailed paired Student’s *t*-test (**h**,**j**). **k**,**l**, In vitro digestion of *FLuc* IVT mRNA with increasing amounts of RNase T2. **k**, Denaturing agarose gel electrophoresis. **l**, Densitometric quantification of full-length mRNA relative to total lane volume. *n* = 3; one-way analysis of variance with two-tailed unpaired Student’s *t*-tests using the AUC (**l**). **m**, Ex vivo luminescence flux in mouse livers 24 h after intravenous injection of 10 μg *NanoLuc* IVT mRNA–LNPs per mouse. *n* = 9 mice from 2 independent experiments; two-tailed unpaired Student’s *t*-test. **n**, Representative liver luminescence images of the mRNAs from **m**. *n* = 4 biologically independent samples. **o**,**p**, *NanoLuc* translation in MEFs. Immunoblot of two replicates at 6 h after transfection (R1 and R2; **o**) and luminescence at 3 h after transfection (**p**). *n* = 3; two-tailed unpaired Student’s *t*-test (**p**). Data are the mean ± s.e.m. unless otherwise indicated; *n* indicates independent biological replicates; only significant values are shown.

**Extended Data Fig. 3:**
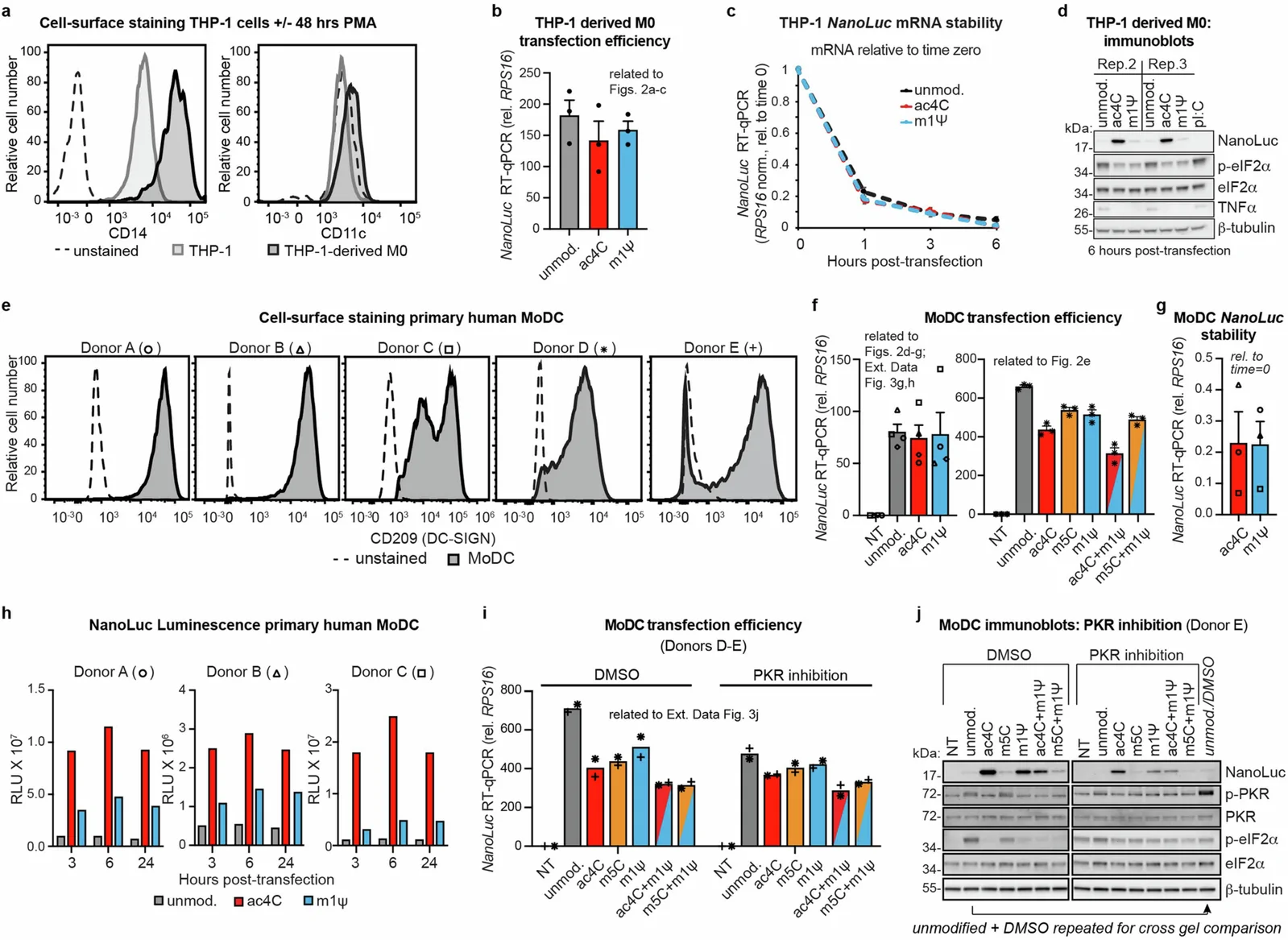
ac^4^C enhances *NanoLuc* translation in human monocytes and dendritic cells. **a**, Representative FACS histograms of cell-surface CD14 and CD11c in parental THP-1 cells and PMA-stimulated THP-1-derived M0 macrophages (n = 3). **b**, *NanoLuc* mRNA levels in THP-1-derived M0 macrophages immediately after 5-min transfection and LNP washout (t = 0), measured by RT-qPCR and normalized to *RPS16* (n = 3). **c**, Fraction *NanoLuc* mRNA remaining at the indicated times post-transfection in THP-1-derived M0 macrophages, normalized to *RPS16* and time zero, from b (n = 3). **d**, Western blot of additional replicates related to Fig. 2b following *NanoLuc* mRNA transfection into THP-1-derived M0 macrophages. **e**, FACS histograms of cell-surface CD209 (DC-SIGN) in primary human monocyte-derived dendritic cells (MoDCs) from five individual donors. **f**, *NanoLuc* mRNA levels in primary human MoDCs (Donors A-D) immediately after 5-min transfection and LNP washout (t = 0), measured by RT-qPCR and normalized to *RPS16* (n = 4). **g**, Fraction of *NanoLuc* mRNA remaining at the indicated times post-transfection in MoDCs, normalized to *RPS16* and time zero, from f (n = 3). **h**, Individual NanoLuc luminescence measurements in primary human MoDCs related to Fig. 2g at the indicated times following IVT mRNA transfection. **i**, *NanoLuc* mRNA levels in MoDCs (Donors D-E) pretreated with PKR inhibitor C16 (1.5 μM, 10 min) prior to 5-min IVT mRNA transfection, measured immediately after LNP washout (t = 0) and normalized to *RPS16* (n = 2). **j**, Representative Western blot of NanoLuc in MoDCs from i, 6 h post-transfection with PKR inhibition (C16, 1.5 μM). Lysate from DMSO-treated cells transfected with unmodified *NanoLuc* is included on the PKR-inhibited blot for comparison. C16 reduces PKR phosphorylation but induces and equilibrates phospho-eIF2α through activation of the integrated stress response^21^, enabling comparison between conditions. All samples were processed in parallel (n = 2 donors). Data are mean ± s.e.m.; n = independent biological replicates.

THP-1-derived M0 macrophages were transfected with LNP-encapsulated *NanoLuc* mRNA. Comparable transfection efficiencies and mRNA stabilities were observed across conditions (Extended Data Fig. 3a–c). Unmodified mRNA induced strong immune activation, as evidenced by increased *IFNB1*, *TNF* and p-eIF2α levels, whereas ac^4^C and m^1^Ψ suppressed these responses to similar levels (Fig. 2a,b and Extended Data Fig. 3d). Nonetheless, ac^4^C-modified mRNA led to increased NanoLuc protein yields (Fig. 2b,c and Extended Data Fig. 3d). A similar pattern emerged in primary monocyte-derived dendritic cells (MoDCs). Both modifications reduced immune activation relative to unmodified mRNA, with ac^4^C leading to modestly lower *IFNB1*, *TNF* and p-eIF2α levels. However, ac^4^C again increased NanoLuc protein yields across samples from human donors (Fig. 2d–g and Extended Data Fig. 3e–h).

**Extended Data Fig. 4:**
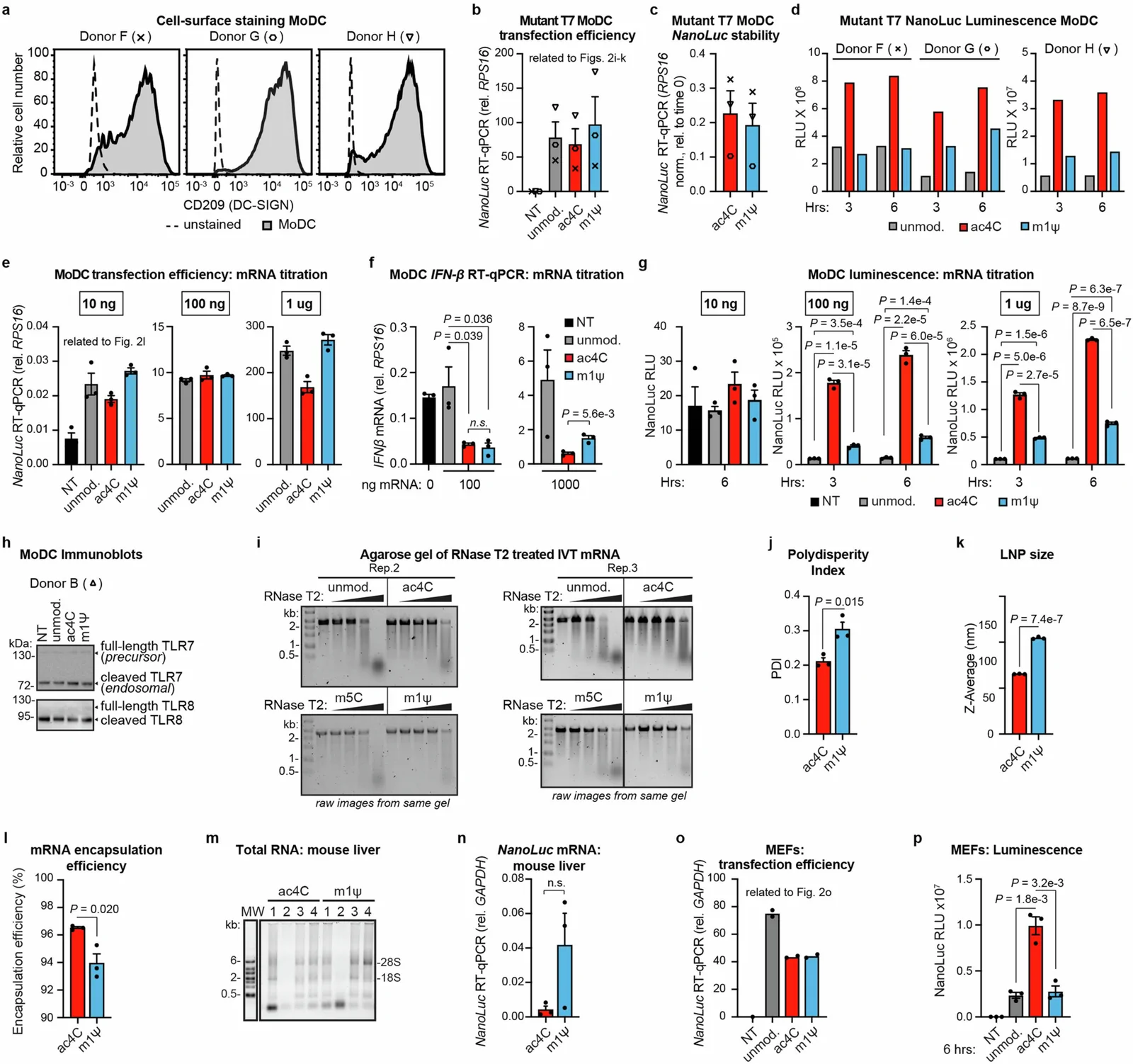
ac^4^C enhances *NanoLuc* translation across diverse cellular contexts independently of immune activation. **a**, FACS histograms of cell-surface CD209 (DC-SIGN) in primary human monocyte-derived dendritic cells (MoDCs) from three donors. **b**, *NanoLuc* mRNA levels in primary human MoDCs (Donors F–H) immediately after 5-min transfection with *NanoLuc* mRNA synthesized using G47A+884G mutant T7 RNA polymerase and LNP washout (t = 0), measured by RT-qPCR and normalized to *RPS16* (n = 3). **c**, Fraction *NanoLuc* mRNA remaining at the indicated times post-transfection in MoDCs, normalized to *RPS16* and time zero, from b (n = 3). **d**, Individual NanoLuc luminescence measurements in primary human MoDCs related to Fig. 2j at the indicated times following IVT mRNA transfection. **e**, *NanoLuc* mRNA levels in primary human MoDCs immediately after 5-min transfection with the indicated amounts of IVT mRNA and LNP washout (t = 0), measured by RT-qPCR and normalized to *RPS16* (n = 3). **f**, *IFNβ1* RT-qPCR in MoDCs 6 h post-transfection with 100 ng or 1000 ng *NanoLuc* IVT mRNA from e, normalized to *RPS16* (n = 3; two-tailed unpaired Student’s t-test). **g**, NanoLuc luminescence in MoDCs from e (n = 3; two-tailed unpaired Student’s t-test). **h**, Immunoblot of TLR7 and TLR8 in primary human MoDCs, showing full-length and cleaved (endosomal, active) forms. **i**, Denaturing agarose gel electrophoresis of additional replicates related to Fig. 2k following digestion of IVT mRNA with RNase T2. **j**, Polydispersity index (PDI) of LNPs encapsulating ac^4^C- or m^1^Ψ-modified *NanoLuc* mRNA, measured by dynamic light scattering (DLS) (n = 3; two-tailed unpaired Student’s t-test). **k**, Average diameter of LNPs encapsulating ac^4^C- or m^1^Ψ-modified *NanoLuc* mRNA prior to tail-vein injection in 6–8-week-old female BALB/c mice, measured by DLS (n = 3; two-tailed unpaired Student’s t-test). **l**, Encapsulation efficiency of *NanoLuc* mRNA containing ac^4^C or m^1^Ψ modifications (n = 3; two-tailed unpaired Student’s t-test). **m**, Denaturing agarose gel electrophoresis of total RNA isolated from mouse livers 24 h after injection of *NanoLuc* IVT mRNA–LNPs. Samples from four mice injected with ac^4^C-modified and four mice injected with m^1^Ψ-modified *NanoLuc* mRNA are shown. **n**, *NanoLuc* mRNA levels in mouse liver samples from m, measured by RT-qPCR and normalized to *GAPDH* (n = 3 mice; two-tailed unpaired Student’s t-test; n.s., not significant). **o**, *NanoLuc* mRNA levels in primary mouse embryonic fibroblasts (MEFs) immediately after 5-min transfection with *NanoLuc* IVT mRNA and LNP washout (t = 0), measured by RT-qPCR and normalized to *GAPDH* (n = 2). **p**, NanoLuc luminescence in MEFs at 6 h post-transfection (n = 3; two-tailed unpaired Student’s t-test). Data are mean ± s.e.m.; n = independent biological replicates.

MoDCs are particularly sensitive to dsRNA, which engages protein kinase R (PKR) to phosphorylate eIF2α and repress translation, and activates Toll-like receptor 3 (TLR3) to drive type I interferon responses^6^. To assess whether residual immune activation contributes to the translational advantage of ac^4^C, we attenuated dsRNA signalling using two orthogonal approaches. Pharmacological-mediated inhibition^21^ of PKR equalized p-eIF2α levels across conditions. However, ac^4^C-modified mRNA still produced the most NanoLuc protein, including relative to m^5^C and double-modified mRNAs (Extended Data Fig. 3i,j). Transfection of IVT mRNAs synthesized with the dsRNA-minimizing mutant T7 polymerase similarly eliminated differences in immune activation between ac^4^C and m^1^Ψ, whereas ac^4^C maintained higher NanoLuc protein expression across donor samples (Fig. 2h–j and Extended Data Fig. 4a–d).

To further disentangle immune signalling from translation, we titrated *NanoLuc* mRNA across a 1,000-fold range in MoDCs and identified a dose (100 ng) that supported strong protein expression without detectable *IFNB1* induction (Extended Data Fig. 4e,f). At this subimmunostimulatory dose, ac^4^C conferred an even greater relative increase in translation compared with m^1^Ψ than at standard input levels (Extended Data Fig. 4g). These findings establish that ac^4^C suppresses immune responses to IVT mRNA while enhancing translation through mechanistically uncoupled processes.

A principal mechanism by which uridine modifications limit immune responses to IVT mRNA is by reducing signalling through the uridine-sensing receptors TLR7 and TLR8. Recent work has shown that Ψ and m^1^Ψ achieve this effect by decreasing RNase-T2-mediated cleavage^22,23^. Endosomal TLR7 and TLR8 were highly expressed in MoDCs and unaltered by transfection (Extended Data Fig. 4h). Given the similarly low interferon induction with ac^4^C, we asked whether this cytidine modification likewise affects RNA cleavage. In vitro cleavage assays revealed comparable resistance to RNase T2 for ac^4^C and m^1^Ψ, whereas m^5^C displayed intermediate sensitivity (Fig. 2k,l and Extended Data Fig. 4i). Thus, the similar suppression of immune responses conferred by ac^4^C and m^1^Ψ probably reflects a shared mechanism, whereas their divergent effects on protein output arise from differences in translation itself.

Finally, we assessed whether the translational advantage of ac^4^C extends to in vivo conditions and to non-immune primary cells. Intravenous delivery of FIII-7 LNP-encapsulated *NanoLuc* mRNA into BALB/c mice^24–26^ led to an approximately 3.5-fold increase in hepatic luminescence for ac^4^C compared with m^1^Ψ at 24 h (Fig. 2m,n). LNP properties were comparable across modifications, and residual hepatic m^1^Ψ mRNA levels exceeded those of ac^4^C. This result excludes differential delivery as an explanation for the observed outcomes (Extended Data Fig. 4j–n). A similar advantage was observed in mouse embryonic fibroblasts (MEFs), for which ac^4^C led to increased NanoLuc protein levels relative to m^1^Ψ despite similar transfection efficiencies (Fig. 2o,p and Extended Data Fig. 4o,p). Together, these observations establish that ac^4^C suppresses immune activation while supporting enhanced protein output across therapeutically relevant contexts.

## Modifications increase ribosome density

Enhanced protein synthesis from ac^4^C-modified mRNA compared with m^1^Ψ-modified mRNA, despite comparable immune suppression, pointed to a direct influence of nucleotide modification on translation dynamics. To investigate this aspect at the level of individual transcripts, we used single-molecule imaging of nascent peptides (SINAPs), which measures translation specifically at mRNAs that have successfully reached the cytoplasm. This approach avoids artefacts from differences in endosomal escape that confound bulk measurements^27–31^.

**Fig. 3:**
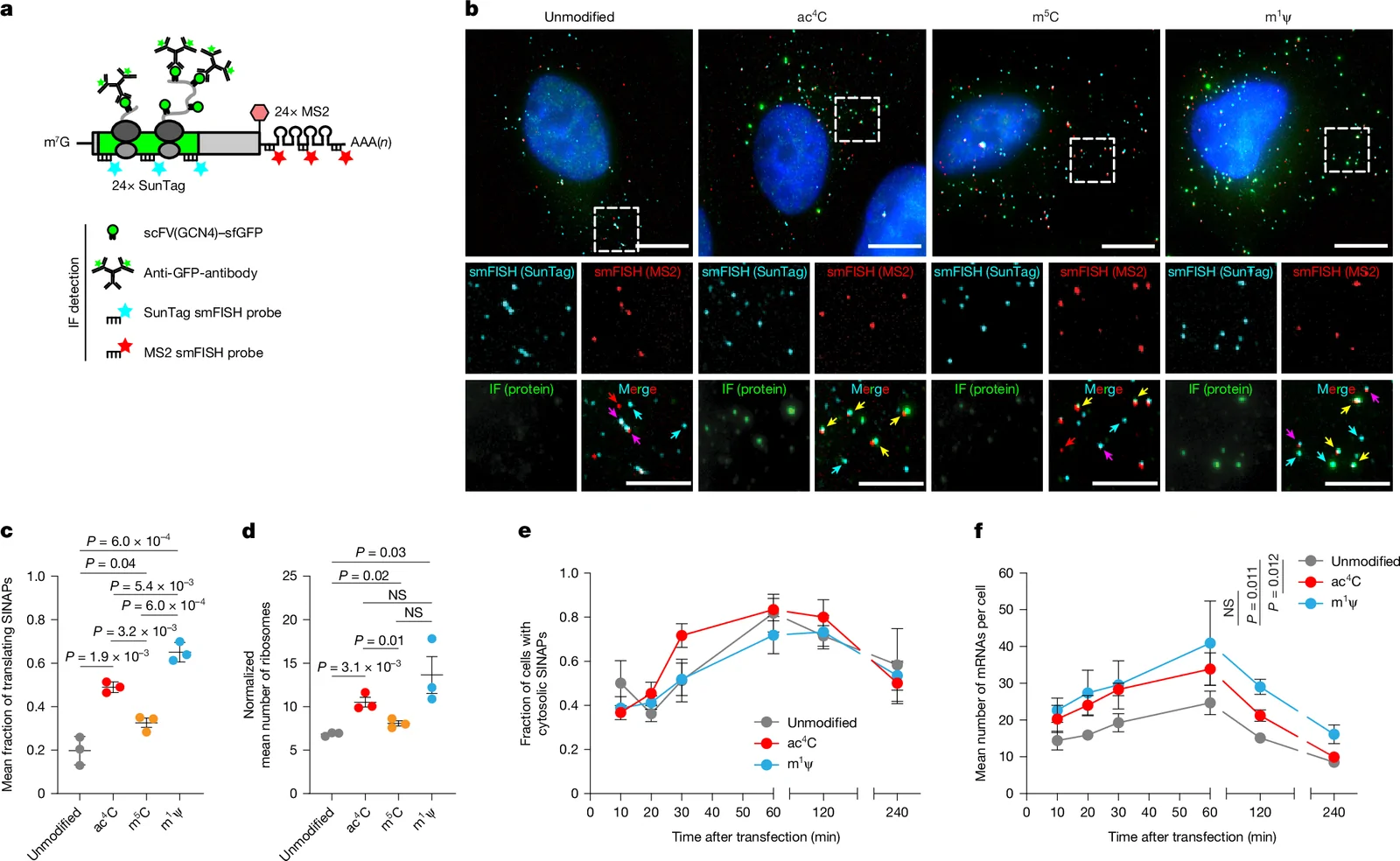
Single-molecule translation imaging reveals increased translational efficiency with modified mRNAs. **a**, Schematic of three-colour smFISH–IF of the SINAPs mRNA reporters. **b**, Representative smFISH–IF images of U-2 OS cells transfected with SINAPs reporter mRNA. Dashed boxes indicate enlarged regions. Cyan, *SunTag* smFISH; red, *MS2* smFISH; green, GFP IF. Yellow, red, cyan and magenta arrows indicate translating intact, *MS2*-alone, *SunTag*-alone and non-translating intact RNAs respectively. Scale bars, 10 μm (top) or 5 μm (bottom). **c**–**f**, Quantification of SINAPs translation. **c**, Mean fraction of translating mRNAs per cell per biological replicate. **d**, Mean number of ribosomes per translating mRNA calculated as the TLS signal normalized by a single mature protein in the same cell, showing the average per biological replicate. **e**, Fraction of cells containing cytosolic (endosome-released) SINAPs mRNAs (average of three biological replicates over time). **f**, Number of cytosolic SINAPs mRNAs per cell (average of three biological replicates over time). *n* = 160–400 cells examined over 3 independent experiments; two-tailed unpaired Student’s *t*-test, *P* values in** f** were combined using Fisher’s method.

The SINAPs reporter consists of an amino-terminal SunTag epitope array that recruits constitutively expressed single-chain variable fragment antibodies fused to superfolder GFP (scFv–sfGFP) to nascent peptides as they emerge from the ribosome exit channel to mark active translation sites (TLSs). A carboxy-terminal auxin-inducible degron minimizes diffusive background by degrading mature protein, and MS2-binding sites (MBSs) in the 3′ UTR recruit stably expressed fluorescent MS2 coat protein (MCP–RFP), which enables simultaneous visualization of individual transcripts and their translation (Fig. 3a).

**Extended Data Fig. 5:**
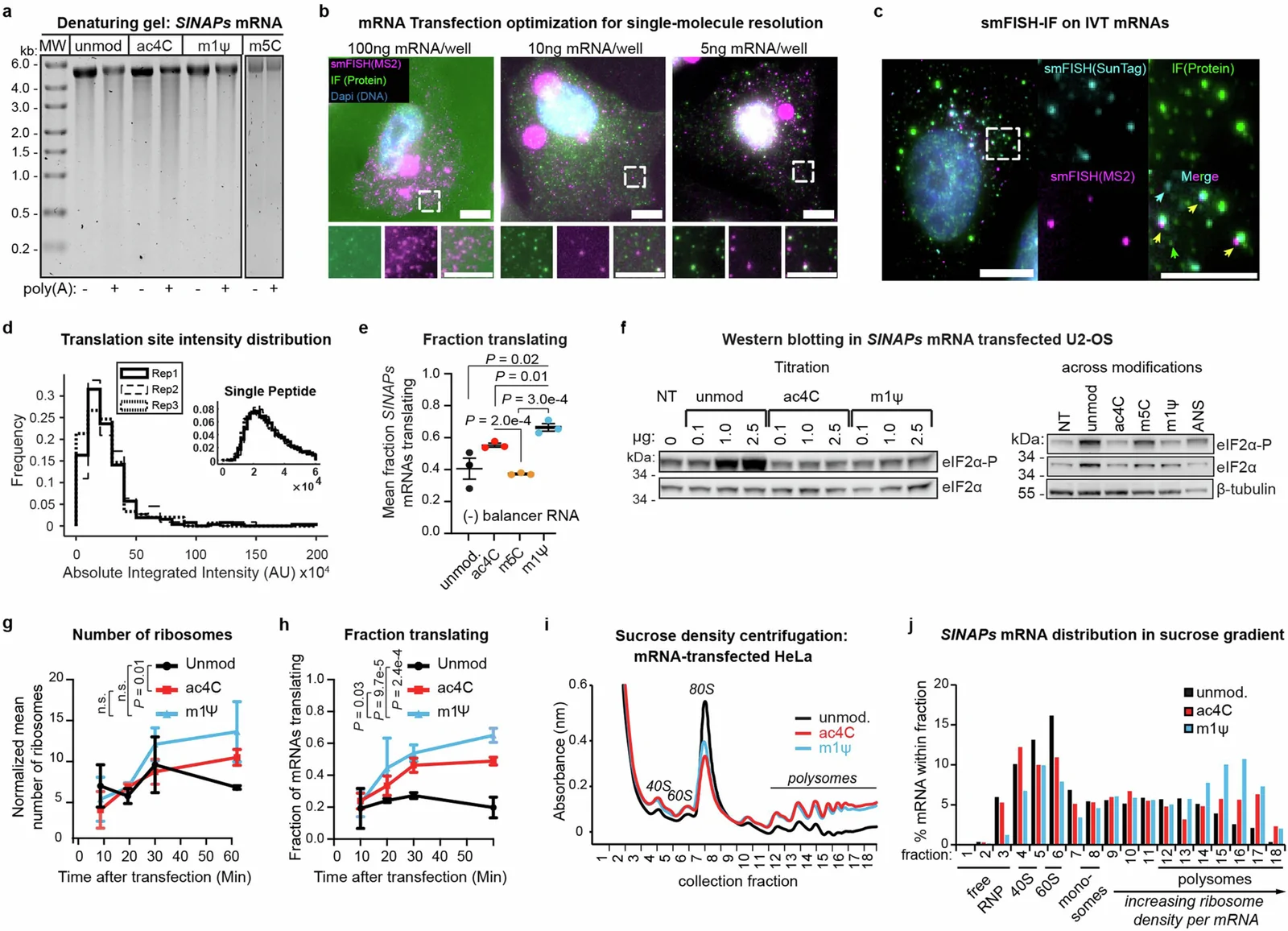
Single-molecule imaging of translation. **a**, Denaturing agarose gel electrophoresis of IVT SINAPs reporter mRNA before (–) and after (+) polyadenylation. Polyadenylated reporter mRNA was used for all SINAPs experiments (n = 3 independent mRNA preparations). **b**, Optimization of mRNA transfection conditions for single-molecule detection of mRNAs and translated proteins. Transfection of 5 ng total mRNA per well in a 24-well plate yielded well-defined smFISH signals and distinguishable mature peptides for IF normalization (n = 3 independent biological replicates). Scale bars, 10 μm (top) and 5 μm (bottom). **c**, Representative smFISH–IF image using two FISH probes and one IF channel. Dashed box is enlarged at right. Cyan, *SunTag* smFISH; magenta, *MS2* smFISH; green, immunofluorescence for protein. Arrows indicate translating mRNA (yellow), single mature protein (green), and non-colocalized *SunTag* RNA (cyan). Scale bars, 10 μm (left) and 5 μm (right). **d**, Distributions of integrated intensities for translation sites and single mature proteins (inset), demonstrating reproducibility across three biological replicates. Intensity values are shown in arbitrary units prior to normalization to single proteins. **e**, Average fraction of translating mRNAs per cell following transfection without mRNA balancer. Symbols represent mean per biological replicate (n = 160-400 cells examined over 3 independent experiments; two-tailed unpaired Student’s t-test compared to ac^4^C-modified mRNA). **f**, Western blot in U-2 OS cells transfected with varying amounts of IVT SINAPs reporter mRNA (left) or with SINAPs reporter mRNAs containing different nucleotide modifications (right). ANS, anisomycin positive control. **g**, Normalized number of ribosomes/mRNA/cell over time. Symbols represent the mean per biological replicate (n = 160-400 cells examined over 3 independent experiments; two-tailed unpaired Student’s t-test combined using Fisher’s method). **h**, Fraction of mRNAs translating per cell over time. Symbols represent the mean per biological replicates (n = 160-400 cells examined over 3 independent experiments; two-tailed unpaired Student’s t-test combined using Fisher’s method). **i**, Polysome profiles from HeLa cells 1.5 h post-transfection with SINAPs reporter mRNA, separated on 10–45% sucrose gradients (absorbance at 254 nm). **j**, Distribution of SINAPs reporter mRNA across the polysome gradient determined by RT-qPCR (n = 2). Data are mean ± s.e.m.; n = independent biological replicates; significant values shown.

IVT SINAPs reporter mRNAs were co-transcriptionally capped, post-transcriptionally polyadenylated and transfected into U-2 OS cells (Extended Data Fig. 5a and Supplementary Table 1). Translation was examined in fixed cells by single-molecule fluorescence in situ hybridization combined with immunofluorescence (smFISH–IF)^32^, with nascent peptides detected by anti-GFP immunostaining rather than direct GFP fluorescence to bypass fluorophore maturation artefacts. Signal amplification by secondary antibodies enabled the detection of individual peptides released as dim puncta that are distinct from the brighter mRNA colocalizing to the TLS. To maintain single-molecule resolution at physiologically relevant mRNA loads (Extended Data Fig. 4f,g), SINAPs mRNAs were co-transfected with a ‘balancer’ mRNA with the same nucleotide modification (Extended Data Fig. 5b). Analysis was restricted to intact transcripts identified by dual-colour smFISH against *SunTag* and *MS2* sequences, excluding abortive or partially degraded RNAs (Fig. 3b and Extended Data Fig. 5c). TLS intensities were normalized to single-peptide signals in the same cell to estimate ribosome occupancy (Methods and Extended Data Fig. 5d).

Under these conditions, ac^4^C-modified and m^1^Ψ-modified mRNAs were more frequently translated and loaded more nascent peptides per transcript than unmodified and m^5^C-modified mRNAs (Fig. 3c,d). Notably, all reporters were efficiently translated at low doses without a balancer, a result consistent with the modification-tuned repression of initiation through p-eIF2α at high RNA loads (Extended Data Fig. 5e,f). These findings align with evidence that nucleotide modifications enhance IVT mRNA translation in part by mitigating stress-linked initiation repression^2,33^. Moreover, the results support SINAPs as a robust approach for measuring translation dynamics in cells.

However, these measurements did not explain the translational advantage of ac^4^C in bulk assays, as m^1^Ψ produced a higher fraction of translating mRNAs and greater ribosome occupancy (Fig. 3c,d). Time-course smFISH–IF experiments further established that ac^4^C does not gain an edge through enhanced endosomal escape. Although ac^4^C modestly increased the fraction of cells with cytosolic SINAPs reporter mRNA at early time points, overall cytosolic mRNA counts per cell and translation engagement were similar for ac^4^C and m^1^Ψ (Fig. 3e,f and Extended Data Fig. 5g,h). Polysome profiling of transfected HeLa cells provided orthogonal evidence. That is, modified mRNA was associated more strongly with heavy polysomes than unmodified transcripts, with m^1^Ψ exhibiting the greatest enrichment (Extended Data Fig. 5i,j). Together, these fixed-cell analyses confirmed that there is increased ribosome engagement for modified transcripts relative to unmodified mRNA. However, the mechanistic basis of the translational advantage of ac^4^C remained unresolved.

## m^1^Ψ slows elongation and induces RQC

**Fig. 4:**
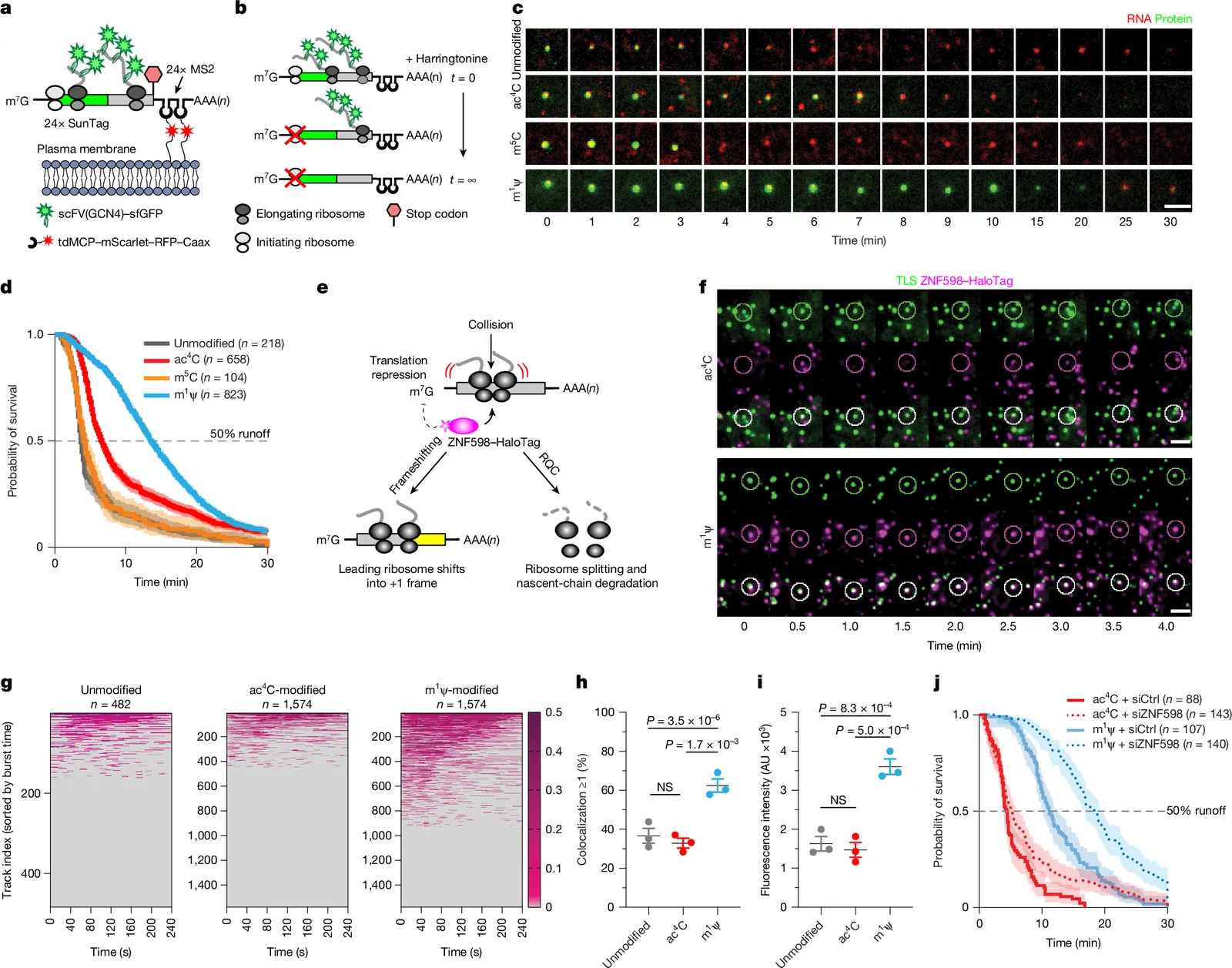
m^1^Ψ slows elongation and increases ribosome collision-dependent RQC engagement. **a**, Schematic of live-cell SINAPs. **b**, Schematic of ribosome runoff. Harringtonine blocks initiation, which allows elongating ribosomes to complete translation. The runoff time reflects the elongation rate. **c**, Representative snapshots from runoff imaging in U-2 OS cells transfected with SINAPs mRNA (Supplementary Video 1). Red, mRNA (tdMCP–mScarlet); green, TLSs (scFv–sfGFP). Scale bars, 2 μm. **d**, Survival curves of ribosome runoff for indicated mRNA modifications. Time zero corresponds to 1 min after harringtonine addition. Curves represent the fraction of mRNAs still translating at each time point calculated using the Kaplan–Meier method; shaded area, 95% confidence intervals (Greenwood’s formula). **e**, Schematic of ZNF598–HaloTag recruitment to collided ribosomes. **f**, Representative images of ZNF598–HaloTag recruitment to TLSs on ac^4^C-modified or m^1^Ψ-modified mRNAs (Supplementary Video 2). Green, TLSs (scFv–sfGFP); magenta, ZNF598–HaloTag (JFX650). Scale bars, 2 μm. **g**, Heatmaps of ZNF598–HaloTag recruitment to TLSs. *y *axis, individual mRNAs sorted by first colocalization event; *x* axis, imaging time. The colour bar indicates normalized ZNF598–HaloTag intensity. **h**,**i**, Quantification of ZNF598 recruitment. **h**, Fraction of TLSs with colocalized ZNF598 (≥1 event >10 s). **i**, Mean fluorescence intensity (arbitrary units (AU)) of colocalized ZNF598. Symbols depict the average per biological replicate; *n* = 10–20 cells examined over 3 independent replicates; one-way analysis of variance. **j**, Survival curves from ribosome runoff assays in cells treated with control (siCtrl) or *ZNF598*-targeting (siZNF598) siRNA, as in **d** (Supplementary Video 3).

To capture translation dynamics not accessible by fixed-cell imaging, we examined elongation in live cells using ‘ribosome runoff’^34^. In this approach, the translation inhibitor harringtonine is used to block new initiation events. Elongating ribosomes are then allowed to complete translation and ‘runoff’ the transcript^30^. SINAPs reporter mRNAs were transfected into U-2 OS cells constitutively expressing scFv–sfGFP and MCP–RFP–CAAX, which tethers mRNAs to the plasma membrane and enables long-term TLS tracking by total internal reflection fluorescence (TIRF) microscopy^35,36^ (Fig. 4a–c). For each translating mRNA, we measured the interval between harringtonine addition and TLS disappearance, then compiled these values into survival curves to infer elongation rates (Fig. 4d, Methods and Supplementary Video 1). Runoff times differed across modifications. That is, unmodified (median = 3.7 min) and m^5^C-modified (median = 4.2 min) mRNAs cleared most rapidly. By contrast, ac^4^C-modified mRNA showed intermediate kinetics (median = 6.7 min) and m^1^Ψ-modified mRNA exhibited a substantial delay (median = 13.7 min). Because runoff time scales inversely with elongation rate, the substantial elongation slowdown with m^1^Ψ provides a mechanistic basis for its paradoxically high ribosome occupancy despite its low protein yield.

**Extended Data Fig. 6:**
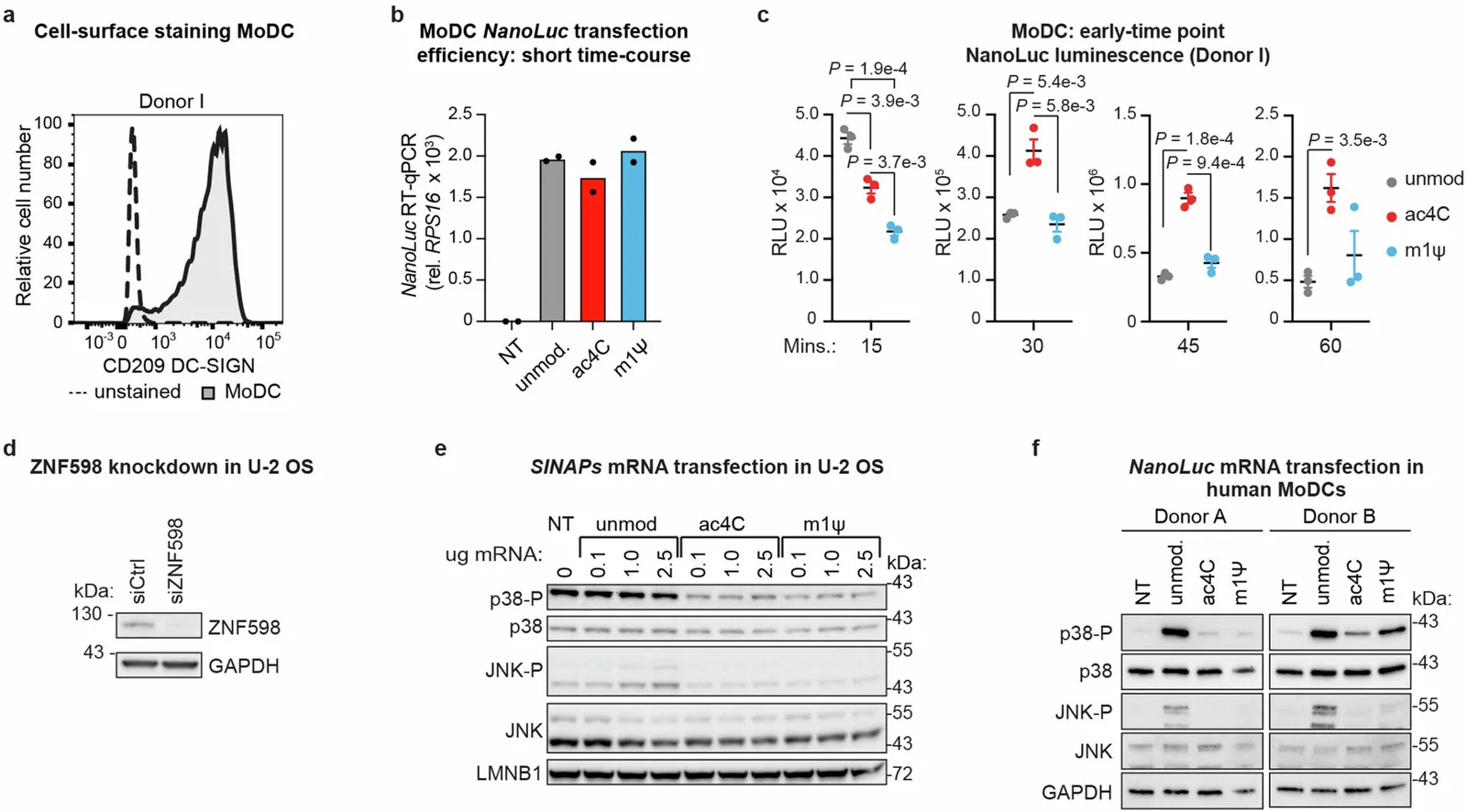
Detection of ribosome collisions in cells transfected with IVT mRNA. **a**, FACS histogram of cell-surface CD209 (DC-SIGN) in primary human MoDCs from Donor I. **b**, *NanoLuc* mRNA levels in MoDCs following transfection, measured by RT-qPCR (n = 2). **c**, NanoLuc luminescence in MoDCs at early time points post-transfection (n = 3; two-tailed unpaired Student’s t-test). **d**, Western blot of ZNF598 following knockdown in U-2 OS cells; GAPDH, loading control. **e**, Western blot of total and phosphorylated p38 (p-p38) and JNK (p-JNK) in U-2 OS cells transfected with SINAPs reporter mRNA containing the indicated nucleotide modifications, markers of ZAKα-dependent MAP kinase signaling downstream of ribosome collisions and RQC engagement; LMNB1, loading control (n = 3). **f**, Western blot of total and phosphorylated p38 (p-p38) and JNK (p-JNK) in primary human MoDCs transfected with *NanoLuc* IVT mRNA, markers of MAP kinase signaling downstream of ribosome collisions; GAPDH, loading control (n = 2). Data are mean ± s.e.m.; n = independent biological replicates.

Early NanoLuc time-course experiments in MoDCs supported this interpretation. Unmodified mRNA produced the highest luminescence at 15 min after transfection but was surpassed by ac^4^C at 30 min and subsequently by m^1^Ψ at 45 min, despite comparable transfection efficiencies (Extended Data Fig. 6a–c). This temporal ordering mirrored the runoff measurements. Unmodified and m^5^C-modified mRNAs elongated rapidly but were constrained by eIF2α-mediated initiation repression (Extended Data Fig. 5f). By contrast, the slow elongation of m^1^Ψ-modified mRNA promoted ribosome accumulation along the CDS, a result consistent with its enrichment in heavy polysome fractions (Extended Data Fig. 5j).

Slow elongation can generate ribosomal traffic jams that promote collisions^37^ and activate ribosome-associated quality control (RQC), which leads to ribosomal subunit splitting and nascent-peptide degradation^38^. To assess whether the slower elongation of m^1^Ψ is associated with increased collision propensity, we monitored recruitment levels of ZNF598, an E3 ligase that ubiquitinates collided ribosomes^39^, to IVT SINAPs reporter mRNA in U-2 OS cells, with both *ZNF598* alleles endogenously tagged with HaloTag^40^ (Fig. 4e). Co-expression of scFv–sfGFP and MCP–RFP–CAAX enabled simultaneous visualization of translation, mRNA and ZNF598 binding by TIRF microscopy^40^ (Fig. 4f, Methods and Supplementary Video 2). Nearly two-thirds of m^1^Ψ-modified transcripts colocalized with ZNF598 compared with roughly one-third of unmodified or ac^4^C-modified mRNAs (Fig. 4g,h). ZNF598–HaloTag fluorescence intensity on m^1^Ψ transcripts was also increased by approximately twofold (Fig. 4i), which indicated more frequent and longer-lived collisions.

To directly link RQC activity to altered elongation dynamics, we depleted *ZNF598* through short interfering RNA (siRNA) (Extended Data Fig. 6d). As a core RQC component, ZNF598 promotes the removal of collided ribosomes, and its depletion extends the time required to resolve the resulting roadblocks^35^. Accordingly, ZNF598 depletion further prolonged the already slow runoff time of m^1^Ψ-modified mRNAs, whereas ac^4^C runoff was unaffected (Fig. 4j and Supplementary Video 3). These results suggest that m^1^Ψ increases collision-driven RQC engagement relative to ac^4^C, which affects protein output through nascent-peptide degradation and potential feedback inhibition of initiation^41–43^. Collision levels on m^1^Ψ-modified transcripts nevertheless remained below the threshold for downstream signalling^44,45^ (Extended Data Fig. 6e,f), which suggests that there is effective buffering by RQC.

## Enhanced translation fidelity with ac^4^C

In addition to engaging RQC, ribosome collisions can stimulate +1 frameshifting^46^, which enables elongation to resume in an alternative reading frame until a premature stop codon is reached. The resulting truncated products reduce functional protein yield and may act as neoantigens capable of triggering unintended immune responses. Consistent with the substantial ribosome collisions observed on m^1^Ψ-modified mRNA, a recent study^3^ reported elevated +1 frameshifting with m^1^Ψ-modified IVT mRNA and detected immune responses to frameshifted peptides in recipients of mRNA-based COVID-19 vaccines.

**Fig. 5:**
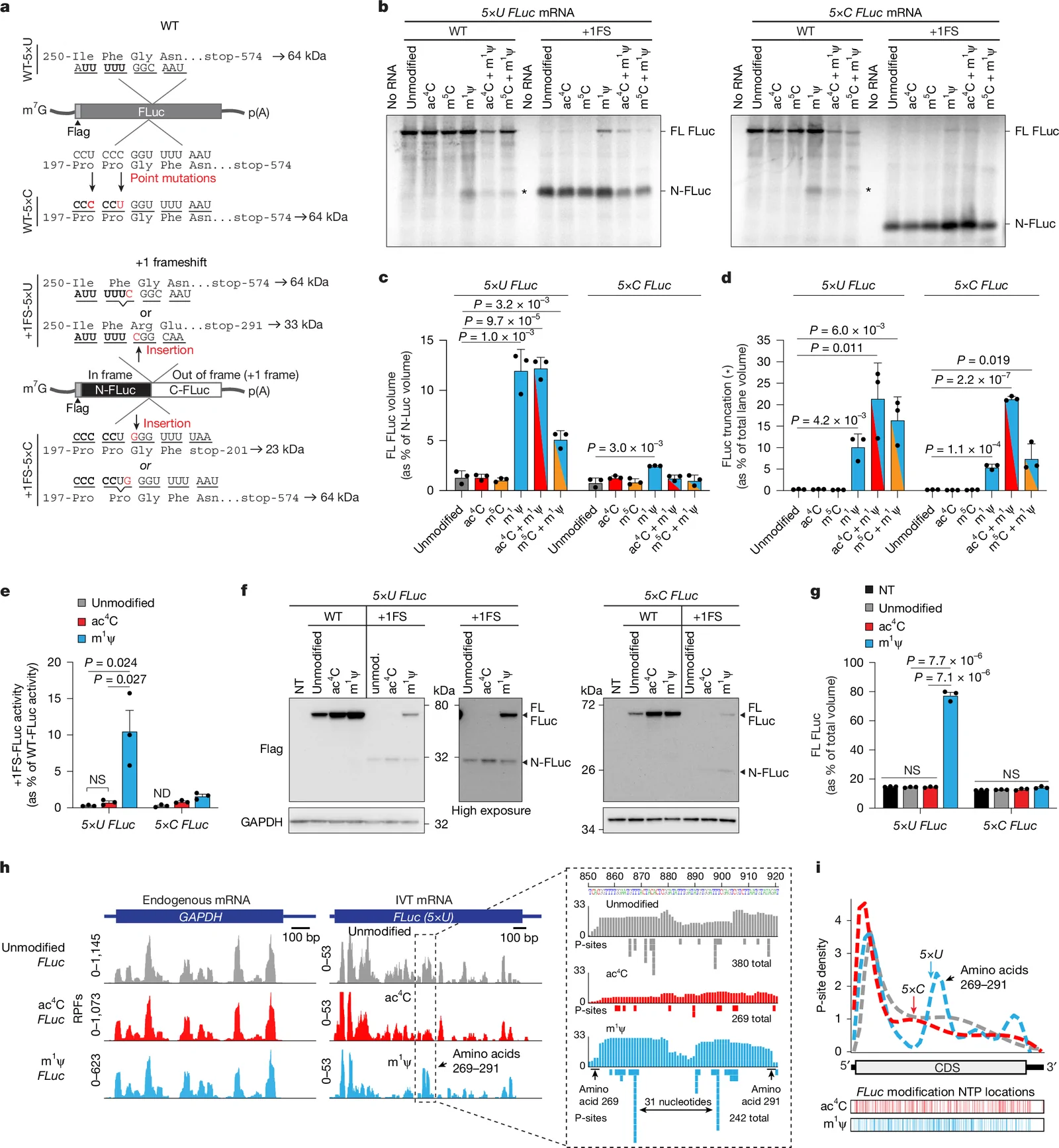
m^1^Ψ increases +1 frameshifting relative to ac^4^C in IVT mRNA. **a**, Schematic of IVT mRNAs containing 5×U or 5×C motifs, encoding N-terminally Flag-tagged FLuc from WT or +1 frameshifted (+1FS) translation. **b**–**d**, *FLuc* translation in RRLs. **b**, Autoradiograph of labelled translation products 80 min after *FLuc* mRNA addition, with full-length (FL) and N-terminal truncation (N-FLuc) products indicated. **c**, Quantification of frameshifting efficiency, expressed as FL/N-FLuc signals.** d**, Quantification of WT-specific truncation (indicated by the asterisk in **b**) relative to total signal. *n* = 3; two-tailed unpaired Student’s *t*-test compared with unmodified *FLuc*. Asterisk indicates a truncated product of around 30 kDa in size. **e**–**g**, *FLuc* translation in transfected HeLa cells. **e**, Luminescence at 6 h after transfection comparing mRNAs of the +1FS and WT reporters. ND, not detected. **f**, Immunoblot of Flag-tagged FLuc at 6 h, with GAPDH as the loading control. **g**, Quantification of frameshifting from **f**, expressed as the FL protein signal relative to total protein. *n* = 3; two-tailed unpaired Student’s *t*-test. **h**,**i**, Ribo-seq of *FLuc*-transfected HeLa cells. **h**, RPF density across mRNAs for GAPDH and the WT-5×U FLuc reporter. **i**, P-site density across the *FLuc* CDS relative to ac^4^C and m^1^Ψ positions, with arrows indicating 5×C and 5×U motifs. Data are the mean ± s.e.m.; *n* indicates independent biological replicates; only significant values are shown.

**Extended Data Fig. 7:**
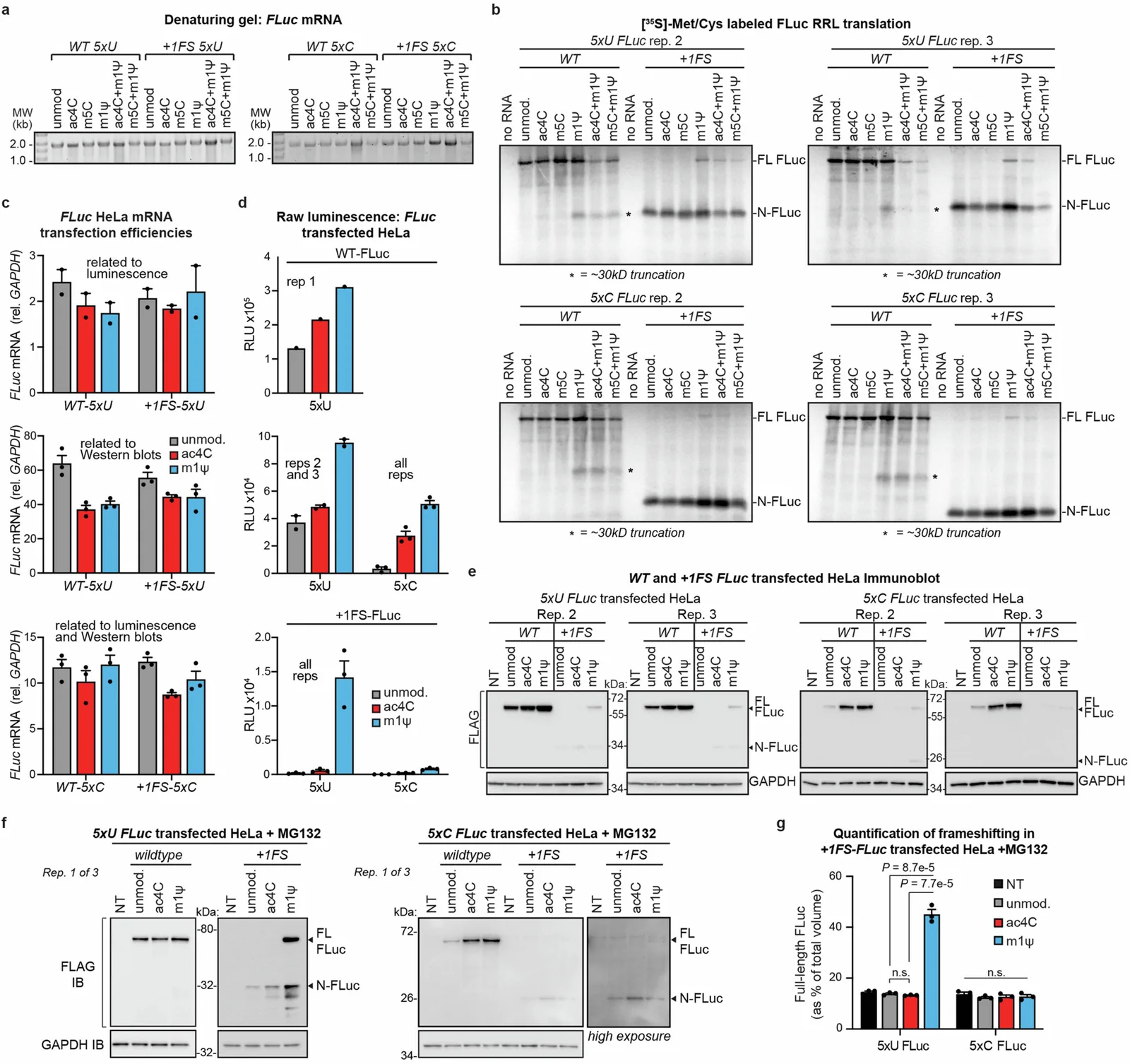
+1 ribosomal frameshifting with IVT mRNA. **a**, Denaturing agarose gel electrophoresis of IVT WT-FLuc and +1FS-FLuc reportermRNA synthesized using G47A + 884 G mutant T7 RNA polymerase. **b**, Autoradiograph of additional replicates related to Fig. 5b, depicting [^35^S]-methionine/cysteine–labeled translation products 80 min after addition of *FLuc* mRNA to rabbit reticulocyte lysates (RRL). **c**, WT-FLuc and +1FS-FLuc reporter mRNA levels in HeLa cells immediately after 5-min transfection and LNP washout (t = 0), measured by RT-qPCR and normalized to *GAPDH* (n = 3). **d**, Absolute luminescence in HeLa cells transfected with WT-FLuc or +1FS-FLuc reporter mRNA related to Fig. 5e at the indicated times post-transfection (n = 3). **e**, Western blot of additional replicates related to Fig. 5f, depicting FLAG-tagged FLuc 6 h post-transfection of WT-FLuc or +1FS-FLuc reporter mRNA in HeLa. Arrows indicate full-length and N-terminally truncated FLuc. **f**, Representative Western blot of FLAG-tagged FLuc 6 h post-transfection of WT-FLuc or +1FS-FLuc reporter mRNA in HeLa treated with the proteasome inhibitor MG132 (n = 3). **g**, Frameshifting quantified from f, expressed as full-length (FL) signal relative to total signal. NT, non-transfected (n = 3; two-tailed unpaired Student’s t-test). Data are mean ± s.e.m.; n = independent biological replicates.

Although ac^4^C modestly slowed elongation relative to unmodified mRNA, it did not increase ZNF598 recruitment. This finding prompted us to assess whether this more limited slowdown affects ribosomal frameshifting. To this end, we generated N-terminally Flag-tagged firefly luciferase (FLuc) reporters. A WT construct containing five tandem uridines (WT-5×U) was compared with a frameshift-reporting variant containing a single-nucleotide insertion immediately downstream (+1FS-5×U) (Fig. 5a and Extended Data Fig. 7a). In the +1FS reporter, full-length FLuc is produced only after +1 frameshifting, whereas failure to frameshift generates an N-terminal truncation product. Analogous 5×C reporters (WT-5×C and +1FS-5×C) were generated to model a cytidine-rich context (Fig. 5a and Extended Data Fig. 7a). All mRNAs were synthesized using mutant T7 polymerase to minimize dsRNA by-products, and RNA sequencing (RNA-seq) confirmed comparably low error rates across conditions (Supplementary Table 2).

In RRLs, full-length FLuc was the predominant product from WT reporter mRNAs, whereas N-terminal truncations were the major products from +1FS reporters (Fig. 5b and Extended Data Fig. 7b). High full-length FLuc production from +1FS-5×U occurred only with m^1^Ψ-modified transcripts, whereas ac^4^C-modified, m^5^C-modified and unmodified transcripts produced minimal signals (Fig. 5b,c). Inclusion of m^1^Ψ in double-modified mRNAs restored frameshifting, which indicated that this effect is intrinsic to m^1^Ψ. By contrast, neither ac^4^C nor m^5^C increased frameshifting above background in the +1FS-5×C reporter (Fig. 5b,c). Thus, although ac^4^C slows elongation, this effect is insufficient to promote frameshifting at C-rich sequences. Instead, frameshifting remained highest with m^1^Ψ, a result consistent with its broader propensity to induce collision-dependent decoding errors. A truncated species indicative of ribosome stalling was also detected from WT transcripts in the presence of m^1^Ψ (Fig. 5b,d).

These findings were recapitulated in transfected HeLa cells, which showed comparable mRNA uptake across conditions (Extended Data Fig. 7c). Luminescence from +1FS-5×U *FLuc* mRNAs reached around 10% of WT in the presence of m^1^Ψ but remained at background levels with unmodified and ac^4^C-modified transcripts (Fig. 5e and Extended Data Fig. 7d). By contrast, frameshifting from 5×C reporter mRNAs remained minimal, with only a modest increase in the presence of m^1^Ψ (Fig. 5e). Immunoblotting, performed with and without proteasome inhibition, confirmed these trends. That is, full-length FLuc from +1FS-5×U was detected only with m^1^Ψ, whereas truncated products predominated in all other conditions (Fig. 5f,g and Extended Data Fig. 7e–g). These data indicate that the substantial frameshifting observed with m^1^Ψ at U-rich sequences is not reproduced by ac^4^C at C-rich sequences.

**Extended Data Fig. 8:**
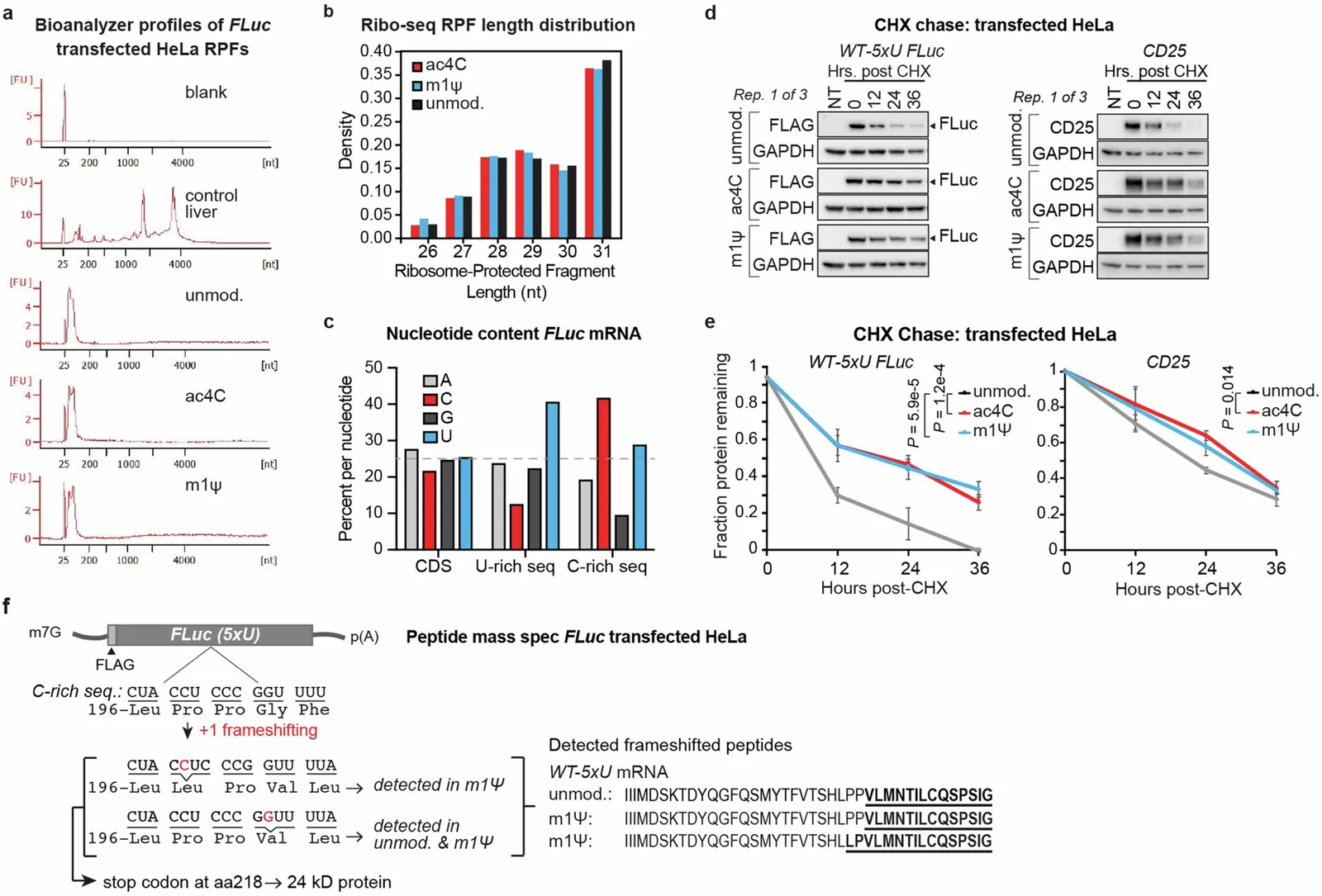
Ribosome profiling and frameshifting analyses. **a**, Bioanalyzer profiles of ribosome-protected fragments (RPFs) generated following translation of IVT WT-5xU *FLuc* mRNA in HeLa cells. **b**, Distribution of RPF lengths in Ribo-seq data from WT-5xU *FLuc*-transfected HeLa cells, from a. **c**, Nucleotide composition across the *FLuc* coding sequence compared with the U-rich region surrounding the pause site detected in m^1^Ψ-modified *FLuc* ribosome profiling data and the C-rich region encompassing the 5xC motif. **d**, Western blot of FLAG-tagged WT-5xU FLuc and CD25 at the indicated times following cycloheximide (CHX) treatment to halt translation, 4 h after mRNA transfection in HeLa cells; GAPDH, loading and protein turnover control. **e**, Densitometric quantification of FLAG-tagged FLuc and CD25 remaining at the indicated times after CHX treatment, normalized to time zero (mean ± s.e.m., n = 3 independent transfections; *P*-values combined using Fisher’s method from two-tailed unpaired Student’s t-tests). **f**, Peptides resulting from +1 frameshifting at the C-rich motif in WT-5xU* FLuc* mRNA-transfected HeLa cells, detected by mass spectrometry.

To connect these observations with ribosome dynamics, we performed ribosome profiling in HeLa cells transfected with WT-5×U *FLuc* mRNAs (Supplementary Table 3). Ribosome-protected fragment (RPF) recovery, read-length distributions and profiles across endogenous transcripts were comparable between conditions (Fig. 5h and Extended Data Fig. 8a,b). Unmodified and ac^4^C-modified *FLuc* produced similar CDS RPF distributions, with no evidence of ribosome stalling despite the modest elongation slowdown with ac^4^C. By contrast, m^1^Ψ-modified *FLuc* showed substantial ribosome accumulation at a U-rich region spanning amino acids 269–291 downstream of the 5×U sequence, with reduced density elsewhere (Fig. 5h). This region is notable on two counts. First, amino acid 291 corresponds to the encoded premature stop codon of the mRNA for the +1FS-5×U reporter, which indicated that the similarly sized truncation product observed in m^1^Ψ-modified WT FLuc RRL reactions probably results from ribosome stalling at this sequence (Fig. 5b). Second, P-site mapping^47^ revealed two enrichment peaks separated by around 31 nucleotides in this interval, a result consistent with stacked or collided ribosomes (Fig. 5h,i). Notably, uridine content across the CDS of the *FLuc* reporter averaged at 25.6% but increased to about 41% in this RPF-dense region, thereby potentially generating a local velocity mismatch under m^1^Ψ-mediated conditions (Fig. 5h,i and Extended Data Fig. 8c). This finding aligns with reports of increased dwell times at m^1^Ψ-containing NUN and NNU dicodons^9^, four of which occur in our settings (Supplementary Table 1). Comparable pausing was absent at cytidine-rich sequences in ac^4^C-modified mRNA, including the 5×C region (Fig. 5i and Extended Data Fig. 8c).

Despite clear ribosome pausing, truncated products were not detected from WT *FLuc* following proteasome inhibition in cells (Fig. 5h and Extended Data Fig. 7f). Likewise, protein stability was comparable across ac^4^C- and m^1^Ψ-modified mRNAs (Extended Data Fig. 8d,e), and mass spectrometry did not detect frameshifting at the 5×U site (Extended Data Fig. 8f). This finding indicated that translation remains largely accurate in optimized coding contexts. However, +1 frameshifted peptides were identified at the CCU–CCC dicodon at amino acids 197–198 in a C-rich region in samples translated from m^1^Ψ-modified or unmodified *FLuc* mRNA. This result provides a rationale for the low-level frameshifting observed with m^1^Ψ in the +1FS-5×C reporters (Fig. 5b-f and Extended Data Fig. 8f). These findings are consistent with the known susceptibility of proline-rich sequences to frameshifting^48,49^. By contrast, the absence of frameshifting in this region with ac^4^C-modified transcripts raises the possibility that enhanced Watson–Crick pairing^12^ confers a fidelity advantage in sequences prone to P-site slippage.

Together, our findings establish elongation dynamics as a central determinant of translational output from synthetic mRNAs, with nucleotide modifications defining distinct pathways that shape ribosome collisions and quality control. We conceptualize these effects through a ‘braking upon modified position’ (BUMP) model. In this scenario, slowed elongation at modified nucleotides increases the likelihood that trailing ribosomes encounter paused leaders, which generates transient traffic along the mRNA. The modifications examined here occupy distinct positions along this spectrum. Unmodified mRNA elongates rapidly but induces p-eIF2α, which limits protein synthesis through initiation repression. ac^4^C modestly slows elongation while avoiding collision-prone ribosome stacking, which supports efficient and accurate ribosome transit. By contrast, m^1^Ψ slows elongation more severely, which promotes ribosome stacking, RQC engagement and collision-induced +1 frameshifting.

The molecular basis for these differences probably lies in their distinct effects in the ribosomal decoding centre. The *N*^4^-acetyl group of ac^4^C enforces a cytidine conformation that stabilizes Watson–Crick base pairing with guanosine to enhance decoding efficiency without perturbing ribosome geometry^12^. By contrast, m^1^Ψ is forced into an energetically unfavourable C2′-endo ribose conformation after translocation to the P-site, which provides a mechanistic basis for the observed increased ribosome dwell times at m^1^Ψ-modified codons^4,9^. These findings reflect a broader principle: codon–anticodon interactions and tRNA abundance have been co-optimized over evolutionary timescales to balance elongation speed, accuracy and co-translational folding^50^. Uniform substitution of canonical nucleosides with chemically distinct analogues in synthetic mRNAs disrupts this equilibrium, which creates a decoding landscape for which no evolutionary tuning exists.

These chemical distinctions extend beyond the decoding centre to shape how modified transcripts are recognized by innate immune sensors. Despite modifying cytidine rather than uridine, ac^4^C achieved immune suppression comparable to m^1^Ψ, an unexpected result given that uridine substitution has been considered the primary determinant of TLR7 and TLR8 evasion. Our data suggest that this equivalence arises from shared resistance to RNase-T2-mediated cleavage, which limits the generation of uridine-rich fragments that activate endosomal sensors^22,23^. These findings expand the paradigm for immune evasion and demonstrate that chemically distinct modifications can converge on common functional outcomes through effects on RNA processing.

From a therapeutic perspective, these results refine how nucleotide modifications should be evaluated and used. m^1^Ψ remains the dominant clinical modification owing to its robust performance and established safety profile, and our data do not argue for its general replacement. Rather, ac^4^C provides a distinct balance of properties, including strong protein output, low immunogenicity and preservation of translational fidelity, which may be advantageous in applications that require accurate protein production. Conversely, m^1^Ψ may remain preferable in contexts when slower elongation is tolerated or beneficial. For example, in cases when reduced elongation rates can support co-translational protein folding or when immune activation is desired, such as vaccination^6,7^. However, the potential for neoantigen generation through frameshifting warrants consideration^3^.

Our study establishes that the functional output of synthetic mRNAs is governed not only by ribosome engagement but also by how productively and accurately ribosomes traverse a modified transcript. We propose that translational BUMPs, defined as localized disruptions in ribosome flow introduced by nucleotide modifications, provide a unifying framework for understanding how chemical changes to RNA are interpreted by the translation machinery. Incorporating this principle into mRNA design may facilitate the development of next-generation therapeutics with improved performance across diverse applications.

## Methods

### Ethics

Human peripheral blood was obtained from de-identified healthy donors through the NIH Clinical Center, Department of Transfusion Medicine, Research Blood Donor Program, under a protocol approved by the NIH Institutional Review Board (IRB no. 99CC0168). All donors provided written informed consent before participation.

### Cell culture

HeLa cells (American Type Culture Collection (ATCC), CCL-2) were cultured in DMEM (Thermo Fisher Scientific, 10313021) supplemented with 2 mM L-glutamine (Thermo Fisher Scientific, 25030164) and 10% bovine calf serum (BCS, HyClone, SH30073.03; DMEM-BCS). THP-1 cells (ATCC, TIB-202) were cultured in RPMI 1640 (Thermo Fisher Scientific, 21870092) supplemented with β-mercaptoethanol (55 mM, Sigma, M3148), 2 mM L-glutamine and 10% FBS (Seradigm, FBS, 97068-085, RPMI-primary). THP-1 cells were differentiated into M0 macrophages through the addition of phorbol 12-myristate 13-acetate (PMA, 162 nM, Sigma-Aldrich, P1585) with 1.5 × 10^6^ cells per 6-well plate (for protein) or 0.375 × 10^6^ cells per 12-well plate (for RNA and luminescence) for 16–24 h. THP-1-derived M0 cells were cultured in RPMI-primary medium for 24 h before transfection. HeLa and THP-1 cells were not authenticated. MEFs from a frozen cryovial were thawed and cultured in DMEM with 15% FBS and 1% l-glutamine. Primary monocytes were isolated from human peripheral blood using an EasySep Direct Human Monocyte Isolation kit (Stem Cell Technologies, 19669) according to the manufacturer’s instructions, followed by the addition of 25 mM HEPES (Quality Biological, 118-089-721), 50 ng ml^–1^ recombinant human IL-4 (Peprotech, 200-04) and 50 ng ml^–1^ recombinant human GM-CSF (Sigma-Aldrich, GF-304) in RPMI primary medium to generate MoDCs.

The SINAP technology relies on several auxiliary proteins: scFv–sfGFP to label the nascent peptides, MCP–RFP to label the RNA and the E3 ligase TIR1 from *Oryza sativa* (*Os*TIR1) for the auxin-inducible degron to deplete the mature proteins^29^. Two U-2 OS cell lines expressing these auxiliary proteins were used in this study: one for live-cell imaging (scFv–sfGFP, *Os*TIR1 (ref. ^51^) and MCP–RFP–CAAX), and the other for fixed-cell (scFv–sfGFP, *Os*TIR2 (ref. ^52^)) experiments. These U-2 OS cells (ATCC, HTB-96) were maintained in DMEM–FBS (Corning, 10-013-CM and Millipore Sigma, F4135), 100 U ml^–1^ penicillin and 100 µg ml^–1^ streptomycin (Millipore, P0781). Cells were cultured at 37 °C in a 5% CO_2_ incubator and passaged approximately every 3 days. Monthly mycoplasma contamination testing was performed to ensure sterility.

### Flow cytometry

The following antibodies were used for flow cytometry experiments: PE-conjugated anti-Hu/NHP CD25 antibody ((CD25-4E3), eBioscience, 12-0257-42), APC-conjugated anti-HuCD14 antibody ((61D3), eBioscience, 17-0149-42), PerCP/cyanine5.5-conjugated anti-human CD11c antibody ((3.9), BioLegend, 301624) and PE-conjugated anti-CD209 (DC-SIGN) antibody ((9E9A8), BioLegend, 330105). In brief, 0.1–1 × 10^6^ cells were stained in 100 µl staining buffer (1% FBS in PBS) for 30 min at room temperature in the dark, washed twice with PBS and resuspended in 1% paraformaldehyde containing staining buffer. Flow cytometry acquisition was performed on a BD FACSymphony A5 and data were examined using FACSDiva software (v.9.3.1, BD Bioscience). Data were further analysed using FlowJo (v.10.8.1, BD).

### Cloning

To generate a 5×C-stretch in the N-terminally Flag-tagged *FLuc* sequence^3^, a PCR strategy was used to introduce synonymous mutations of proline 197 and 198 (Supplementary Table 1). After confirmation of the mutation by Sanger sequencing, WT-5×C FLuc was back cloned into the original (WT-5×U FLuc) plasmid with BsrGI (NEB, R3575) and Bsu36I (NEB, R0524). Single-nucleotide insertion next to the C-rich sequence was accomplished by inserting a mutated DNA fragment (IDT) using BsrGI and Bsu36I. The sequence for the C=U NanoLuc reporter was purchased as a DNA fragment (IDT) and cloned using BbsI-HF (NEB, R3539) and BsmI (NEB, R0134).

### In vitro transcription and polyadenylation

Linearized and purified plasmid DNA (purchased from Genscript; see Supplementary Table 1 for sequences) was IVT with T7 polymerase (NEB, M0251L) or G47A+884G mutant T7 (gift from Y.-X. Wang’s Laboratory) in combination with inorganic pyrophosphatase (*Escherichia coli*, NEB, M0361L). Co-transcriptional capping was achieved with CleanCap AG (TriLink, N-7113) according to the manufacturer’s instructions (TriLink). SINAPs reporter mRNAs were post-transcriptionally polyadenylated with *E. coli* poly(A)polymerase (NEB, M0276) supplemented with murine RNase inhibitor (NEB, M0314) for 30 min at 37 °C according to the manufacturer’s instructions. For modified transcripts, ac^4^CTP (Jena Bioscience, NU-988L) or m^5^CTP (TriLink, N-1014-5) replaced CTP, and m^1^ΨTP (TriLink, N-1081) or ΨTP (TriLink, N-1019-5) replaced UTP in the reaction mix. Purification of polyadenylated RNA was achieved by magnetic separation using RNAClean XP beads (Beckman Coulter, A63987) according to the manufacturer’s instructions. Denaturing agarose gel electrophoresis was performed using NorthernMax 10× Denaturing gel buffer (Thermo Fisher Scientific, AM8676). Gels were stained after electrophoresis using SYBR Green II RNA gel stain (1:10,000 in TBE, Thermo Fisher Scientific, S7564) for 30 min in the dark to visualize RNA. ac^4^C-modified IVT mRNAs were diluted before gel loading to account for increased staining intensity.

### Nucleoside mass spectrometry

Modified and unmodified IVT mRNAs (100 ng each) were digested in 35 µl using snake venom phosphodiesterase (0.2 U, Abnova, P5263), calf intestinal phosphatase (2 U, Promega, M1821) and benzonase (2 U, Millipore Sigma, E1014) prepared with 1 mM MgCl_2_ (Quality Biological, 351-033), 5 mM Tris (pH 8, Invitrogen, AM9855G), 10 nmol butylated hydroxytoluene (Sigma-Aldrich, B1378) and 5 µg tetrahydrouridine (Calbiochem, 584222) (modified from a previously described method^53^) and incubated for 2 h at 37 °C. Samples were diluted with 15 µl LC–MS buffer A (0.0075% formic acid (Supelco, 5330020050) in ultrapure water) and filtered for 35 min at 3,273*g* and 4 °C through 0.2 µm Supor AcroPrep Advance 96-well plates (Cytiva, 50-206-3147). Of the filtrate, 39 µl was subjected to mass spectrometry analysis on an Agilent Technologies triple quad 6495C mass spectrometer. For HPLC (Agilent 1290 Infinity II), buffer A (described above) and buffer B (0.0075% formic acid in acetonitrile (Honeywell, LC015) were used in combination with an alkyl reversed-phase column (Zorbax RRHD StableBond Aq, 2.1 × 150 mm, 1.8 µm, 80 Å, Agilent Technologies, 859700-914); concentration buffer B: 0–1 min 0%, 1–1.4 min 0.1%, 1.4–2.8 min 0.4%, 2.8–4.2 min 0.9%, 4.2–5.6 min 1.6%, 5.6–8 min 4%, 8–11.5 min 15%, 15–15.5 min 50%, 15.5–16.5 min 50%, 16.5–17 min 0% and 17–18 min 0%.

### In vitro translation

In vitro translation assays were performed by incubating 50 ng unmodified or modified luciferase mRNA for 3 min at 65 °C before adding 3.5 μl RRL (Promega, L4960) in a final volume of 5 μl at 30 °C. Reactions were stopped at 20, 40, 60, 80, 180 and 360 min by placing on dry ice. In vitro translation assays incorporating [^35^S]-L-methionine/cysteine were performed by incubating 100 ng unmodified or modified luciferase mRNA for 3 min at 65 °C before adding 3.5 μl RRL containing an amino acid mixture (minus methionine) and 10 μCi [^35^S]-L-methionine/cysteine (Revvity, NEG772002MC) in a final volume of 5 μl at 30 °C according to the manufacturer’s instructions. Reactions were stopped at 80 min by adding 2× Laemmli loading buffer. Proteins were denatured for 10 min at 70 °C before separation on 14% SDS–polyacrylamide gels for PAGE. Radioactive signals were captured on a phosphoscreen for 72 h before detection with a Typhoon molecular bioimager (GE).

### IVT mRNA transfections

For NanoLuc, NanoLuc synthesized with G47A+884G mutant T7 polymerase, C=U NanoLuc and CD25 experiments in HeLa cells, cells were transfected in suspension with 2.5 µg IVT mRNA per 1 × 10^6^ cells using 3.75 µl MessengerMax reagent (Invitrogen, LMRNA015) in OptiMEM (Thermo Fisher Scientific, 31985062) according to the manufacturer’s instructions. After 5 min (standard condition) or 24 h (extended transfection) of exposure to the mRNA–LNP complexes, the cells were washed twice with PBS, resuspended in fresh DMEM–BCS and seeded separately for protein, RNA and luminescence experiments. At the indicated time points, cells were lysed directly in TRIzol reagent (100 µl, Thermo Fisher Scientific, 15596026) or scraped into PBS before lysis in supplemented RIPA buffer (protease and phosphatase inhibitor). For FLuc experiments, plated HeLa cells were transfected at about 50% confluency with around 2.5 µg IVT mRNA per 6-well plate with 3.75 µl MessengerMax reagent in OptiMEM, as described above. Cells for RNA analysis were lysed immediately after transfection in TRIzol reagent. At 6 h after transfection, cells for protein analysis were washed twice with PBS, scraped into PBS and lysed in cell culture lysis buffer (Promega, E1500) supplemented with protease and phosphatase inhibitor. To generate dsRNA-treated controls, HeLa cells were transfected with 0.5 µg ml^–1^ polyI:C (Millipore Sigma, P1530) for 2 h, using 12 µl MessengerMax reagent and 4 ml OptiMEM, according to the manufacturer’s instructions. Cells were collected for protein and RNA analyses 6 h after polyI:C treatment. THP-1-derived M0 macrophages, primary human MoDCs and MEFs were transfected with 1 µg IVT mRNA per 1 × 10^6^ live cells in suspension before plating (MoDCs and MEFs) or in seeded adherent cells (THP-1). For U-2 OS experiments, 500,000 cells were seeded 24 h before transfection of 1 μg RNA using 1.5 μl Lipofectamine MessengerMax according to the manufacturer’s protocol. Anisomycin (Sigma Aldrich, A9789-5MG) control cells were treated at 0.2 μg ml^–1^. The cells were collected with supplemented RIPA buffer (protease and phosphatase inhibitor) after 1.5 h. For titration experiments, RNA and MessengerMax reagent were each serial-diluted 1:10 before forming LNP complexes. Conditions were otherwise as described for HeLa cells.

### Electroporation

HeLa cells (1.1 × 10^6^) were resuspended in 100 µl Neon NxT resuspension buffer R, and aqueous RNA (1.1 μg in 10 μl) was added and gently mixed. The mixture (100 μl) was aspirated into a 100 μl Neon NxT Tip on a respective pipette and docked into a Neon NxT Pipette Station with the tube containing 2 ml buffer E100. Electroporation was performed using the HEK293 mRNA1 protocol, and the cells were immediately transferred to complete medium for plating.

### Luminescence

For in vitro translation assays, reactions were diluted with 95 μl of 1 mg ml^–1^ BSA, then 10 μl of the dilution was mixed with 40 μl PBS and 50 μl Nano-Glo reagent (Promega, N1120) according to the manufacturer’s instructions. In *NanoLuc*-transfected cells, 100 µl Nano-Glo assay reagent was used per 10,000 cells. For detection of FLuc luminescence, cleared lysate was diluted to correspond to 10,000 transfected cells in 10 µl Cell Culture lysis buffer (Promega, E1500). Luminescence was determined by adding 100 µl Luciferase assay reagent according to the manufacturer’s instructions, followed by detection using a SpectraMax iD3 (Molecular Devices) instrument.

### Protein stability

To inhibit the proteasome, HeLa cells transfected with IVT mRNA were cultured with 25 µM MG132 (Cell Signaling Technologies, 2194S) in DMEM–BCS for 6 h, followed by protein lysis. To assess protein stability, 100 µg ml^–1^ cycloheximide (Sigma, C7698) was added to HeLa cells 12 h after transfection. Cells were collected for protein analyses at the indicated time points.

### PKR inhibition

After 5–6 days of differentiation, MoDCs were counted and pretreated with the PKR inhibitor C16 (1.5 μM, 0.0075% DMSO, Sigma, 527450) for 10 min. The cells were washed and transfected with 1 μg RNA per 1 × 10^6^ cells as described above and treated for 6 h before collection.

### Western blot analysis

Cell lysates were cleared by centrifugation at full speed for 15 min at 4 °C, and the protein concentration was quantified using a Pierce BCA Protein Assay kit (Thermo Fisher Scientific, A55865). For general expression analysis, equal amounts of protein (5–30 μg) were loaded on 4–12% Bis-Tris NuPAGE gels (Thermo Fisher Scientific, NP0321), separated using NuPAGE MOPS SDS running buffer (Thermo Fisher Scientific, NP0001) and transferred onto 0.2 μm nitrocellulose membranes from a Trans-Blot Turbo RTA Mini kit (Bio-Rad, 1704270) according to the manufacturer’s instructions. For CHX-chase, an equal volume of lysate (10 μl) was analysed.

Membranes were blocked with 5% milk in 0.05% Tween-20 TBS buffer and incubated in a solution containing 5% milk in 0.05% Tween-20 TBS buffer and the following primary antibodies: rabbit anti-eIF2α (1:1,000, Cell Signaling Technology, 9722), rabbit anti-p-eIF2α ((E90), 1:1,000, Abcam, ab32157), mouse anti-NanoLuc ((965808),m1:500, R&D, MAB10026), rabbit anti-TNF ((EPR22598-212), 1:1,000, Abcam, ab255275), mouse anti-β-tubulin ((D3U1W), 1:1,000, Cell Signaling Technology, 86298), rabbit anti-CD25 ((SP176), 1:300, Abcam, ab231441), mouse anti-Flag ((M2), 1:1,000, Sigma-Aldrich, F1804), mouse anti-JNK ((1A12E1), 1:1,000, Proteintech, 66210-1-Ig), rabbit anti-phospho-SAPK/JNK ((Thr183/Tyr185) (81E11) 1:1,000, Cell Signaling Technology, 4668S), rabbit anti-phospho-p38 MAPK (Thr180/Tyr182) (1:1,000, Cell Signaling Technology, 9211S), rabbit anti-p38 MAPK (1:1,000, Cell Signaling Technology, 9212S), rabbit anti-TLR8 (1:1,000, Thermo Fisher Scientific, PA5-102413), rabbit anti-TLR7 ((EPR2088(2)), 1:1,000, Abcam, ab124928), rabbit anti-lamin B1 ((EPR8985(B)), 1:1,000, Abcam, ab133741), rabbit anti-ZNF598 ((5H5L17), 1:1,000, Thermo Fisher Scientific, 703601), mouse anti-PKR ((1441CT628.33.40), 1:1,000, Thermo Fisher Scientific, M5-37667), rabbit anti-phospho-PKR/EIF2AK2-T446 ((ARC0293), 1:1,000, ABclonal, AP1134) and mouse anti-GAPDH ((6C5), 1:1,000, Santa Cruz Biotechnology, sc-32233). After overnight incubation at 4 °C, membranes were washed 3 times in 0.05% Tween-20 TBS buffer, followed by incubation with the horseradish-peroxidase-conjugated secondary antibodies anti-mouse IgG (1:5,000, GE Healthcare, NA931) or anti-rabbit IgG (1:10,000, Cell Signaling Technology, 7074). Western blots were visualized by enhanced chemiluminescence using ECL Western Blotting Detection Reagents (Cytiva, RPN2209), SuperSignal West Pico PLUS Chemiluminescent Substrate (Thermo Fisher Scientific, 34580) or SuperSignal West Femto Maximum Sensitivity Substrate (Thermo Fisher Scientific, 34096). Chemiluminescence was detected using a ChemiDoc Imaging System (Bio-Rad). Densitometry was performed using ImageLab software (Bio-Rad).

### RNase T2 treatment

RNA (500 ng) was diluted with water. Human RNase T2 protein (sf9, His, MedChemExpress, HY-P76006) was added at the indicated concentrations in sodium acetate buffer (final 50 mM, pH 4.5) and EDTA (final 2 mM, pH 8.0). After incubation at 37 °C for 5 min, reactions were quenched with denaturing gel buffer, heated to 65 °C for 5 min and separated on a denaturing gel.

### Translation imaging

#### Microscopy

Fixed-cell data were acquired on a wide-field upright Nikon Eclipse Ni microscope, which was controlled by Nikon Elements software. The system was equipped with a Spectra X LED light engine (Lumencor), an Orca 4.0 v.2 sCMOS camera (Hamamatsu) and a ×60 oil-immersion objective lens (1.4 NA, Nikon). The *x-y* pixel size was 108.3 nm.

Live-cell data were acquired using a custom inverted Nikon Eclipse Ti-2E microscope, which was controlled by Nikon Elements software. The setup included an iLas2 Ring TIRF system with a ×60 apochromatic oil-immersion TIRF objective lens (1.49 NA, Nikon MRD01691), an ORCA-Fusion BT sCMOS camera (Hamamatsu) with a 6.5 µm pixel size, an LUNF-XL multilaser unit with 405 nm, 488 nm, 561 nm and 640 nm lasers (50 mW, 60 mW, 50 mW and 40 mW, respectively), with a TRF89901-EMV2 ET quad-band filter set (Chroma) optimized for 405, 488, 561 and 640 nm wavelengths for TIRF application. The *x-y* pixel size was 108.3 nm.

#### Fixed-cell smFISH–IF experiments

smFISH–IF was performed following previously described protocols^32,36^. In brief, 12 mm no. 1 German glass coverslips (Electron Microscopy Sciences, 72290-03) were carefully distributed into the wells of a 24-well tissue culture plate (Falcon, 353226) and cleaned with 3 M sodium hydroxide for 5 min followed by 3 washes in Dulbecco’s PBS (DPBS) (Corning, 21-031-CM). The coverslips were coated with a 1:400 dilution of fibronectin (Sigma-Aldrich, F1141-2 mg) in DPBS for 30 min at 37 °C, followed by one wash with DMEM. Next, 25,000 U-2 OS cells stably expressing the SINAPs accessory proteins *Os*TIR2–IRES–scFV–sfGFP were seeded on each coverslip. One day after seeding, the medium was exchanged and supplemented with 1 µM 5-phenyl-indole-3-acetic acid (Fisher Scientific, NC195789). IVT mRNA (10 ng) was transfected into the cells using 1 µl Lipofectamine MessengerMAX transfection reagent (Invitrogen, LMRNA003) following the manufacturer’s protocol. Cells were incubated with the transfection reagent for 10 min, washed 3 times with prewarmed DMEM–10% FBS and left to incubate for an additional 1 h in DMEM–FBS. The cells were washed 3 times with PBS supplemented with 5 mM magnesium chloride (PBSM, Millipore, M2670), then subsequently fixed at room temperature for 10 min in 4% paraformaldehyde (Electron Microscopy Sciences, 50-980-492) diluted in PBSM. Three 5-min washes were performed with 1× PBSM to remove the fixation buffer. Cells were permeabilized in a buffer (PBSM + 5 mg ml^–1^ BSA (VWR, 0332) + 0.1% Triton-X100 (Millipore, T8787)) at room temperature for 10 min, followed by another three 5-min washes with 1× PBSM. The cells were treated with a pre-hybridization buffer consisting of 2× SSC (Corning, 46-020-CM), 10% formamide (Millipore Sigma, F9037) and 5 mg ml^–1^ BSA for 30 min. During the pre-hybridization step, the final hybridization solution was prepared: 60 nM SunTag_v4-Cy5 smFISH probes, 60 nM MBS_v5-Cy3 smFISH probes^36^, chicken anti-GFP antibody (1:1,000, Aves Labs, GFP-1010), 100 units ml^–1^ SUPERase·In (Thermo Fisher Scientific, AM2694), 1 mg ml^–1^ competitor *E. coli* tRNA (Millipore Sigma, 10109541001), 2 mM ribonucleoside vanadyl complex (NEB, S1402S), 10% formamide, 2× SSC and 10% w/v dextran sulfate (Millipore Sigma, D8906). The coverslips were incubated for a total of 3 h in the hybridization solution at 37 °C. After hybridization, the coverslips were washed 4 times with a solution of 10% formamide in 2× SSC. The coverslips were stained with a secondary antibody (Alexa-488-goat anti-chicken IgY secondary antibody, Thermo Fisher Scientific, A-11039) in a buffer (10% formamide, 2× SSC) twice at 37 °C for 20 min. The cells were quickly washed 3 times with 2× SSC to remove any unbound secondary antibodies before a final 5-min wash in 2× SSC. The coverslips were carefully removed from the culture dish and mounted with ProLong Diamond antifade reagent containing DAPI (Invitrogen, P36962) on a pre-cleaned frosted glass slide (Thermo Fisher Scientific, 12-552-3). The coverslips were left in the dark for more than 24 h and sealed with clear nail polish. Fixed-cell imaging was performed on the day following each respective smFISH–IF experiment.

#### smFISH–IF time-course experiments with balancer mRNAs

Cells were transfected with a mixture of 10 ng SINAPs reporter mRNA and 490 ng *CD25* balancer mRNA with the same nucleotide modification using 1 µl Lipofectamine MessengerMAX transfection reagent (Invitrogen, LMRNA003) following the manufacturer’s instructions. All experimental wells were transfected at the same time with the same master mix of reagents. Cells were incubated with the transfection mixture for 10 min, after which the transfection medium was removed and cells were washed three times with pre-warmed, equilibrated culture medium, at which point the time was marked as zero (*t* = 0). At 10, 20, 30, 60, 120 and 240 min after the wash, different wells were fixed and subject to the smFISH–IF procedure as described above.

#### Ribosome runoff experiments

For live-cell imaging experiments, U-2 OS cells stably expressing *Os*TIR1–IRES–scFV–sfGFP and tdMCP–mScarlet–tagRFPT–CAAX were seeded onto 35 mm glass-bottom dishes (Cellvis, D35-20-1.5-N) 48 h before transfection (around 80,000 cells per dish). The medium was refreshed 24 h before transfection, and 500 µM 3-indoleacetic acid (Millipore Sigma, I2886) was added to degrade mature proteins and to minimize background fluorescence.

On the day of the experiment, each dish was transfected with 100 ng IVT mRNA diluted in 125 µl Opti-MEM mixed with 5 µl Lipofectamine MessengerMax transfection reagent (Invitrogen, LMRNA003) diluted in 120 µl Opti-MEM following the manufacturer’s protocol. Cells were incubated with the transfection reagent for 1 h, washed 3 times with prewarmed DMEM–FBS and then rinsed with FluoroBrite DMEM (Gibco, A1896701) supplemented with 10% FBS. Following 1 h of incubation in imaging medium, dishes were transferred to the microscope stage for live-cell imaging. Cells were maintained at 37 °C and 5% CO_2_ during imaging inside a Toki hit temperature-controlled stage top incubator. Positively transfected cells actively undergoing translation were identified by detecting translation signals in the GFP channel as well as positive RFP signal in the RNA channel, with each cell displaying approximately 20–40 tethered mRNAs.

Harringtonine treatment and ribosome runoff experiments were performed according to a previously established protocol^32^. In brief, for each experiment, 3–4 positively transfected cells with active TLSs and close to each other were selected for imaging. After saving the cell positions and microscope focus, 0.75 ml imaging medium was removed from the dish and mixed with harringtonine in a 1.5 ml centrifuge tube, then added back to the dish, gently and thoroughly mixed to a final concentration of 9 µg ml^–1^ with the original medium in the dish by pipetting. Imaging started 1 min after adding harringtonine and the selected cells were imaged every 10 s for 30 min, with sequential laser excitation at 488 nm and 561 nm. Both channels were captured with a 500 ms camera exposure time.

#### smFISH–IF experiments with harringtonine ribosome runoffs

Cells were transfected with 10 ng SINAPs reporter mRNA using 1 µl Lipofectamine MessengerMAX transfection reagent (Invitrogen, LMRNA003), following the manufacturer’s instructions. All experimental wells were transfected at the same time with the same master mix of reagents. Cells were incubated with the transfection mixture for 10 min, after which the transfection medium was removed, and cells were washed 3 times with prewarmed, equilibrated culture medium, at which point the time was marked as zero (*t* = 0). Harringtonine treatment was performed as described above, with the final concentration of harringtonine as 9 µg ml^–1^. At 0, 3, 5 and 15 min after harringtonine addition, the cells were washed 3 times with PBS supplemented with 5 mM magnesium chloride (PBSM, Millipore, M2670), then subsequently fixed at room temperature for 10 min in 4% paraformaldehyde (Electron Microscopy Sciences, 50-980-492) diluted in PBSM. The subsequent smFISH–IF procedure was performed as described above.

#### Ribosome runoff experiments after knocking down *ZNF598*

At 48 h before imaging, 5 pmol DsiRNA (Integrated DNA Technologies; ZNF598: hs.Ri.ZNF598.13.1-3; or control siRNA: 51-01-14-03) was diluted in 50 µl Opti-MEM mixed with 1.5 µl Lipofectamine RNAiMAX transfection reagent (Invitrogen, 13778). While the transfection mixture was incubated at room temperature for 10 min, U-2 OS cells stably expressing *Os*TIR1–IRES–scFV–sfGFP and tdMCP–mScarlet–tagRFPT–CAAX were seeded onto 4-chamber 35 mm glass-bottom dishes (Cellvis, D35C4-20-1.5-N) (30,000 cells per well). The transfection mixture was added to the dish immediately after cell seeding. Ribosome runoff was performed as described above with the following modifications: 20 ng mRNA was transfected per well, and a final concentration of 9 µg ml^–1^ harringtonine was added per well to initiate the runoff.

### Image analysis and quantification

#### Ribosome runoff

Single-molecule imaging analysis with a Matlab pipeline built around U-Track has been previously described^36,54^. The first step involved the detection of mRNAs and TLSs in each frame using the AirLocalize algorithm^55,56^. The second step used the U-Track software for tracking individual mRNAs and TLSs separately. The third step used a custom-built colocalization algorithm to link the detected mRNA and TLS tracks. After automatic detection, all tracks were manually inspected and verified using a custom-built TrackViewer in Matlab. To calculate ribosome off-time, we only analysed tracks with translating mRNAs at the beginning (the first five frames when harringtonine was added), and when the RNA signal persisted after the disappearance of the translation signal (for 3 frames). This approach ensured that we did not count mRNAs leaving the imaging field due to untethering from the membrane. The ribosome runoff time was defined as the time point when the TLS intensity dropped below 10% of the one at *t* = 0. We calculated the Kaplan–Meier survival probability with all runoff times (Fig. 4d).

#### smFISH–IF

Fixed-cell smFISH–IF experiments were analysed using a custom-built Matlab pipeline as previously described^32^. To detect RNA, we first manually segmented cell boundaries of successfully transfected cells and their DAPI-stained nuclei with FISH-Quant^57^. After filtering, an intensity threshold was used to identify potential fluorescent spots in the image. The locations and the fluorescent intensities of the candidate spots were fitted using a 3D Gaussian function. The *SunTag* and *MS2* FISH probes were labelled with two different colours and detected separately. To correct chromatic aberration, we prepared 100 nm TetraSpek multicolour beads (Invitrogen, T7279) on a coverslip and imaged at the same channels as our experimental samples^58^. *SunTag* and *MS2* spots were colocalized with a nearest-neighbour analysis with a 3-pixel threshold after chromatic correction. We focused on intact mRNAs that contained both *SunTag* and *MS2* FISH signals. To calculate the TLS intensity, we fit a 15 × 15-pixel region around the *SunTag* FISH spot in the IF channel to a 3D Gaussian function. To measure the intensity of a single mature protein, a similar single-particle detection method was performed in the IF channel excluding the 15 × 15-pixel RNA-containing region. By normalizing the total integrated intensity of the TLS by the median single-peptide intensity in the same cell, we estimated the number of ribosomes actively translating on the mRNA.

#### ZNF598–HaloTag colocalization detection and track analysis

To quantify interactions between ZNF598–HaloTag and TLSs, we developed a custom colocalization detection script in Matlab (R2021a) tailored for weak live-cell single-molecule fluorescence intensity time series. For each trace, the fluorescence intensity of ZNF598 was first smoothed using a 3-frame moving average filter to reduce frame-to-frame noise. Positive events were defined as contiguous time windows when the smoothed signal exceeded a threshold for at least 5 frames, corresponding to 10 s at a 2-s frame rate. The threshold was user defined (typically 1.5 times the background intensity and may be adjusted to slightly higher for traces with low signal variability). To tolerate brief signal disappearance (for example, due to molecules moving out of the evanescent field), we incorporated a merging criterion. Events separated by fewer than 3 frames were merged if the gap region exceeded 75% of the threshold or the pre-gap and post-gap intensities were similar (normalized difference less than 20%). The colocalization detection events were saved in a logical mask variable for each trace. These variables were subsequently used to compute interaction durations, ZNF598–HaloTag intensities and the fraction of colocalization. The detection was visually validated on more than 50 randomly selected traces per condition to ensure accuracy and robustness across replicate datasets. We confirmed correct colocalization start and end points and verified that the script appropriately merged or excluded brief subthreshold gaps.

### Mouse studies

DLin-MC3-DMA was purchased from MedKoo Biosciences. 18PG and DMG-PEG-2000 were obtained from Avanti Polar Lipids. Cholesterol was from Sigma-Aldrich.

#### LNP synthesis and characterization

Parameters for LNP synthesis were as previously described^24–26^. In brief, to prepare the organic phase, a mixture of DLin-MC3 DMA, cholesterol, DMG-PEG2000 and 18PG was dissolved in ethanol. To prepare the aqueous phase, corresponding mRNA was prepared in magnesium acetate buffer (25 mM, pH 4.0, Fisher Scientific, SB85-1). All mRNA samples were stored at −80 °C and thawed on ice before use. For LNP synthesis, the aqueous and ethanol phases prepared were mixed at a 3:1 ratio in a flash nanocomplexation device using syringe pumps, purified by dialysis against deionized water using a 100-kDa molecular weight cutoff cassette (Thermo Fisher Scientific) at 4 °C for 24 h and stored at 4 °C before injection. The size, polydispersity index and zeta potentials of LNPs were measured using dynamic light scattering (ZetaPALS, Brookhaven Instruments). Diameters are reported as the intensity mean average.

#### Characterization of the encapsulation efficiency of mRNA LNPs

The encapsulation efficiency of mRNA in LNPs was evaluated using a Quant-iT RiboGreen assay (Thermo Fisher Scientific, R11490). To disrupt the LNP structure and release the encapsulated mRNA, LNP samples were treated with 0.5% w/v Triton X-100 (Sigma-Aldrich, T8787). Both Triton-treated and untreated LNP samples were diluted to a concentration of less than 1 μg mRNA per ml before being mixed with an equal volume of RiboGreen working solution (200-fold dilution). Standard curves were prepared using free mRNA solutions with or without 0.5% w/v Triton X-100, covering a concentration range of 0.1–1.0 μg mRNA per ml. Fluorescence measurements (excitation of 480 nm, emission of 520 nm) were recorded, and the concentrations of free mRNA (untreated samples) and total mRNA (Triton-treated samples) in the LNP formulations were quantified by comparing the fluorescence intensities against the corresponding standard curves^59^.

#### Animals

All animal procedures were performed with ethical compliance and approval by the Johns Hopkins Institutional Animal Care and Use Committee (protocol no. MO23E31). Female BALB/c mice (6–8 weeks) were obtained from the Jackson Laboratory and randomly grouped. Mice were generally fed a diet containing low fibre (5%), protein (20%) and fat (5–10%). The pelleted feed was supplied. Mice were supplied feed free choice and they ate 4–5 g a day (12 g per 100 g body weight per day). Water was supplied free choice and they usually drank 3–5 ml a day (1.5 ml per 10 g body weight per day). Water was supplied using automatic waterers. Mouse rooms were maintained at 30–70% relative humidity and a temperature of 18–26 °C (64–79 °F) with at least 10 room air changes per hour. The mice were housed in standard shoebox cages with filter tops under standard specific pathogen-free conditions with a 12-h light–dark cycle. Mice were provided with corncob as bedding. Sample sizes were selected based on our previous published work using similar mRNA–LNPs in vivo immune profiling and biodistribution studies^25^, for which comparable group sizes were sufficient to detect biologically meaningful differences. The in vivo IVIS experiments were independently replicated twice, and the datasets were pooled for analyses. Mice were randomly allocated into experimental groups. In vivo experiments and data collection were performed blinded to group allocation. Investigators remained blinded during data analysis whenever feasible.

The LNPs were intravenously injected into mice via the lateral tail vein at a predetermined dose per mouse. The mice were intraperitoneally injected with 100 μl of 30 mg ml^–1^ Nano-Glo Fluorofurimazine In Vivo substrate (FFz, Promega, N4100) solution and were anaesthetized in a ventilated anaesthesia chamber with 1.5% isoflurane in oxygen and imaged at 5 min after the injection with an in vivo imaging system (IVIS, Perkin-Elmer). Luminescence was quantified using Living Image software (Perkin-Elmer).

### Sucrose density centrifugation

At 1.5 h after transfection, HeLa cells transfected with IVT mRNA were washed with ice-cold PBS and scraped into lysis buffer (50 mM HEPES pH 7.4, 100 mM potassium acetate, 15 mM magnesium acetate, 1 mM DTT, 1% Triton X-100 and emetine (360 µM, Sigma, 324693-250MG)) on ice. Lysates were incubated at 4 °C for 10 min before clearing for 10 min at 4 °C and 10,000*g*. RNA concentration was determined using a Qubit 4 Fluorometer (Thermo Fisher Scientific) and about 60 µg was diluted to a total volume of 600 µl with lysis buffer before layering on top of 10–45% sucrose gradients (25 mM HEPES pH 7.4, 100 mM potassium acetate, 5 mM magnesium acetate and 1 mM DTT) prepared with a Biocomp Gradient Master. Centrifugation to separate ribosome fractions was performed for 1 h and 45 min at 41,000 rpm (SW41Ti, Optima XPN-80, Beckman Coulter) at 4 °C. Gradients were fractionated, and UV (A260) absorbance across the gradients was measured using a top-down Biocomp Piston Gradient Fractionator with a Triax flow cell per the manufacturer’s instructions. To each fraction, 2 volumes of 100% ice-cold ethanol was added and the RNA was precipitated at −80 °C. RNA was isolated by pelleting for 30 min at 18,500*g* and 4 °C, followed by resuspension in LET buffer (25 mM Tris pH 8.0, 100 mM LiCL and 20 mM EDTA), addition of 1% SDS and double acid phenol–chloroform–LET extraction. Supernatants containing RNA were isolated after ammonium acetate–ethanol precipitation containing 1 µl GlycoBlue Coprecipitant (Thermo Fisher Scientific, AM9516) per sample.

### RNA isolation and RT–qPCR

Unless described otherwise, total RNA was prepared by lysing cells at the indicated time points in TRIzol (100 µl reagent per 1–2 × 10^5^ cells) or adding an equal volume to in vitro lysates and extracted according to the manufacturer’s instructions. RNA was reverse transcribed with oligo(dT) primers or random hexamers using a Superscript IV system (Thermo Fisher Scientific, 18090200) according to the manufacturer’s suggestions, followed by qPCR with specific primers (Supplementary Table 1) using LightCycler 480 SYBR Green I master mix (Roche, 04887352001) in a LightCycler 96 Instrument (Roche).

### RNA-seq library preparation and analysis

In brief, 1 µg modified and unmodified mRNA of the +1 FS FLuc reporter was used to generate cDNA libraries with a NEBNext Ultra II Directional RNA Library Prep kit for Illumina (NEB, E7760) starting from the fragmentation step. NEBNext Multiplex Oligos for Illumina (96 Unique Dual Index Primer Pairs, NEB, E6440) were used with 6 cycles of PCR amplification. The concentrations of the indexed libraries were analysed on an Agilent 4200 TapeStation (Agilent Technologies) using a D1000 kit (Agilent Technologies). Equimolar amounts of the indexed libraries were pooled to obtain a 2 nM library mixture. After further dilution, the final 750 pM library was sequenced single-end with dual index (122 × 8 × 8) using Illumina NextSeq2000 P1 reagents (100 cycles) on an Illumina NextSeq2000 instrument following the manufacturer’s instructions (Illumina).

### RNA-seq analysis

The quality of raw reads was assessed using FastQC (v.0.12.1)^60^. The average per-base quality score across all the files was greater than 32, and contamination of Illumina adapters was observed at the 3′ end of the reads. These adapters were removed using Trim Galore (v.0.6.11)^61^. As the sequenced RNA was from a construct with a known open reading frame, the libraries (both raw and trimmed) were aligned using bowtie2 (v.2.5.3)^62,63^. The average alignment rate of the raw reads was 94% and increased to 99.52% for the trimmed reads. The aligned reads were sorted, indexed and summary statistics (flagstat) were obtained using samtools (v.1.23)^64^. The mapped sequences with MAPQ > = 5 were used to calculate the insertion and deletion rate in terms of per nucleotide using Qualimap (v.2.2.1)^65^ across the open reading frame. The libraries were visualized using IGV^66,67^.

### Ribo-seq sample and library preparation

Hela cells were grown to 70–80% confluency before co-transfection with 3 µg mRNA for the WT 5×U FLuc reporter per 10 cm dish for each modification (unmodified, ac^4^C and m^1^Ψ) using MessengerMax reagent (22.5 μl per dish). Three 10 cm^2^ plates were transfected for each modification. After 3 h, the cells were washed with PBS and the samples were collected on wet ice in 500 µl of lysis buffer (see the section ‘Sucrose density centrifugation’ with inclusion of complete mini protease inhibitor cocktail, Roche, 11 836 153 001). Cell lysates were next triturated 10 times and clarified by centrifugation at 20,000*g* for 10 min at 4 °C.

Ribosome profiling and RPF purification was performed according to a published protocol^68^ but with modifications. In brief, for ribosome profiling, micrococcal nuclease I (MNase I, 2,400 U) was used to digest clear lysate equivalent to an optical density of 3.5 at room temperature for 40 min. MNase I was used in place of the more commonly used RNase I because both ac^4^C and m^1^Ψ confer resistance to RNase I cleavage^69,70^, which would have introduced systematic bias against modified transcripts and precluded direct comparison of ribosome footprint densities across modification states. Reactions were stopped using 10 mM EGTA and 5 μl SUPERaseIn. Digested lysates of unmodified, ac^4^C-modificed and m^1^Ψ-modified samples were loaded onto 10–45% sucrose gradients as described above for 1 h and 40 min. The monosome fraction was collected and RNA was isolated from ribosome-protected fragments using the phenol–chloroform–LET method (described in the section ‘Sucrose density centrifugation’). Next, 25–35 bp size RNA was purified from 15% urea–PAGE using gel extraction buffer (300 mM sodium acetate (pH 5.5), 1 mM EDTA and 0.25% (w/v) SDS). Library preparation was performed using a QiaSeq miRNA Library kit (Qiagen, 331502) and sequenced on a miSeq Illumina platform.

### Ribo-seq analysis

Raw sequencing reads were processed to remove adapter sequences and to extract unique molecular identifiers (UMIs) using UMI-tools (v.1.1.5)^71^ with the following settings: --extract method=regex –bc pattern=‘.+(?P<discard_1>AACTGTAGGCACCATCAAT){s<=2} (?P<umi_1>.{12})(?P<discard_2>.+)’. UMI-extracted reads were mapped to the *FLuc* sequence using Bowtie (v.1.3.1)^72^ with the following settings: -a -v 2 –norc. Extracted UMIs and mapping coordinates were used to remove duplicate reads using the dedup function from UMI-tools with the following options: --method unique --extract-umi-method=read_id --umi-separator=“_“.

RPFs exhibiting clear periodicity were identified using the length_filter function from the riboWaltz (v.2.0) package^47^, with parameters set to length_filter_mode = “periodicity” and periodicity_threshold = 50. P-site offsets were subsequently estimated using the psite function, with the following settings: flanking = 6, cl = 99, and extremity = “auto”. The computed offsets were then applied to assign P-site positions to individual reads using the psite_info function from riboWaltz.

### Immunoprecipitation and mass spectrometric analysis

Lysates from HeLa cells transfected with IVT WT or +1FS FLuc reporter mRNAs with or without MG132 treatment were prepared as described above. Immunoprecipitation of Flag-tagged FLuc was performed using Flag M2 beads (Sigma-Aldrich, F3165). In brief, 900 μg and 350 μg Hela cell lysate from cells transfected with 5×U FLuc and +1FS-5×U FLuc reporter mRNA in the presence of MG132, respectively, was used for immunoprecipitation. Next, 5 μg and 2 μg of Flag-M2 antibody, respectively, was used for immunoprecipitation at 4 °C for 2 h followed by the addition of Protein-G Sepharose beads for 30 min. Affinity-purified beads were washed 3 times with TBS and resuspended in 25 mM HEPES, pH 8.0, heated at 95 °C for 5 min to denature the proteins, followed by digestion overnight with trypsin (Thermo Fisher Scientific) at 37 °C. Digests were purified using the proprietary peptide clean up columns from an EasyPEP Mini MS Sample Prep kit (Thermo Scientific, A40006). Peptide mixtures were vacuum centrifuged to dryness and stored at −80 °C until analysis by mass spectrometry.

Dried peptide fractions were reconstituted in 0.1% TFA and subjected to nanoflow liquid chromatography (Thermo EASY-nLC 1200, Thermo Fisher Scientific) coupled to an Orbitrap LUMOS mass spectrometer (Thermo Fisher Scientific). Peptides were separated using a low pH gradient with 5–50% acetonitrile over 120 min in mobile phase containing 0.1% formic acid at 300 nl min^–1^ flow rate. Mass spectrometry scans were performed in the Orbitrap analyser at a resolution of 120,000 with an ion accumulation target set at 4 × 10^5^ and max IT set at 50 ms over a mass range of 385–2,000 *m/z*. Ions with determined charge states between 2 and 5 were selected for fragment ion scans. A cycle time of 3 s was used, and a quadrupole isolation window of 1.4 *m/z* was used for tandem mass spectrometry (MS/MS) analyses. An Orbitrap at 15,000 resolutions with a normalized AGC set at 100 followed by maximum injection time set at 100 ms with a normalized collision energy setting of 30 was used for MS/MS analyses.

Acquired MS/MS spectra were searched against a fasta file containing the luciferase protein sequence along with potential different slippage proteins using a SEQUEST HT in Proteome Discoverer 2.4 software (Thermo Fisher Scientific). The precursor ion tolerance was set at 10 ppm, and the fragment ion tolerance was set at 0.02 Da, along with methionine oxidation included as dynamic modification. Carbamidomethylation of cysteine residues was set as a static modification. Trypsin was specified as the proteolytic enzyme, with up to two missed cleavage sites allowed. Searches used a reverse sequence decoy strategy to control for the false peptide discovery, and identifications were validated using fixed value PSM Validator algorithms. Both highly confident and medium confident peptides were considered as potential true positives. Untransfected Hela cells were used as a negative control.

### Reporting summary

Further information on research design is available in the Nature Portfolio Reporting Summary linked to this article.

## Online content

Any methods, additional references, Nature Portfolio reporting summaries, source data, extended data, supplementary information, acknowledgements, peer review information; details of author contributions and competing interests; and statements of data and code availability are available at 10.1038/s41586-026-10729-8.

## Supplementary information

Supplementary information

Reporting Summary

Supplementary Tables Supplementary Table 1: List of IVT mRNA and primer sequences. Supplementary Table 2: Summary of IVT mRNA sequencing of the +1FS-5×U FLuc reporter, with error statistics in the open reading frame (MAPQ ≥ 5 and trimmed). Supplementary Table 3: Ribo-seq quality control summary, with total reads, duplication rates and unique mapping to the mRNAs of the WT-5×U FLuc reporter.

Supplementary Video 1 Harringtonine-induced ribosome runoff of IVT mRNAs, related to Fig. 3. Representative live-cell imaging of translating IVT mRNAs following harringtonine treatment. TLSs were visualized using the SINAPs reporter system. Fluorescence decreases as elongating ribosomes complete translation and dissociate from the mRNA. Time is indicated in minutes.

Supplementary Video 2 ZNF598–HaloTag colocalization to actively translating IVT mRNAs, related to Fig. 4. Representative live-cell imaging of ZNF598–HaloTag recruitment to actively translating IVT mRNAs visualized using the SINAPs reporter system. TLSs and ZNF598 localization were monitored simultaneously to assess colocalization with translating mRNAs. Time is indicated in seconds. Scale bars, 2 µm.

Supplementary Video 3 Harringtonine-induced ribosome runoff of IVT mRNAs in cells treated with siRNA, related to Fig. 4. Representative live-cell imaging of harringtonine-induced ribosome runoff in cells treated with siRNA targeting *ZNF598*. TLSs were visualized using the SINAPs reporter system, and fluorescence decay reflects ribosome clearance from translating IVT mRNAs. Time is indicated in minutes. Scale bars, 10 µm.

## Data Availability

RNA-seq data are available from the Gene Expression Omnibus under the identifier GSE301223. Ribo-seq data are available from the NCBI BioProject under the identifier PRJNA1280991.

## Code availability

Custom code for analysis of live-cell imaging data is available at GitHub (https://github.com/binwulab/uLocalize).

## References

[1] Rohner, E., Yang, R., Foo, K. S., Goedel, A. & Chien, K. R. Unlocking the promise of mRNA therapeutics. *Nat. Biotechnol.* **40**, 1586–1600 (2022). 36329321 10.1038/s41587-022-01491-z

[2] Andries, O. et al. N^1^-methylpseudouridine-incorporated mRNA outperforms pseudouridine-incorporated mRNA by providing enhanced protein expression and reduced immunogenicity in mammalian cell lines and mice. *J. Control. Release* **217**, 337–344 (2015). 26342664 10.1016/j.jconrel.2015.08.051

[3] Mulroney, T. E. et al. *N*^1^-methylpseudouridylation of mRNA causes +1 ribosomal frameshifting. *Nature* **625**, 189–194 (2024). 38057663 10.1038/s41586-023-06800-3 PMC10764286

[4] Monroe, J. et al. N1-Methylpseudouridine and pseudouridine modifications modulate mRNA decoding during translation. *Nat. Commun.* **15**, 8119 (2024). 39284850 10.1038/s41467-024-51301-0 PMC11405884

[5] Svitkin, Y. V., Gingras, A. C. & Sonenberg, N. Membrane-dependent relief of translation elongation arrest on pseudouridine- and *N*^1^-methyl-pseudouridine-modified mRNAs. *Nucleic Acids Res.* **50**, 7202–7215 (2022). 34933339 10.1093/nar/gkab1241 PMC9303281

[6] Verbeke, R., Hogan, M. J., Loré, K. & Pardi, N. Innate immune mechanisms of mRNA vaccines. *Immunity* **55**, 1993–2005 (2022). 36351374 10.1016/j.immuni.2022.10.014 PMC9641982

[7] Metkar, M., Pepin, C. S. & Moore, M. J. Tailor made: the art of therapeutic mRNA design. *Nat. Rev. Drug Discovery* **23**, 67–83 (2024). 38030688 10.1038/s41573-023-00827-x

[8] Karikó, K., Buckstein, M., Ni, H. & Weissman, D. Suppression of RNA recognition by Toll-like receptors: the impact of nucleoside modification and the evolutionary origin of RNA. *Immunity* **23**, 165–175 (2005). 16111635 10.1016/j.immuni.2005.06.008

[9] Rozman, B. et al. *N*^1^-Methylpseudouridine directly modulates translation dynamics. *Nature* **651**, 533–541 (2026). 41535458 10.1038/s41586-025-09945-5

[10] Cappannini, A. et al. MODOMICS: a database of RNA modifications and related information. 2023 update. *Nucleic Acids Res.* **52**, D239–D244 (2024). 38015436 10.1093/nar/gkad1083 PMC10767930

[11] Arango, D. et al. Acetylation of cytidine in mRNA promotes translation efficiency. *Cell* **175**, 1872–1886 (2018). 30449621 10.1016/j.cell.2018.10.030 PMC6295233

[12] Schiffers, S. & Oberdoerffer, S. ac4C: a fragile modification with stabilizing functions in RNA metabolism. *RNA* **30**, 583–594 (2024). 38531654 10.1261/rna.079948.124 PMC11019744

[13] Arango, D. et al. Direct epitranscriptomic regulation of mammalian translation initiation through N4-acetylcytidine. *Mol. Cell* **82**, 2797–2814 (2022). 35679869 10.1016/j.molcel.2022.05.016 PMC9361928

[14] Karijolich, J. & Yu, Y.-T. Converting nonsense codons into sense codons by targeted pseudouridylation. *Nature* **474**, 395–398 (2011). 21677757 10.1038/nature10165 PMC3381908

[15] Dousis, A., Ravichandran, K., Hobert, E. M., Moore, M. J. & Rabideau, A. E. An engineered T7 RNA polymerase that produces mRNA free of immunostimulatory byproducts. *Nat. Biotechnol.* **41**, 560–568 (2023). 36357718 10.1038/s41587-022-01525-6 PMC10110463

[16] Kirschman, J. L. et al. Characterizing exogenous mRNA delivery, trafficking, cytoplasmic release and RNA–protein correlations at the level of single cells. *Nucleic Acids Res.* **45**, e113 (2017). 28449134 10.1093/nar/gkx290 PMC5499550

[17] Majer, O., Liu, B. & Barton, G. M. Nucleic acid-sensing TLRs: trafficking and regulation. *Curr. Opin. Immunol.* **44**, 26–33 (2017). 27907816 10.1016/j.coi.2016.10.003 PMC5446938

[18] Anderson, B. R. et al. Nucleoside modifications in RNA limit activation of 2′−5′-oligoadenylate synthetase and increase resistance to cleavage by RNase L. *Nucleic Acids Res.* **39**, 9329–9338 (2011). 21813458 10.1093/nar/gkr586 PMC3241635

[19] Diez, M. et al. iCodon customizes gene expression based on the codon composition. *Sci. Rep.* **12**, 12126 (2022). 35840631 10.1038/s41598-022-15526-7 PMC9287306

[20] Sharfe, N., Dadi, H. K., Shahar, M. & Roifman, C. M. Human immune disorder arising from mutation of the alpha chain of the interleukin-2 receptor. *Proc. Natl Acad. Sci. USA* **94**, 3168–3171 (1997). 9096364 10.1073/pnas.94.7.3168 PMC20340

[21] Szaruga, M. et al. Activation of the integrated stress response by inhibitors of its kinases. *Nat. Commun.* **14**, 5535 (2023). 37684277 10.1038/s41467-023-40823-8 PMC10491595

[22] Lu, T. et al. N1-methylpseudouridine mRNA modification enhances efficiency and specificity of gene overexpression by preventing Prkra-mediated global translation repression. *Nucleic Acids Res.* **53**, gkaf963 (2025). 41099713 10.1093/nar/gkaf963 PMC12529930

[23] Berouti, M. et al. Pseudouridine RNA avoids immune detection through impaired endolysosomal processing and TLR engagement. *Cell* **188**, 4880–4895 (2025). 40580950 10.1016/j.cell.2025.05.032

[24] Zhu, Y. et al. Multi-step screening of DNA/lipid nanoparticles and co-delivery with siRNA to enhance and prolong gene expression. *Nat. Commun.* **13**, 4282 (2022). 35879315 10.1038/s41467-022-31993-y PMC9310361

[25] Zhu, Y. et al. Screening for lipid nanoparticles that modulate the immune activity of helper T cells towards enhanced antitumour activity. *Nat. Biomed. Eng.* **8**, 544–560 (2024). 38082180 10.1038/s41551-023-01131-0 PMC11162325

[26] Zhu, Y. et al. Optimization of lipid nanoparticles for gene editing of the liver via intraduodenal delivery. *Biomaterials* **308**, 122559 (2024). 38583366 10.1016/j.biomaterials.2024.122559 PMC11099935

[27] Morisaki, T. et al. Real-time quantification of single RNA translation dynamics in living cells. *Science* **352**, 1425–1429 (2016). 27313040 10.1126/science.aaf0899

[28] Pichon, X. et al. Visualization of single endogenous polysomes reveals the dynamics of translation in live human cells. *J. Cell Biol.* **214**, 769–781 (2016). 27597760 10.1083/jcb.201605024 PMC5021097

[29] Wu, B., Eliscovich, C., Yoon, Y. J. & Singer, R. H. Translation dynamics of single mRNAs in live cells and neurons. *Science* **352**, 1430–1435 (2016). 27313041 10.1126/science.aaf1084 PMC4939616

[30] Yan, X., Hoek, T. A., Vale, R. D. & Tanenbaum, M. E. Dynamics of translation of single mRNA molecules in vivo. *Cell* **165**, 976–989 (2016). 27153498 10.1016/j.cell.2016.04.034 PMC4889334

[31] Wang, C., Han, B., Zhou, R. & Zhuang, X. Real-time imaging of translation on single mRNA transcripts in live cells. *Cell* **165**, 990–1001 (2016). 27153499 10.1016/j.cell.2016.04.040 PMC4905760

[32] Latallo, M. J., Livingston, N. M. & Wu, B. Translation imaging of single mRNAs in established cell lines and primary cultured neurons. *Methods* **162-163**, 12–22 (2019). 30905747 10.1016/j.ymeth.2019.03.021

[33] Svitkin, Y. V. et al. N1-methyl-pseudouridine in mRNA enhances translation through eIF2α-dependent and independent mechanisms by increasing ribosome density. *Nucleic Acids Res.* **45**, 6023–6036 (2017). 28334758 10.1093/nar/gkx135 PMC5449617

[34] Watkins, L., Li, M. & Wu, B. Translation elongation: measurements and applications. *RNA Biol.* **22**, 1–10 (2025). 40377059 10.1080/15476286.2025.2504727 PMC12087489

[35] Goldman, D. H., Livingston, N. M., Movsik, J., Wu, B. & Green, R. Live-cell imaging reveals kinetic determinants of quality control triggered by ribosome stalling. *Mol. Cell* **81**, 1830–1840 (2021). 33581075 10.1016/j.molcel.2021.01.029 PMC8052310

[36] Livingston, N. M. et al. Bursting translation on single mRNAs in live cells. *Mol. Cell* **83**, 2276–2289 (2023). 37329884 10.1016/j.molcel.2023.05.019 PMC10330622

[37] Lyons, E. F. et al. Translation elongation as a rate-limiting step of protein production. *Cell Syst.* **17**, 101490 (2026). 41679296 10.1016/j.cels.2025.101490

[38] Brandman, O. et al. A ribosome-bound quality control complex triggers degradation of nascent peptides and signals translation stress. *Cell* **151**, 1042–1054 (2012). 23178123 10.1016/j.cell.2012.10.044 PMC3534965

[39] Juszkiewicz, S. et al. ZNF598 Is a quality control sensor of collided ribosomes. *Mol. Cell* **72**, 469–481 (2018). 30293783 10.1016/j.molcel.2018.08.037 PMC6224477

[40] De La Cruz, A. C. et al. Single-protein/RNA imaging reveals ZNF598 as a limiting factor in resolving collided ribosomes. *EMBO J.* **44**, 5215–5232 (2025). 40750700 10.1038/s44318-025-00523-z PMC12436648

[41] Filbeck, S., Cerullo, F., Pfeffer, S. & Joazeiro, C. A. P. Ribosome-associated quality-control mechanisms from bacteria to humans. *Mol. Cell* **82**, 1451–1466 (2022). 35452614 10.1016/j.molcel.2022.03.038 PMC9034055

[42] Hickey, K. L. et al. GIGYF2 and 4EHP Inhibit translation initiation of defective messenger RNAs to assist ribosome-associated quality control. *Mol. Cell* **79**, 950–962 (2020). 32726578 10.1016/j.molcel.2020.07.007 PMC7891188

[43] Garzia, A. et al. The E3 ubiquitin ligase and RNA-binding protein ZNF598 orchestrates ribosome quality control of premature polyadenylated mRNAs. *Nat. Commun.* **8**, 16056 (2017). 28685749 10.1038/ncomms16056 PMC5504347

[44] Wu, C. C., Peterson, A., Zinshteyn, B., Regot, S. & Green, R. Ribosome collisions trigger general stress responses to regulate cell fate. *Cell* **182**, 404–416 (2020). 32610081 10.1016/j.cell.2020.06.006 PMC7384957

[45] Sinha, N. K. et al. The ribotoxic stress response drives UV-mediated cell death. *Cell* **187**, 3652–3670 (2024). 38843833 10.1016/j.cell.2024.05.018 PMC11246228

[46] Simms, C. L., Yan, L. L., Qiu, J. K. & Zaher, H. S. Ribosome collisions result in +1 frameshifting in the absence of no-go decay. *Cell Rep.* **28**, 1679–1689 (2019). 31412239 10.1016/j.celrep.2019.07.046 PMC6701860

[47] Lauria, F. et al. riboWaltz: optimization of ribosome P-site positioning in ribosome profiling data. *PLoS Comput. Biol.* **14**, e1006169 (2018). 30102689 10.1371/journal.pcbi.1006169 PMC6112680

[48] O’Connor, M. Imbalance of tRNAPro isoacceptors induces +1 frameshifting at near-cognate codons. *Nucleic Acids Res.* **30**, 759–765 (2002). 11809889 10.1093/nar/30.3.759 PMC100296

[49] Hoffer, E. D. et al. Structural insights into mRNA reading frame regulation by tRNA modification and slippery codon–anticodon pairing. *eLife* **9**, e51898 (2020). 33016876 10.7554/eLife.51898 PMC7577736

[50] Moss, M. J., Chamness, L. M. & Clark, P. L. The effects of codon usage on protein structure and folding. *Annu. Rev. Biophys.* **53**, 87–108 (2024). 38134335 10.1146/annurev-biophys-030722-020555 PMC11227313

[51] Nishimura, K., Fukagawa, T., Takisawa, H., Kakimoto, T. & Kanemaki, M. An auxin-based degron system for the rapid depletion of proteins in nonplant cells. *Nat. Methods* **6**, 917–922 (2009). 19915560 10.1038/nmeth.1401

[52] Yesbolatova, A. et al. The auxin-inducible degron 2 technology provides sharp degradation control in yeast, mammalian cells, and mice. *Nat. Commun.* **11**, 5701 (2020). 33177522 10.1038/s41467-020-19532-z PMC7659001

[53] Heiss, M., Borland, K., Yoluc, Y. & Kellner, S. Quantification of modified nucleosides in the context of NAIL-MS. *Methods Mol. Biol.* **2298**, 279–306 (2021). 34085252 10.1007/978-1-0716-1374-0_18

[54] Jaqaman, K. et al. Robust single-particle tracking in live-cell time-lapse sequences. *Nat. Methods* **5**, 695–702 (2008). 18641657 10.1038/nmeth.1237 PMC2747604

[55] Lionnet, T. et al. A transgenic mouse for in vivo detection of endogenous labeled mRNA. *Nat. Methods* **8**, 165–170 (2011). 21240280 10.1038/nmeth.1551 PMC3076588

[56] Thompson, R. E., Larson, D. R. & Webb, W. W. Precise nanometer localization analysis for individual fluorescent probes. *Biophys. J.* **82**, 2775–2783 (2002). 11964263 10.1016/S0006-3495(02)75618-X PMC1302065

[57] Mueller, F. et al. FISH-quant: automatic counting of transcripts in 3D FISH images. *Nat. Methods* **10**, 277–278 (2013). 23538861 10.1038/nmeth.2406

[58] Adivarahan, S. et al. Spatial organization of single mRNPs at different stages of the gene expression pathway. *Mol. Cell* **72**, 727–738 (2018). 30415950 10.1016/j.molcel.2018.10.010 PMC6592633

[59] Li, S. et al. Payload distribution and capacity of mRNA lipid nanoparticles. *Nat. Commun.* **13**, 5561 (2022). 36151112 10.1038/s41467-022-33157-4 PMC9508184

[60] Andrews, S. et al. FastQC. A quality control tool for high throughput sequence data. *Babraham Bioinformatics* https://www.bioinformatics.babraham.ac.uk/projects/fastqc/ (2010).

[61] Krueger, F. Trim galore. A wrapper tool around Cutadapt and FastQC to consistently apply quality and adapter trimming to FastQ files. *Babraham Bioinformatics* https://www.bioinformatics.babraham.ac.uk/projects/trim_galore/ (2015).

[62] Langmead, B. & Salzberg, S. L. Fast gapped-read alignment with Bowtie 2. *Nat. Methods* **9**, 357–359 (2012). 22388286 10.1038/nmeth.1923 PMC3322381

[63] Langmead, B., Wilks, C., Antonescu, V. & Charles, R. Scaling read aligners to hundreds of threads on general-purpose processors. *Bioinformatics* **35**, 421–432 (2019). 30020410 10.1093/bioinformatics/bty648 PMC6361242

[64] Li, H. et al. The Sequence Alignment/Map format and SAMtools. *Bioinformatics* **25**, 2078–2079 (2009). 19505943 10.1093/bioinformatics/btp352 PMC2723002

[65] Okonechnikov, K., Conesa, A. & Garcia-Alcalde, F. Qualimap 2: advanced multi-sample quality control for high-throughput sequencing data. *Bioinformatics* **32**, 292–294 (2016). 26428292 10.1093/bioinformatics/btv566 PMC4708105

[66] Robinson, J. T. et al. Integrative genomics viewer. *Nat. Biotechnol.* **29**, 24–26 (2011). 21221095 10.1038/nbt.1754 PMC3346182

[67] Thorvaldsdottir, H., Robinson, J. T. & Mesirov, J. P. Integrative Genomics Viewer (IGV): high-performance genomics data visualization and exploration. *Brief. Bioinform.* **14**, 178–192 (2013). 22517427 10.1093/bib/bbs017 PMC3603213

[68] Ingolia, N. T., Brar, G. A., Rouskin, S., McGeachy, A. M. & Weissman, J. S. The ribosome profiling strategy for monitoring translation in vivo by deep sequencing of ribosome-protected mRNA fragments. *Nat. Protoc.* **7**, 1534–1550 (2012). 22836135 10.1038/nprot.2012.086 PMC3535016

[69] Gutierrez, C. S. et al. Pseudouridine residues as substrates for serum ribonucleases. *RNA* **31**, 1542–1556 (2025). 40835455 10.1261/rna.080404.125 PMC12530130

[70] Gerashchenko, M. V. & Gladyshev, V. N. Ribonuclease selection for ribosome profiling. *Nucleic Acids Res.* **45**, e6 (2017). 27638886 10.1093/nar/gkw822 PMC5314788

[71] Smith, T., Heger, A. & Sudbery, I. UMI-tools: modeling sequencing errors in unique molecular identifiers to improve quantification accuracy. *Genome Res.* **27**, 491–499 (2017). 28100584 10.1101/gr.209601.116 PMC5340976

[72] Langmead, B., Trapnell, C., Pop, M. & Salzberg, S. L. Ultrafast and memory-efficient alignment of short DNA sequences to the human genome. *Genome Biol.* **10**, R25 (2009). 19261174 10.1186/gb-2009-10-3-r25 PMC2690996

