# Supplementary material for: N4-Acetylcytidine enhances synthetic mRNA translation yield and fidelity
Source: Nature. 2026 Jul 1;656(8129):1034–43. doi: 10.1038/s41586-026-10729-8 (PMC13518219; doi:10.1038/s41586-026-10729-8)

nature portfolio

https://doi.org/10.1038/s41586-026-10729-8

Supplementary information

N^4-Acetylcytidine enhances synthetic mRNA translation yield and fidelity

In the format provided by the
authors and unedited

Fig.1e NanoLuc Extended Data Figure 1g

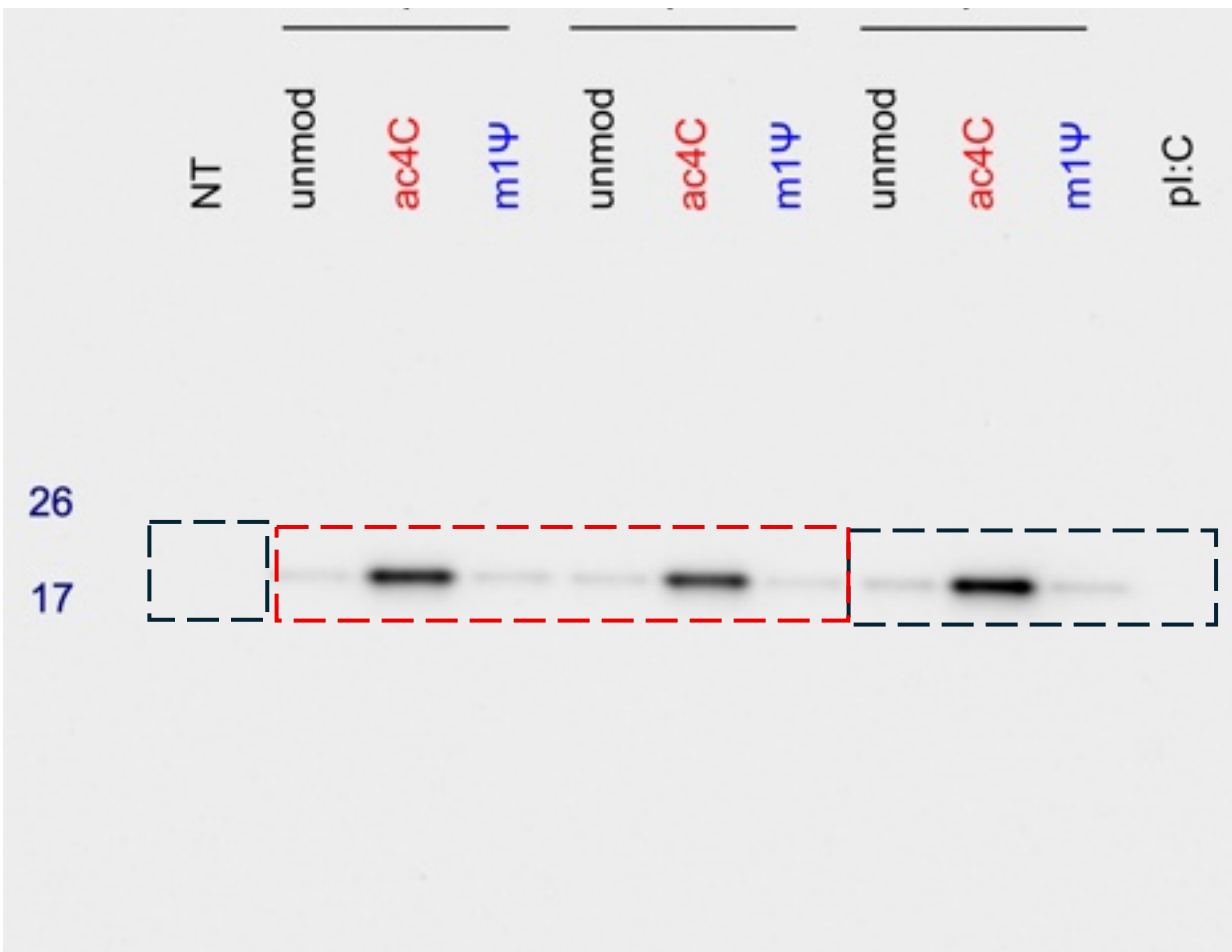

Fig.1e p-eIF2-α Extended Data Figure 1g

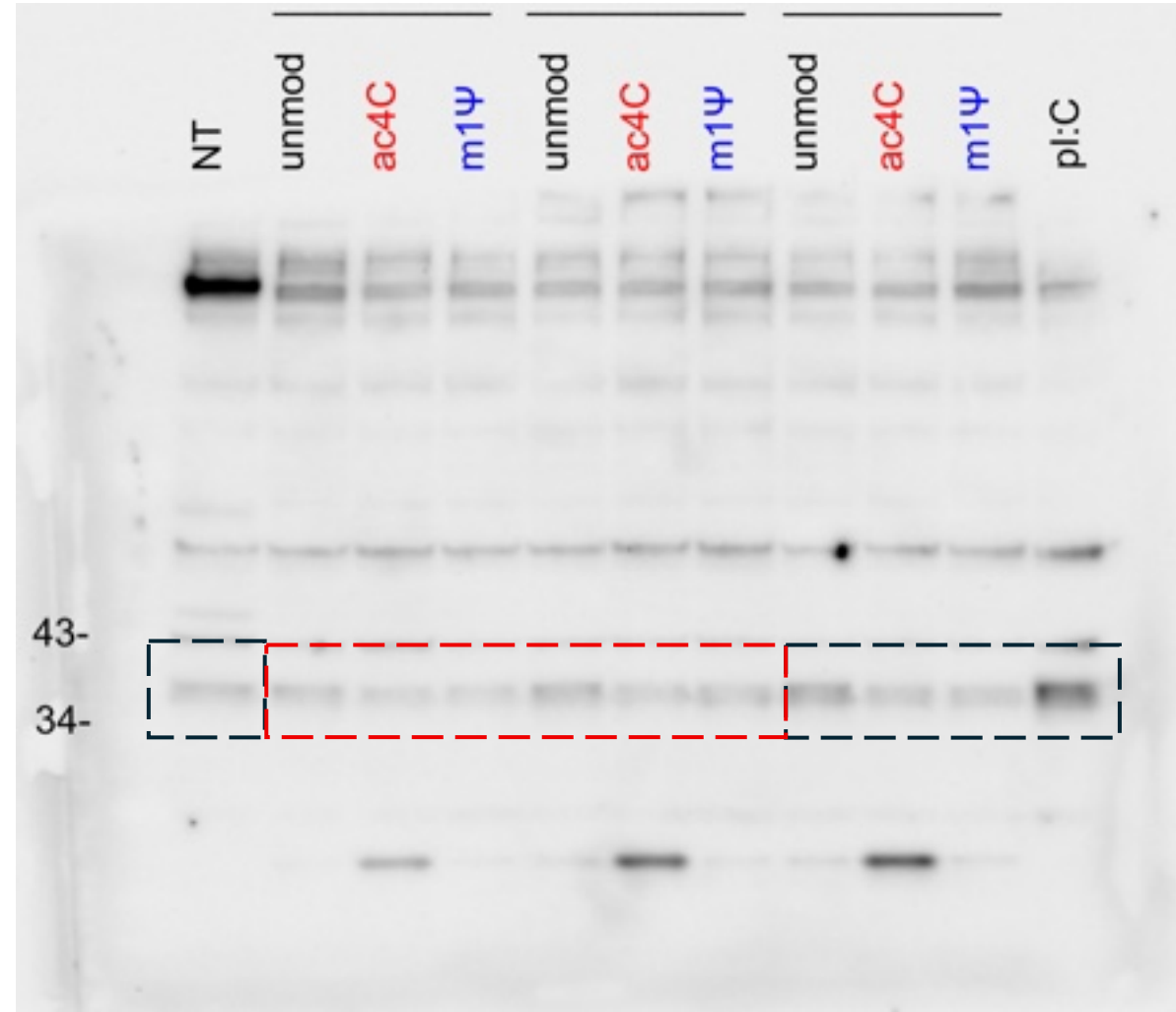

Fig.1e eIF2-α Extended Data Figure 1g

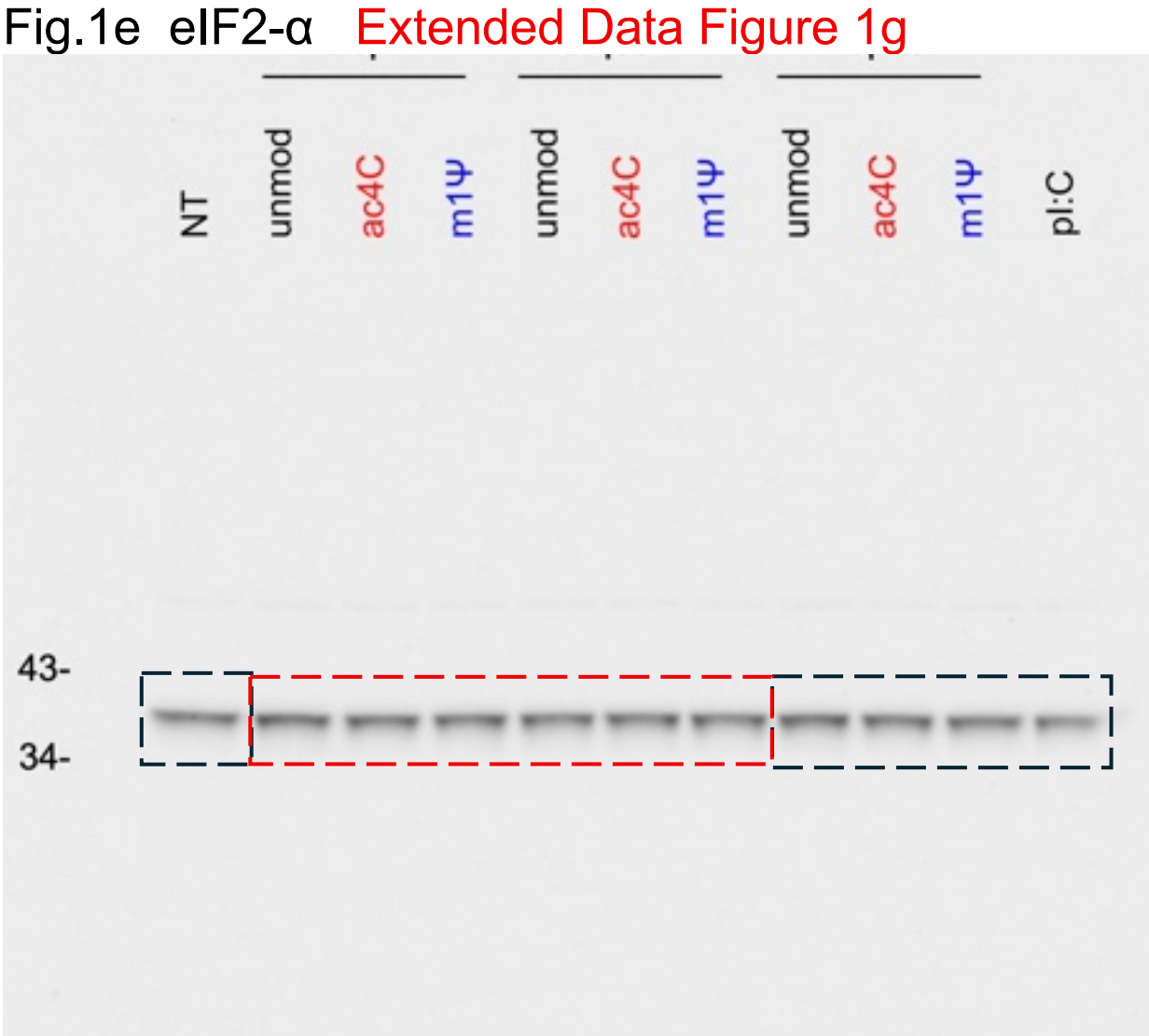

Fig.1e β-tubulin Extended Data Figure 1g

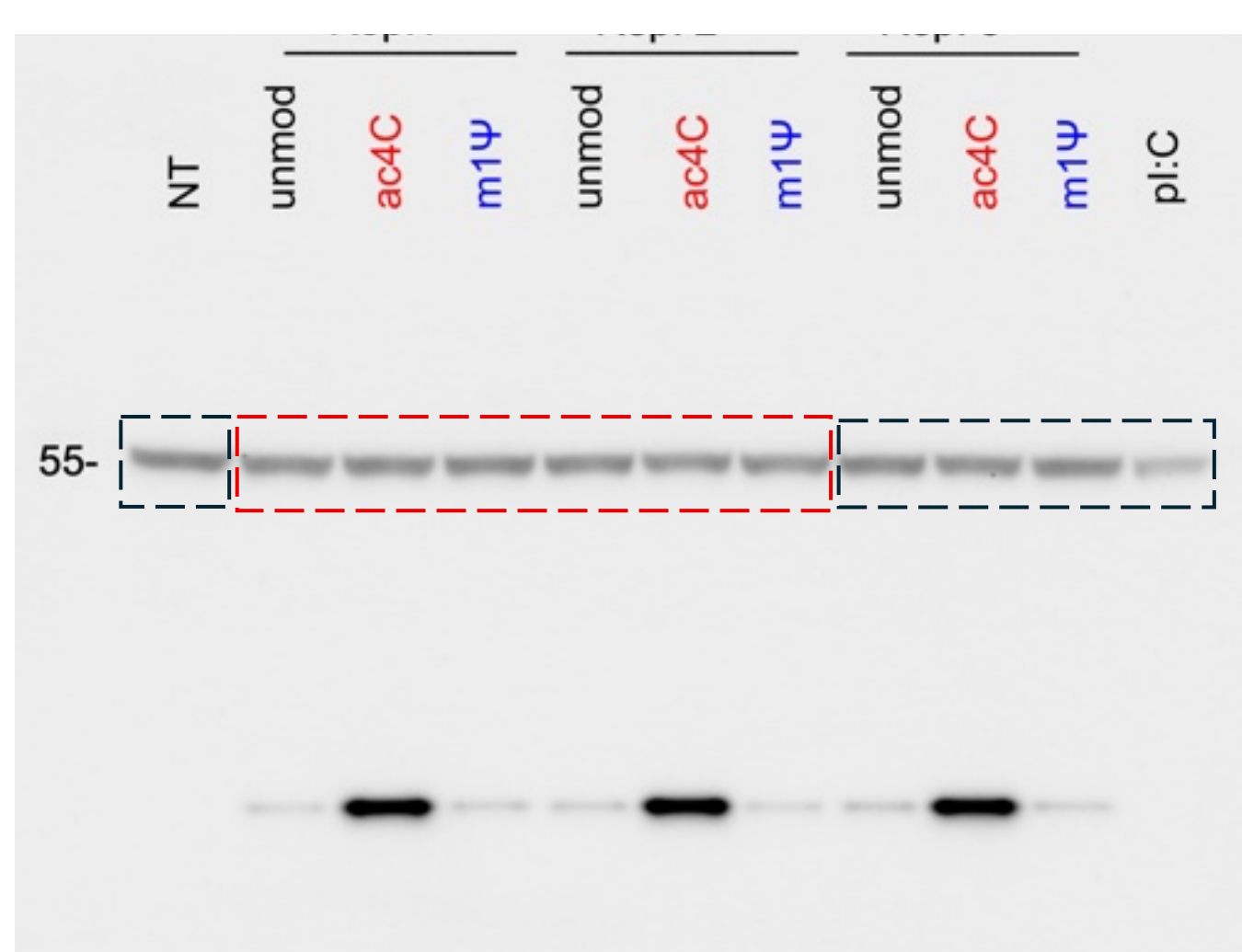

Fig.1i NanoLuc Extended Data Figure 1i

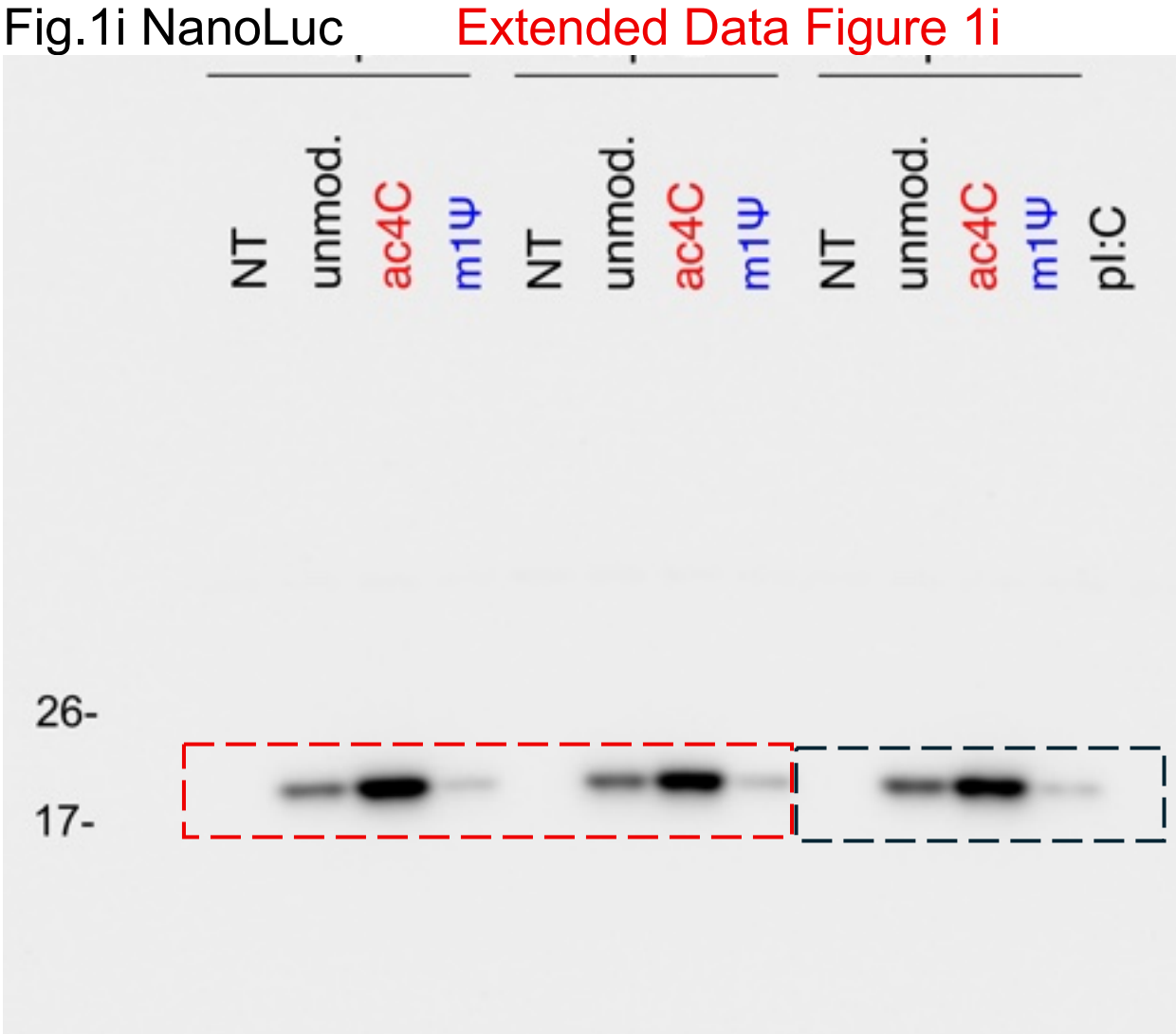

Fig.1i p-eIF2-α Extended Data Figure 1i

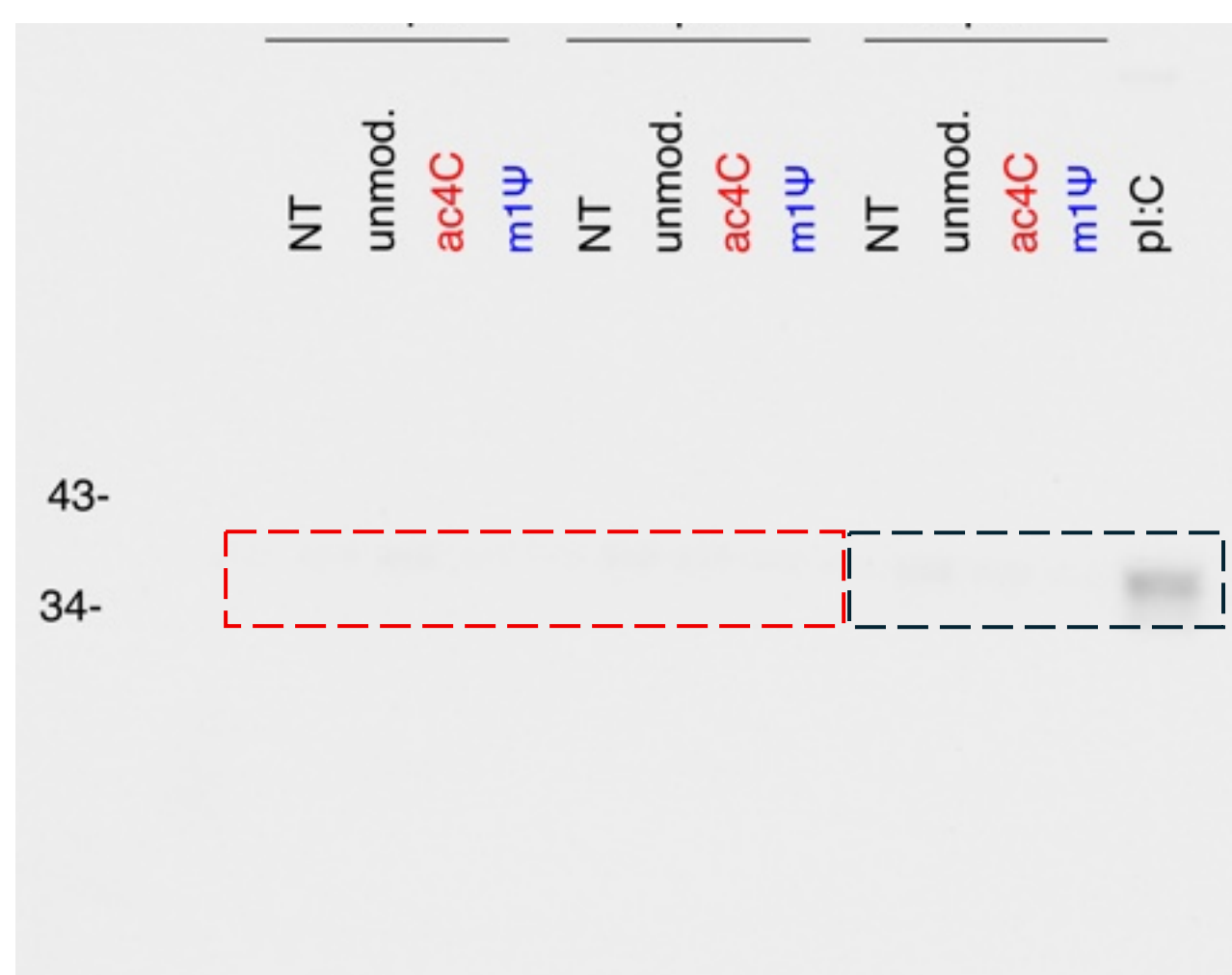

Fig.1i eIF2-α Extended Data Figure 1i

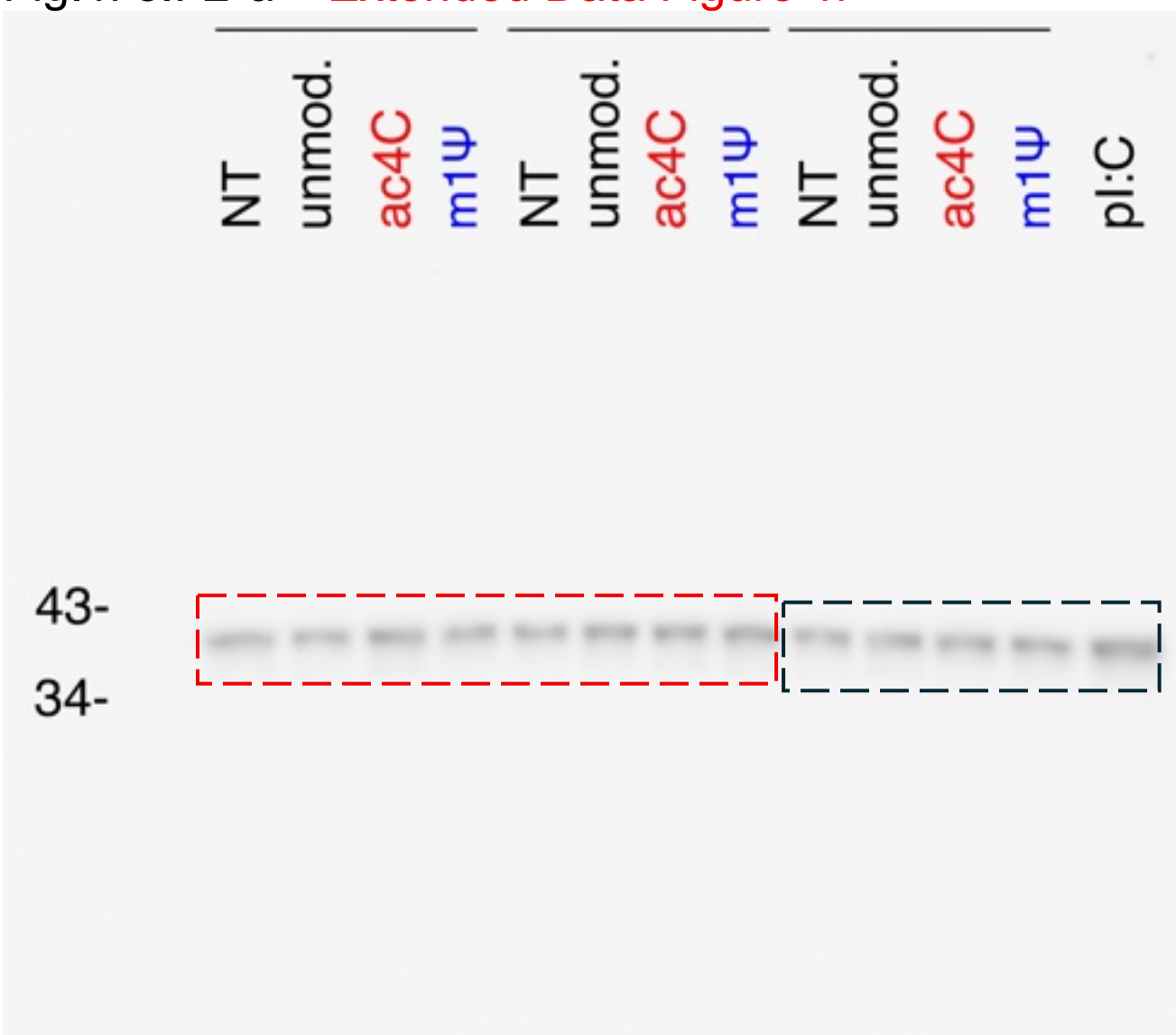

Fig.1i β-tubulin. Extended Data Figure 1i

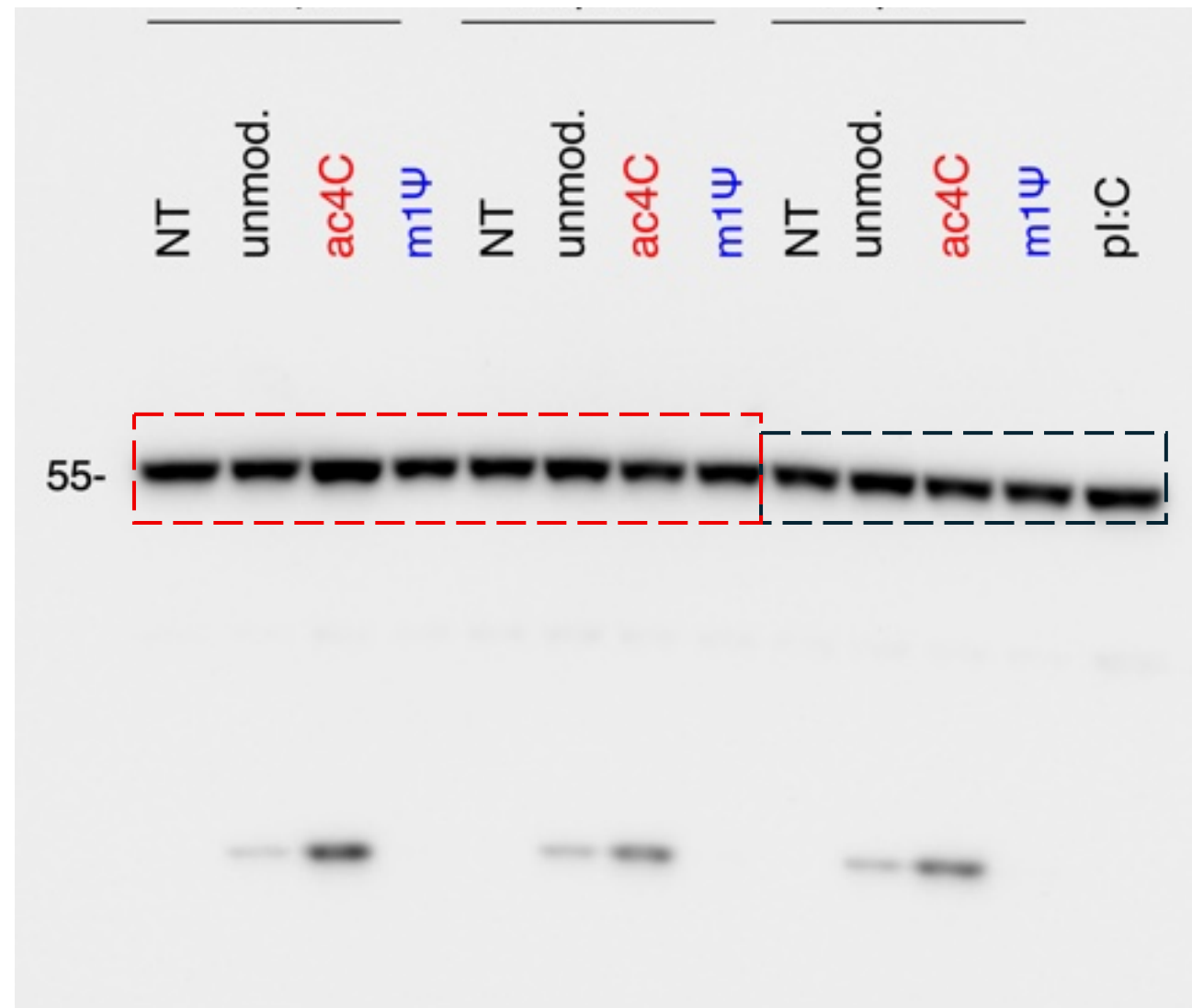

Fig.1l Electroporation NanoLuc

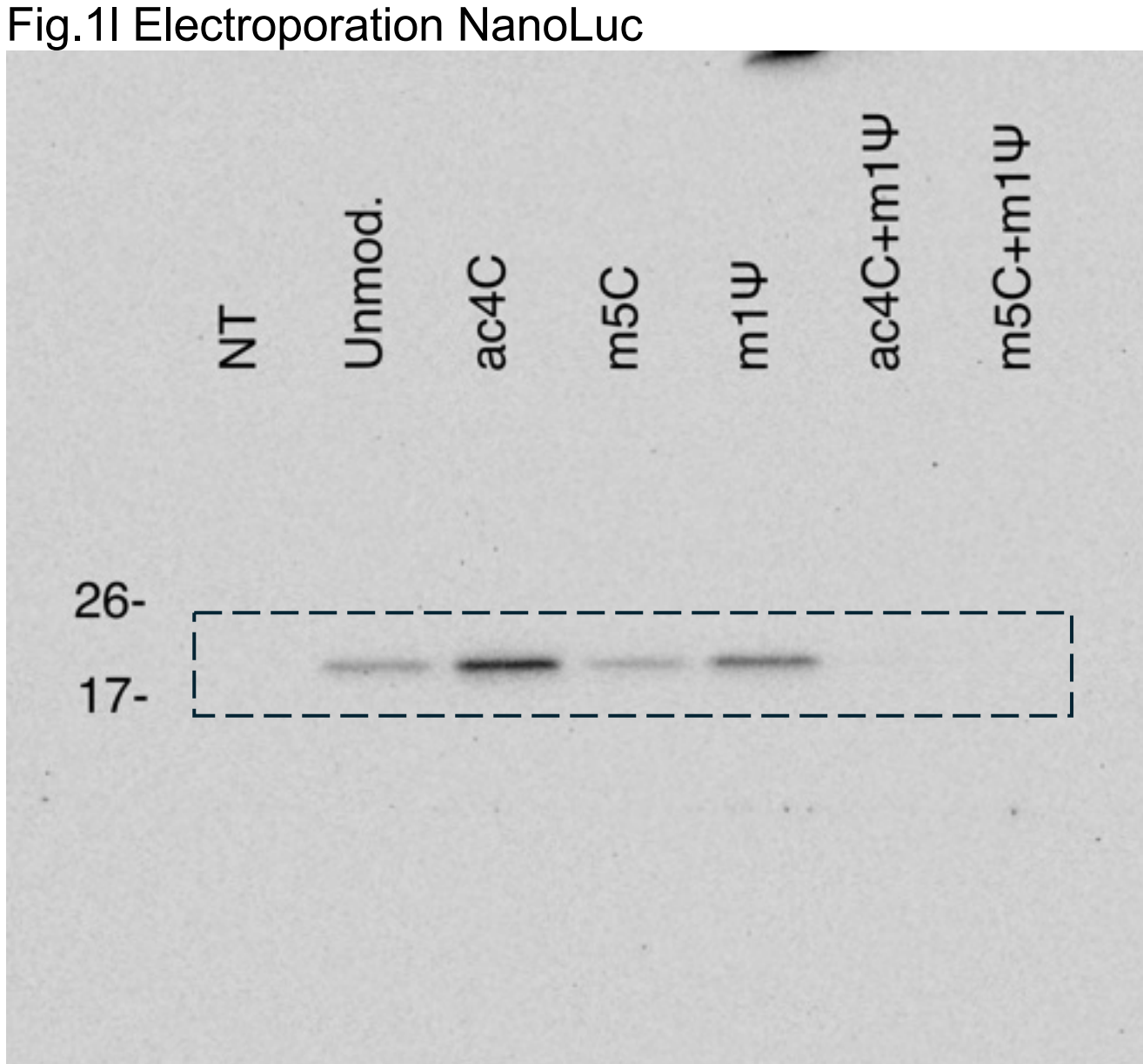

Fig.1l Electroporation β-tubulin

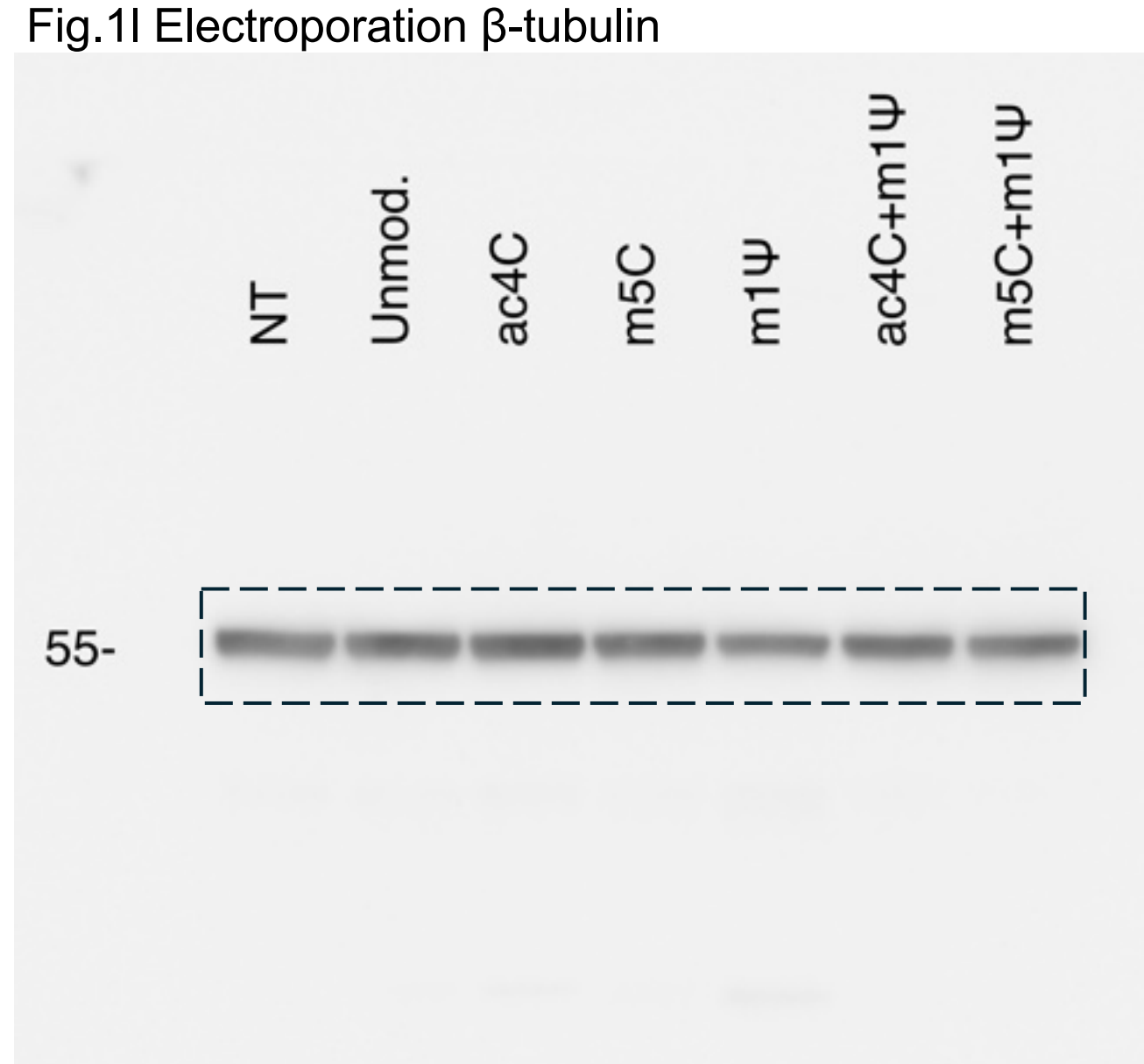

Fig.1o RRL NanoLuc Extended Data Figure 1k

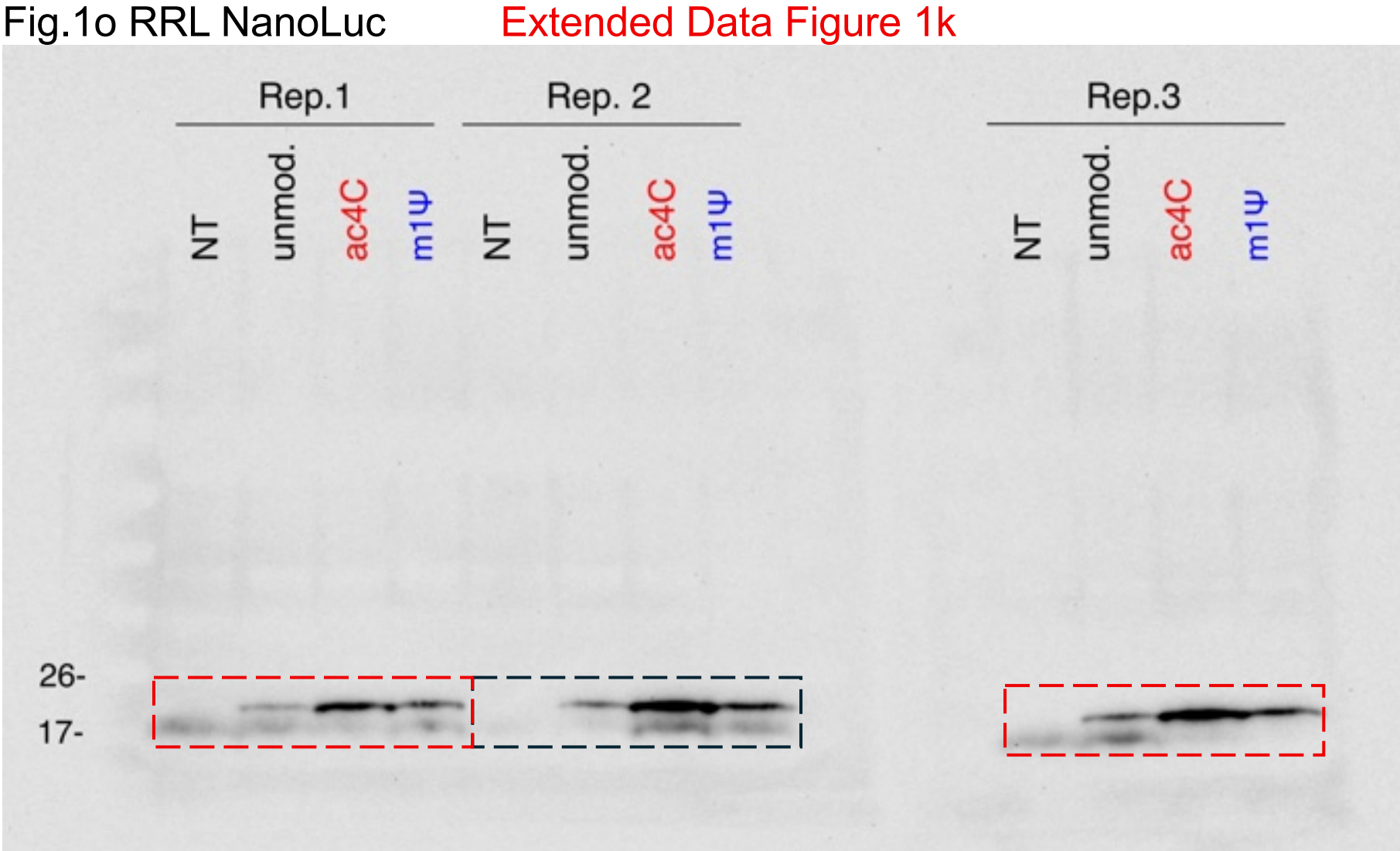

Fig.1o RRL eIF2-α Extended Data Figure 1k

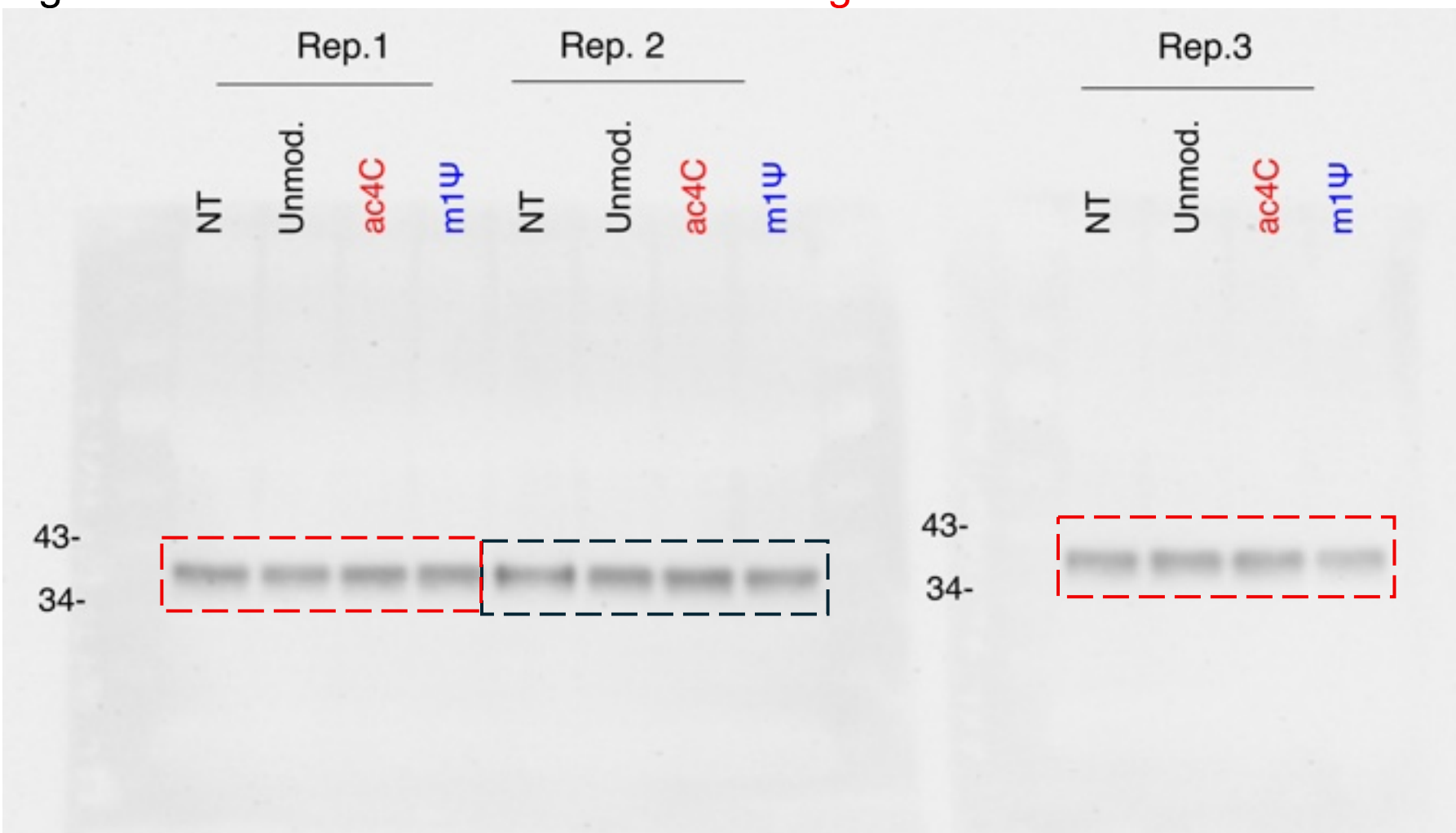

Fig.1o RRL p-eIF2-α Extended Data Figure 1k

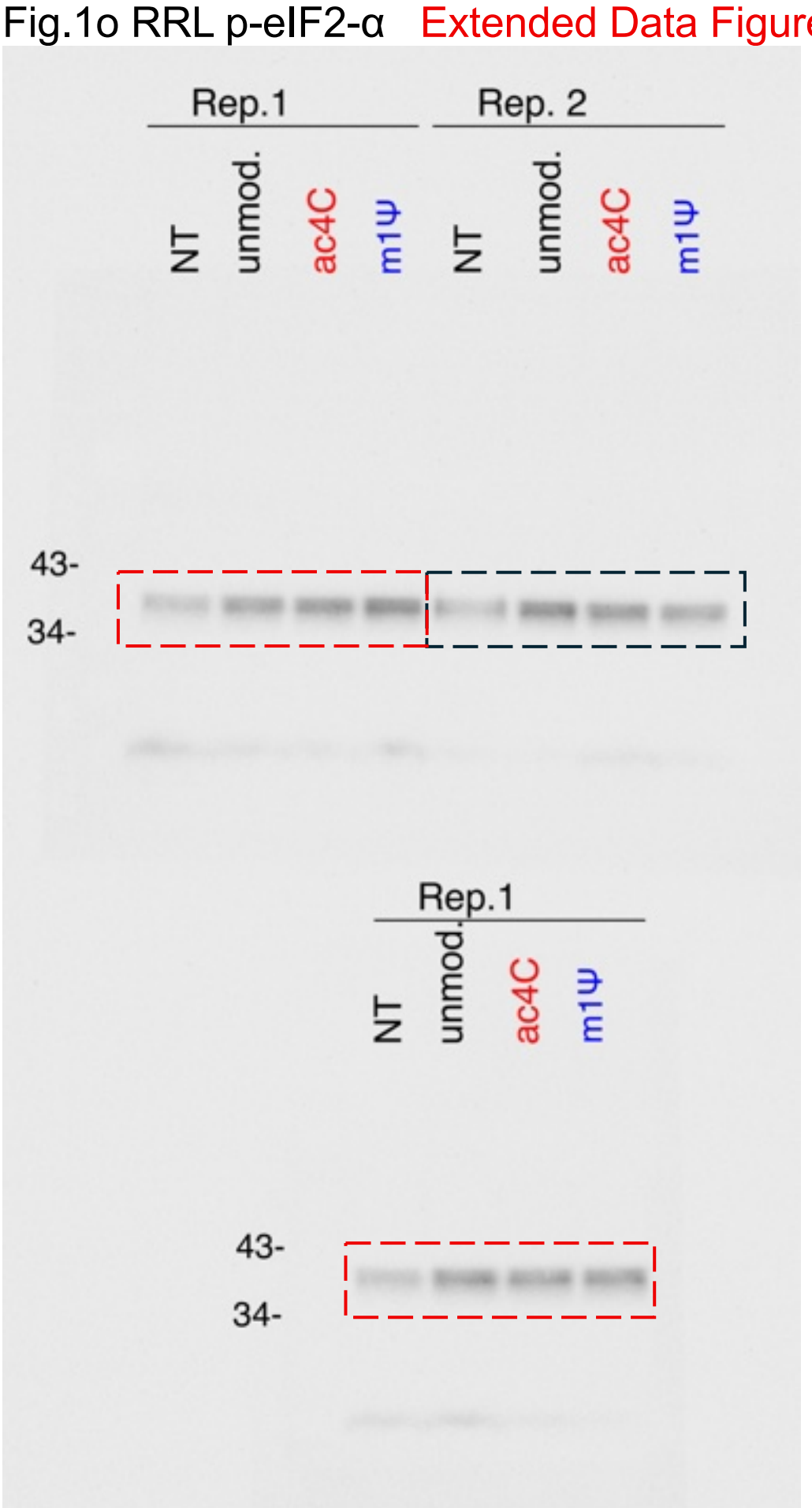

Fig.1q CD25

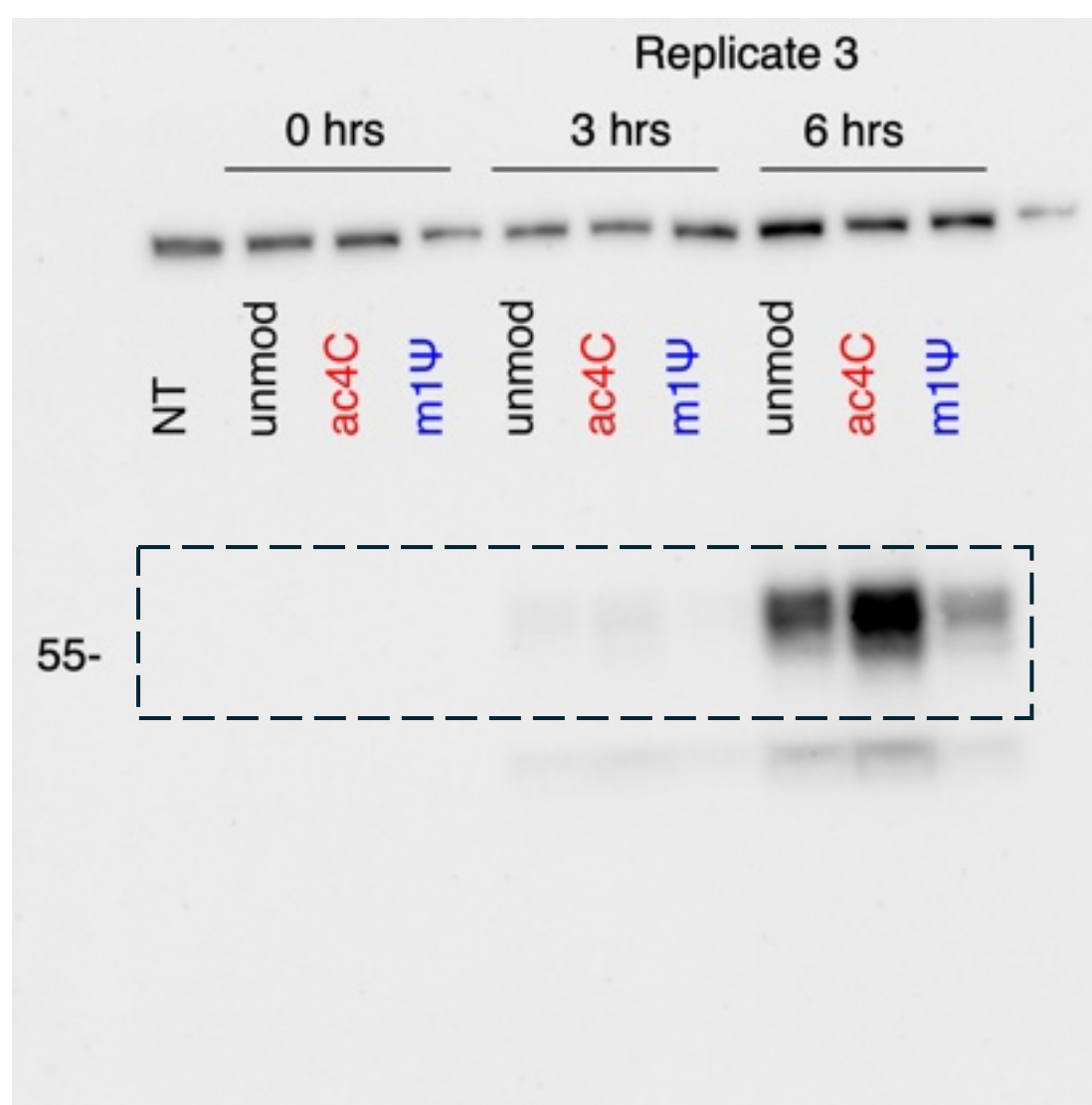

Fig.1q β-tubulin

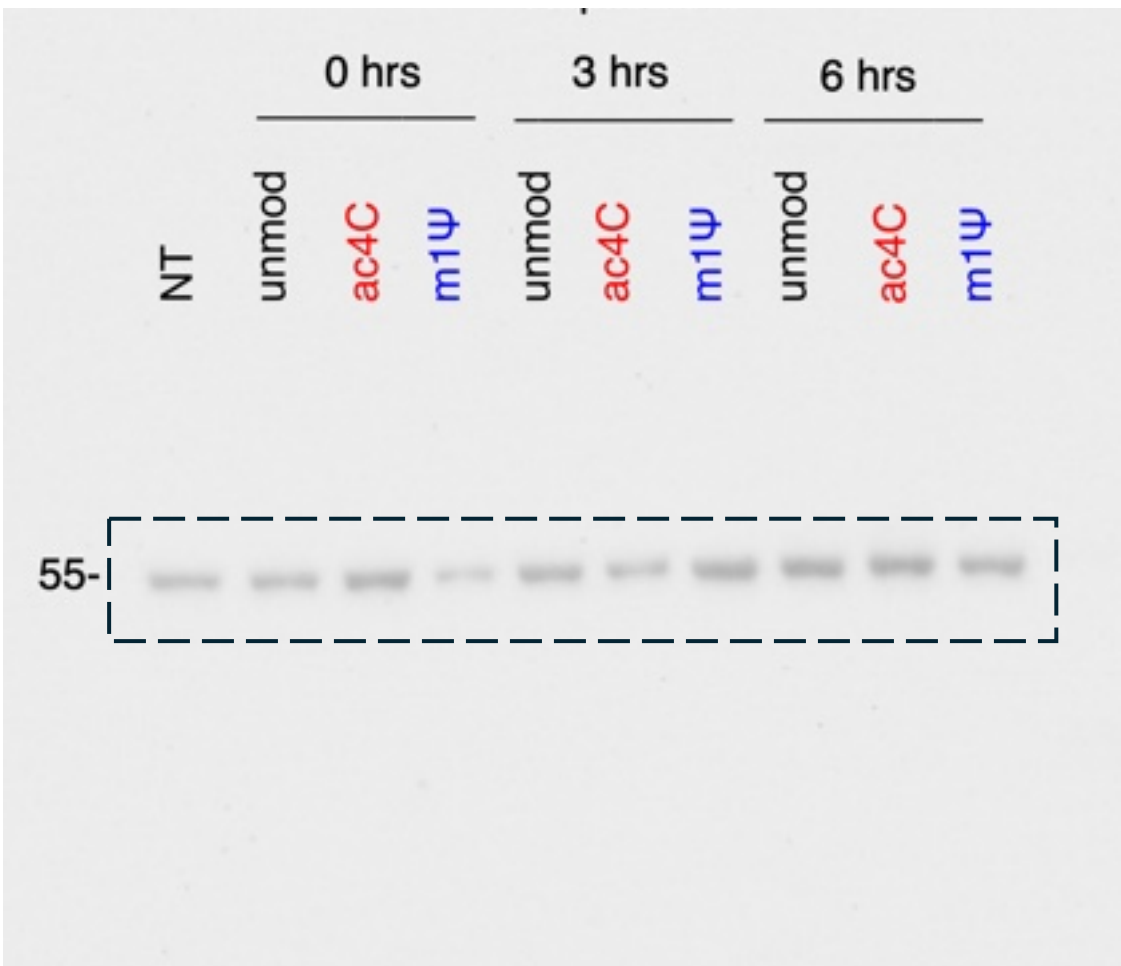

Extended Data Figure 1a Denaturing gel of *NanoLuc* mRNA

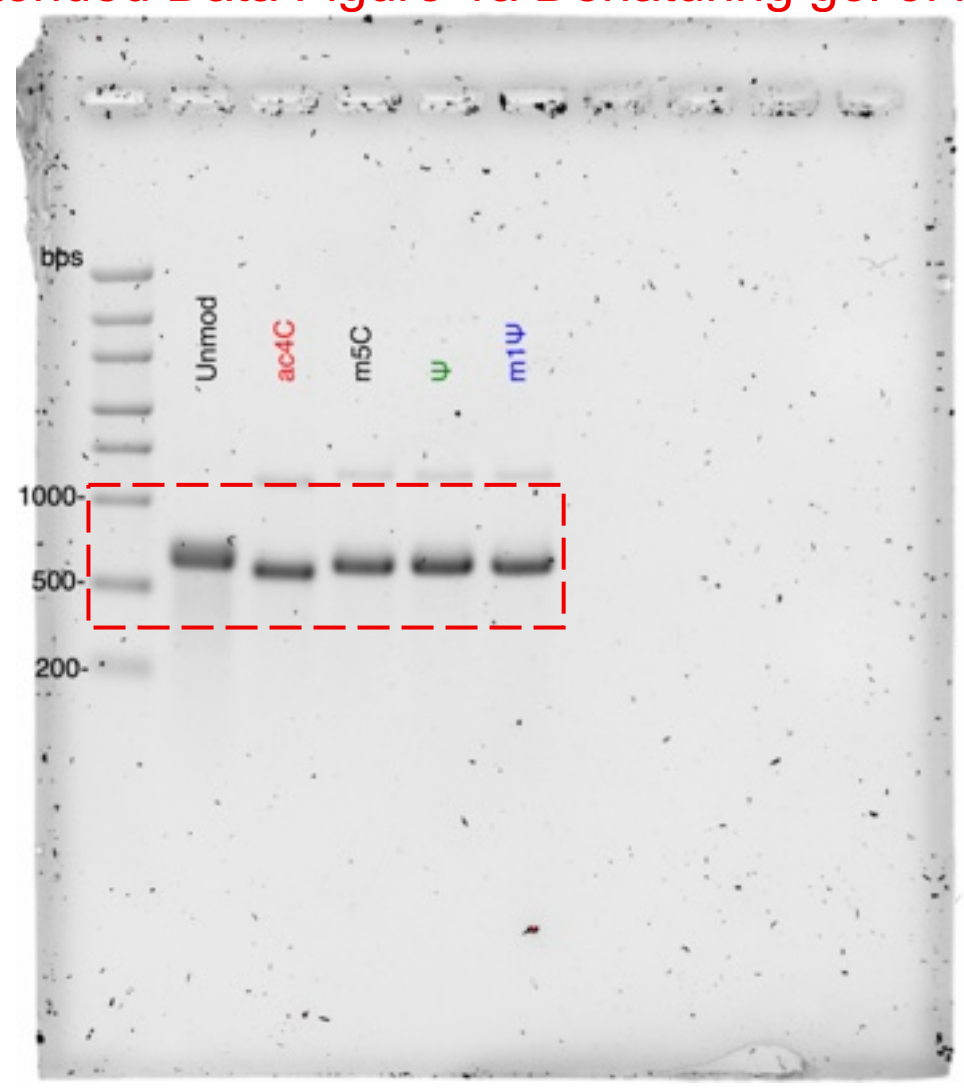

Extended Data Figure 1e NanoLuc

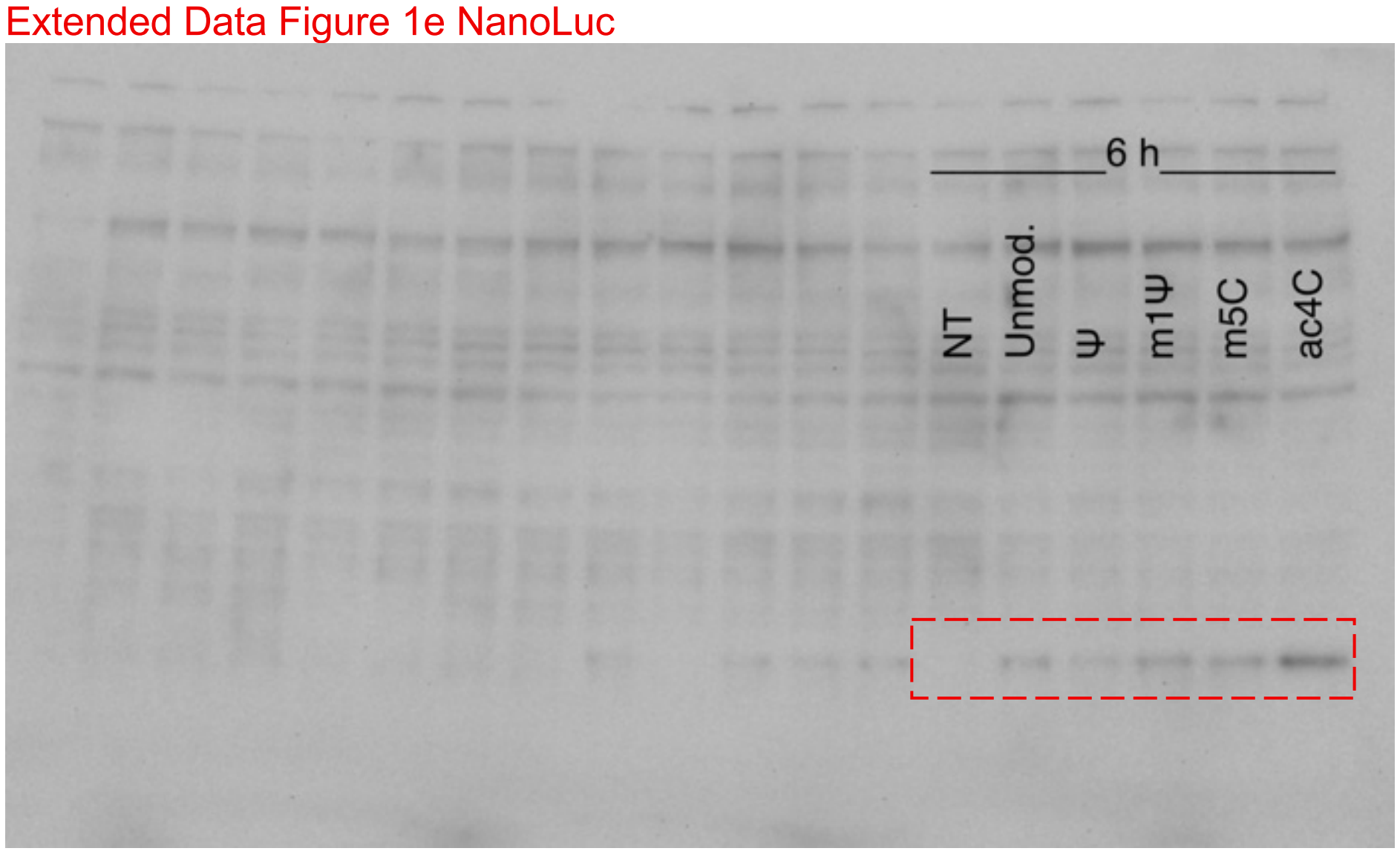

Extended Data Figure 1e GAPDH

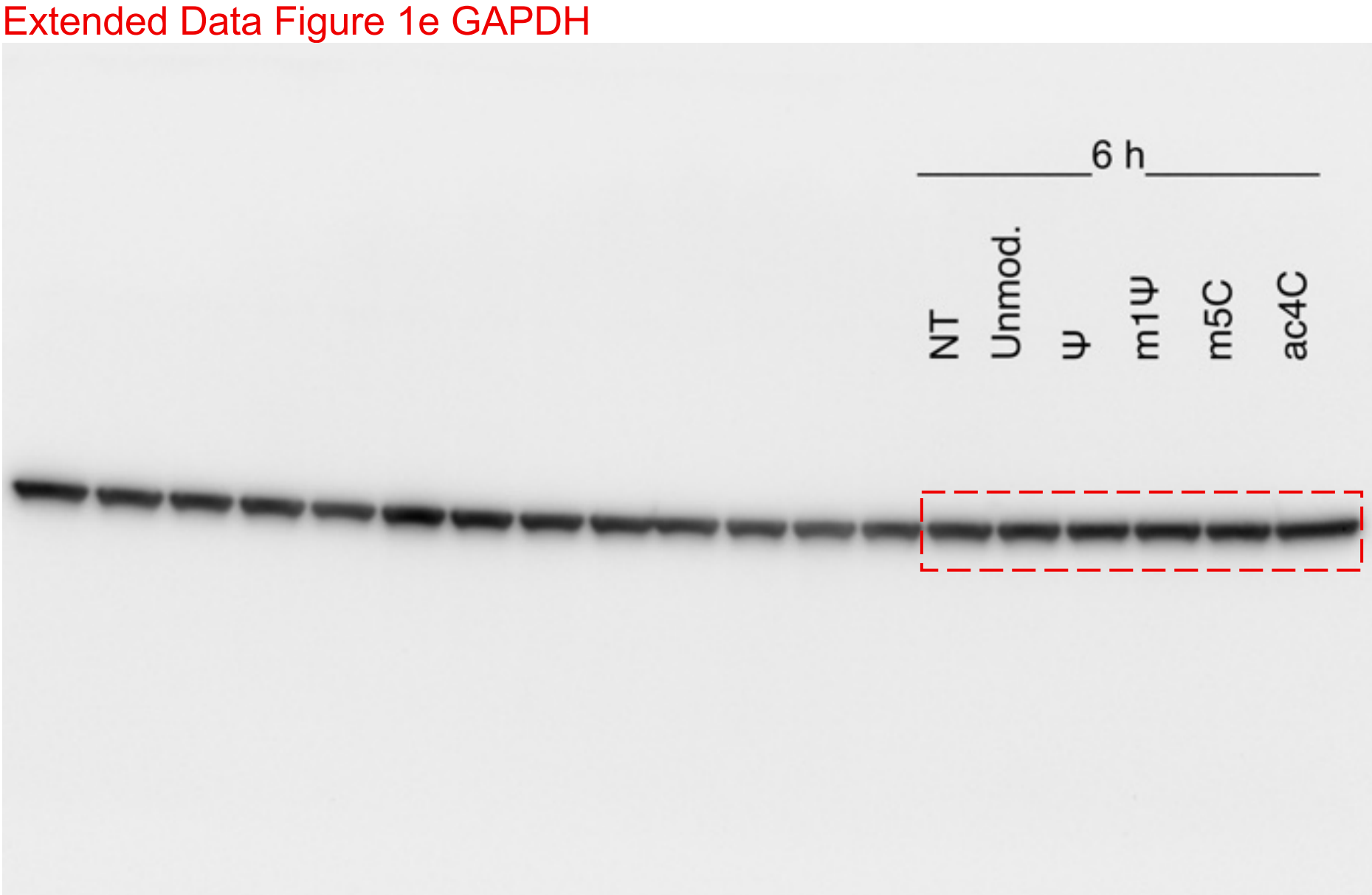

Extended Data Figure 1h *Mutant T7 NanoLuc* mRNA
Denaturing gel

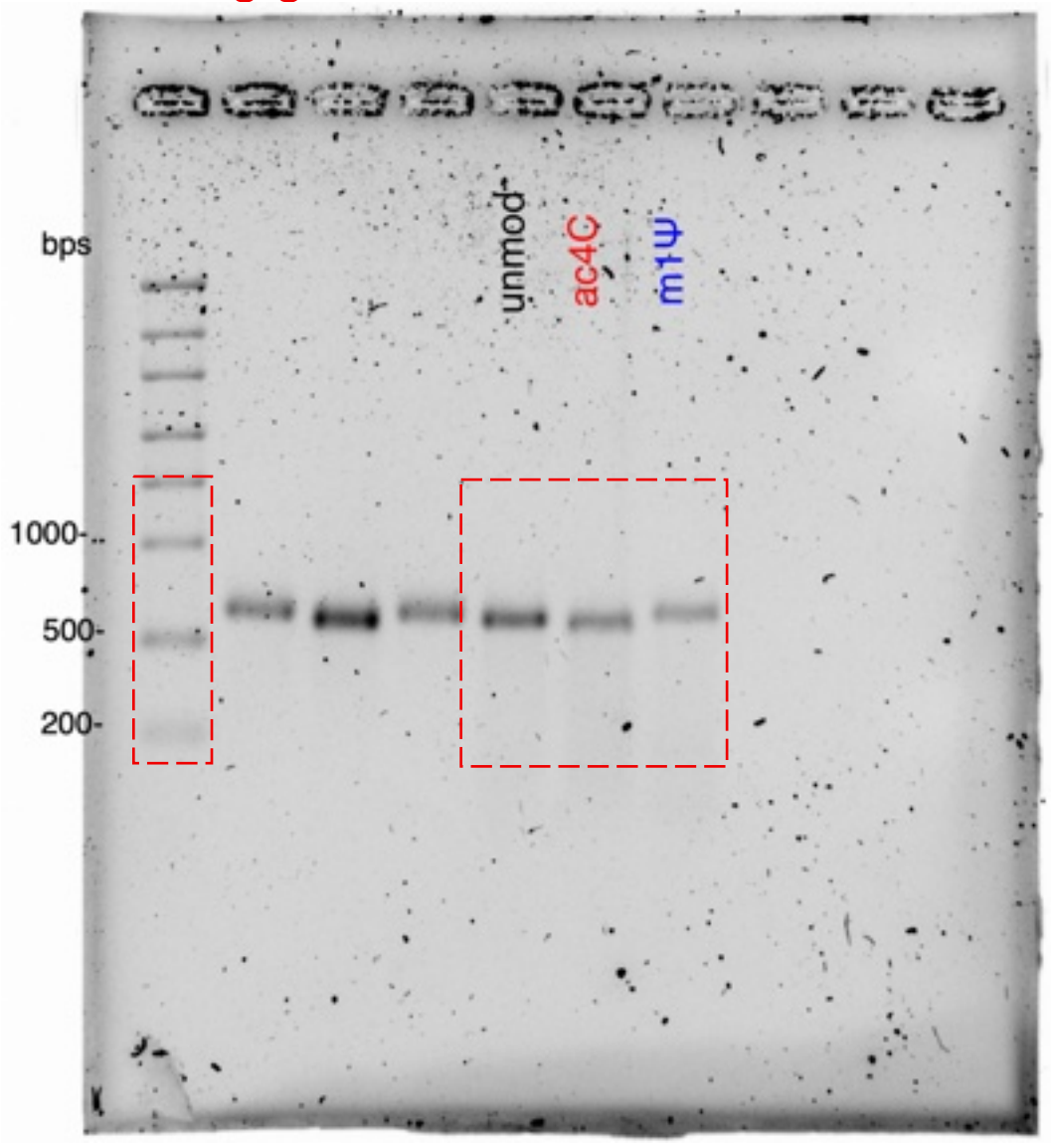

Extended Data Figure 1j *NanoLuc* mRNA
Denaturing gel

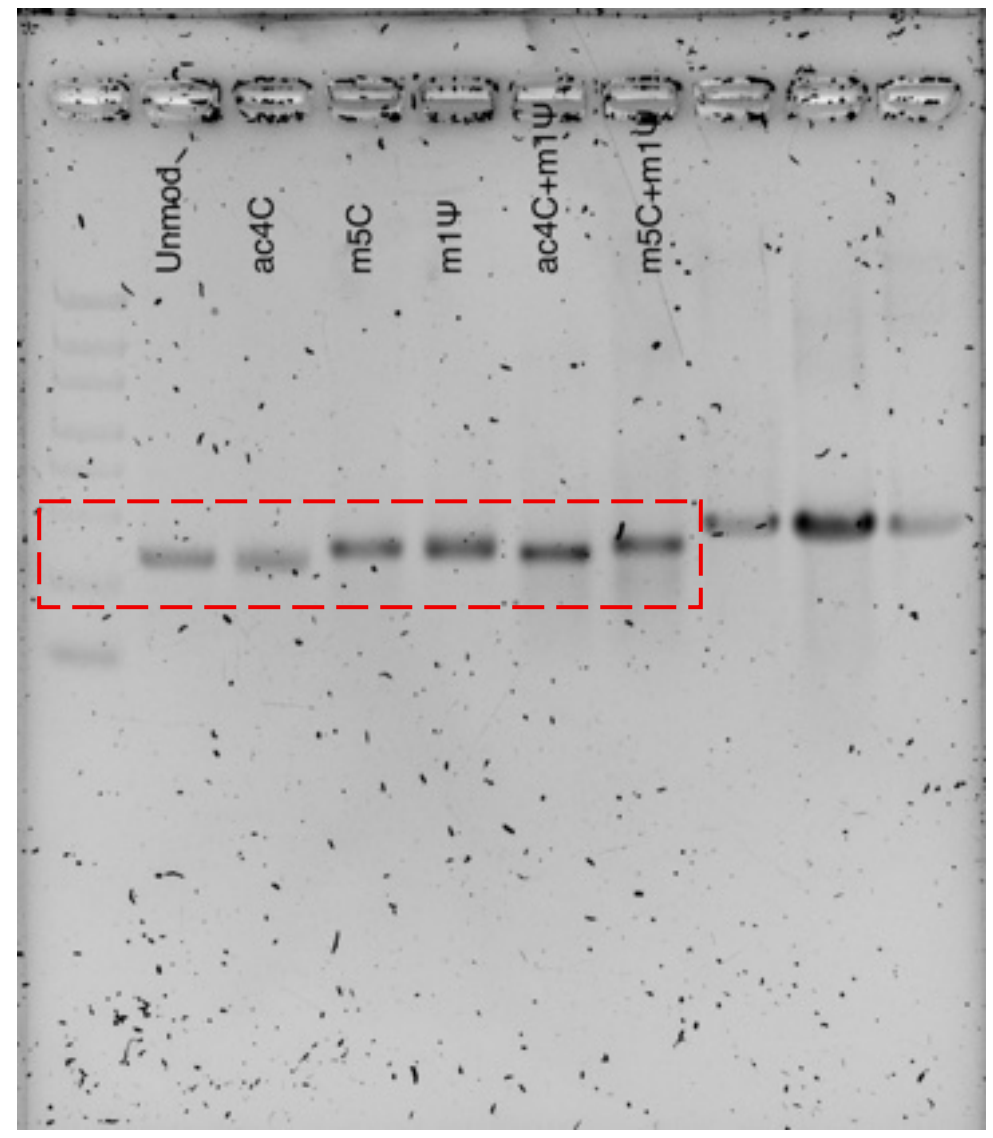

Extended Data Figure 2a *C=U NanoLuc* mRNA.
Denaturing gel

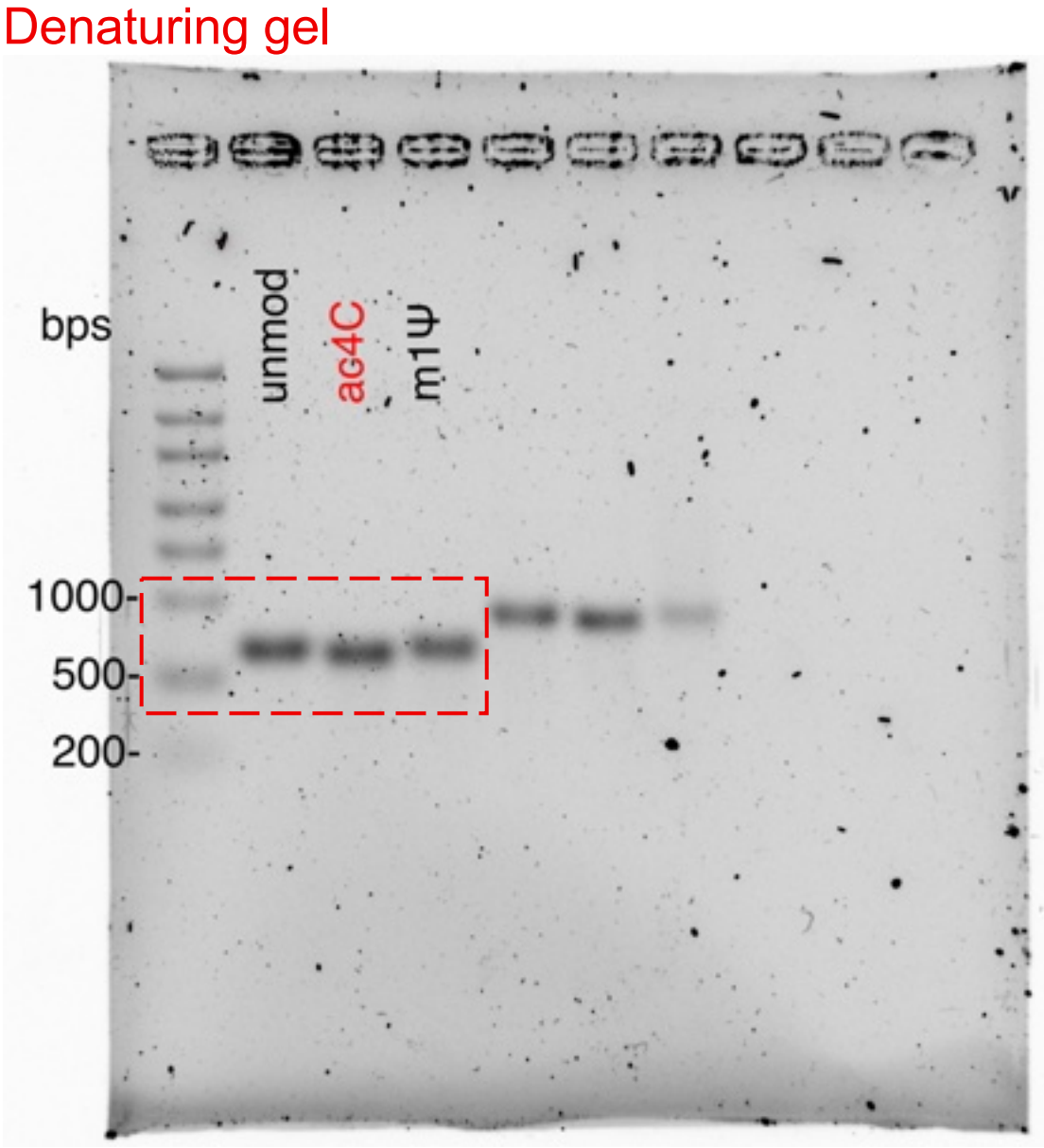

Extended Data Figure 2g *CD25* mRNA
Denaturing gel

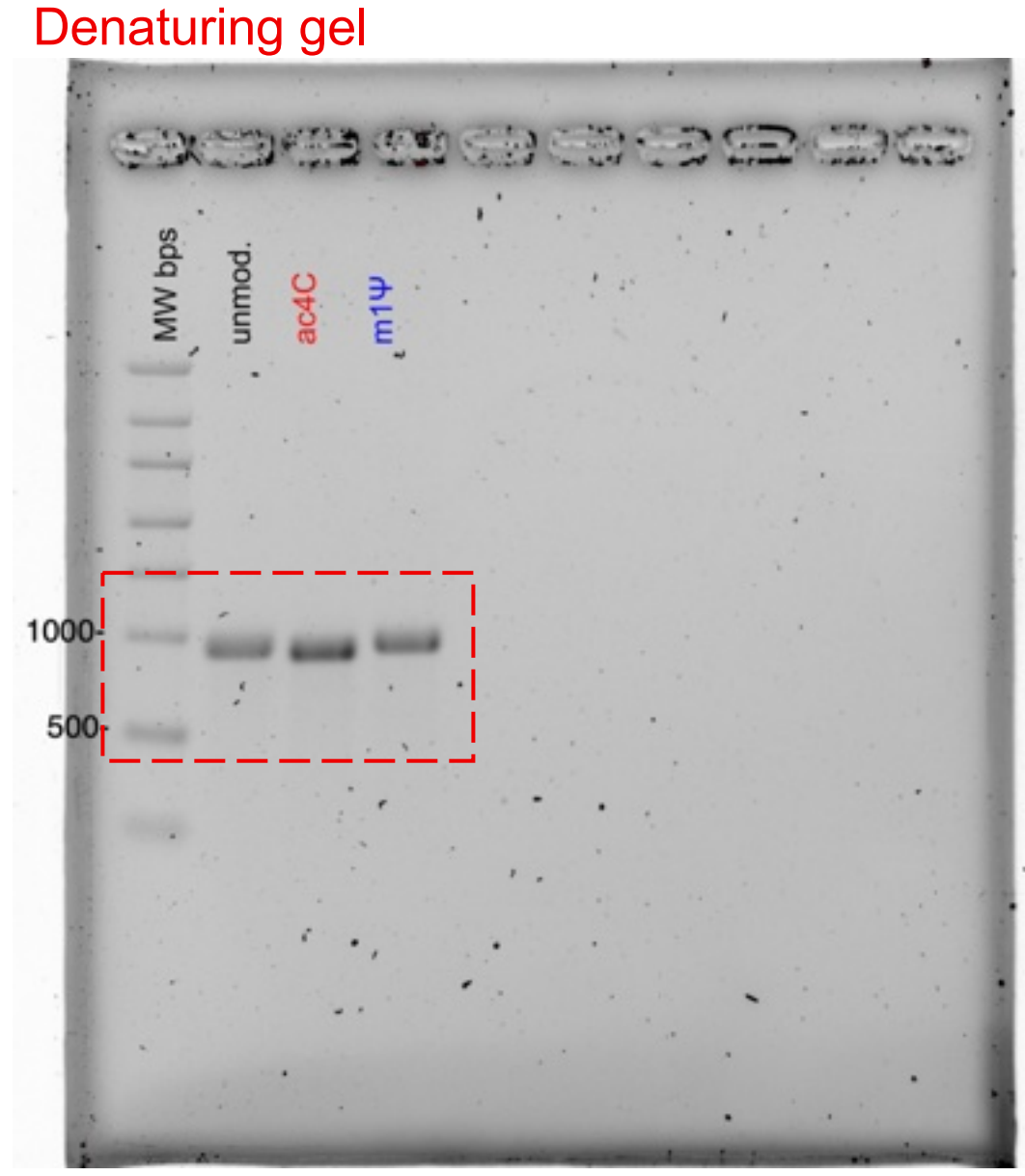

Extended Data Figure 2e NanoLuc

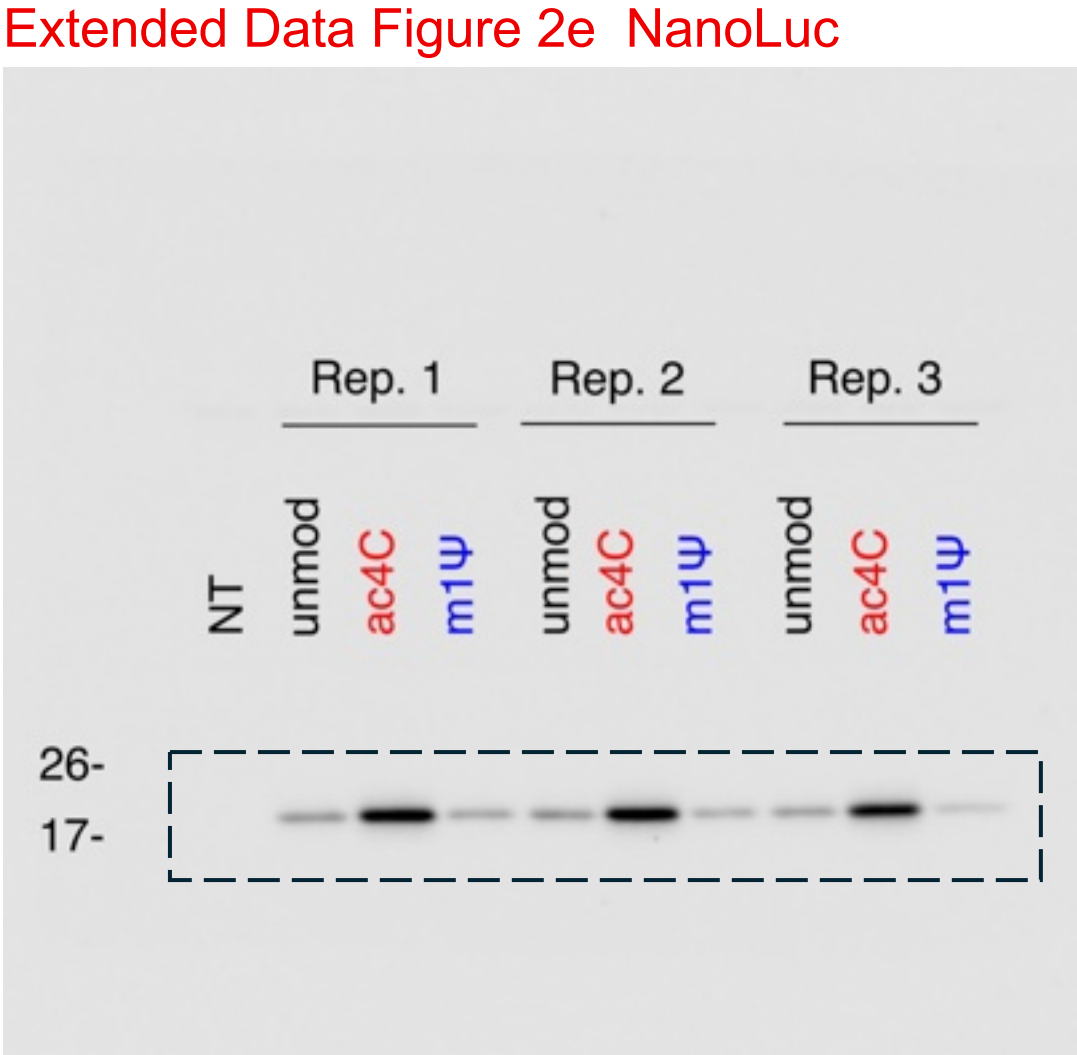

Extended Data Figure 2e p-eIF2-α

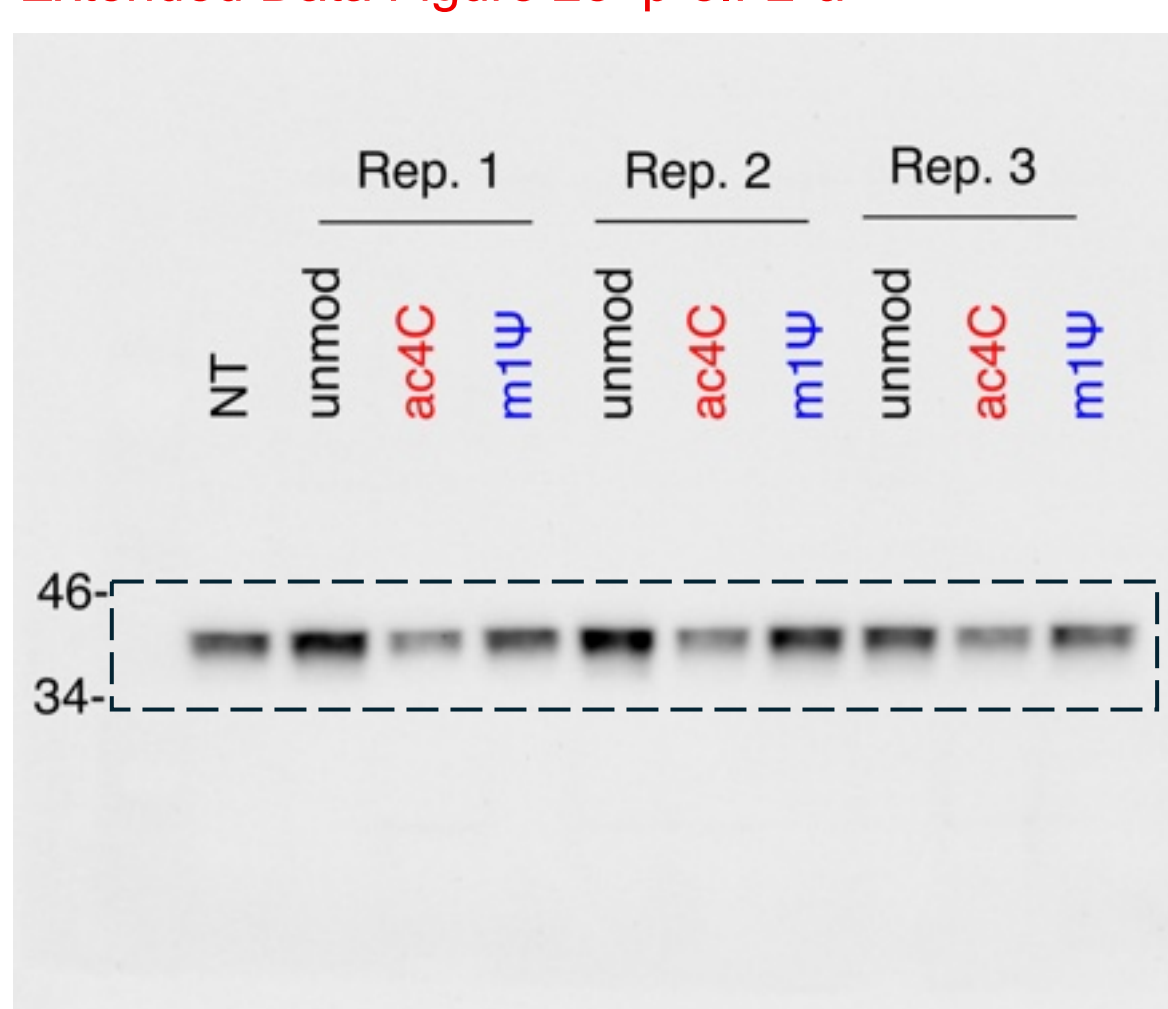

Extended Data Figure 2e eIF2-α

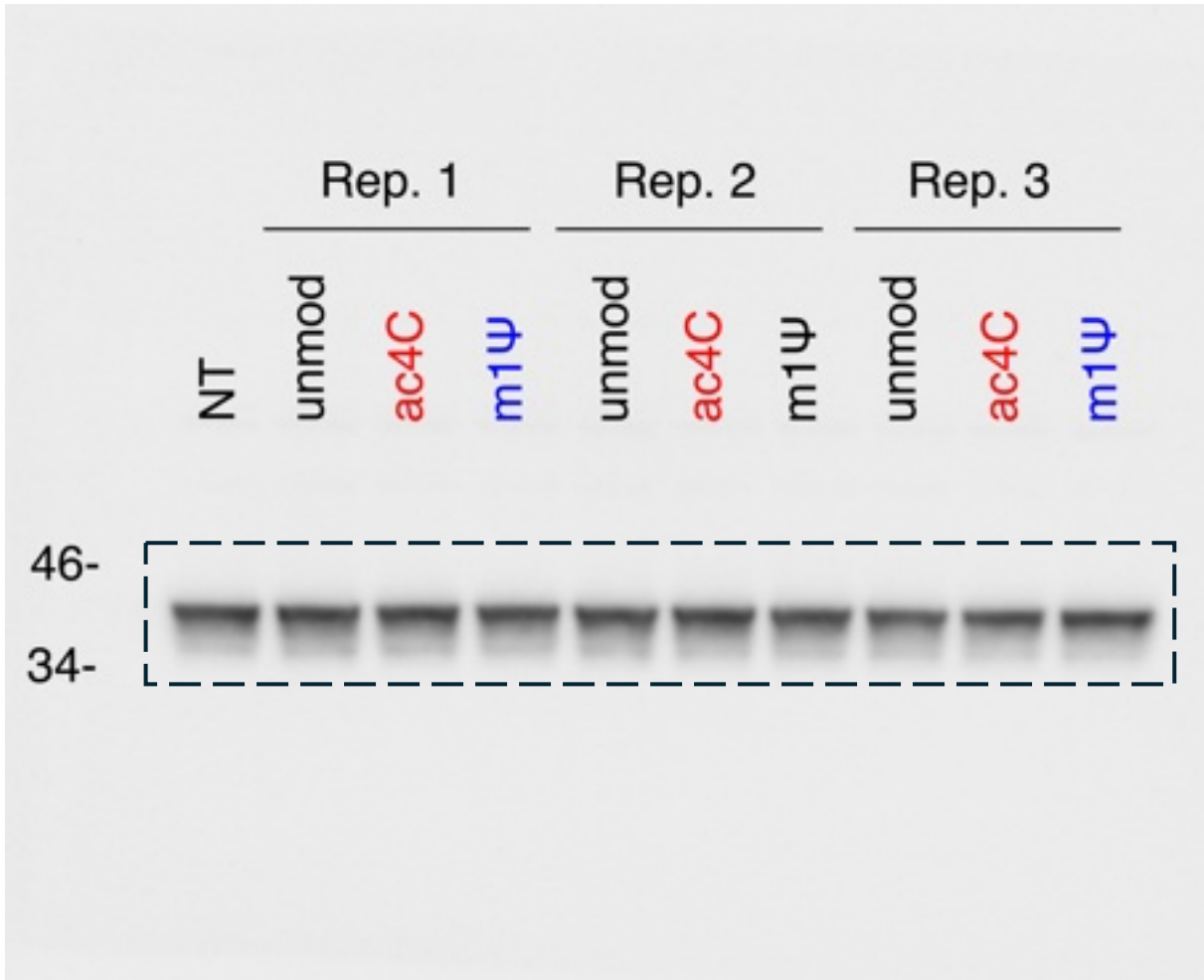

Extended Data Figure 2e β-tubulin

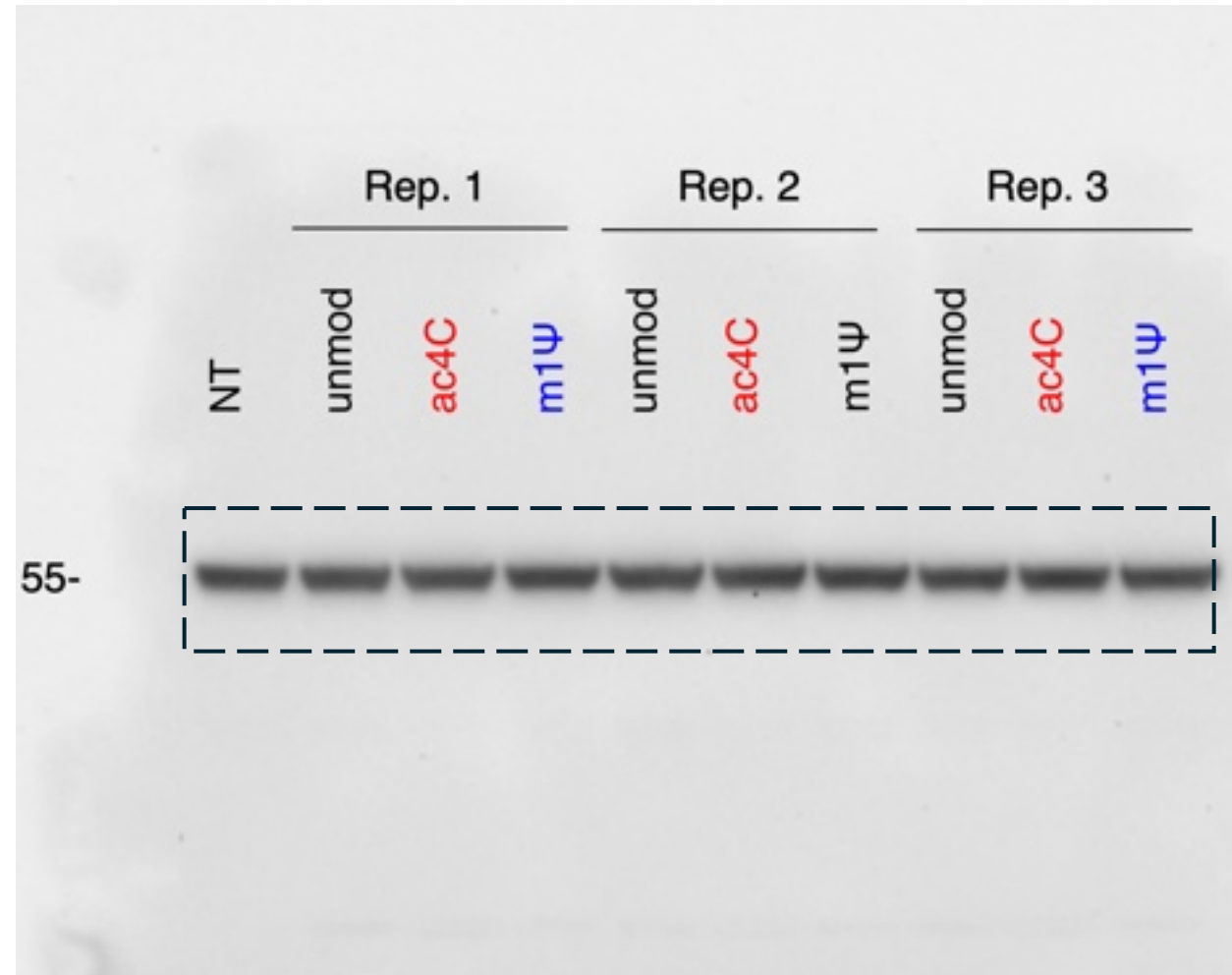

Extended Data Figure 2i

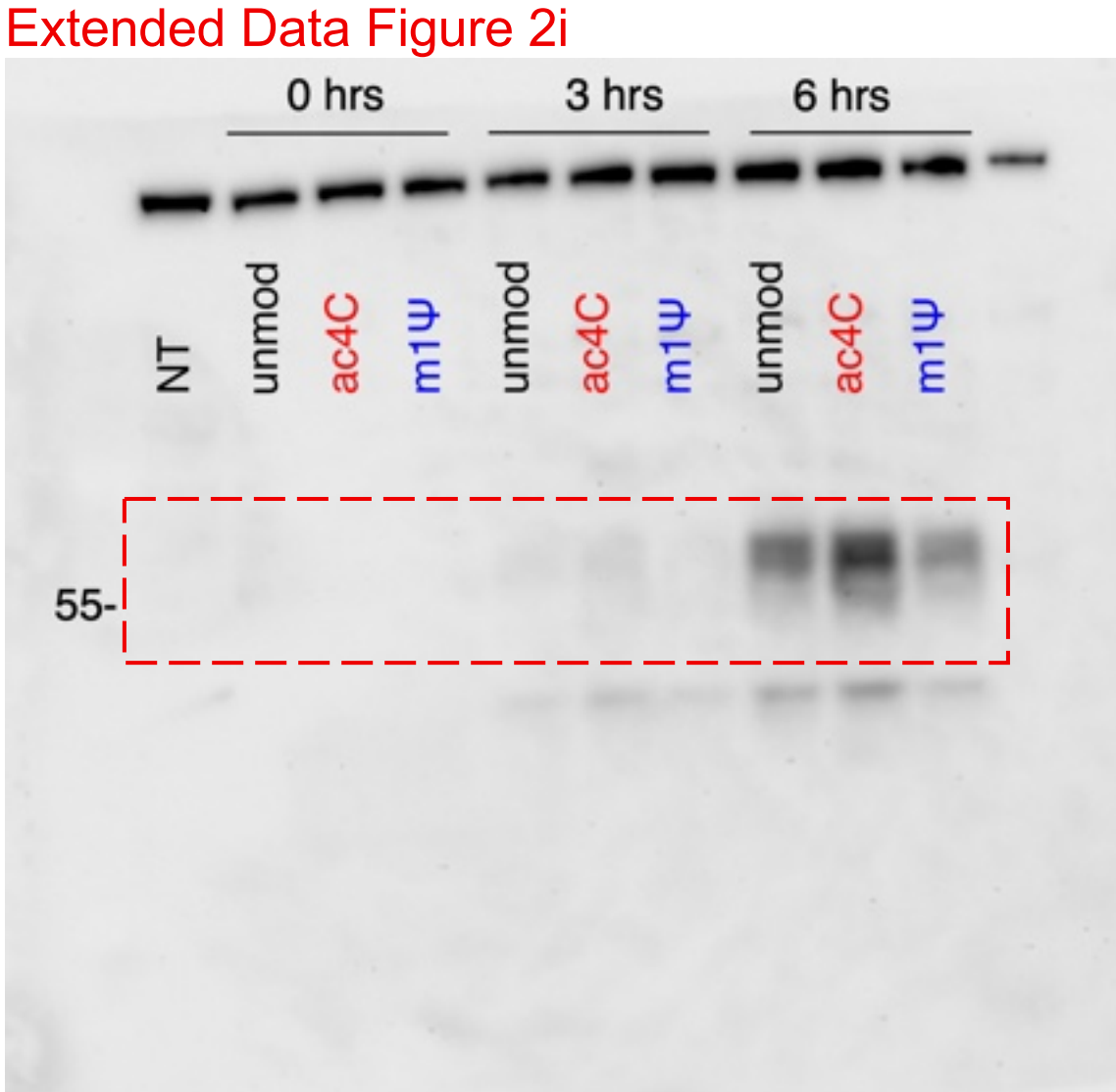

Extended Data Figure 2i

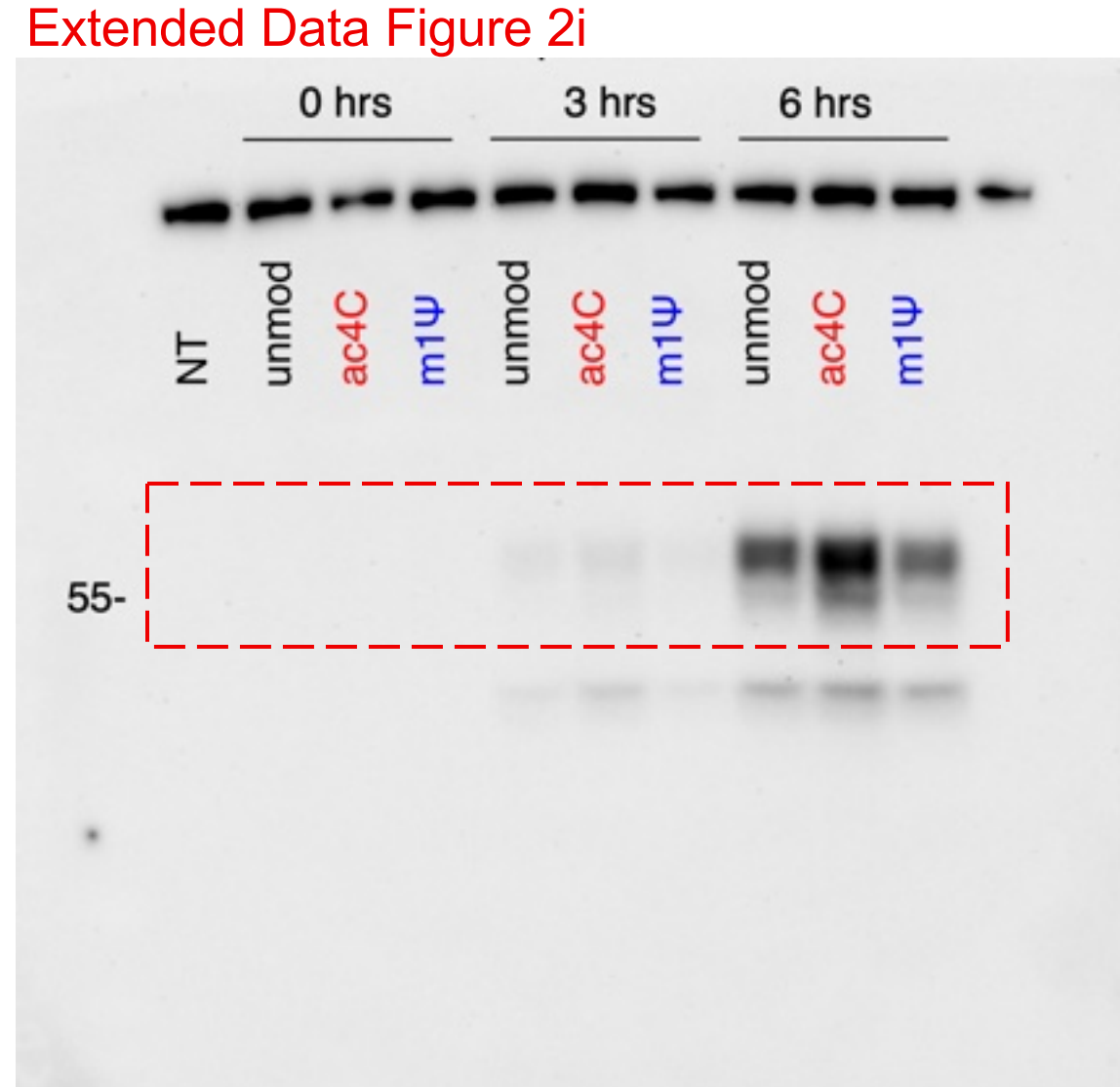

Extended Data Figure 2i

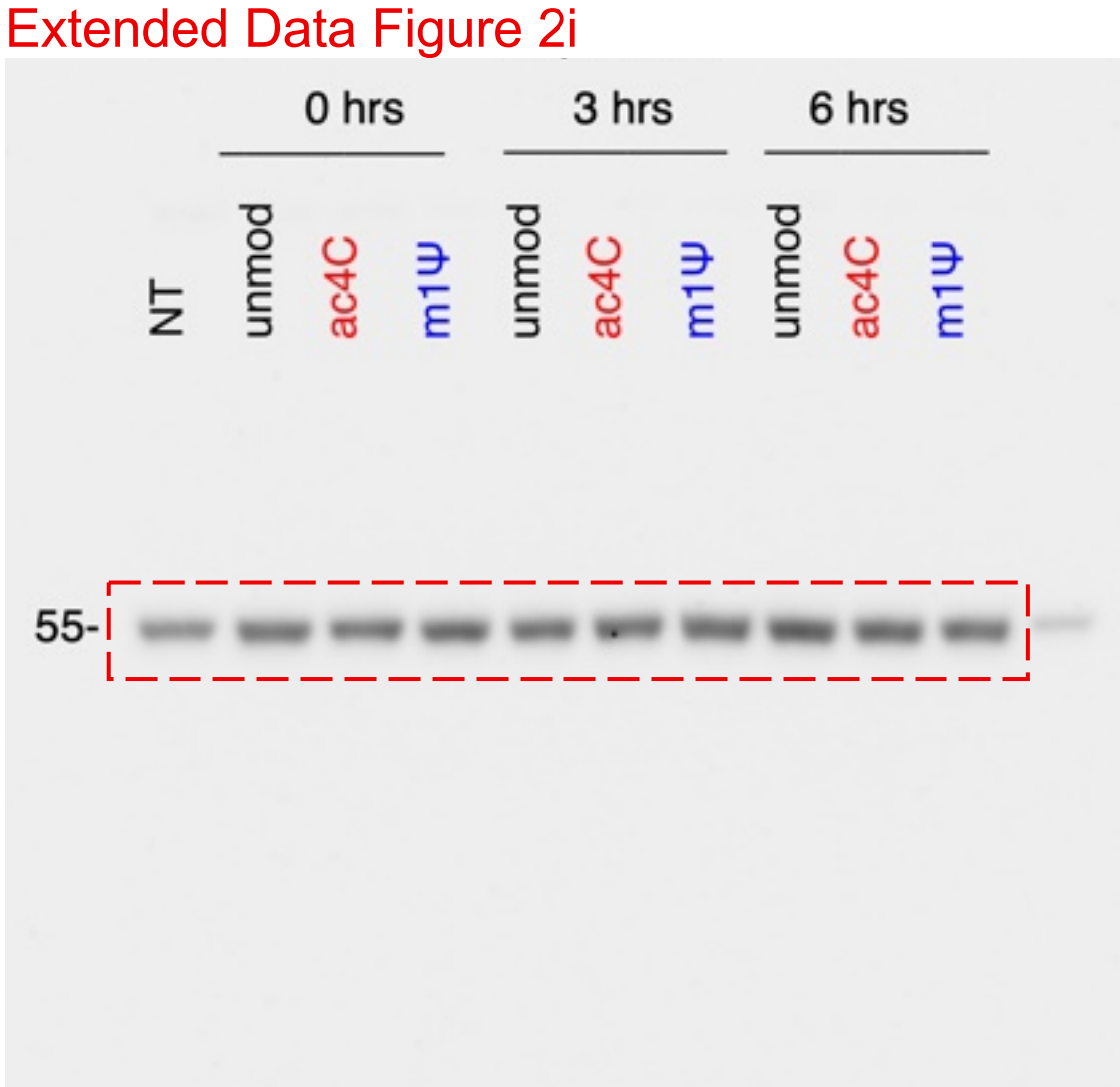

Extended Data Figure 2i

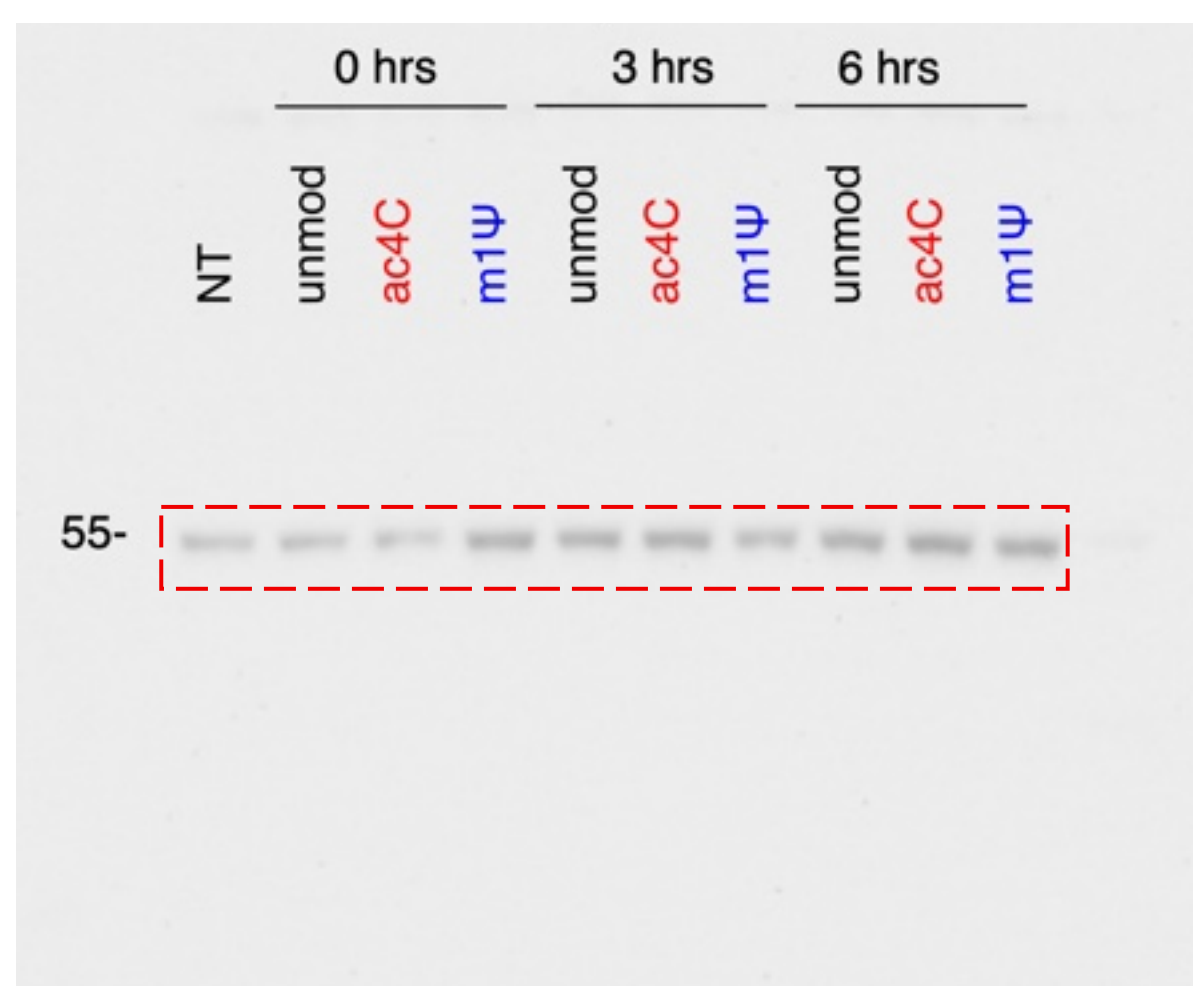

Fig.2b THP-1 NanoLuc Extended Data Figure 3d

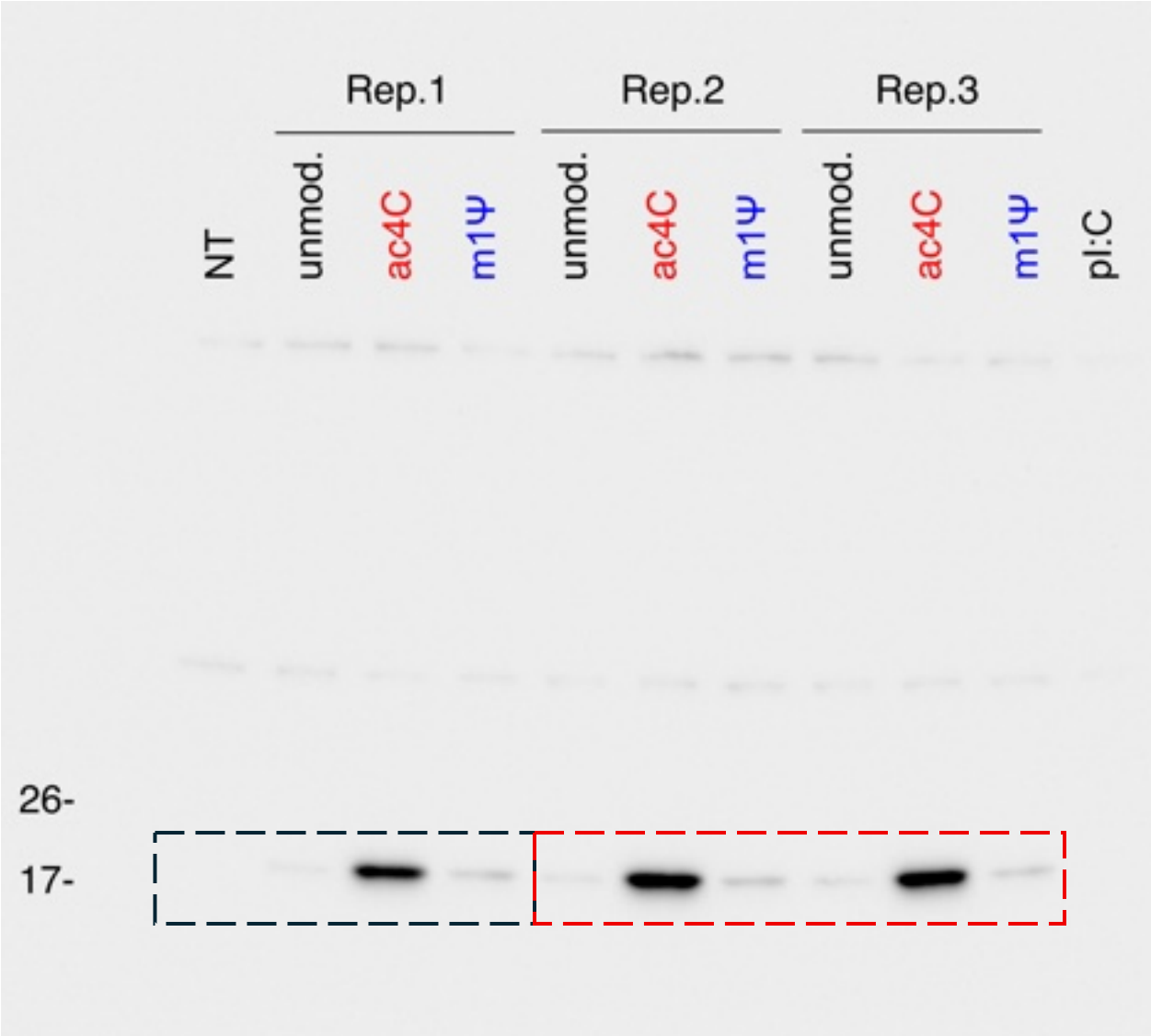

Fig.2b THP-1 p-eIF2-α Extended Data Figure 3d

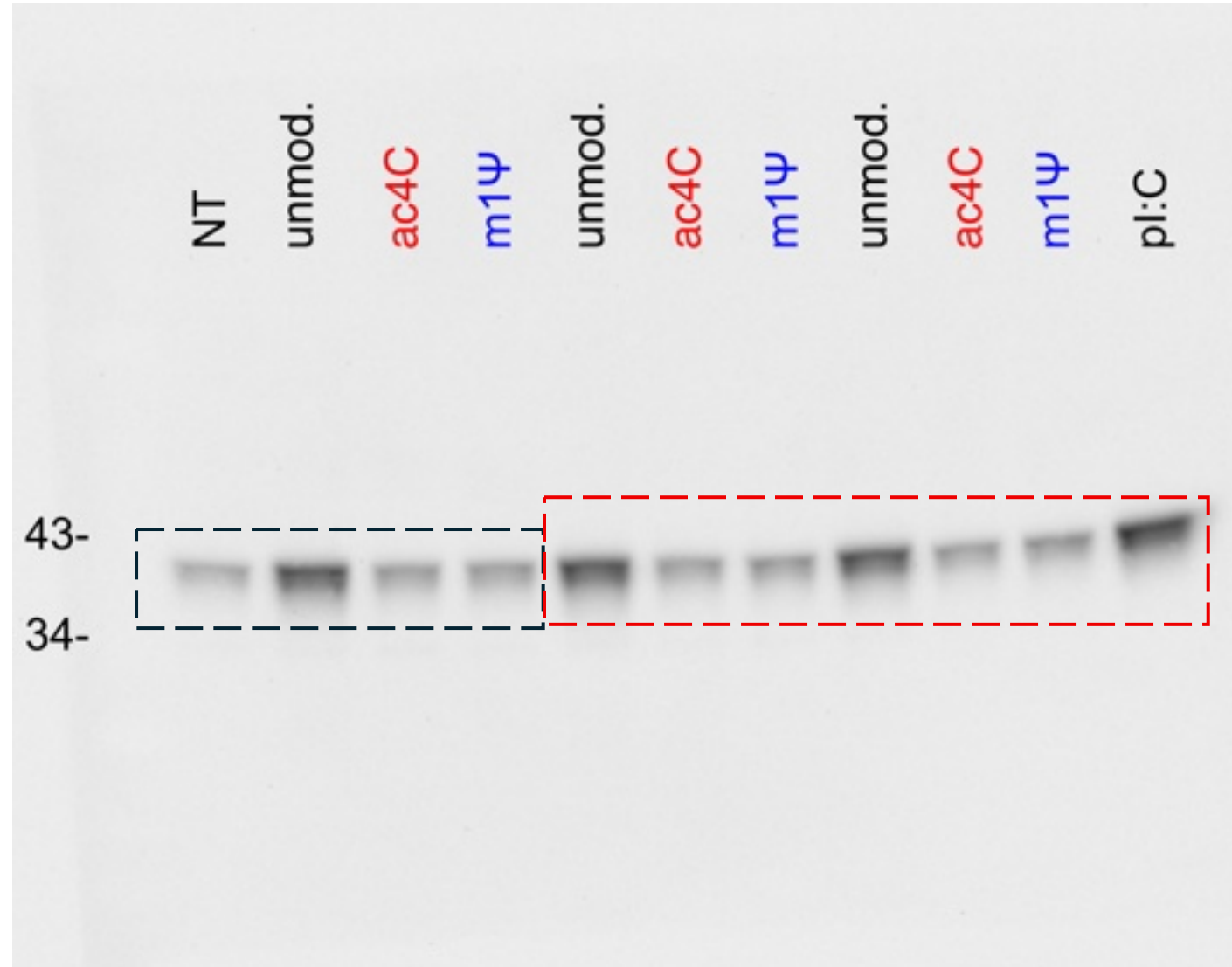

Fig.2b THP-1 eIF2-α Extended Data Figure 3d

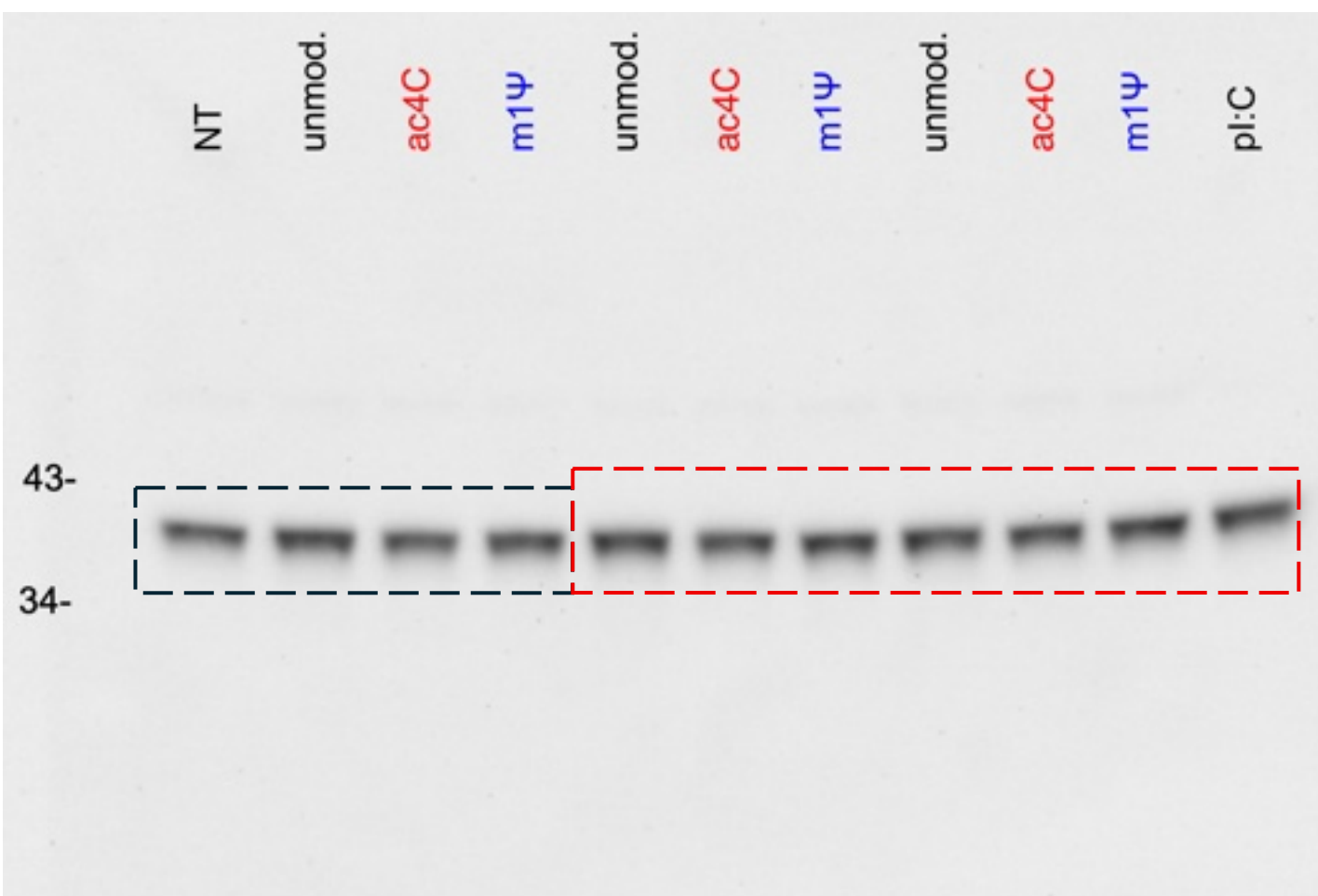

Fig.2b THP-1 TNF-α Extended Data Figure 3d

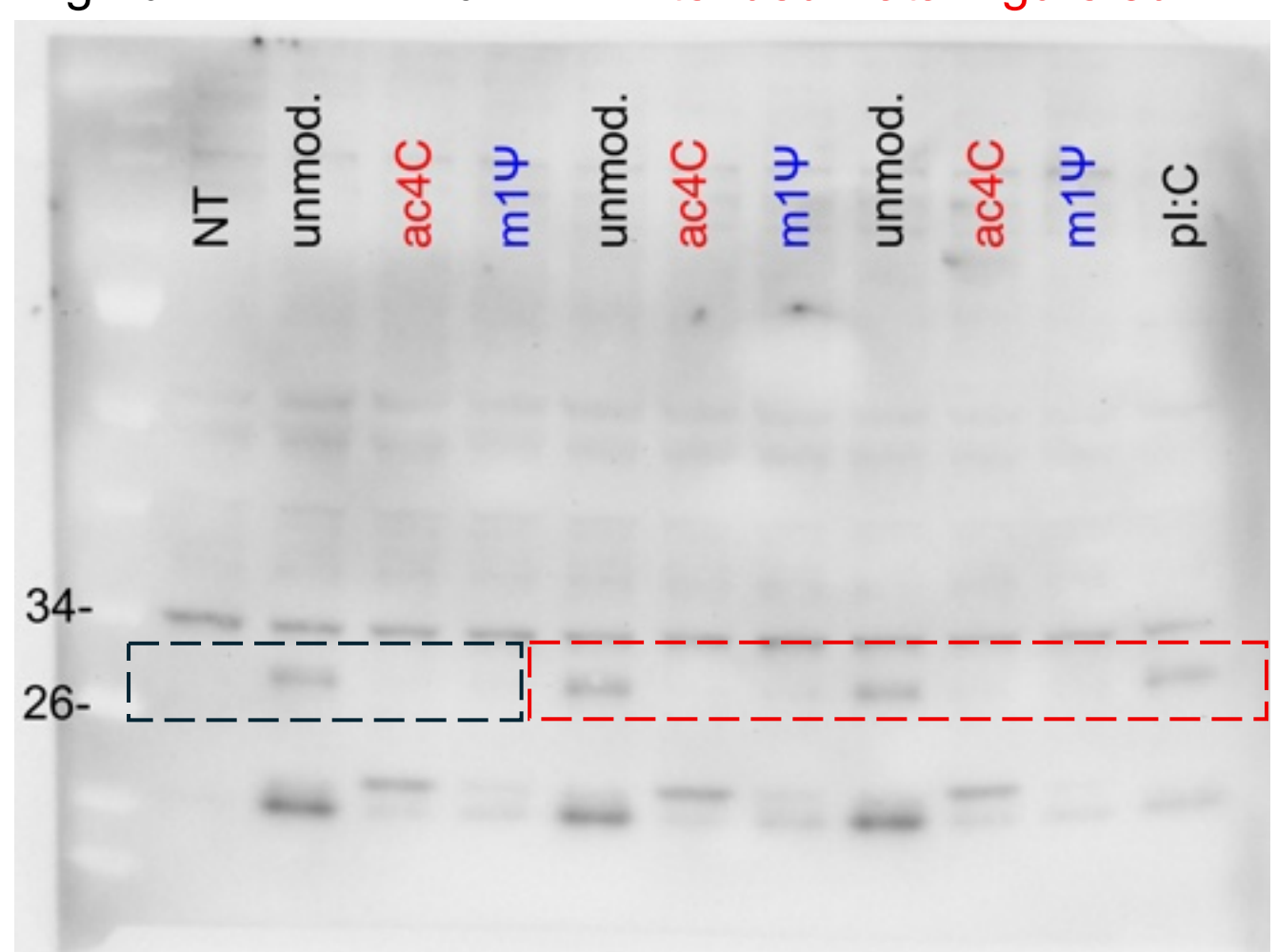

Fig.2b THP-1 β-tubulin Extended Data 3d

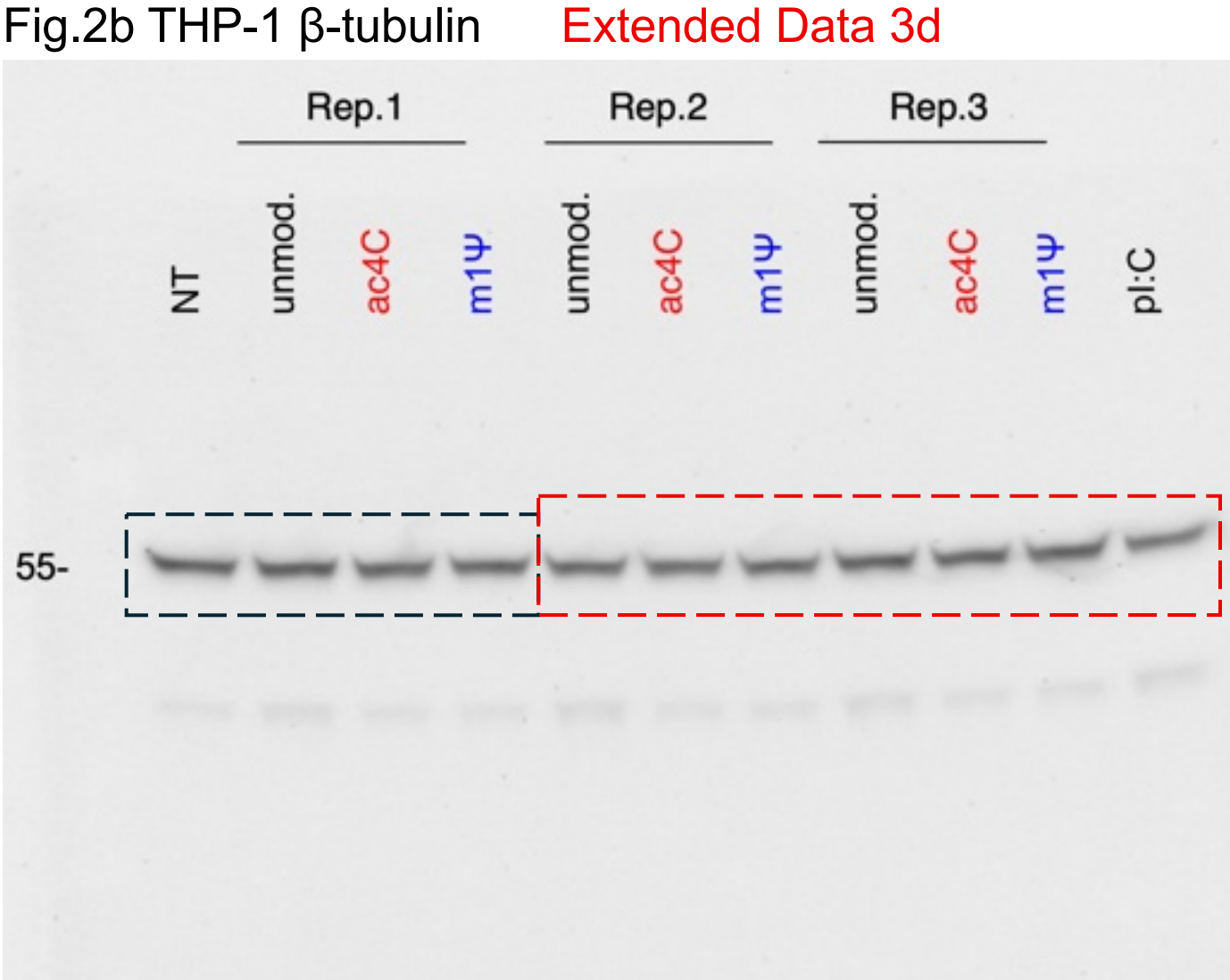

Fig.2e NanoLuc

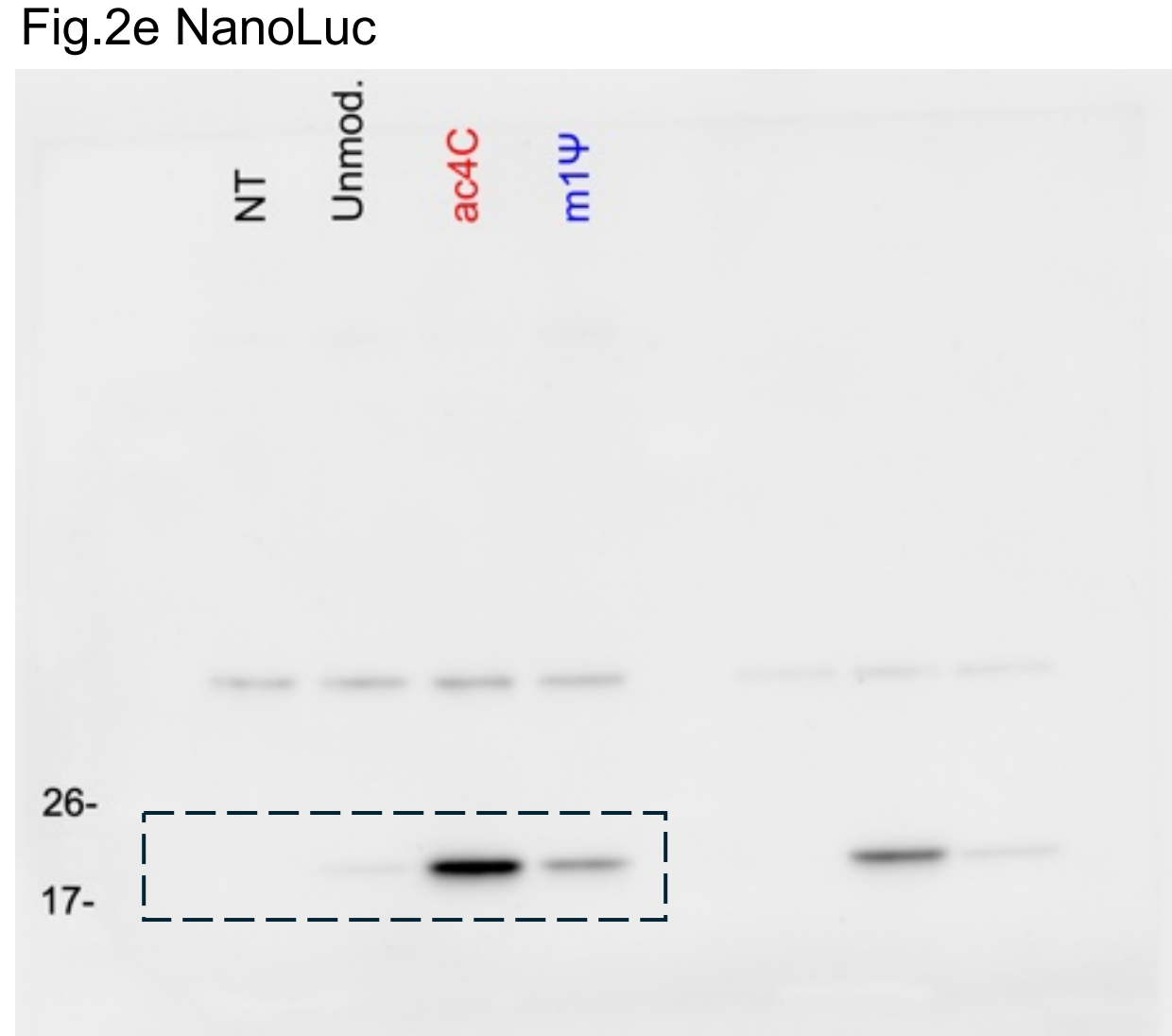

Fig.2e NanoLuc

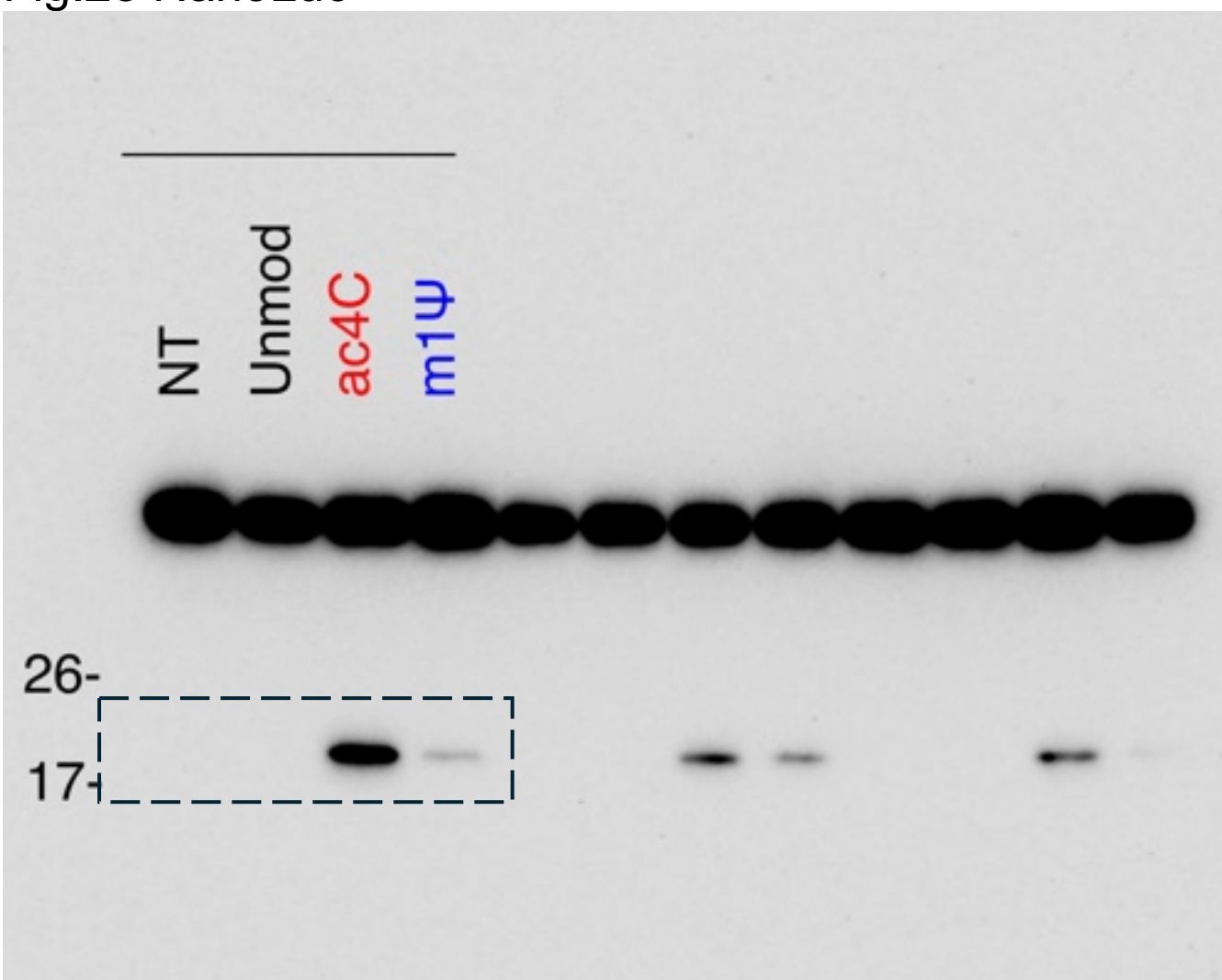

Fig.2e NanoLuc

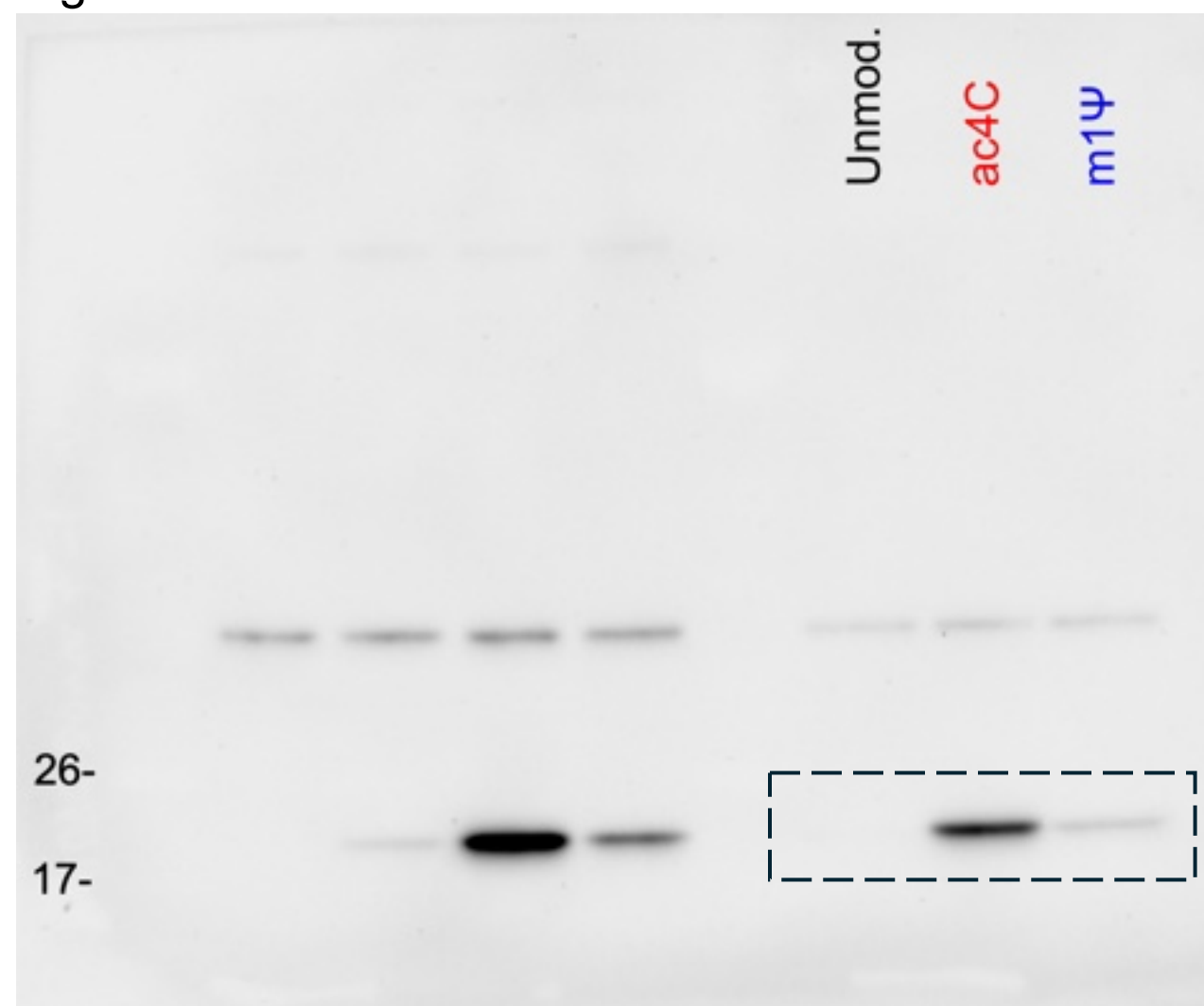

Fig.2e NanoLuc

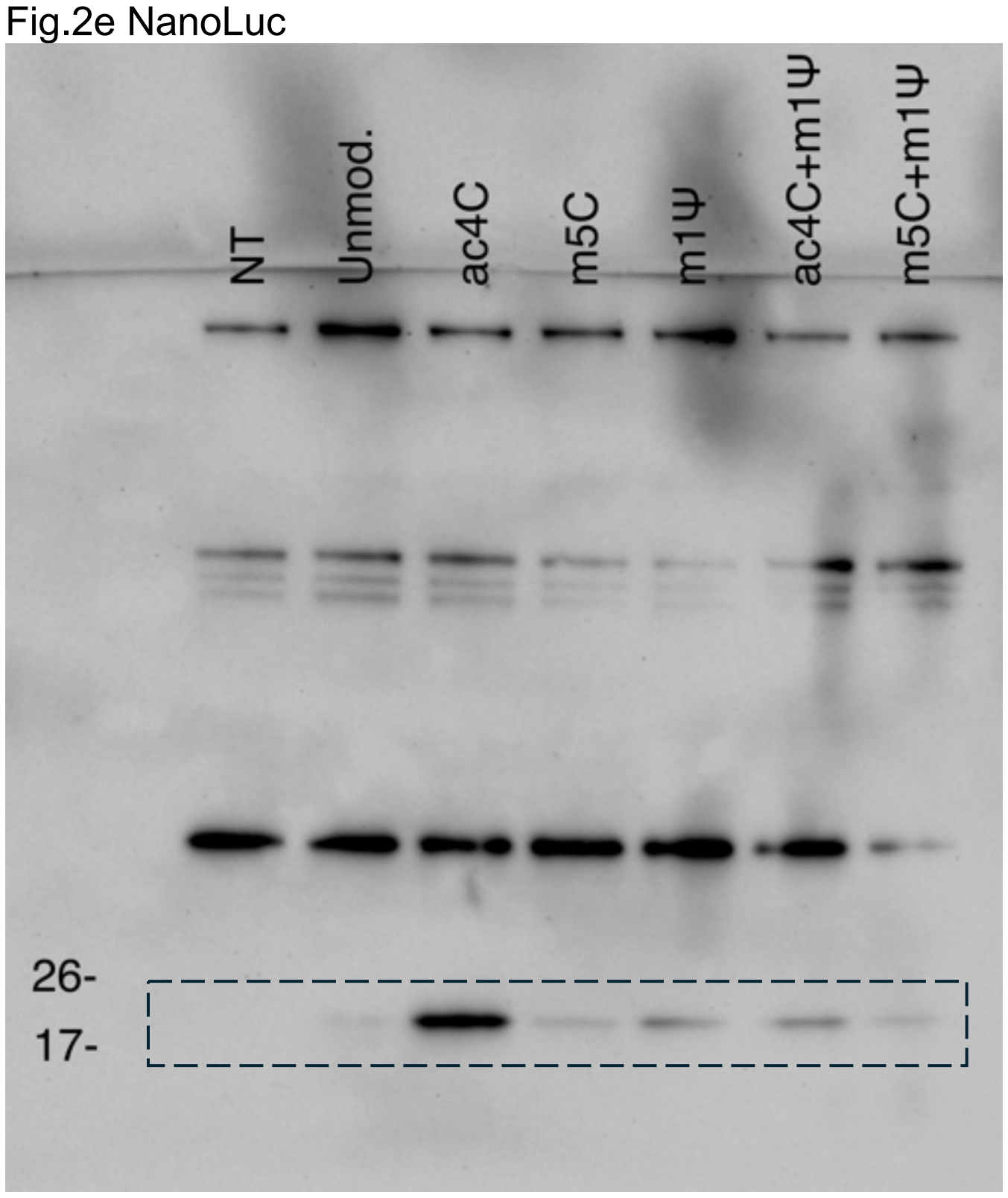

Fig.2e p-eIF2-α and β-tubulin

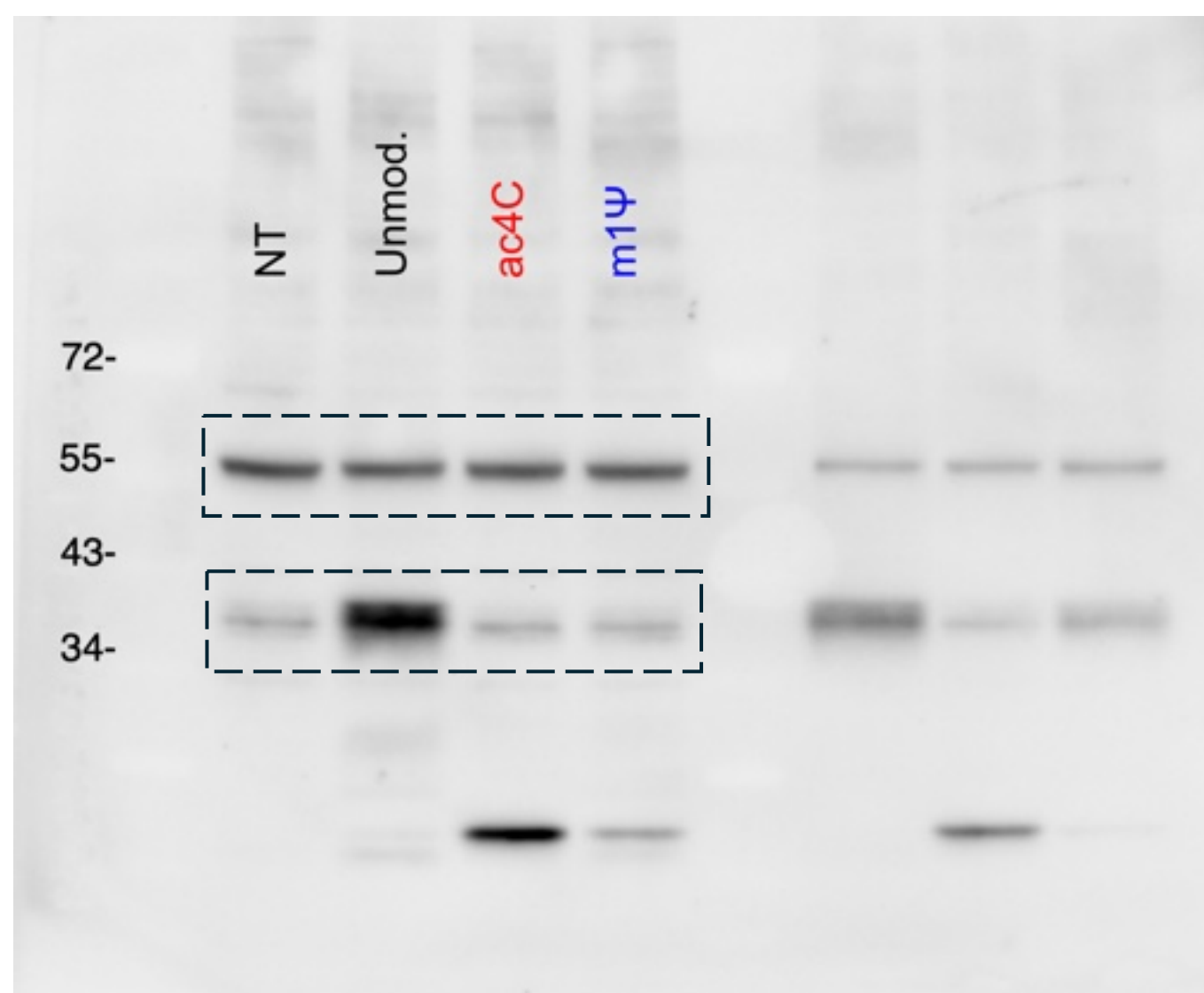

Fig.2e p-eIF2-α

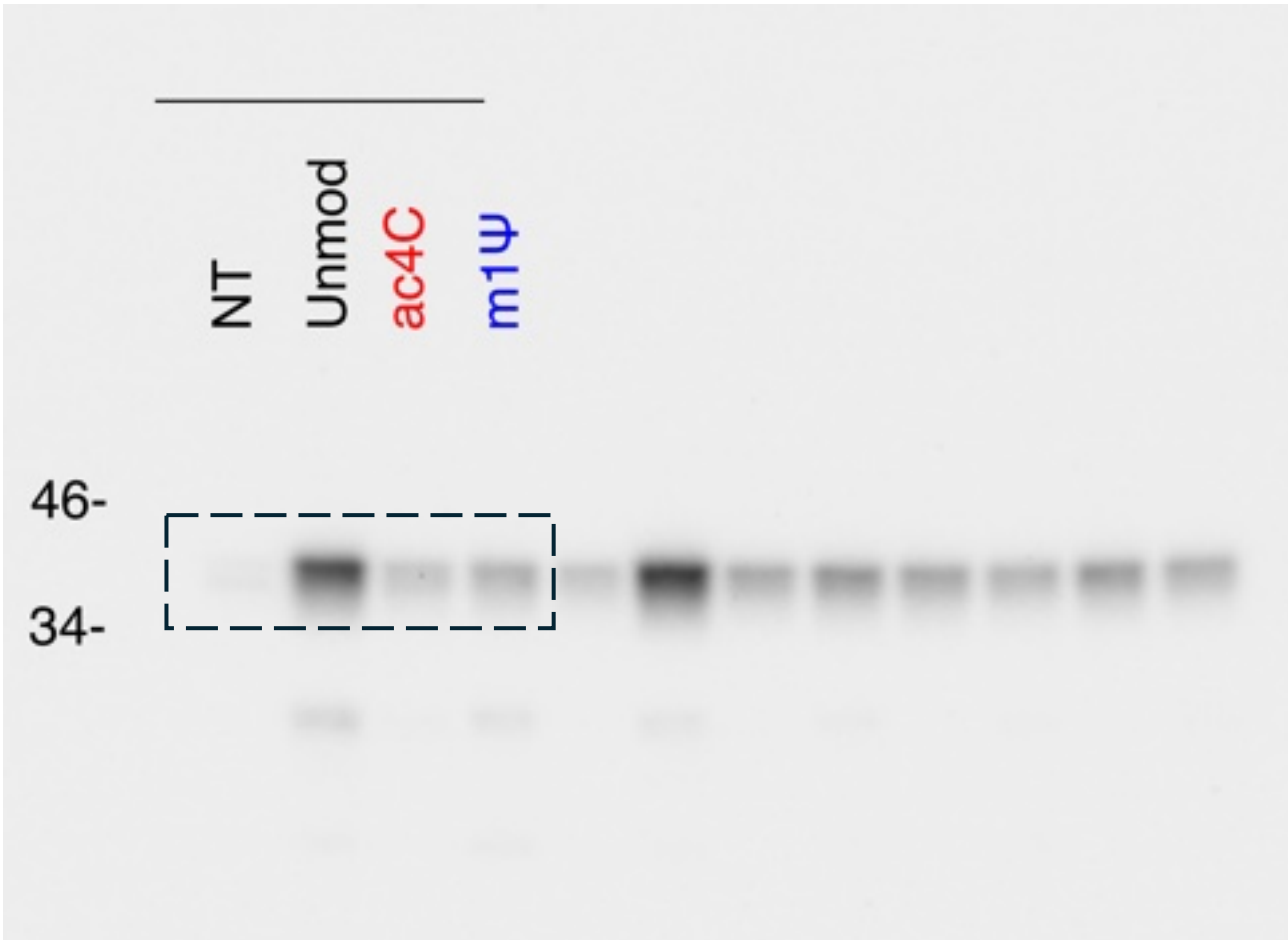

Fig.2e p-eIF2-α and β-tubulin

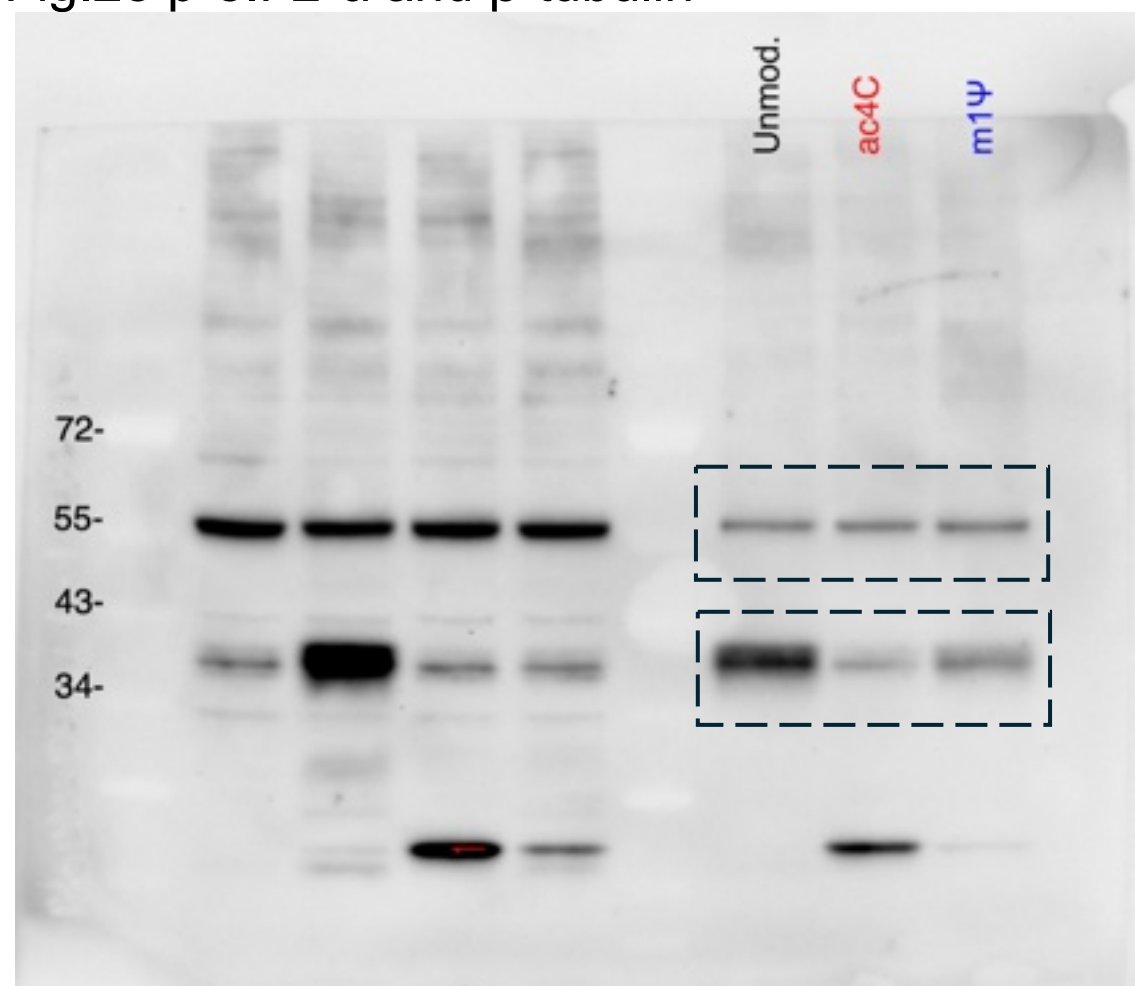

Fig.2e p-eIF2-α

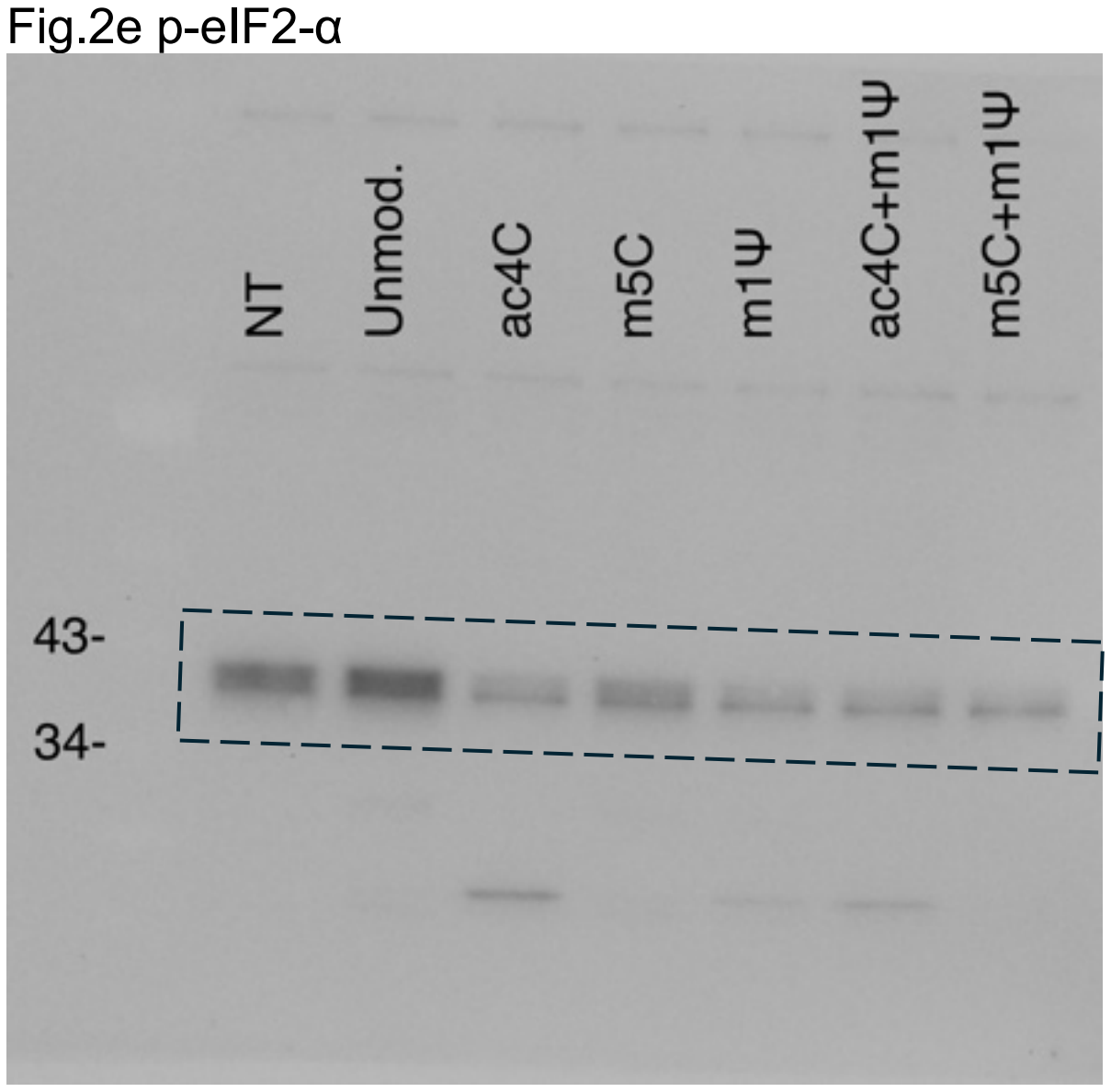

Fig.2e β-tubulin

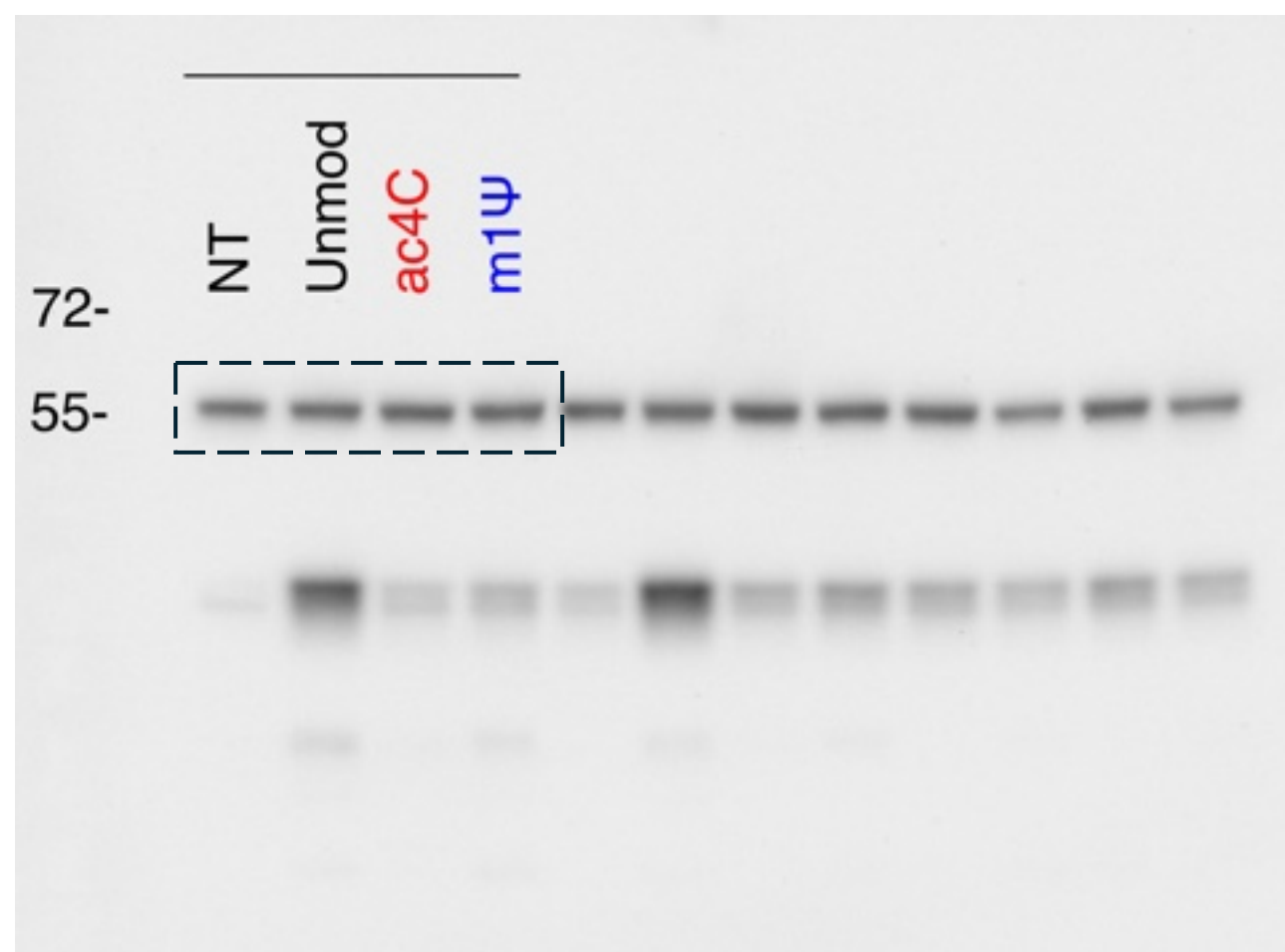

Fig.2e β-tubulin

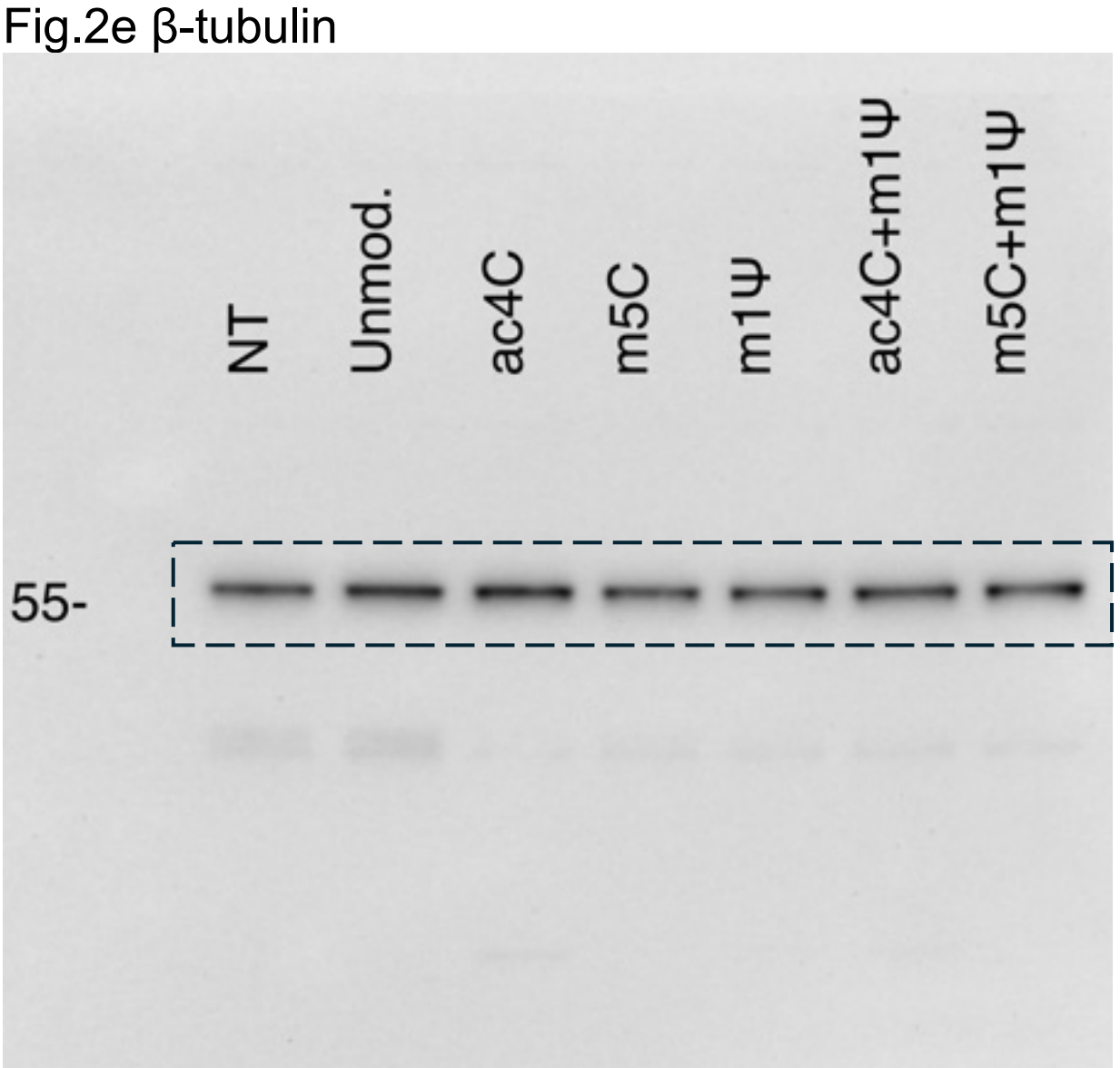

Fig.2e eIF2-α

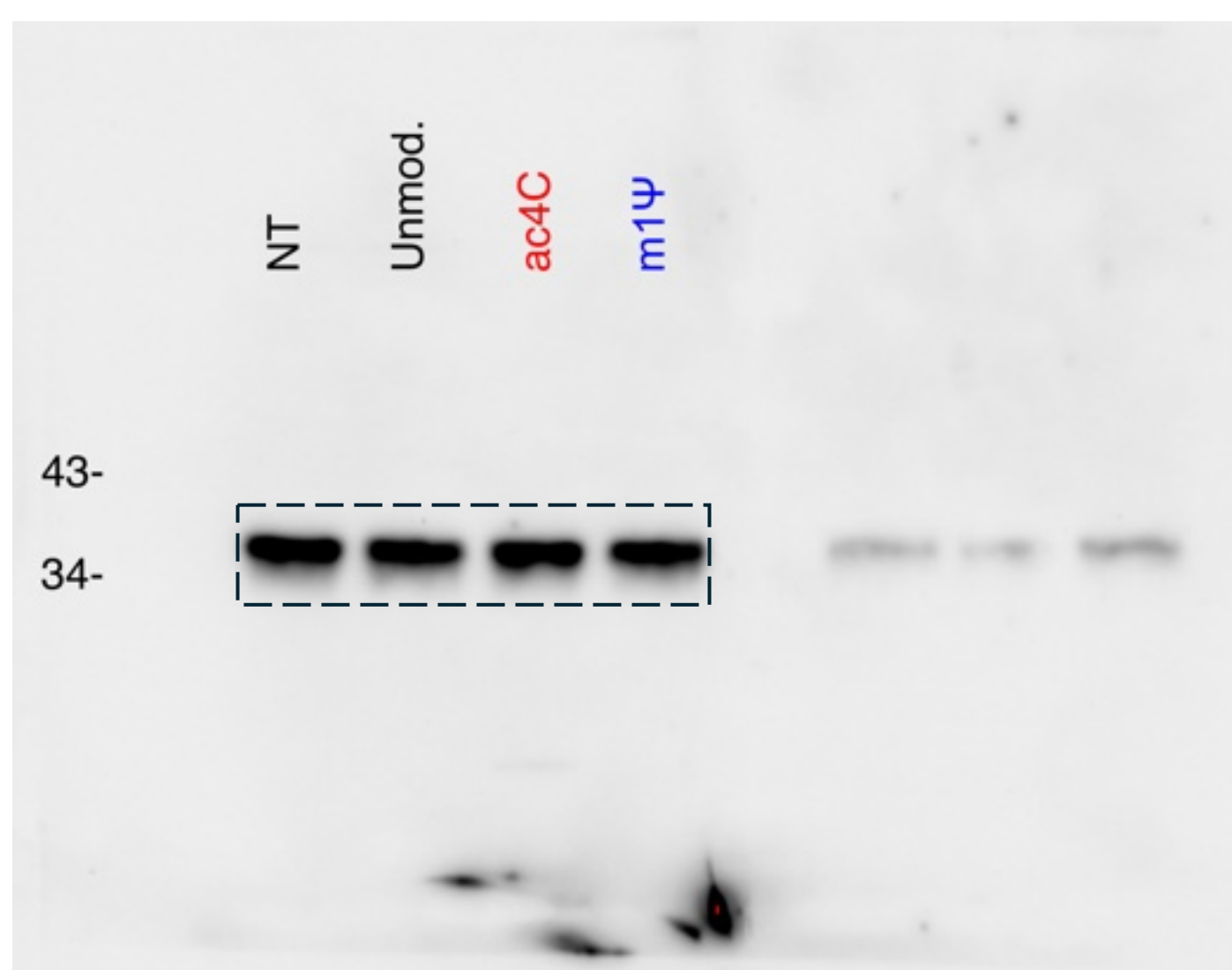

Fig.2e eIF2-α

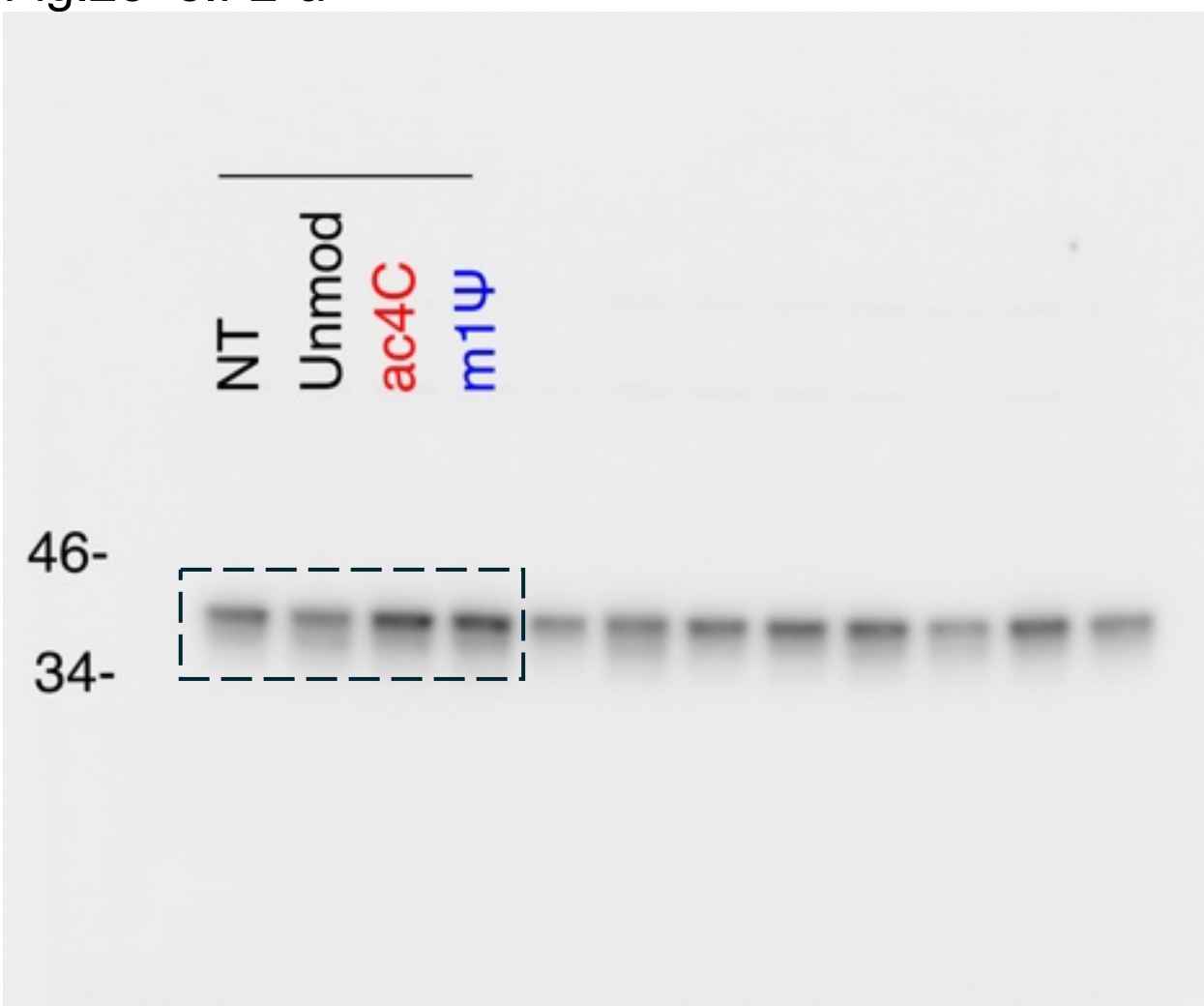

Fig.2e eIF2-α

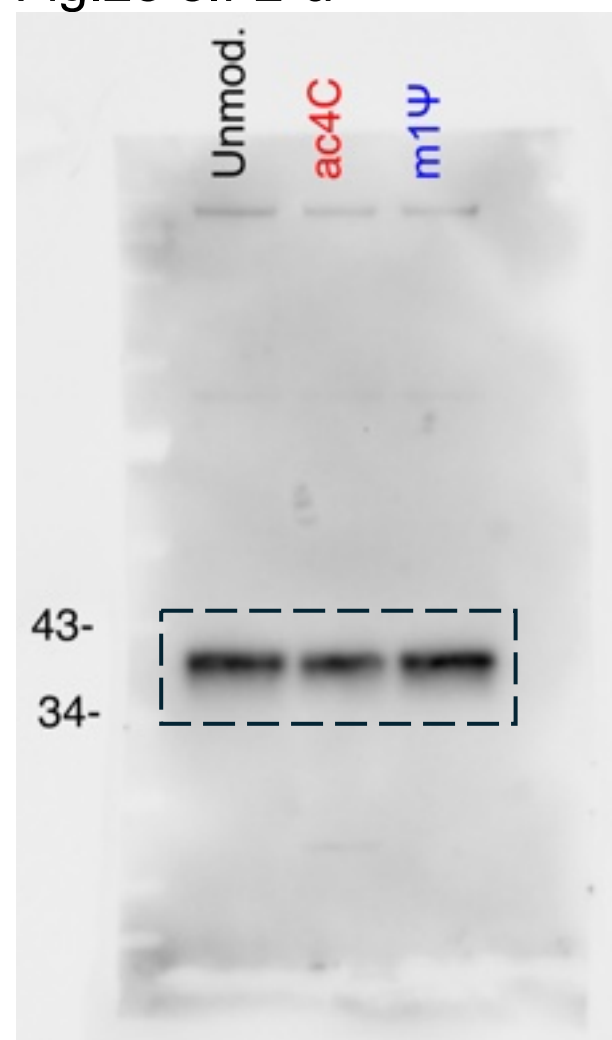

Fig.2e eIF2-α

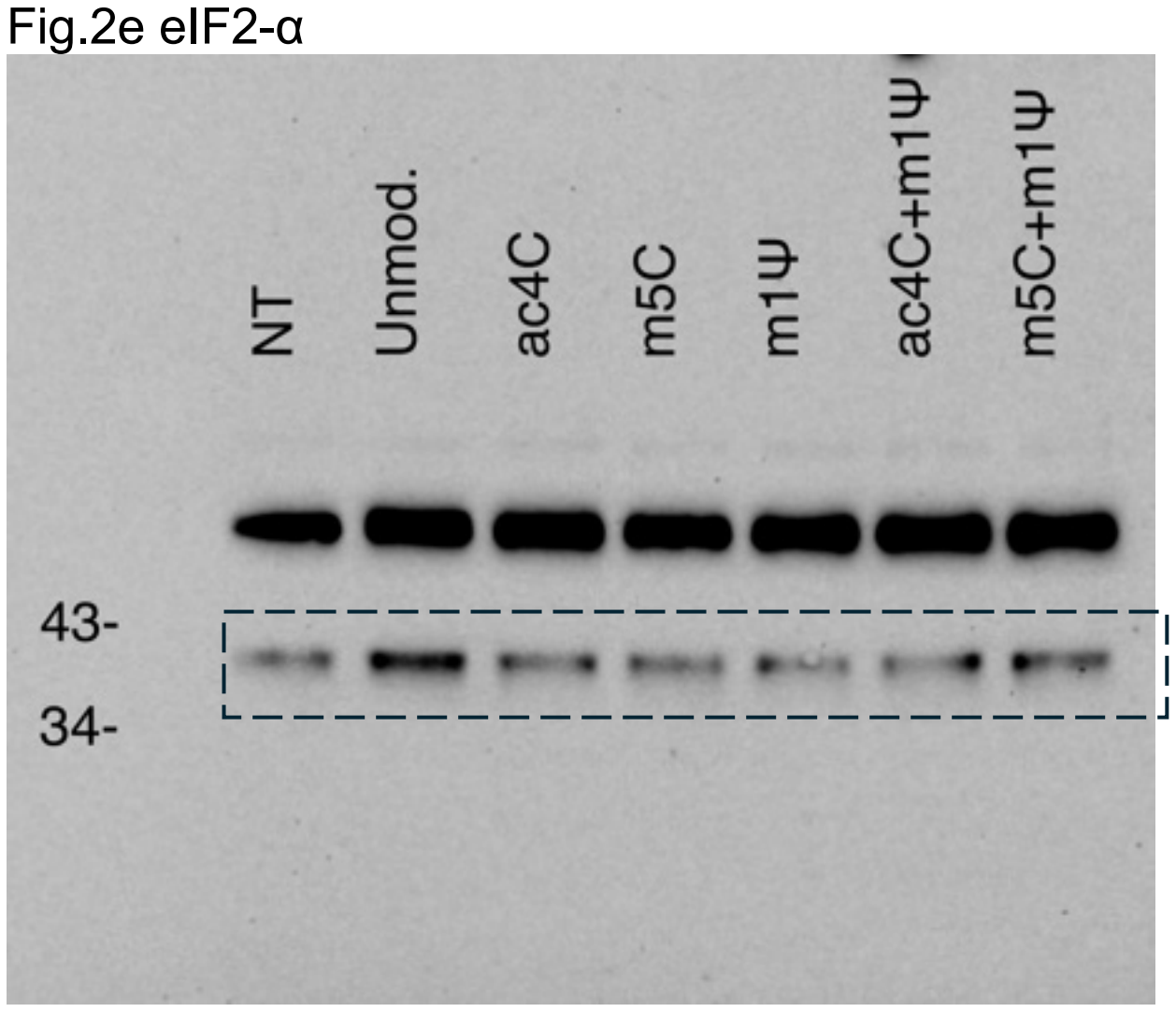

Fig.2e TNF-α

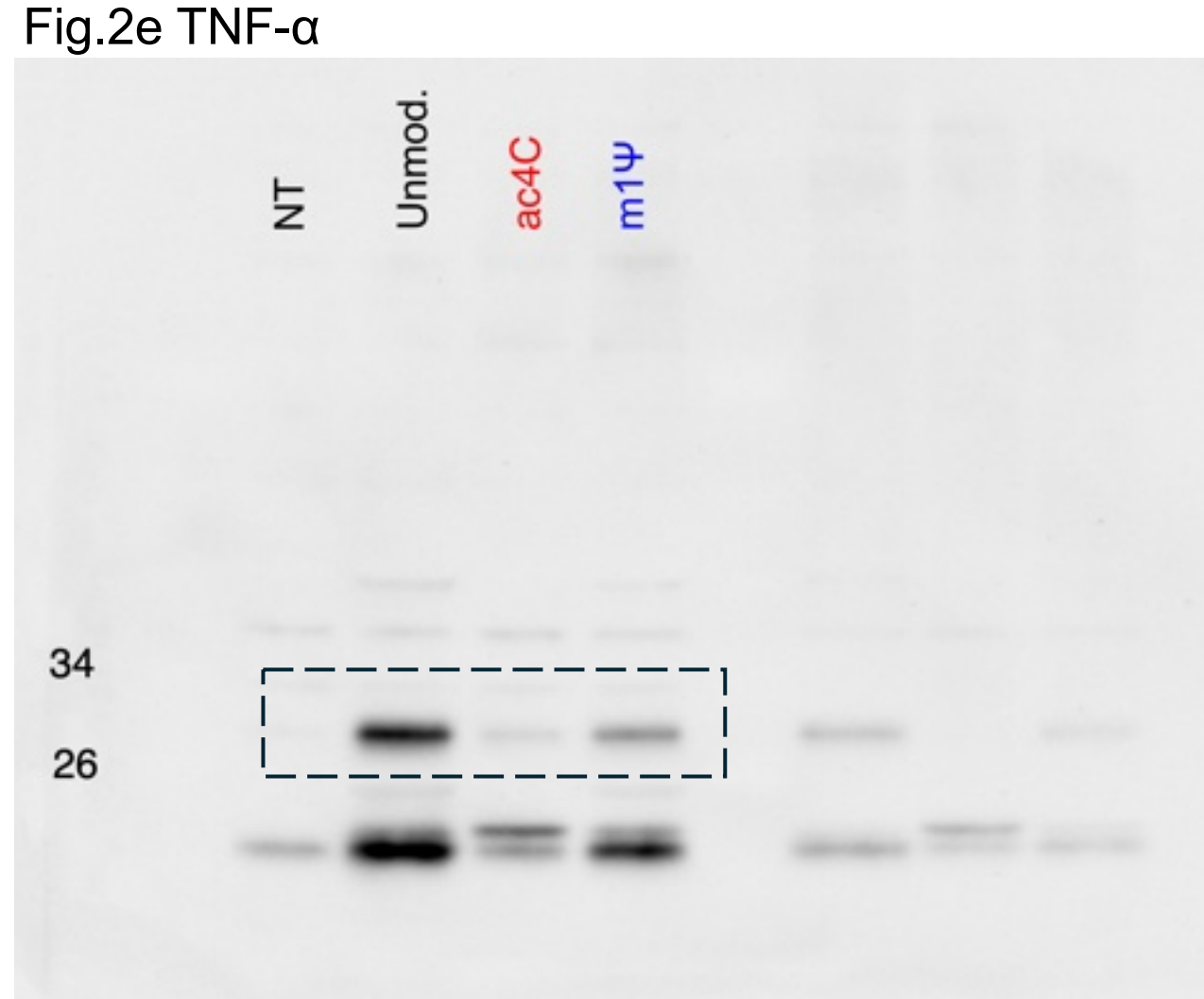

Fig.2e TNF-α

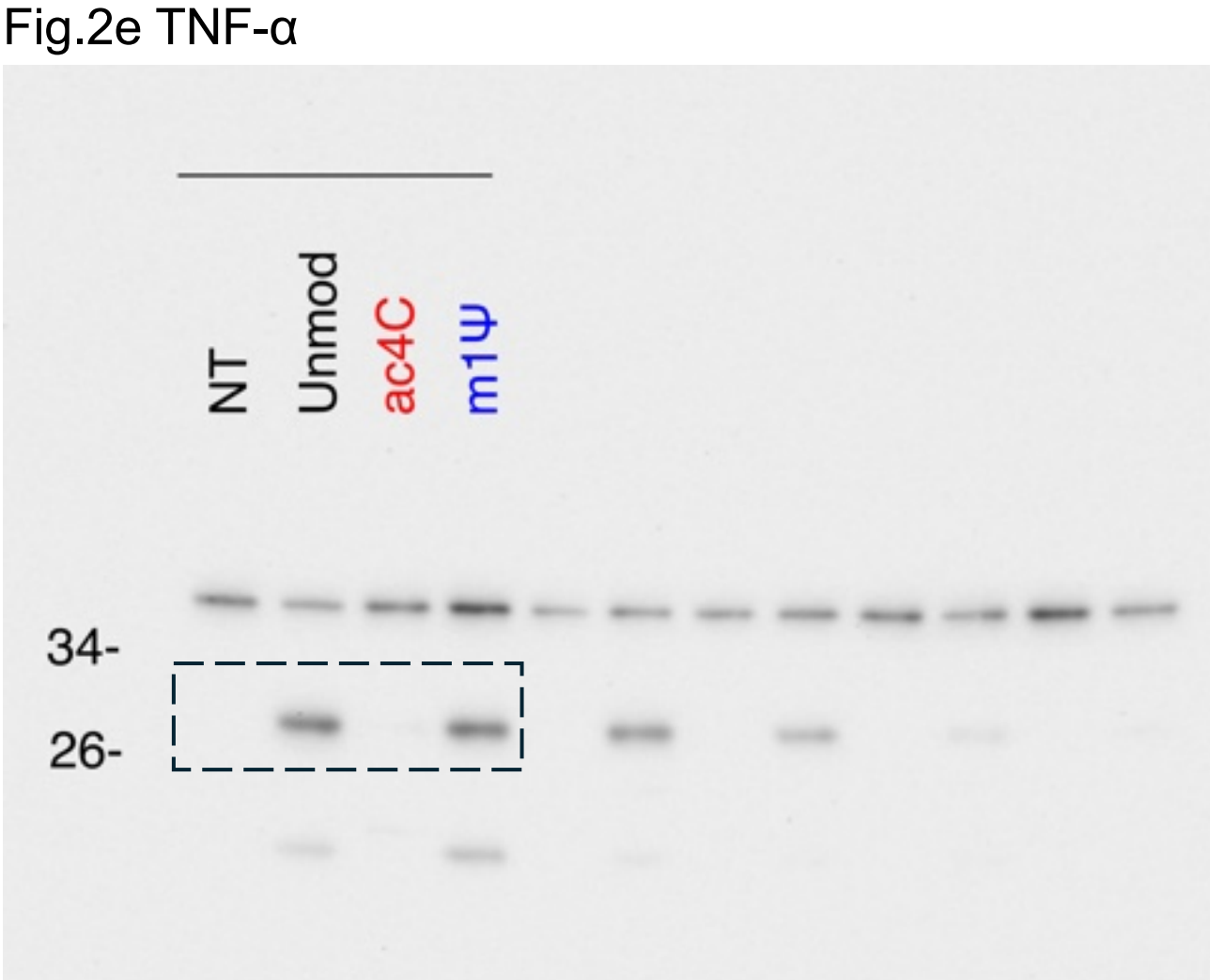

Fig.2e TNF-α

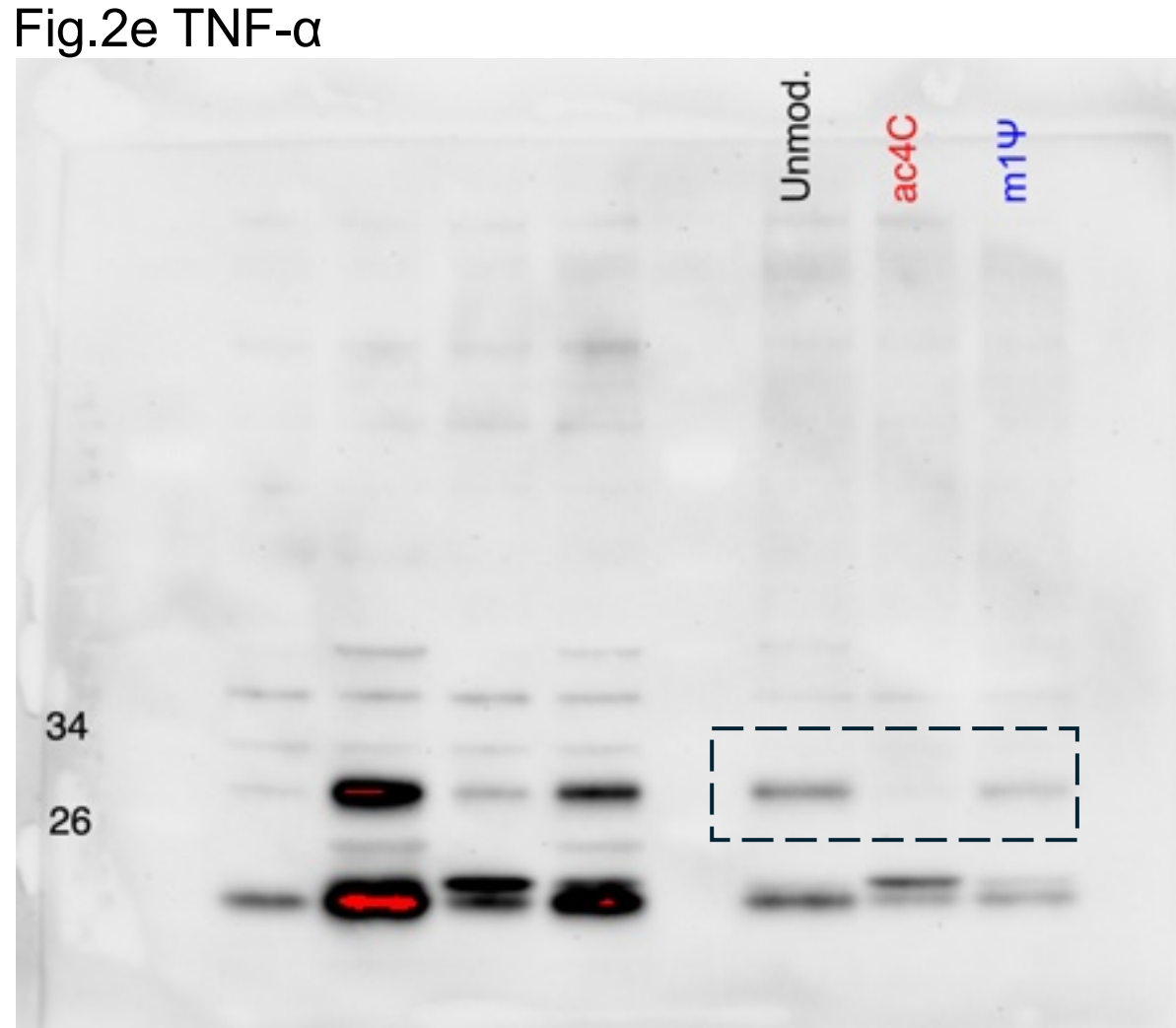

Fig.2e TNF-α

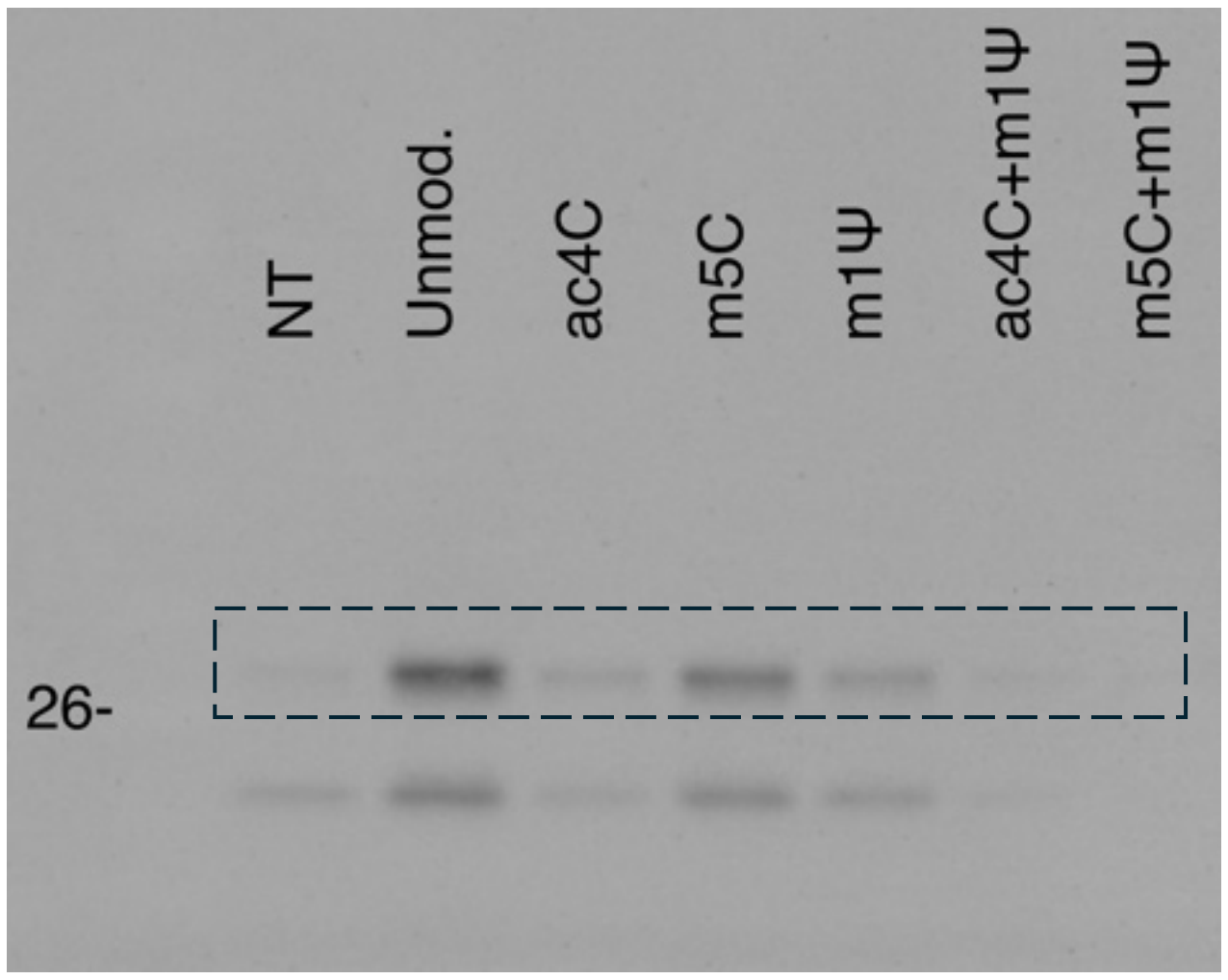

Fig.2i NanoLuc

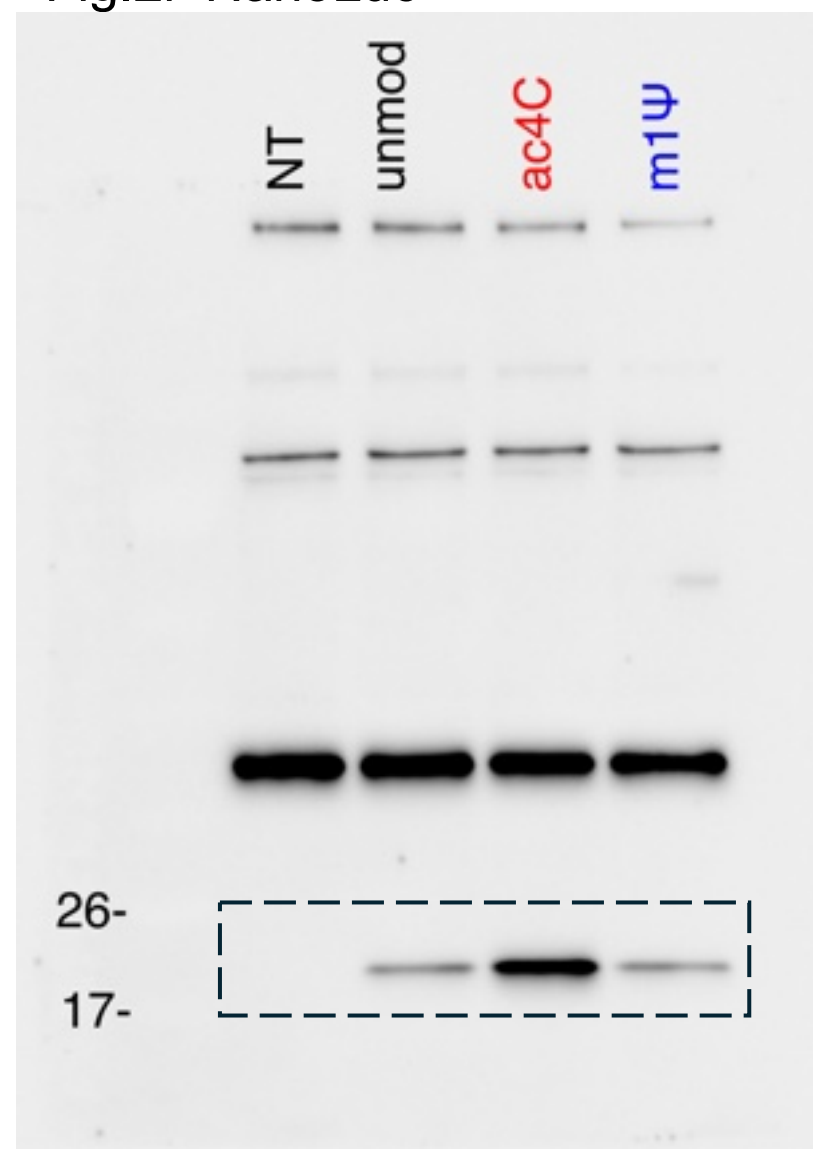

Fig.2i NanoLuc

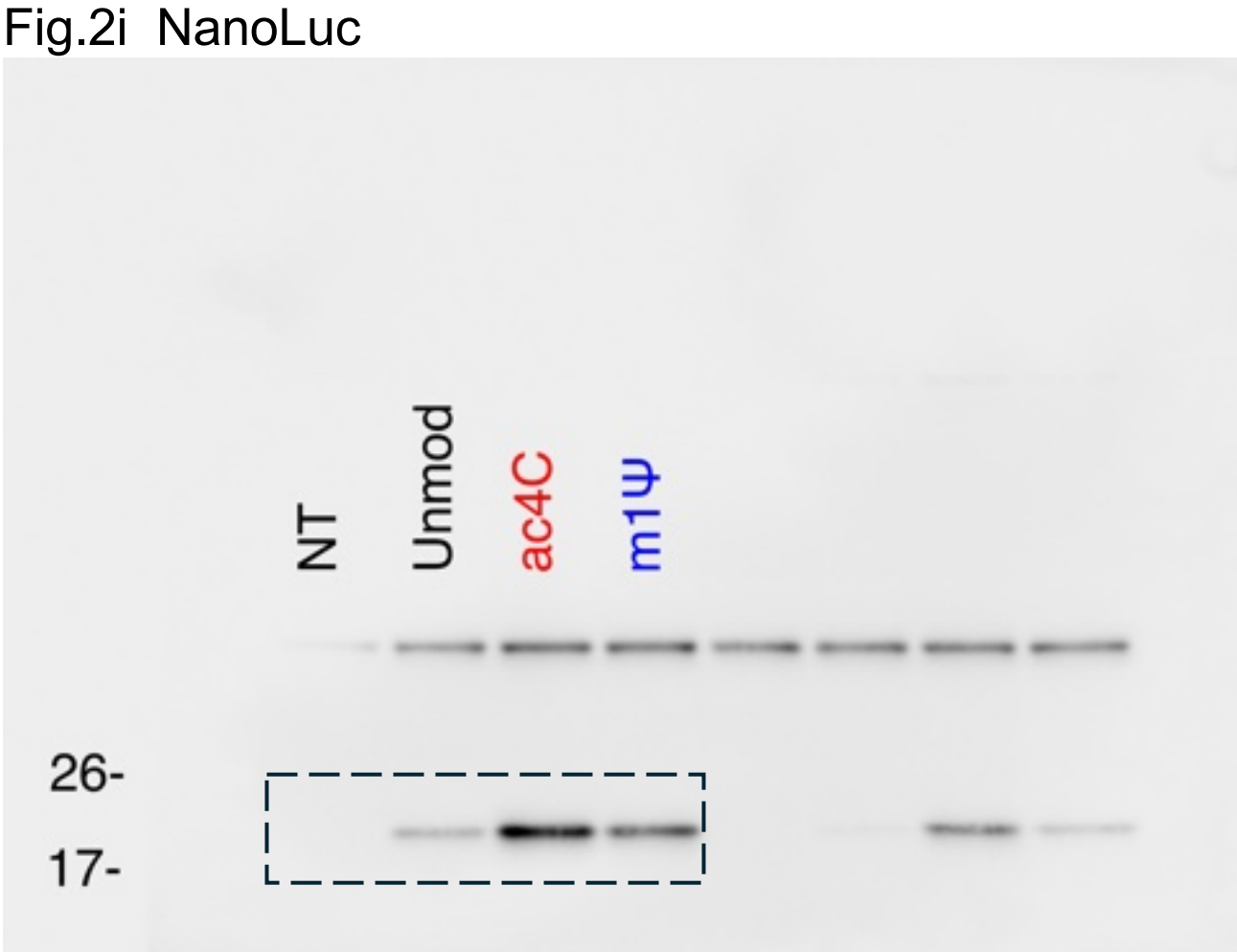

Fig.2i NanoLuc

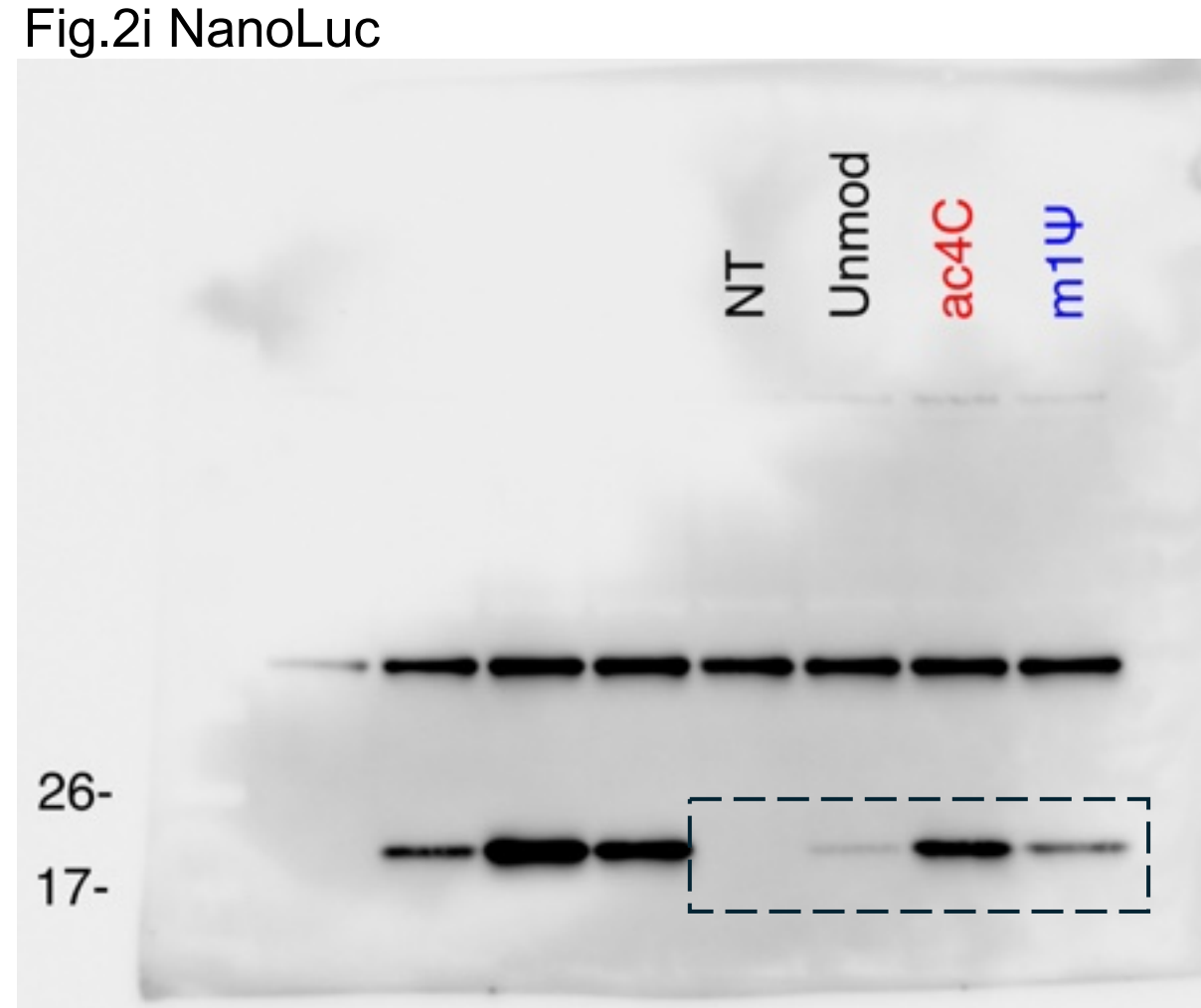

Fig.2i p-eIF2-α

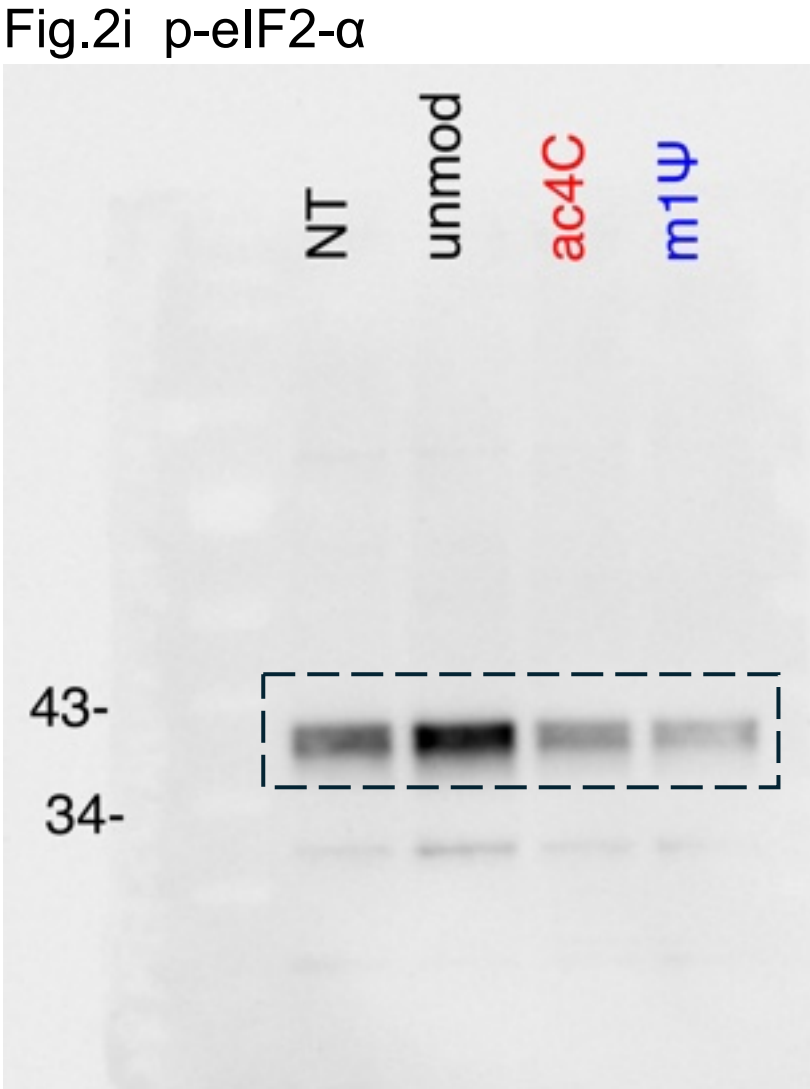

Fig.2i p-eIF2-α

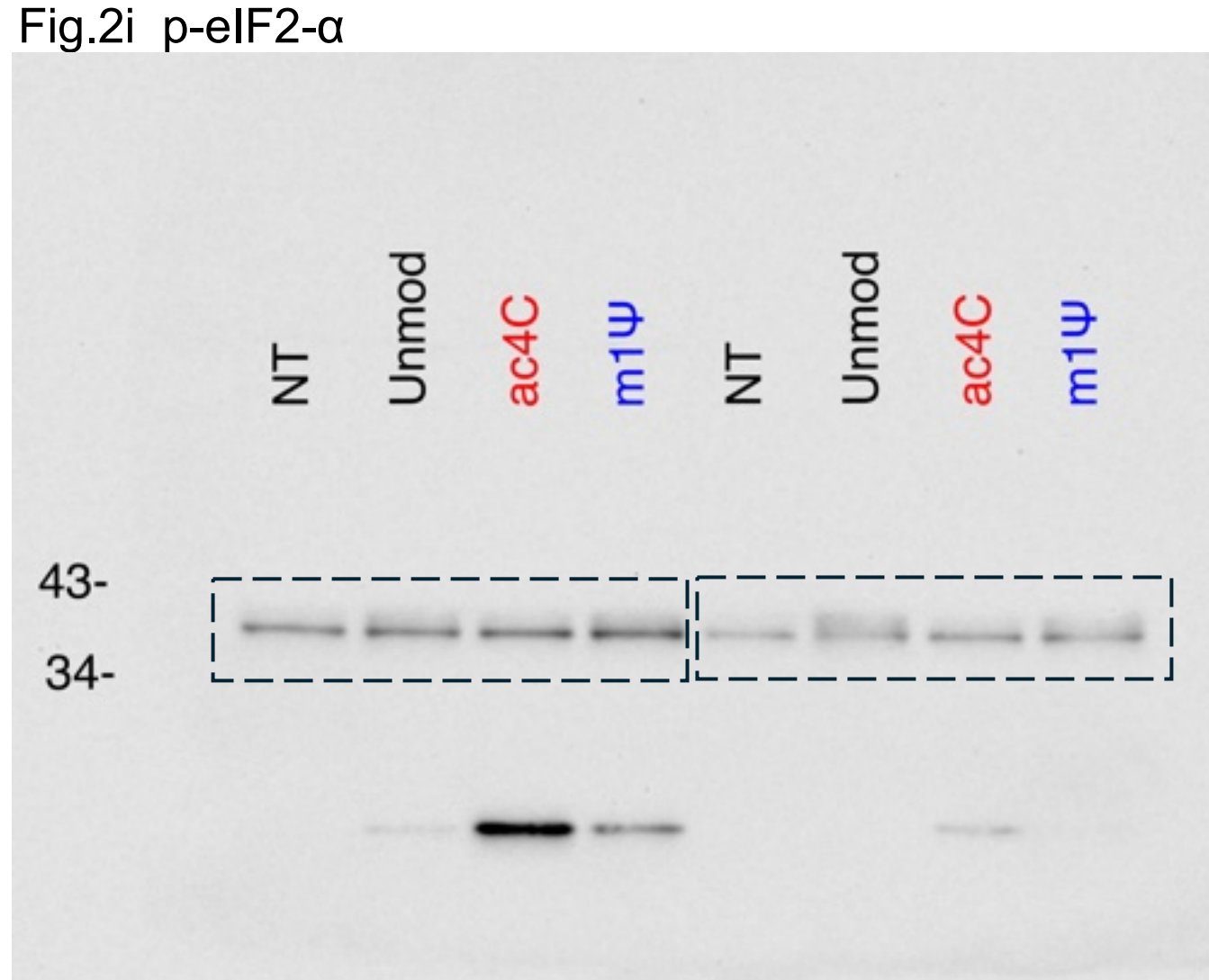

Fig.2i eIF2-α.

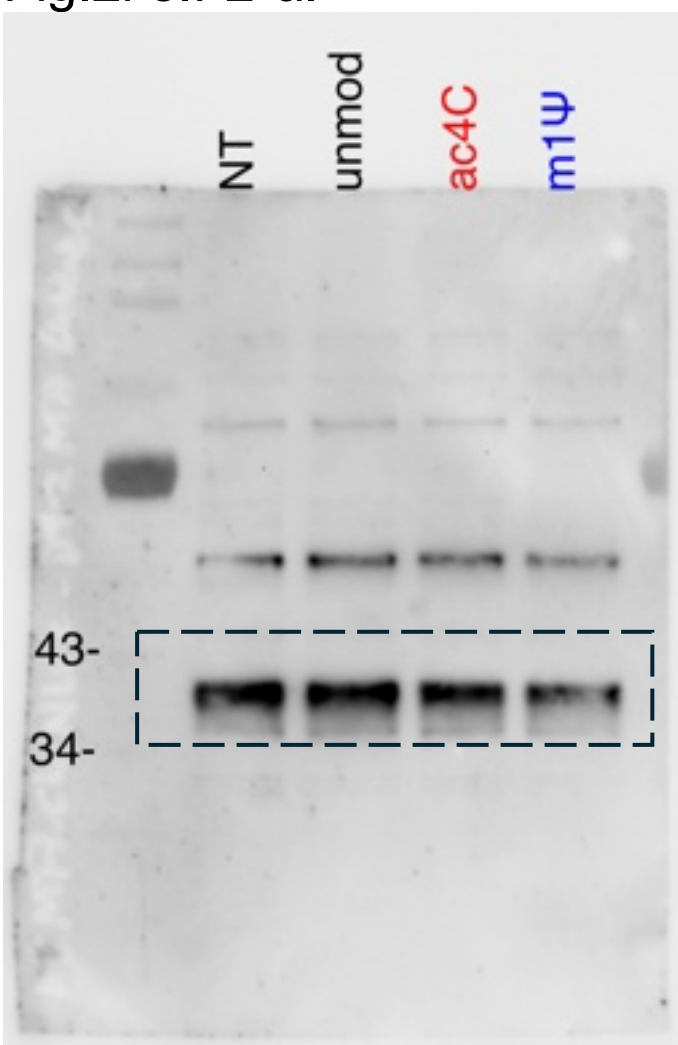

Fig.2i eIF2-α

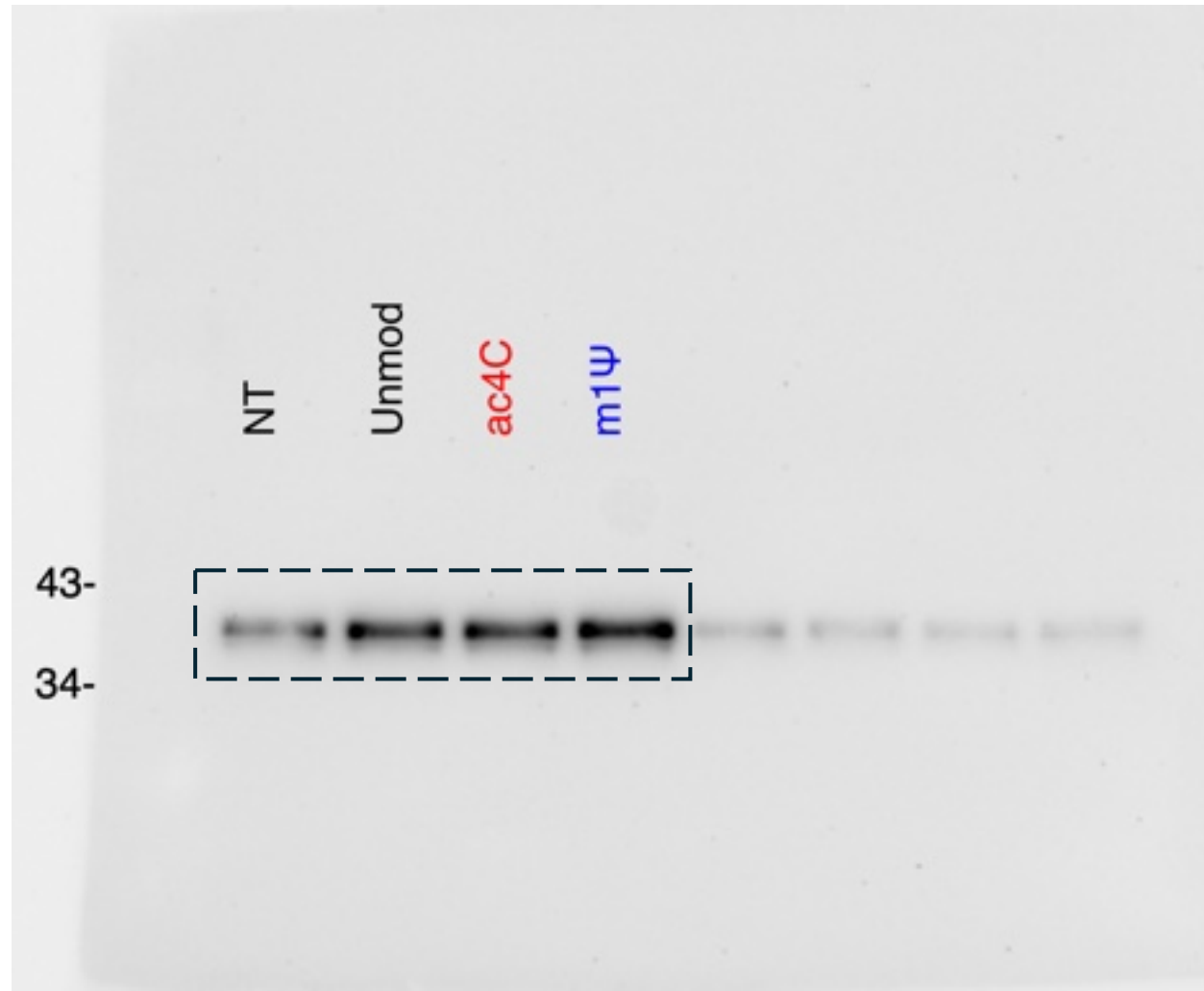

Fig.2i eIF2-α

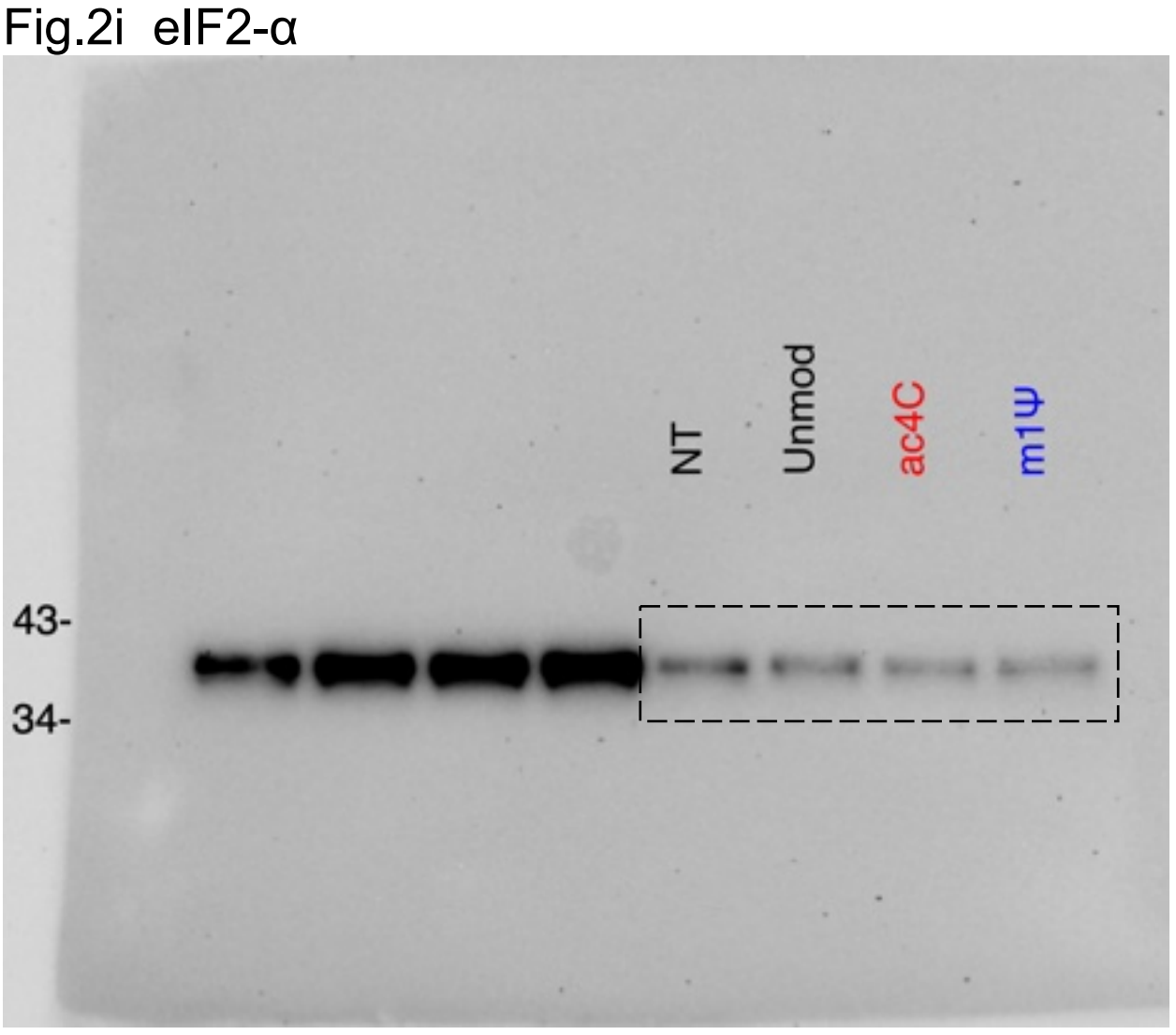

Fig.2i TNF-α

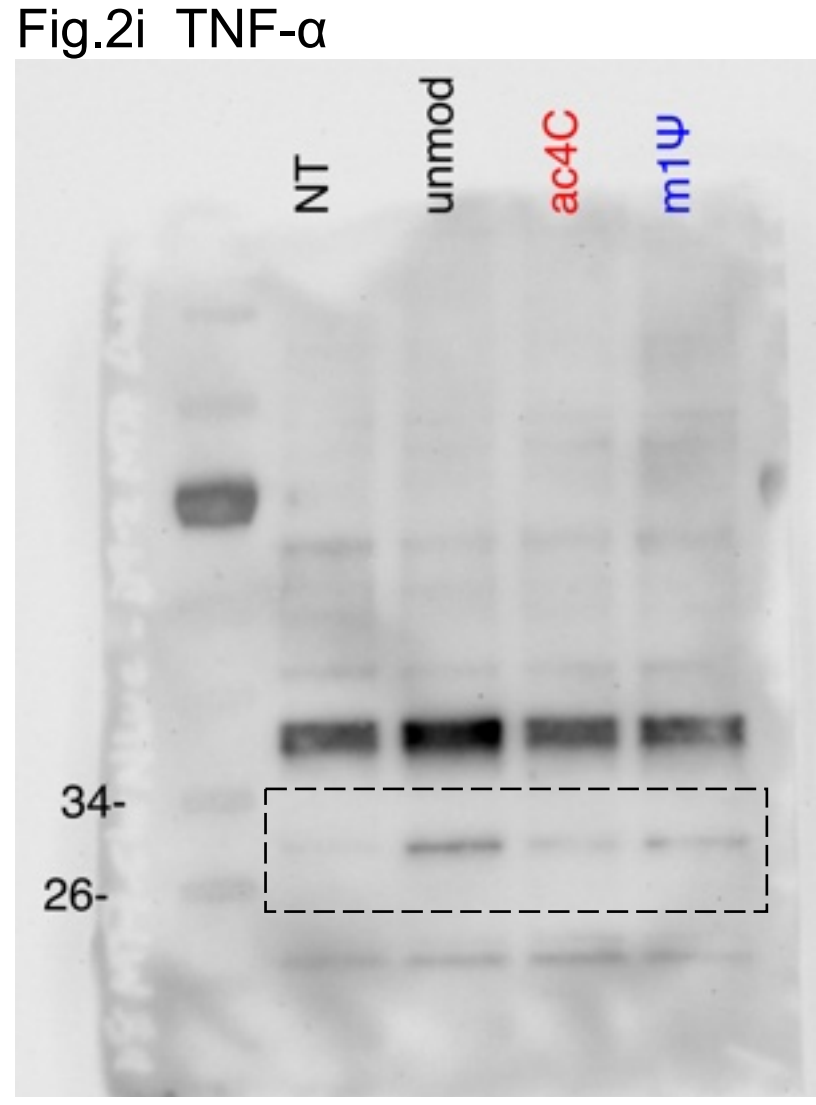

Fig.2i TNF-α

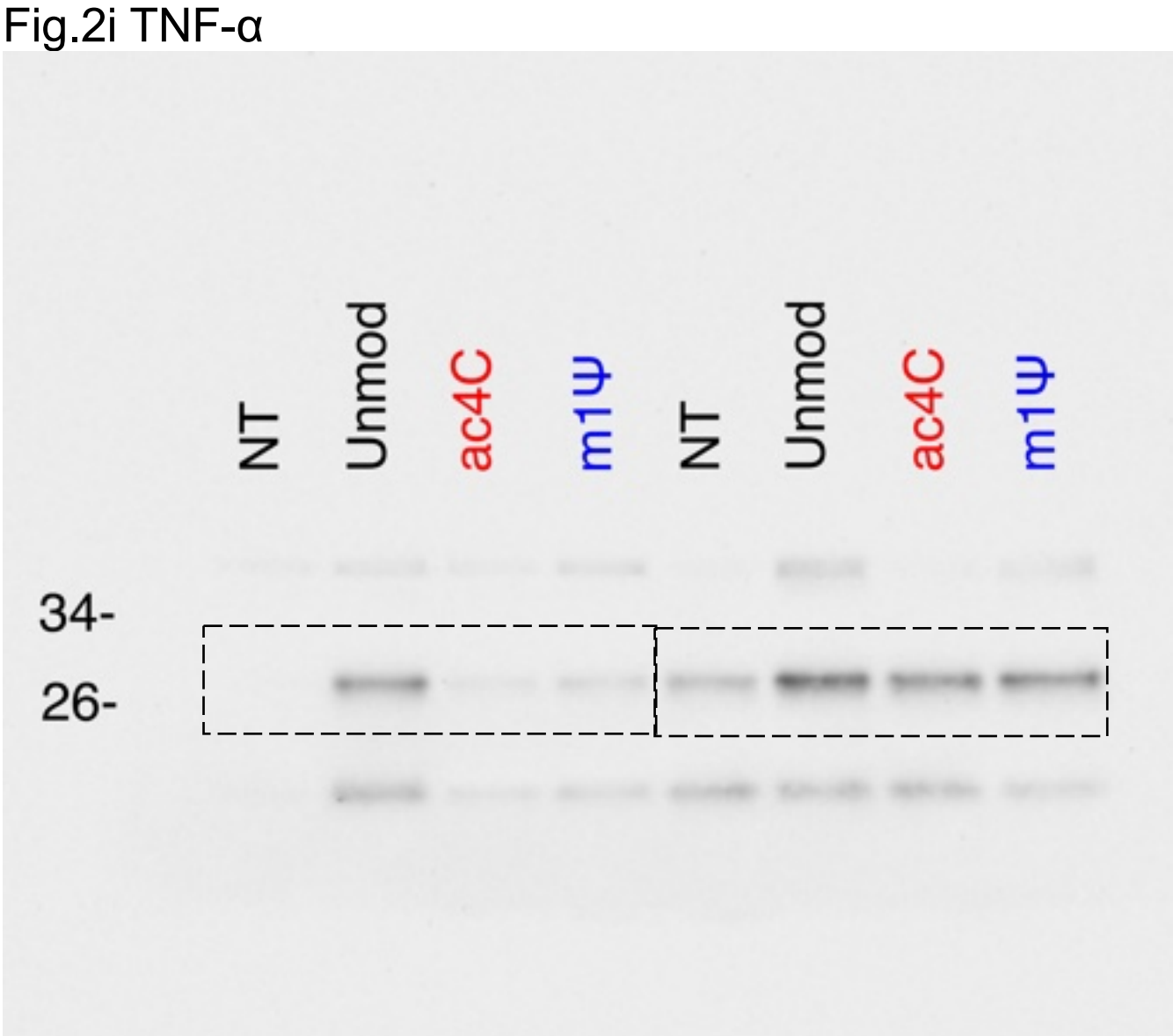

Fig.2i β-tubulin

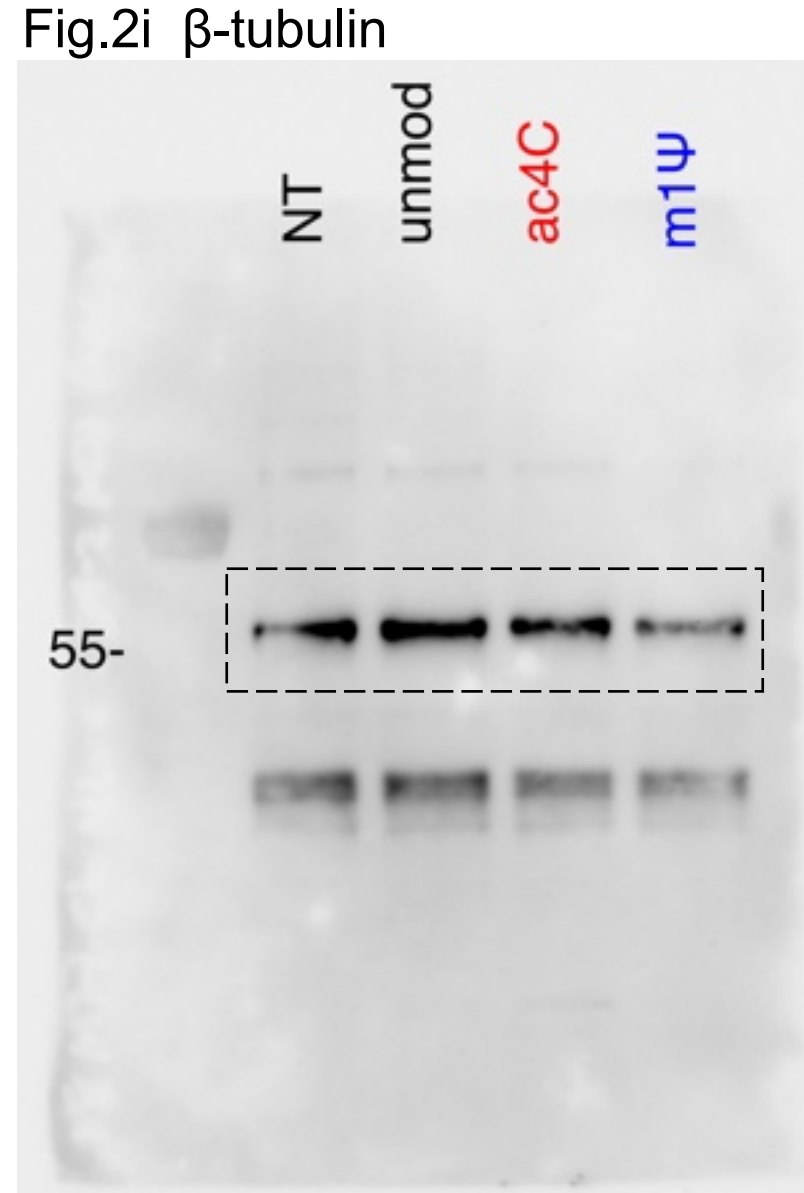

Fig.2i β-tubulin

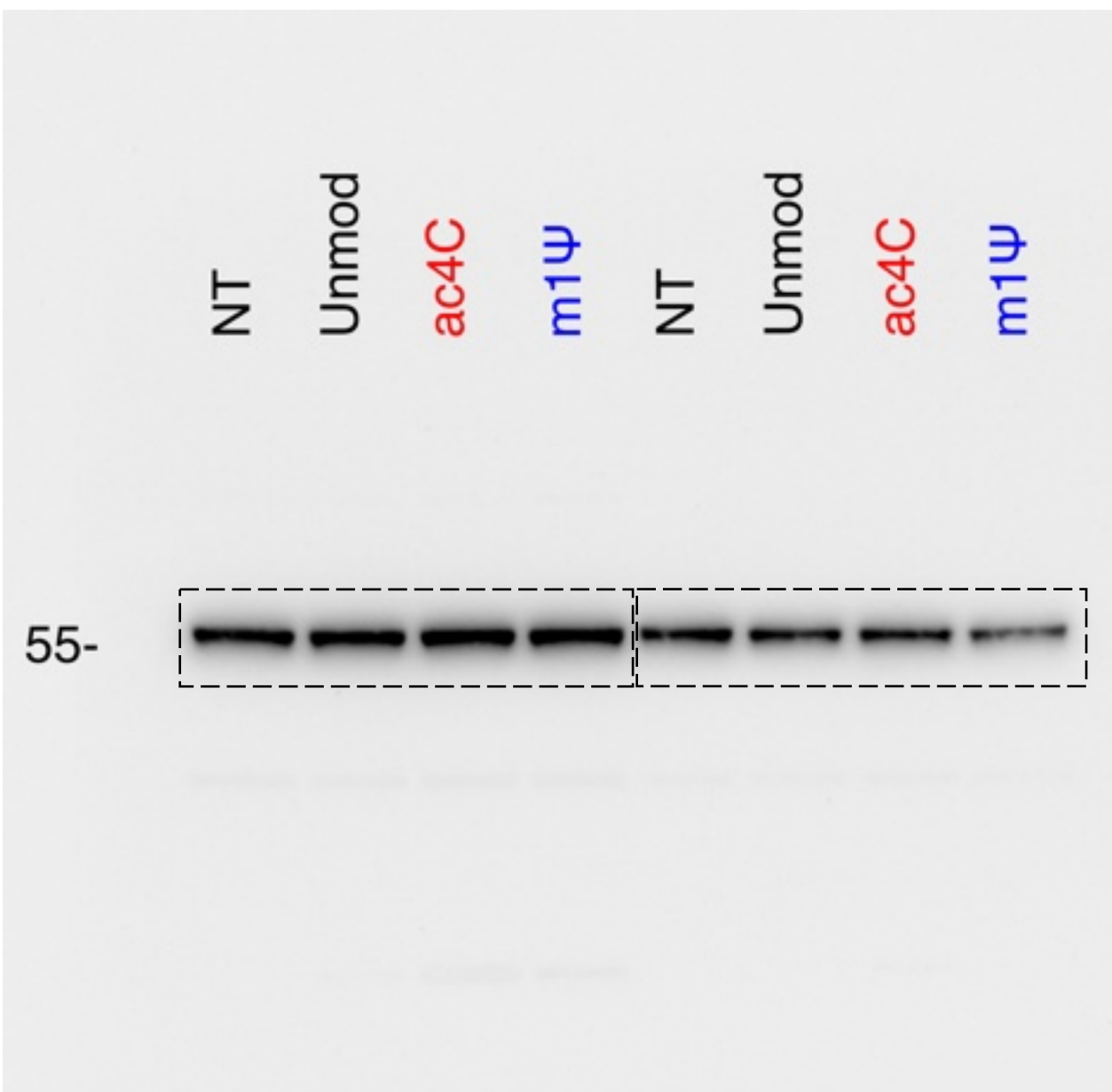

Fig.2k RNase T2 Digestion Denaturing gel

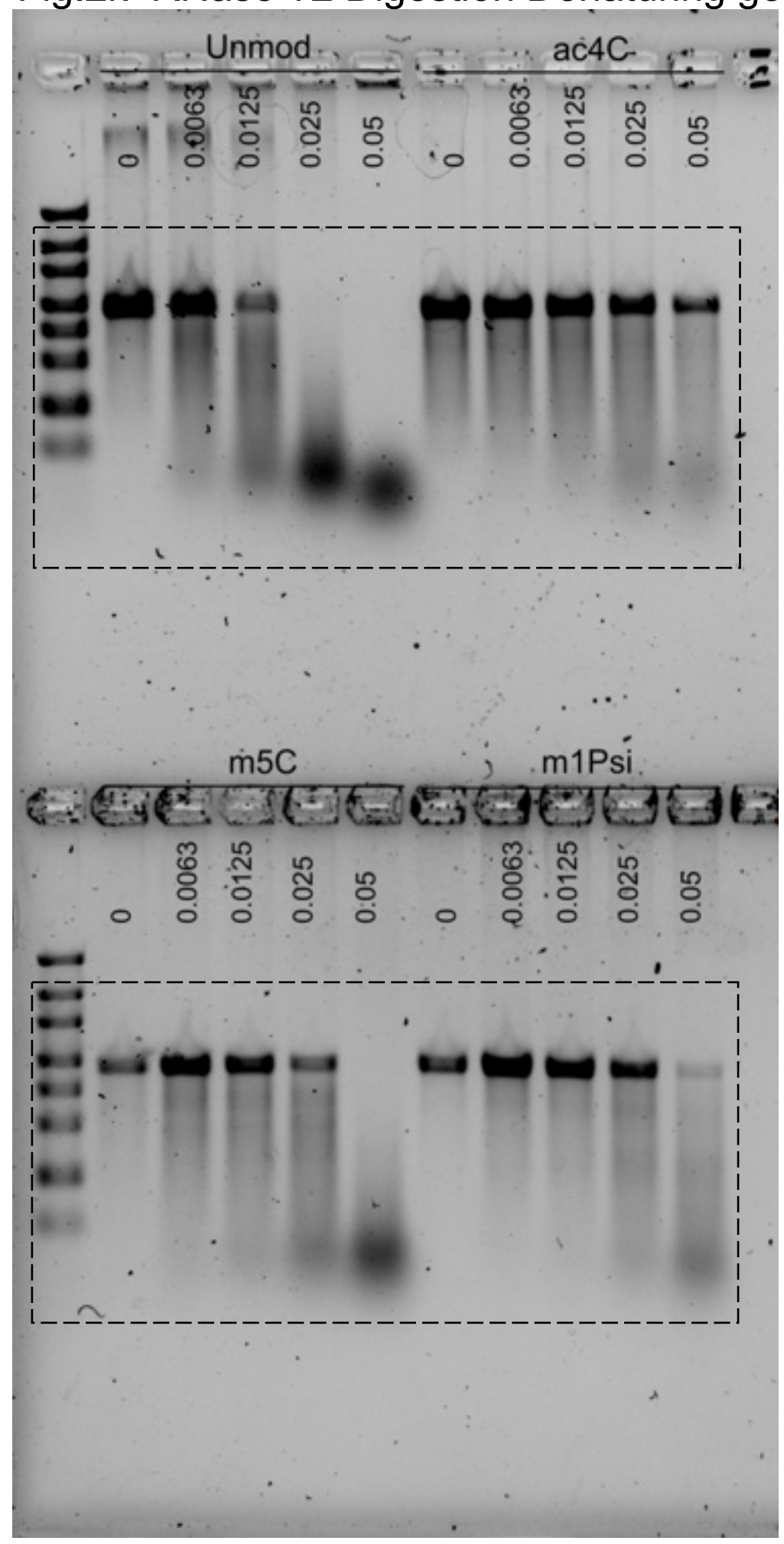

Fig.2o MEF NanoLuc

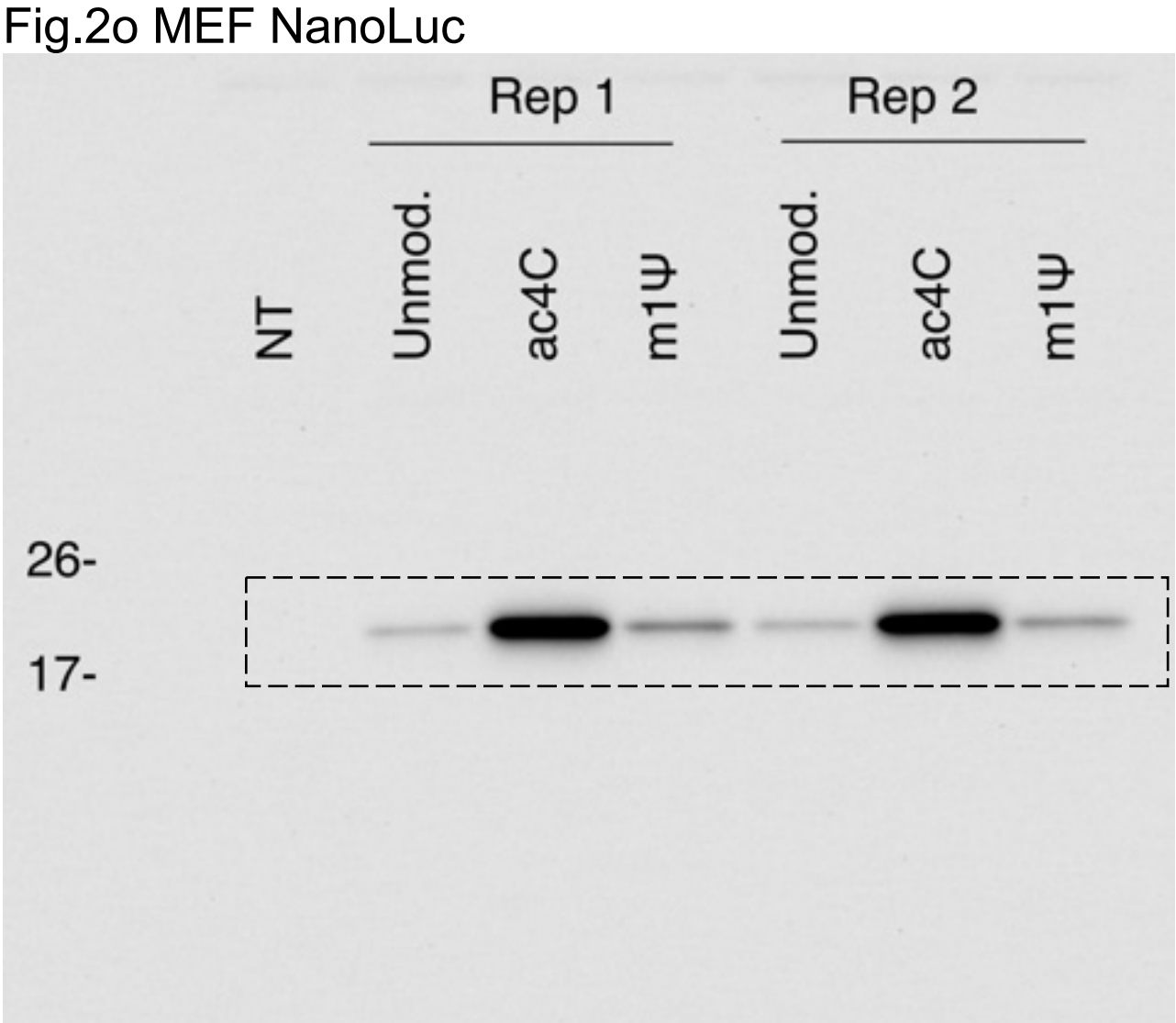

Fig.2o MEF p- eIF2-α

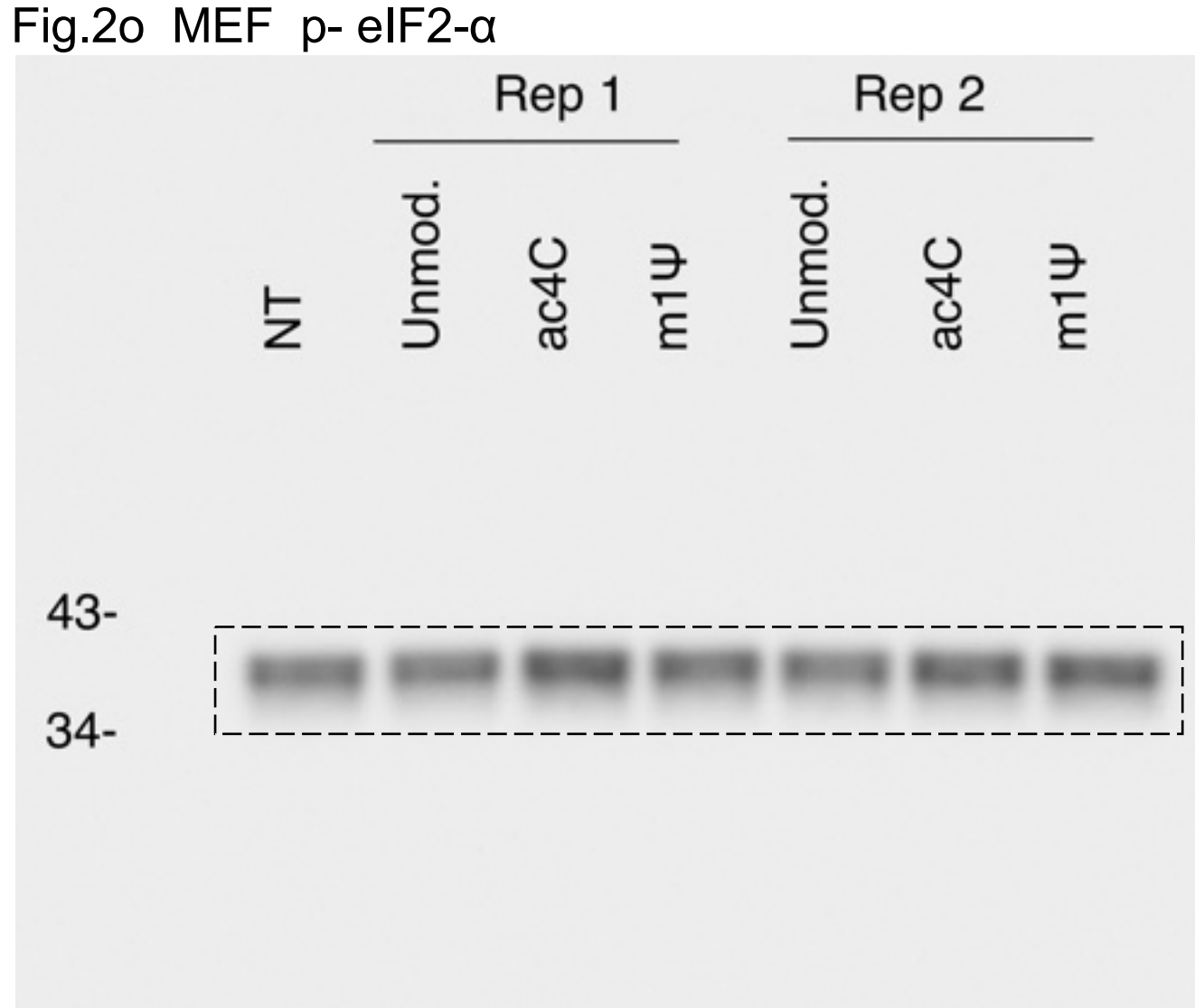

Fig.2o MEF eIF2-α

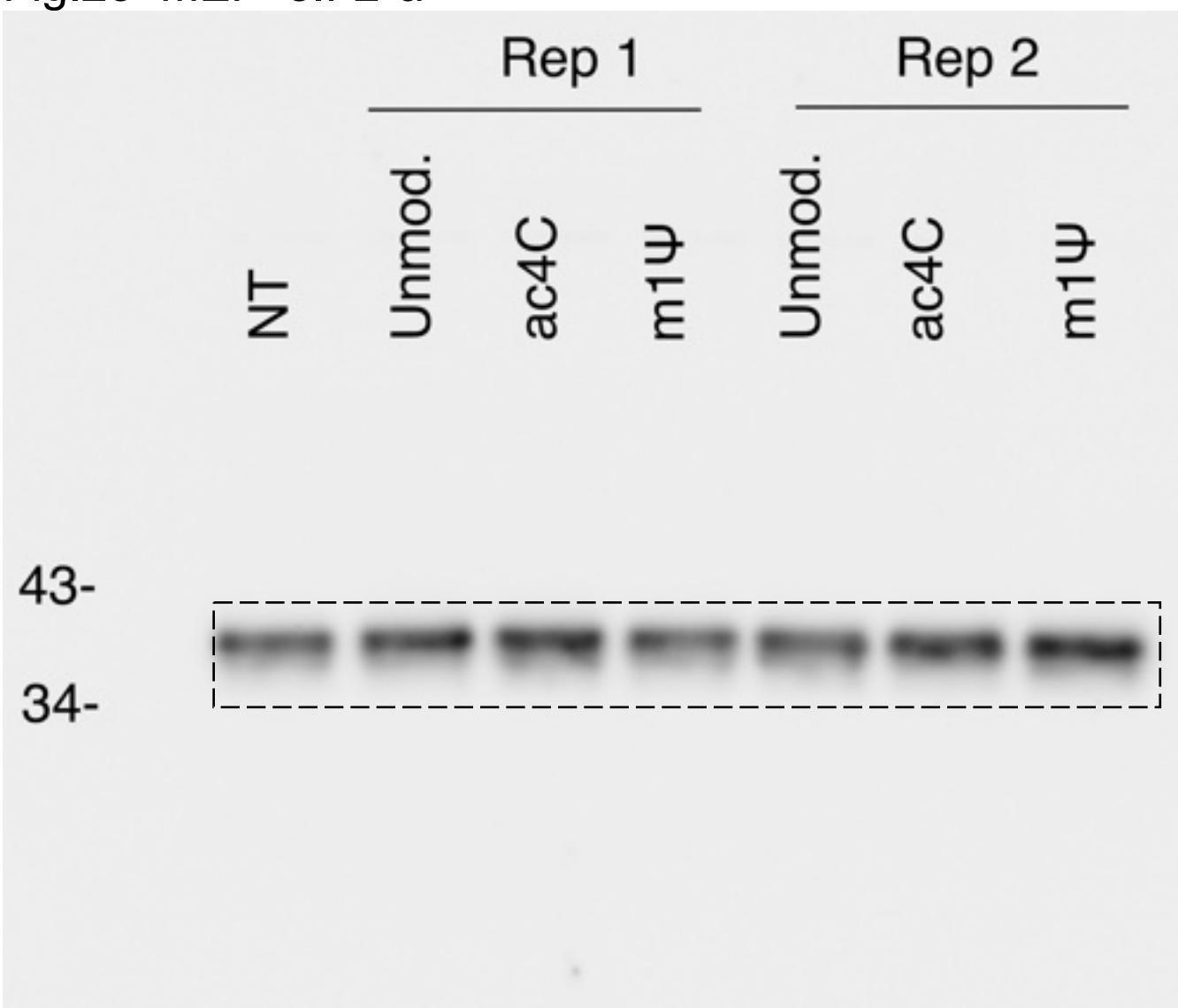

Fig.2o MEF β-tubulin

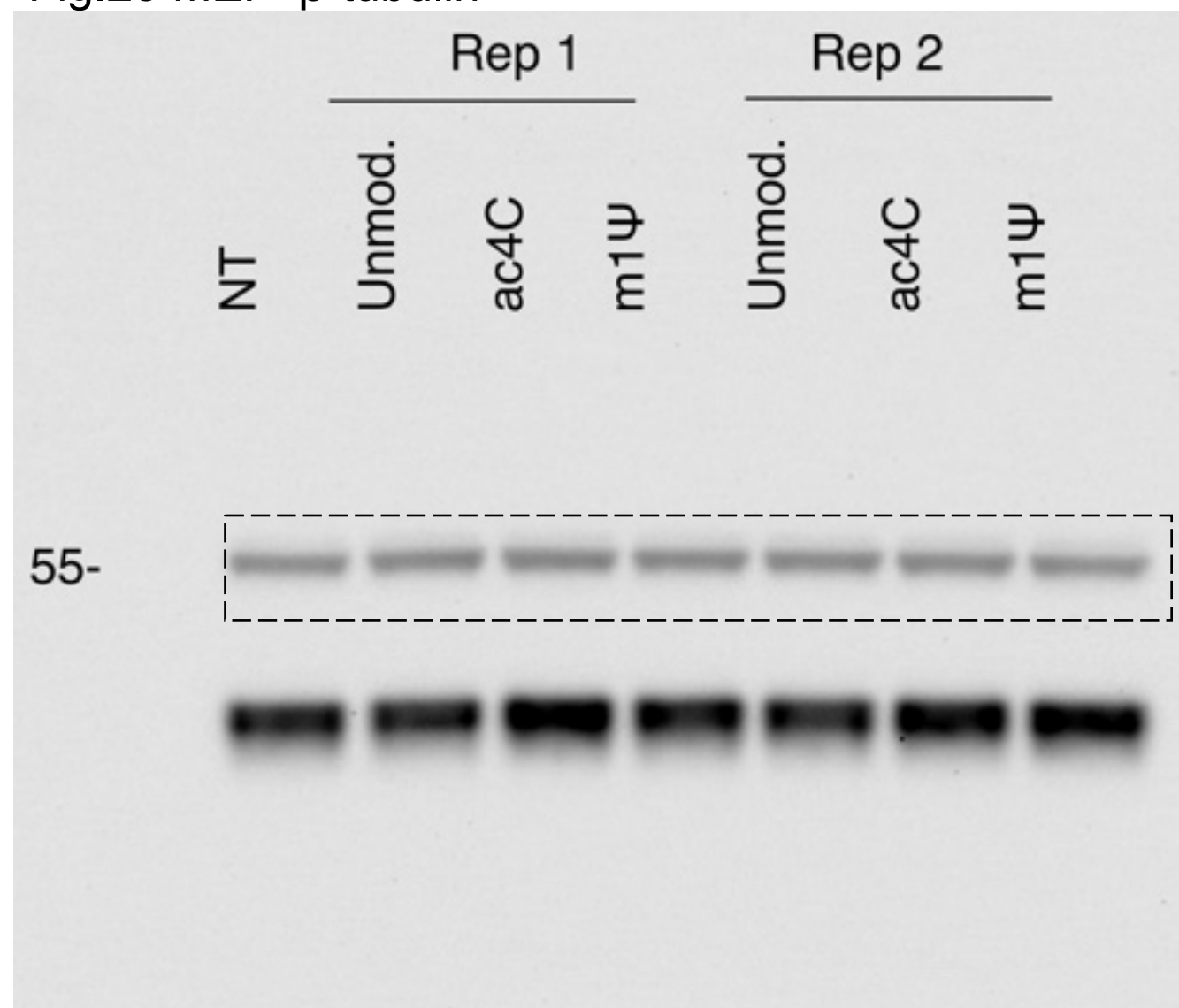

Extended Data Figure 3j NanoLuc DMSO

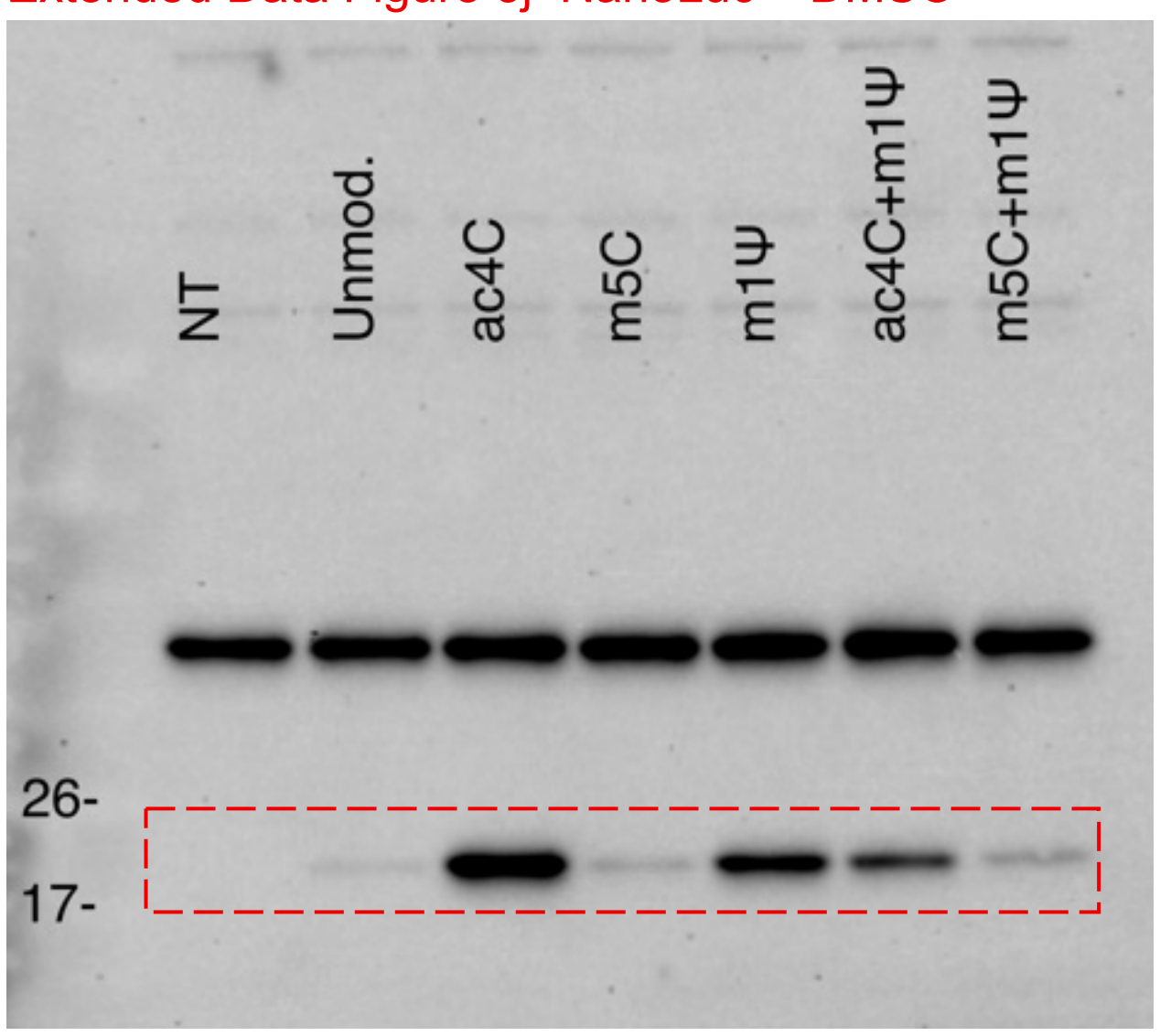

Extended Data Figure 3j NanoLuc PKR

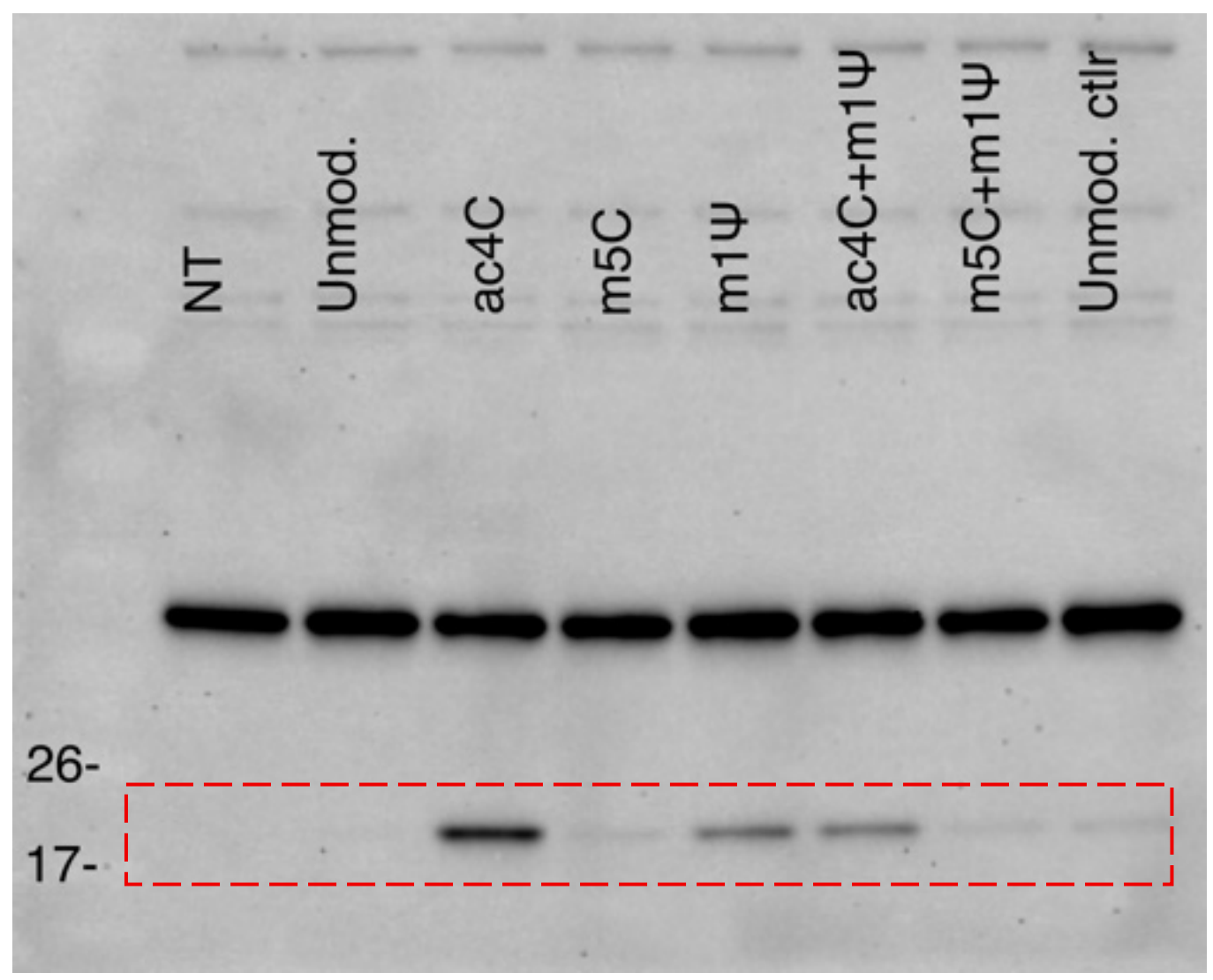

Extended Data Figure 3j p-PKR DMSO

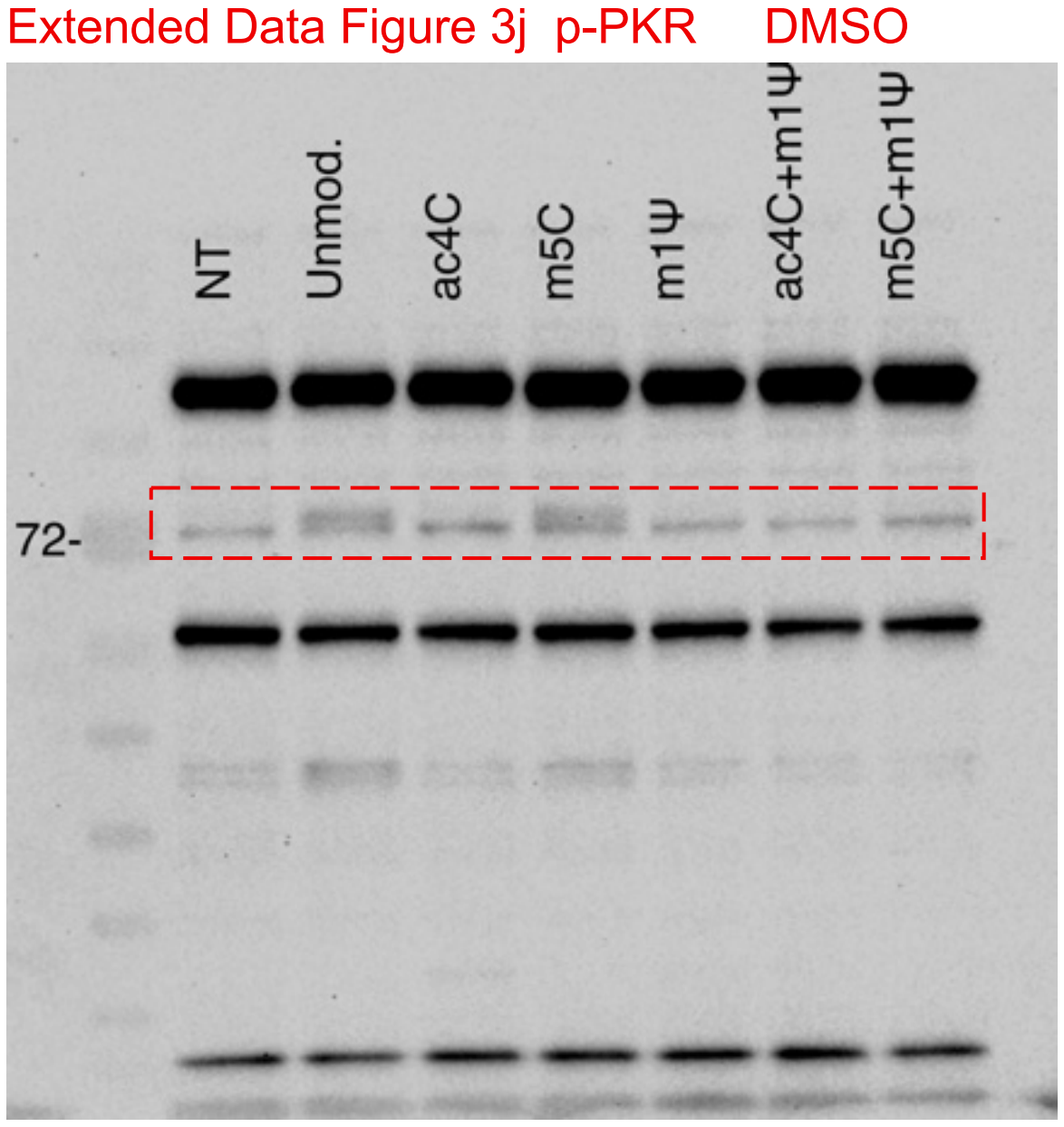

Extended Data Figure 3j p-PKR PKR inhibition

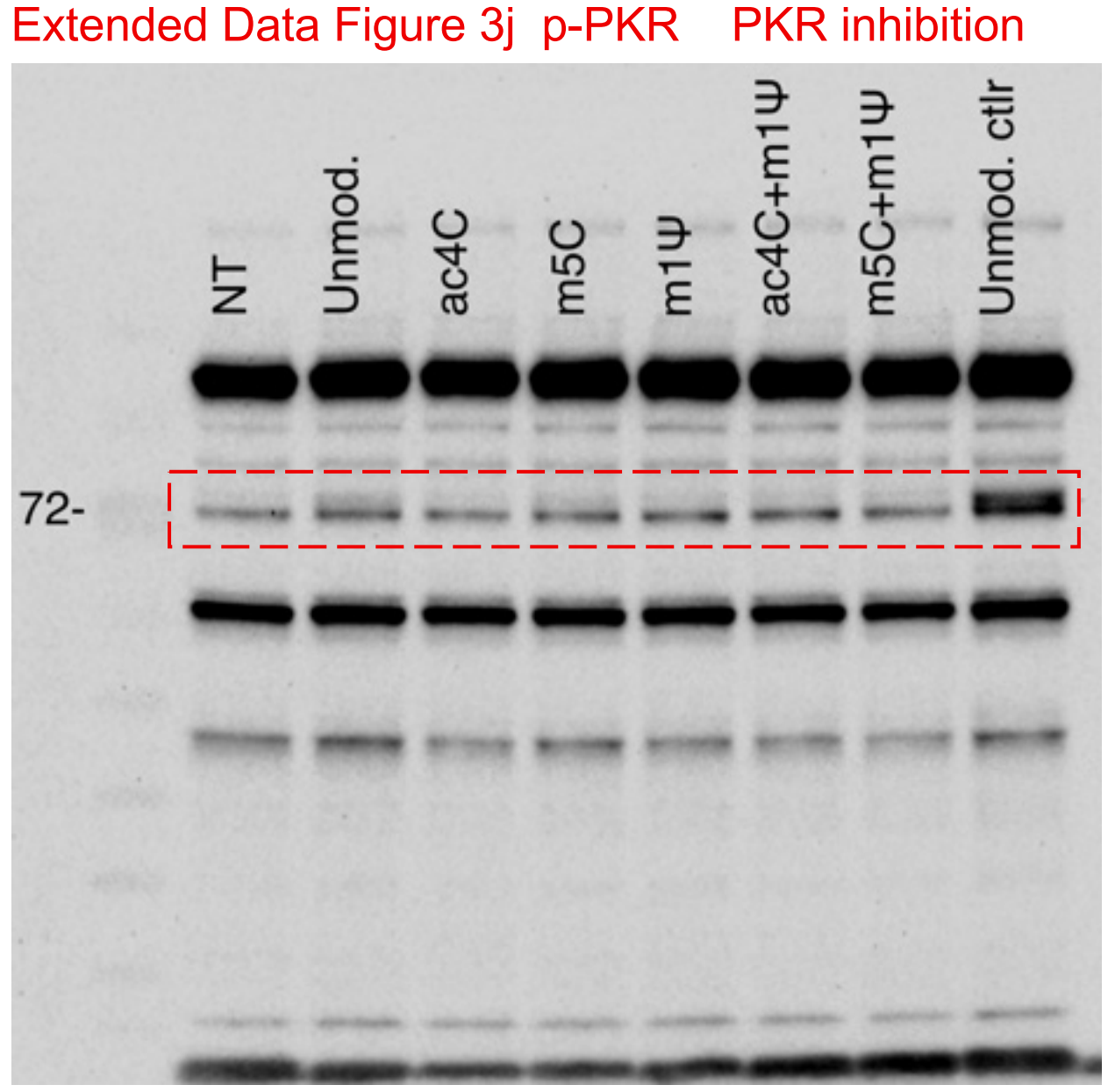

Extended Data Figure 3j PKR DMSO

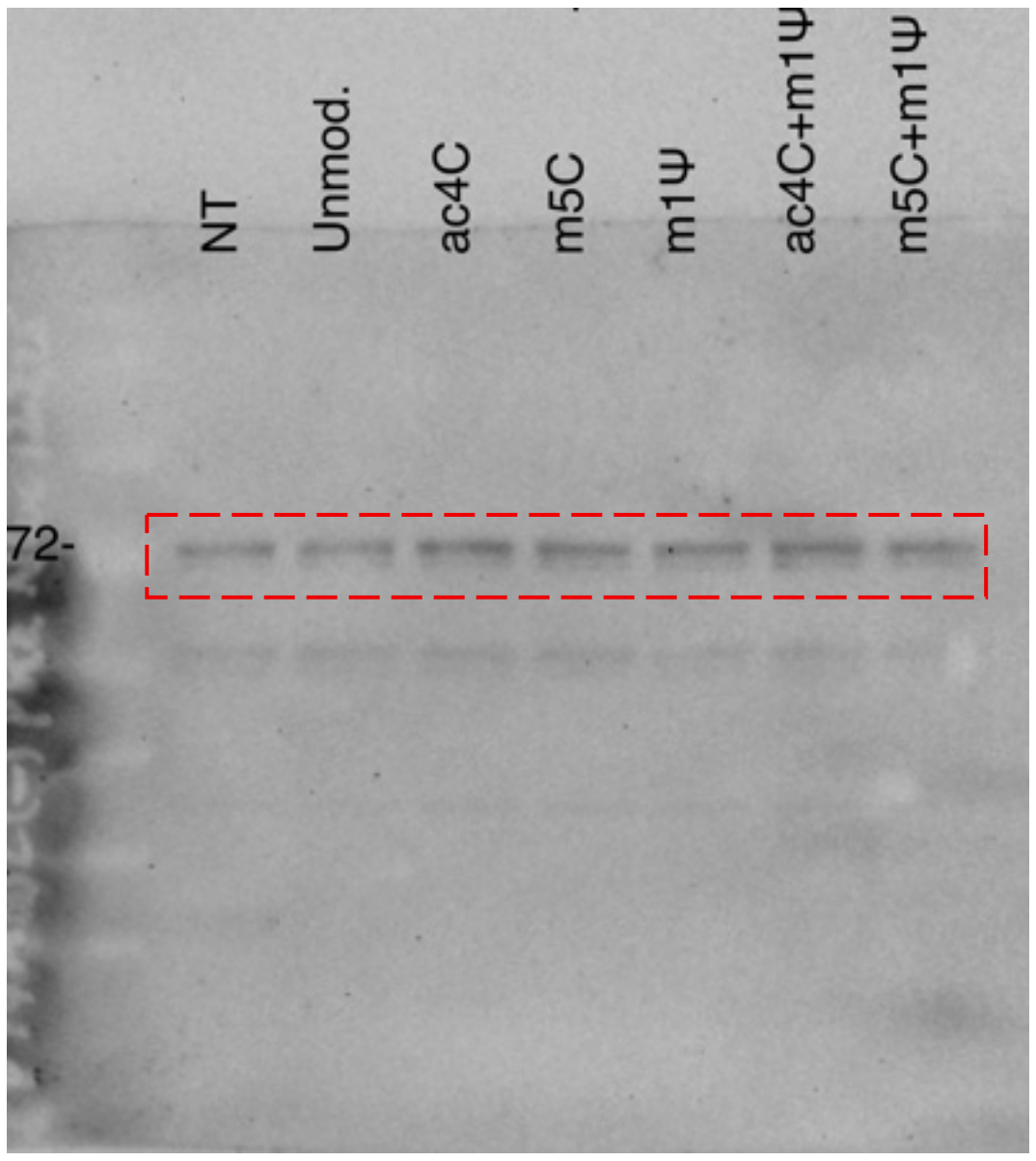

Extended Data Figure 3j PKR PKR inhibition

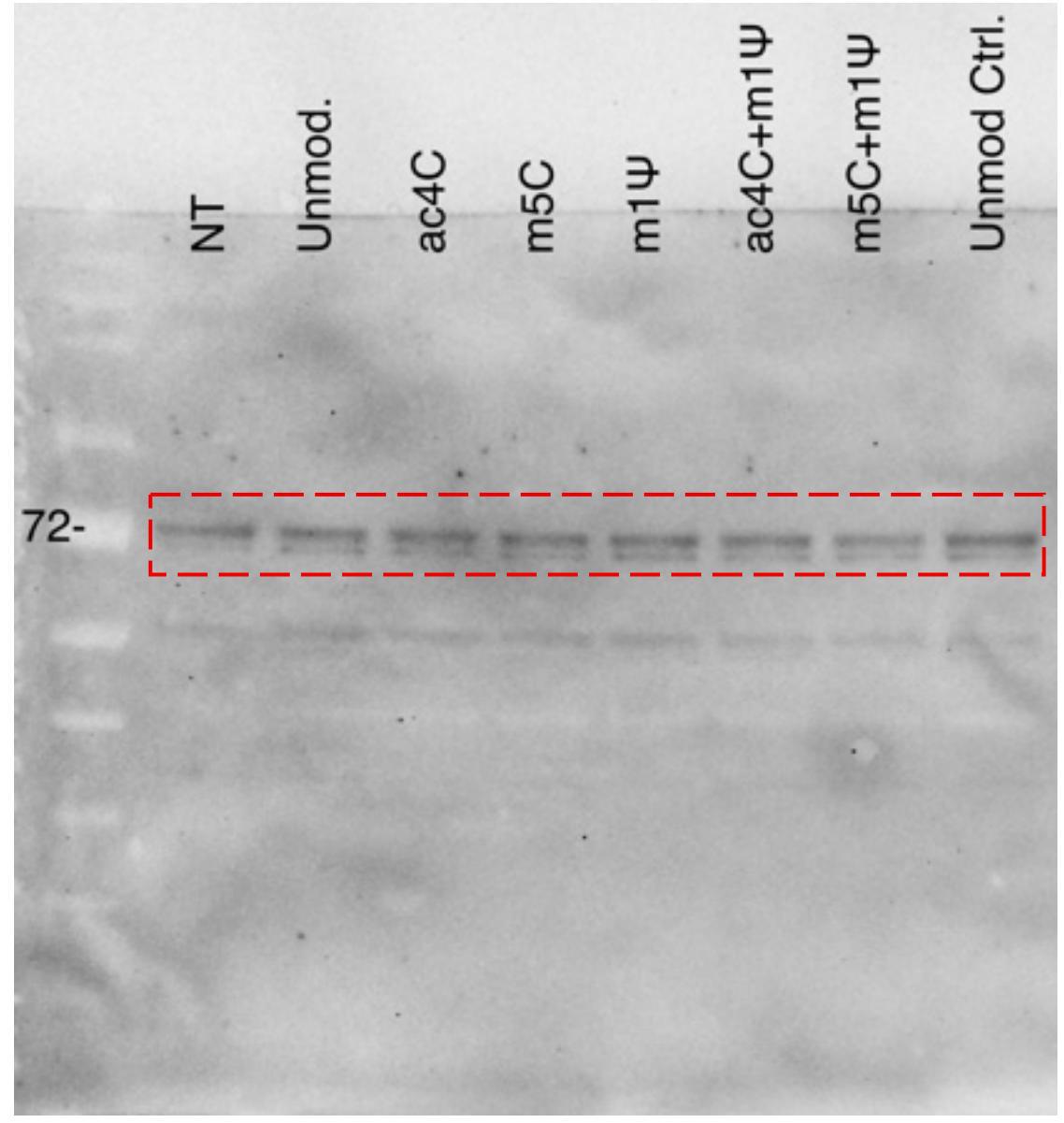

Extended Data Figure 3j p-eIF2-α DMSO

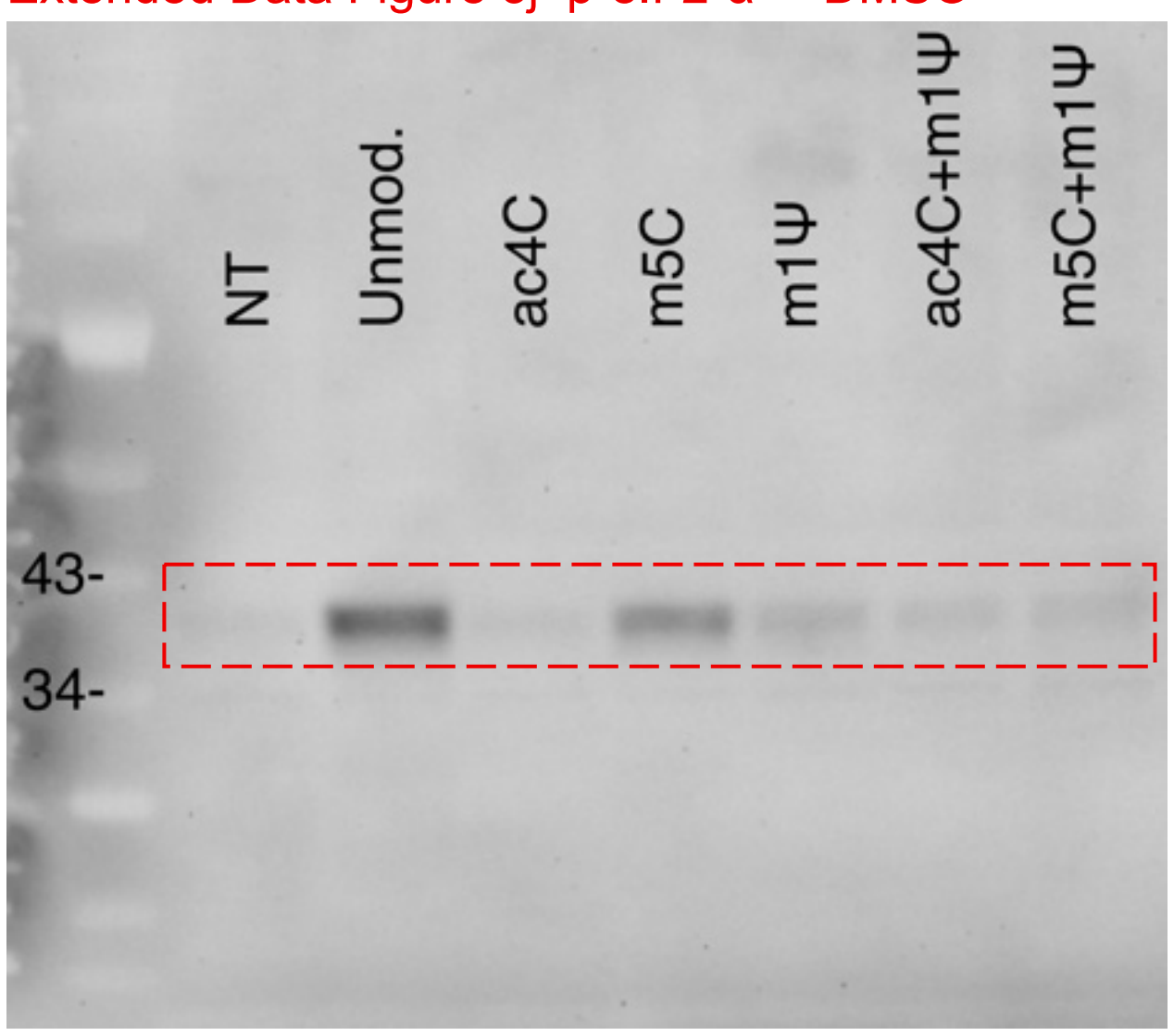

Extended Data Figure 3j p-eIF2-α PKR inhibition

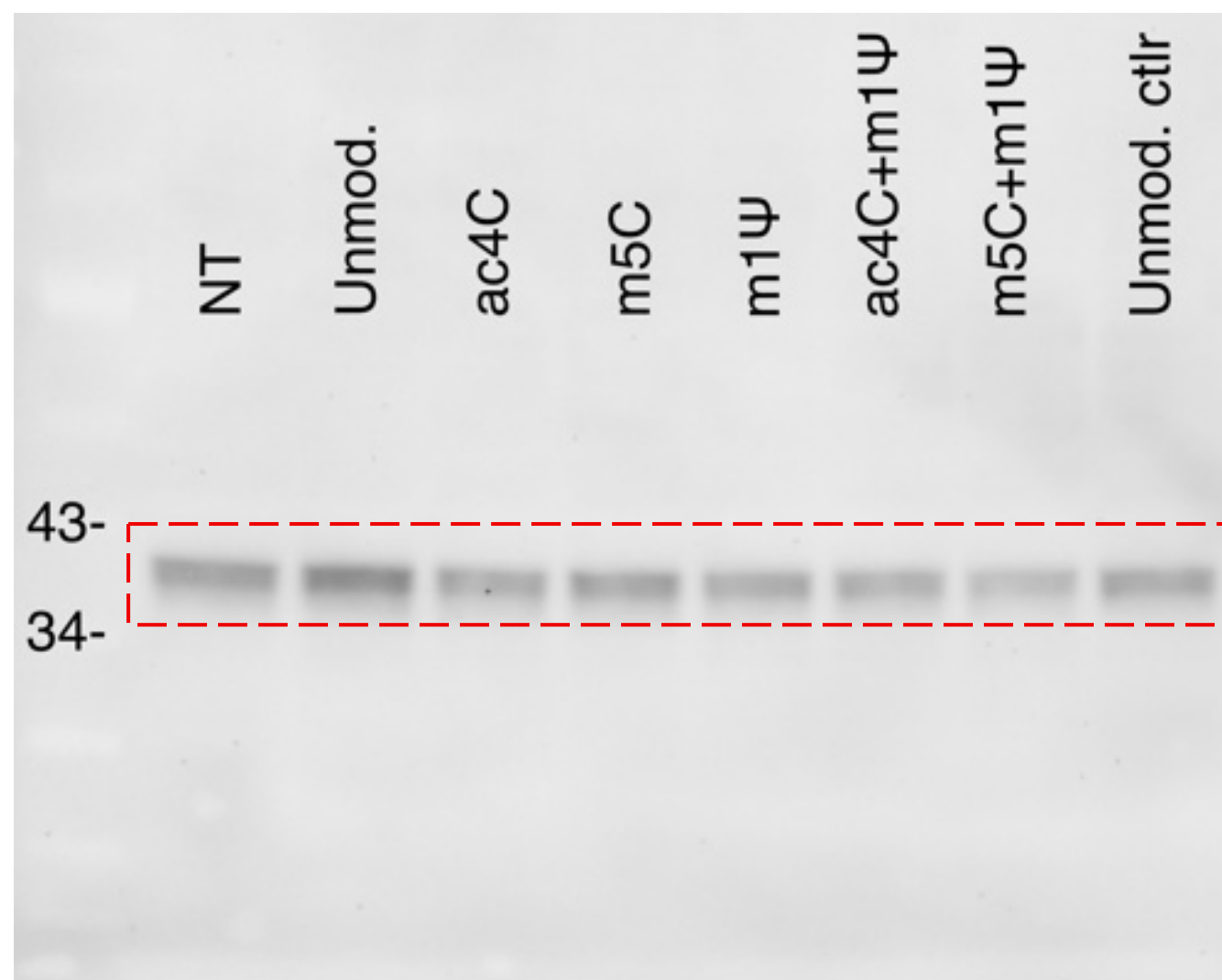

Extended Data Figure 3j eIF2-α DMSO

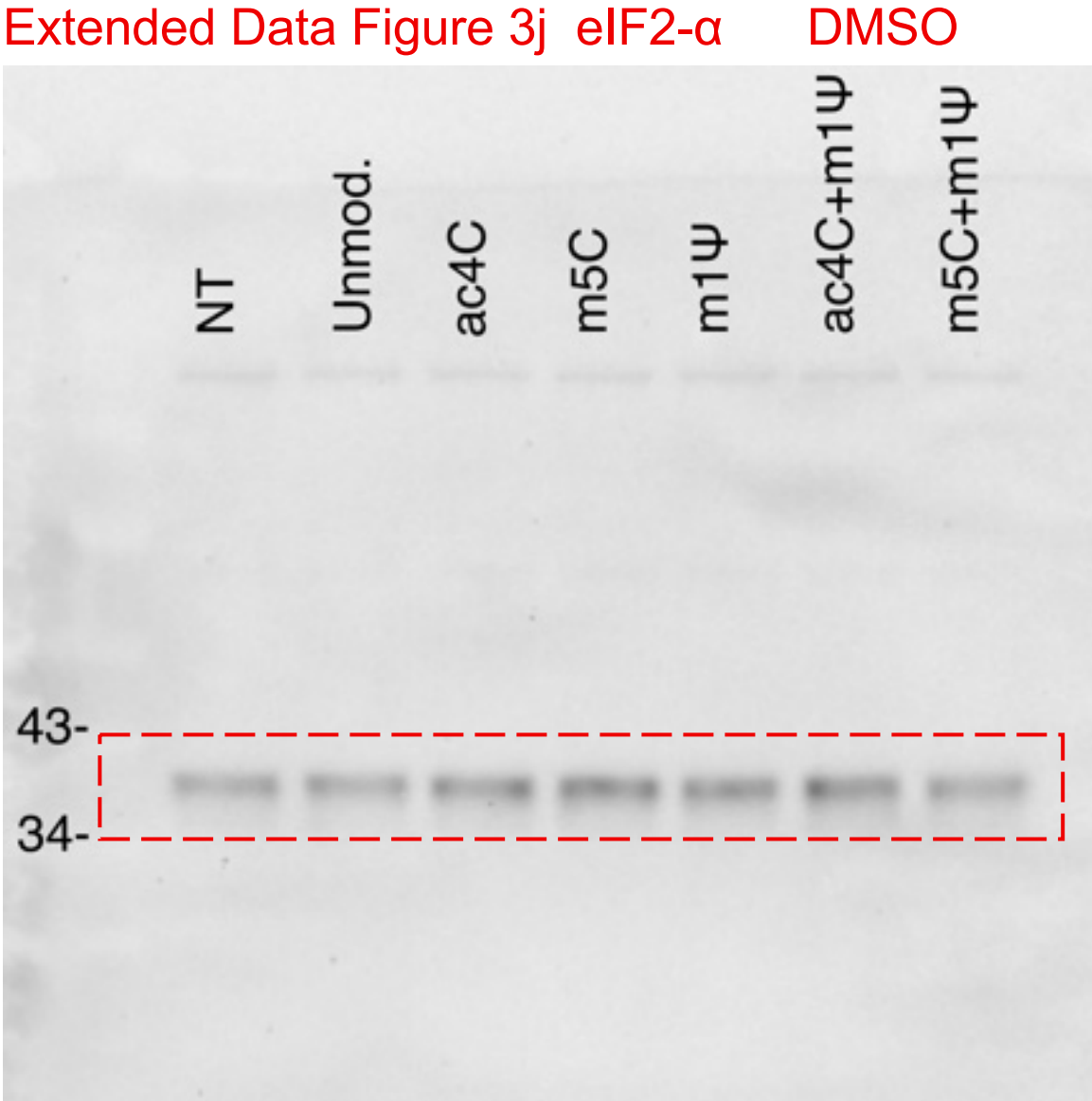

Extended Data Figure 3j eIF2-α PKR inhibition

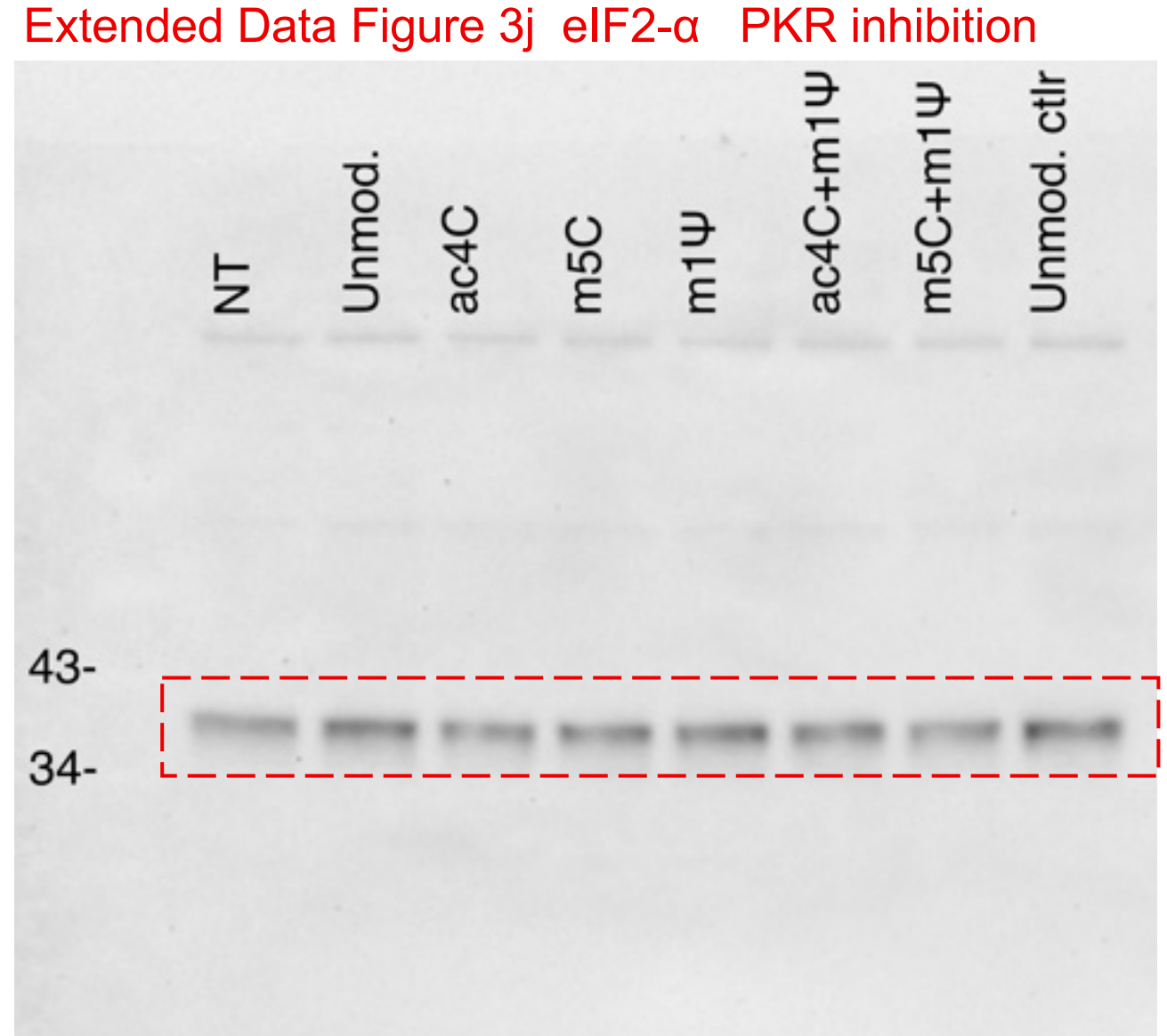

Extended Data Figure 3j β-tubulin DMSO

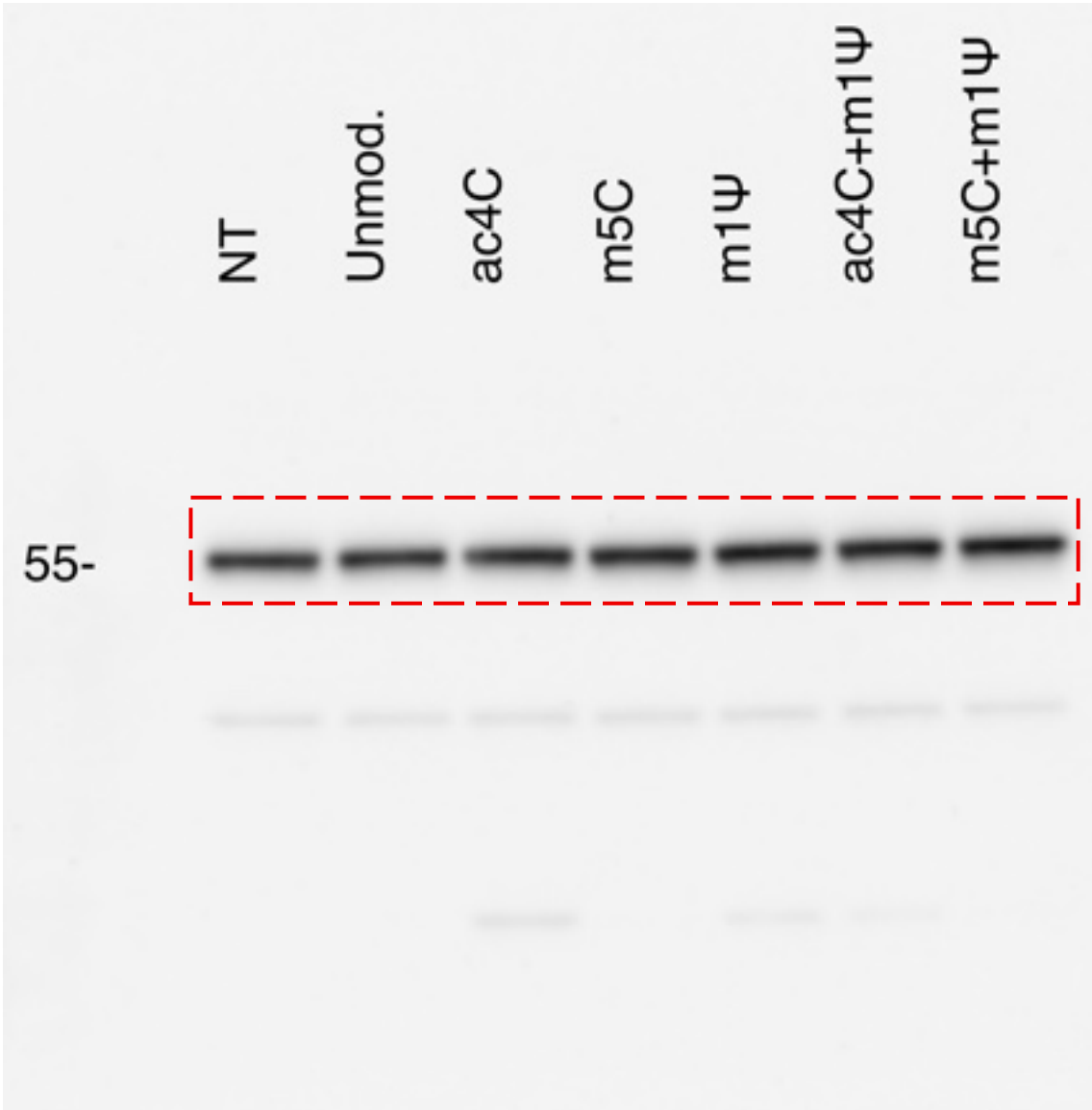

Extended Data Figure 3j β-tubulin PKR inhibition

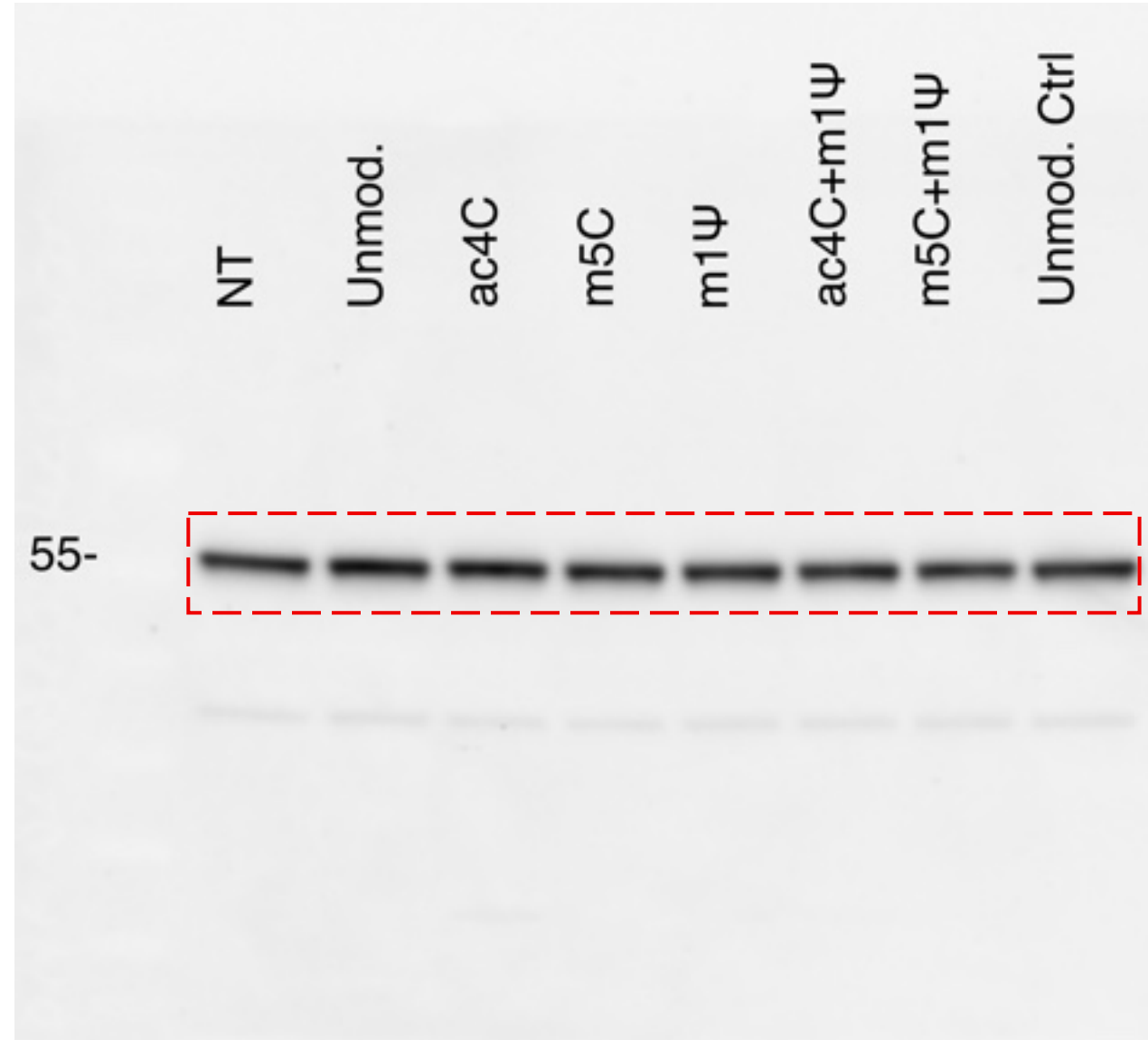

Extended Data Figure 4h TLR7 MoDc

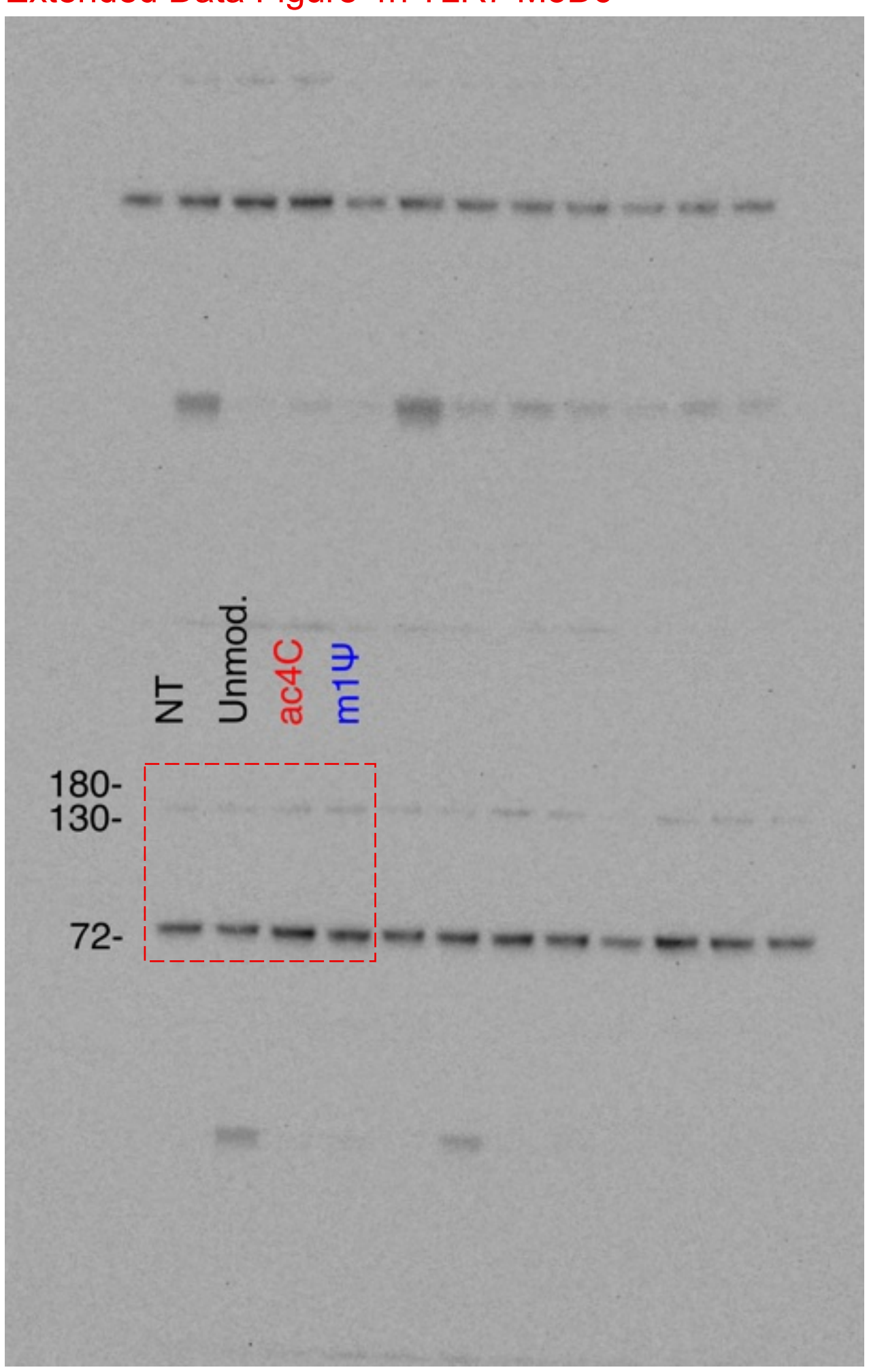

Extended Data Figure 4h TLR8 MoDc

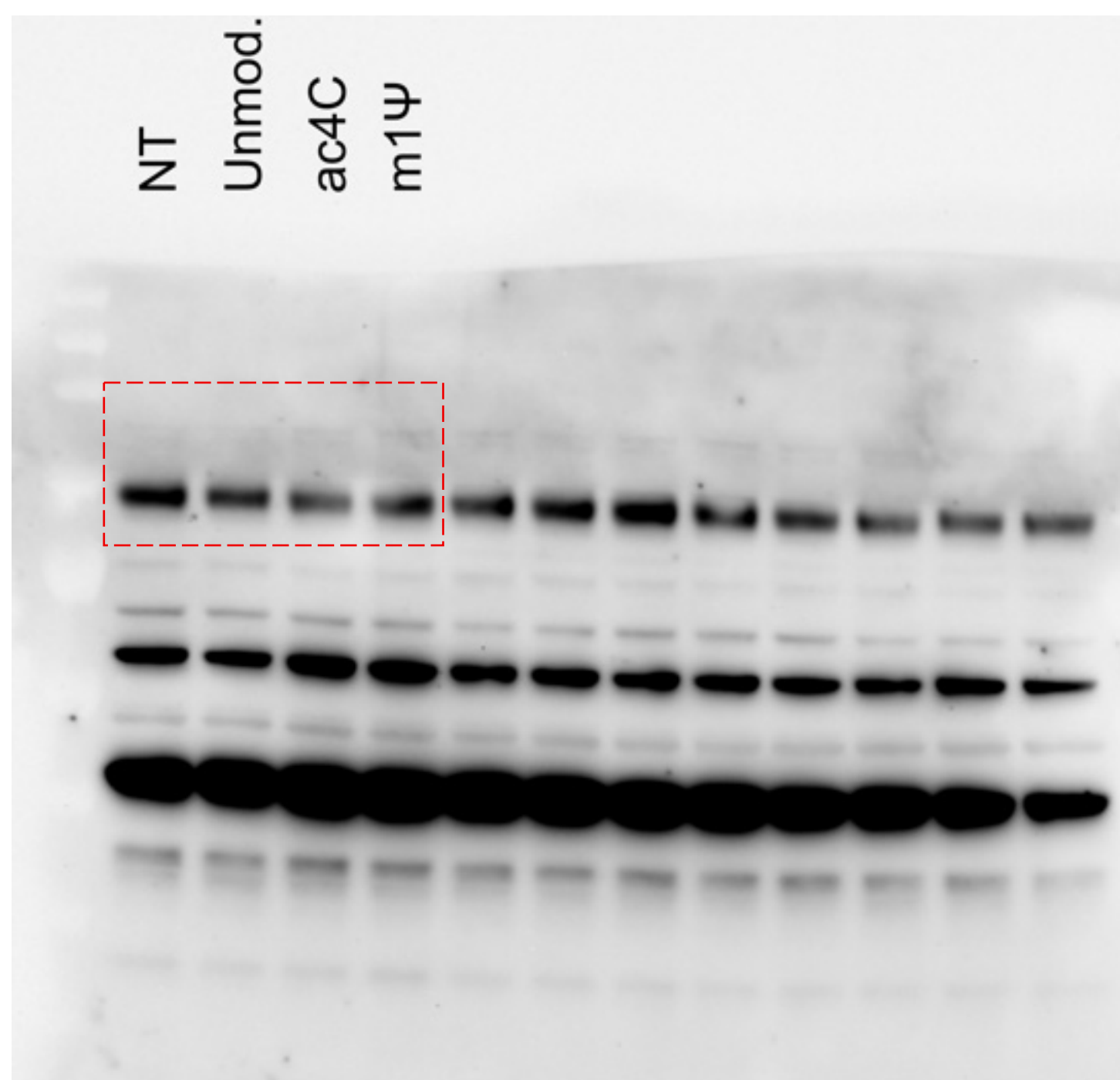

Extended Data figure 4i RNase T2 Denaturing gel

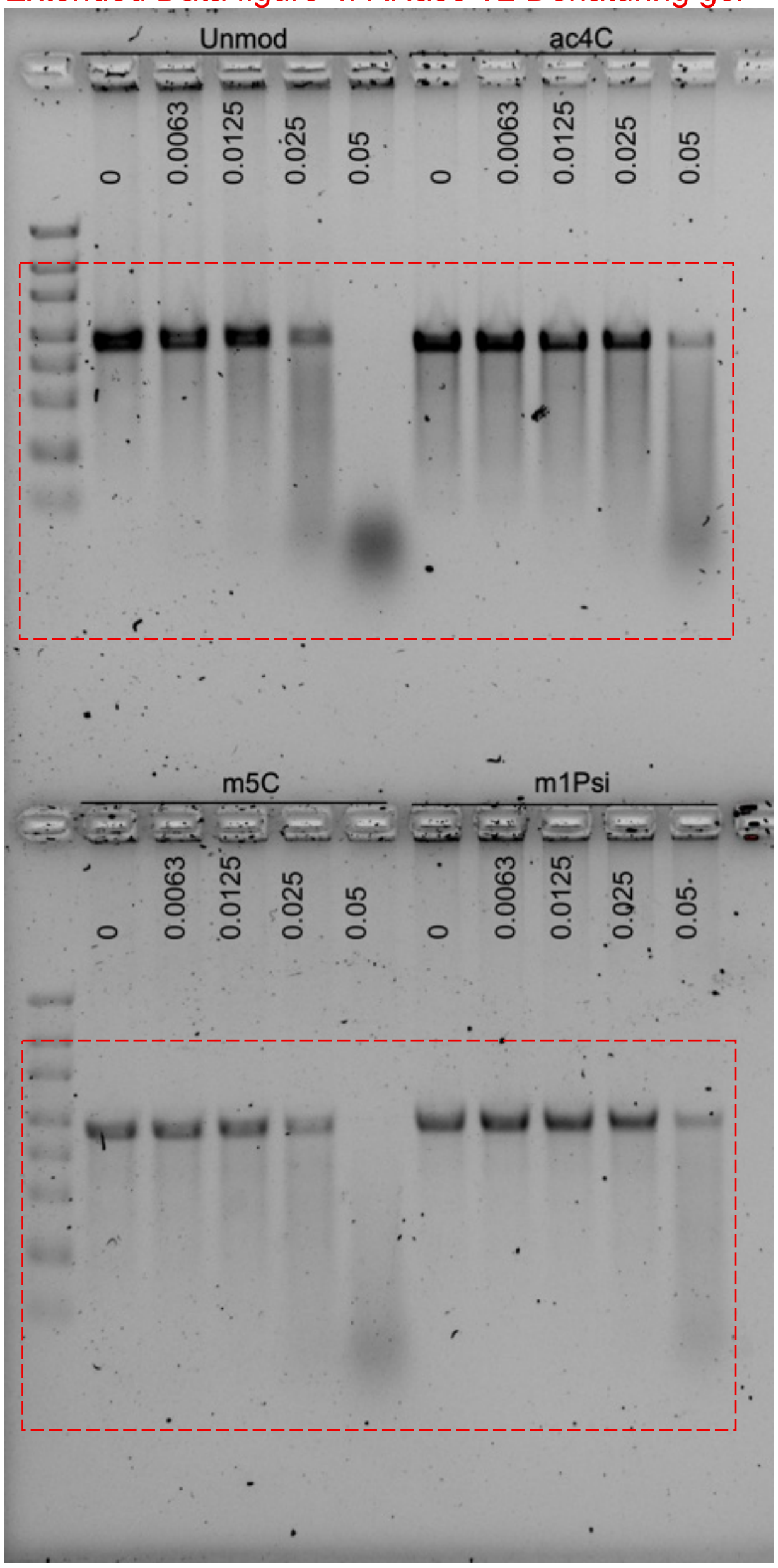

Extended Data 4e RNase T2 Denaturing gel

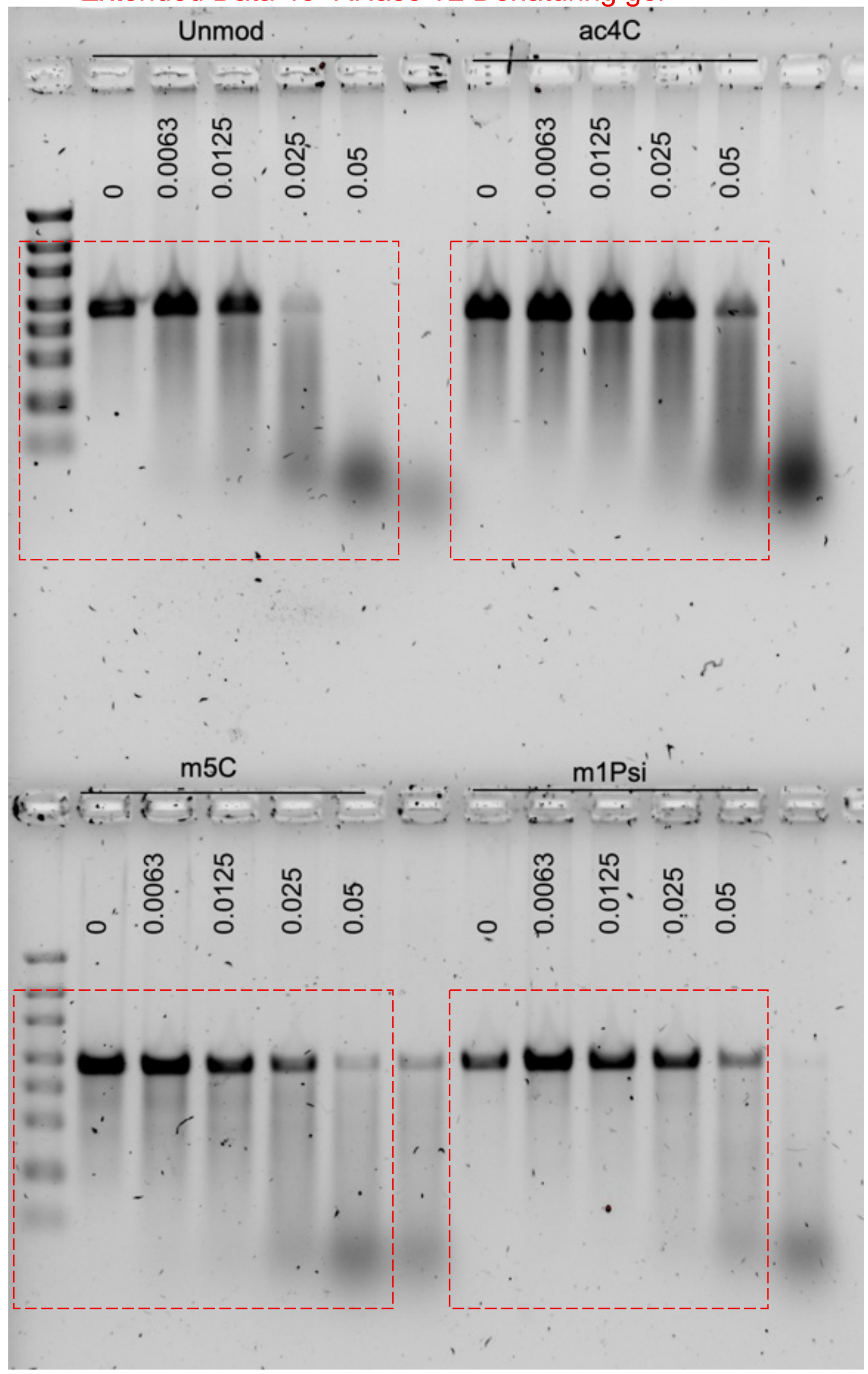

Extended Data Figure 4m Denaturing gel Total RNA

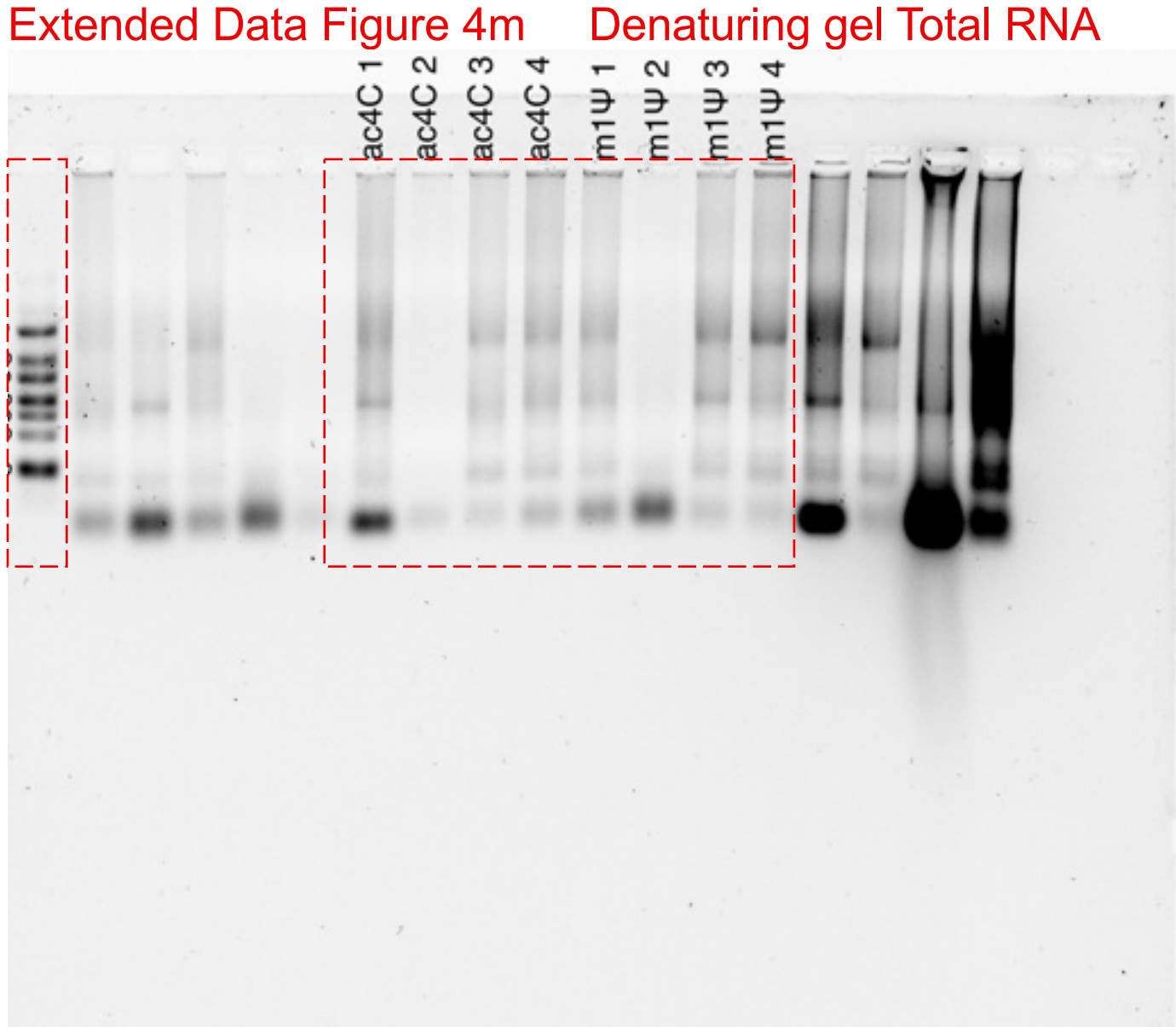

Extended data Figure 4p MEFs NanoLuc

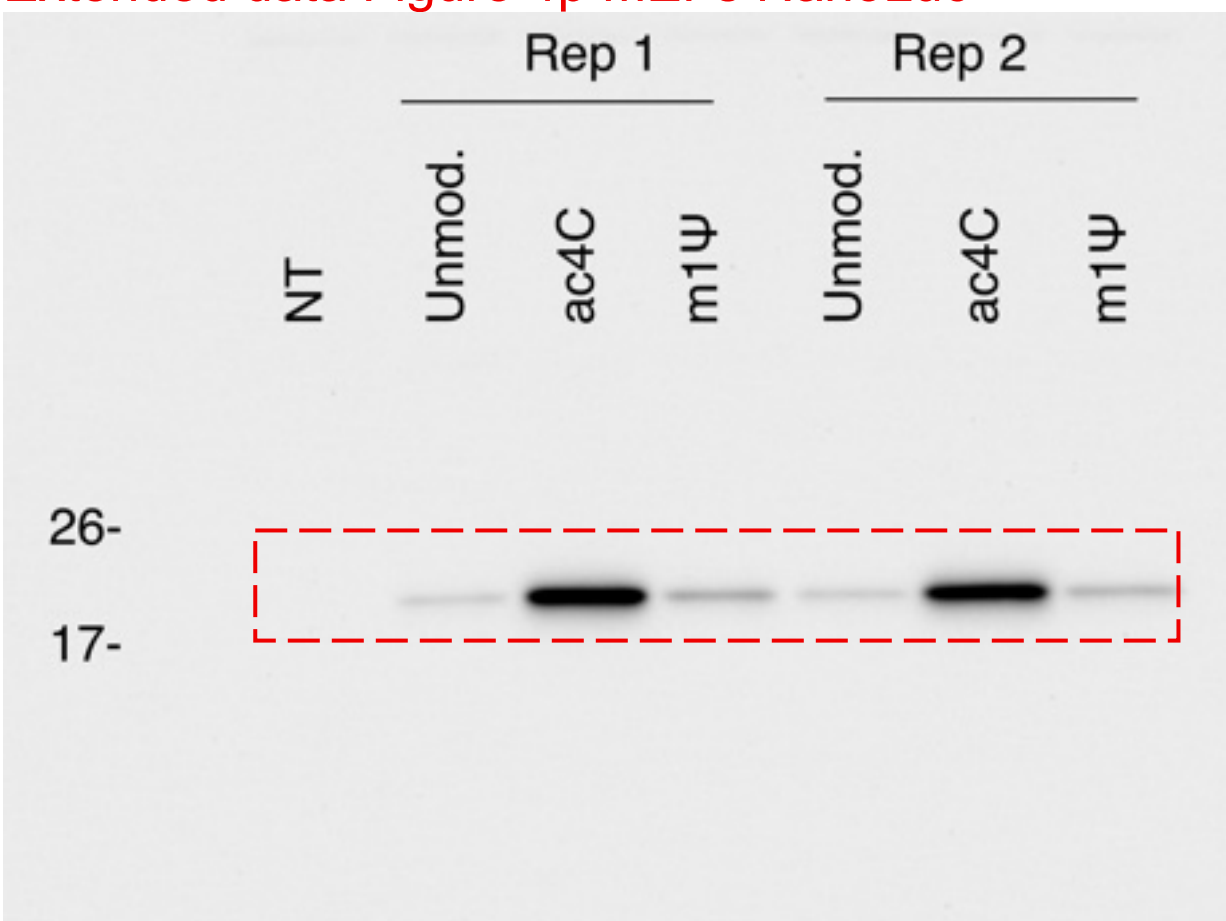

Extended data Figure 4p MEFs p- eIF2-α

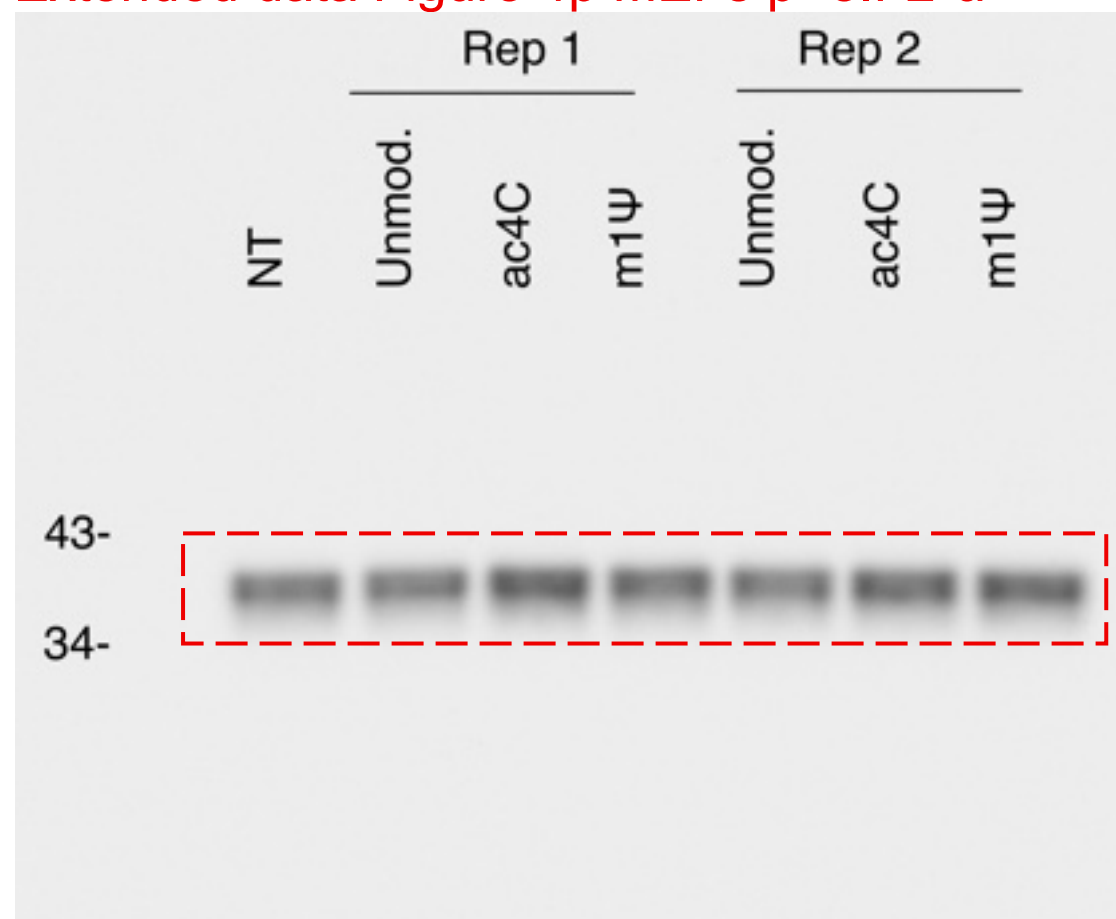

Extended data Figure 4p MEFs eIF2-α

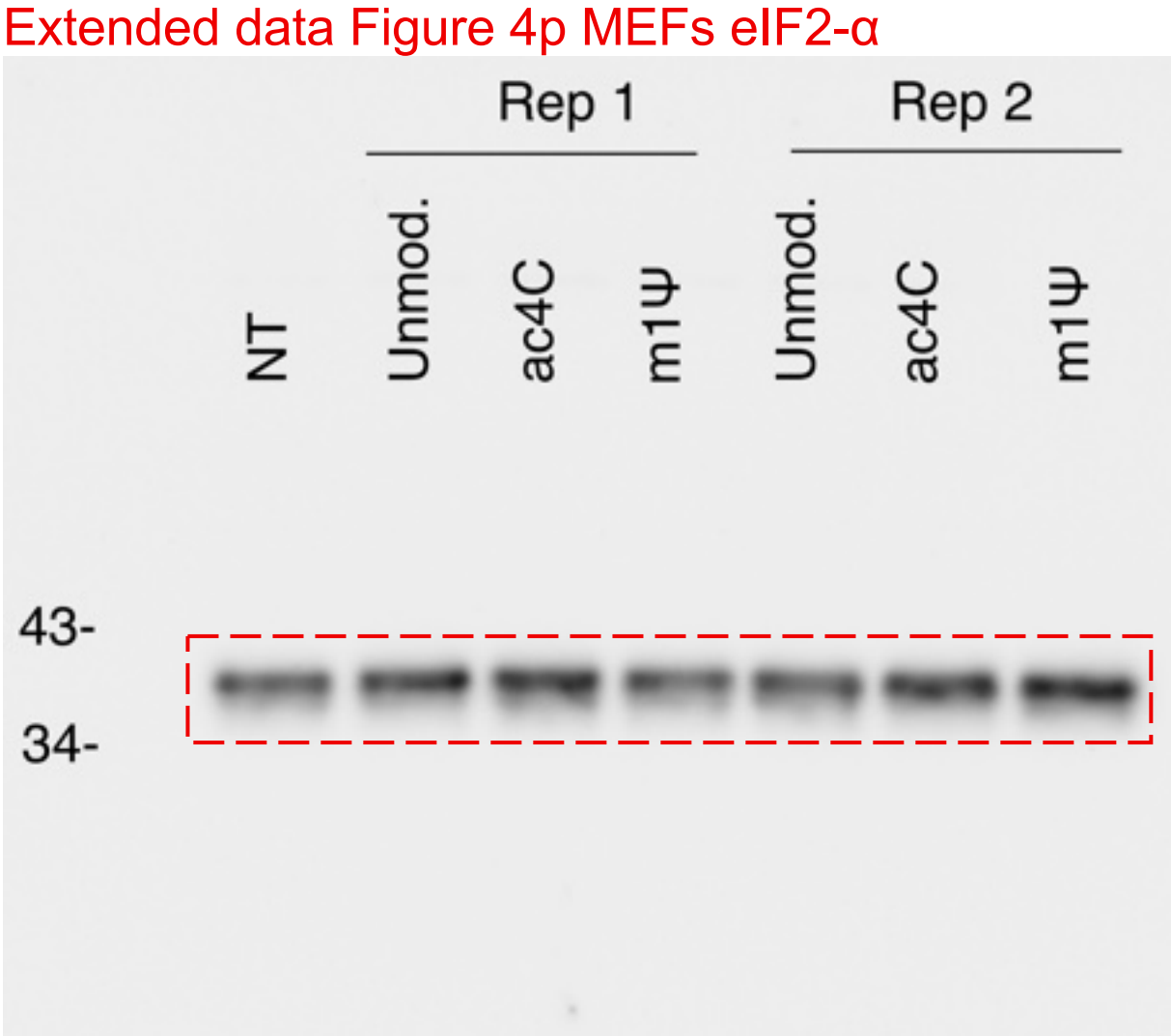

Extended data Figure 4p MEFs β-tubulin

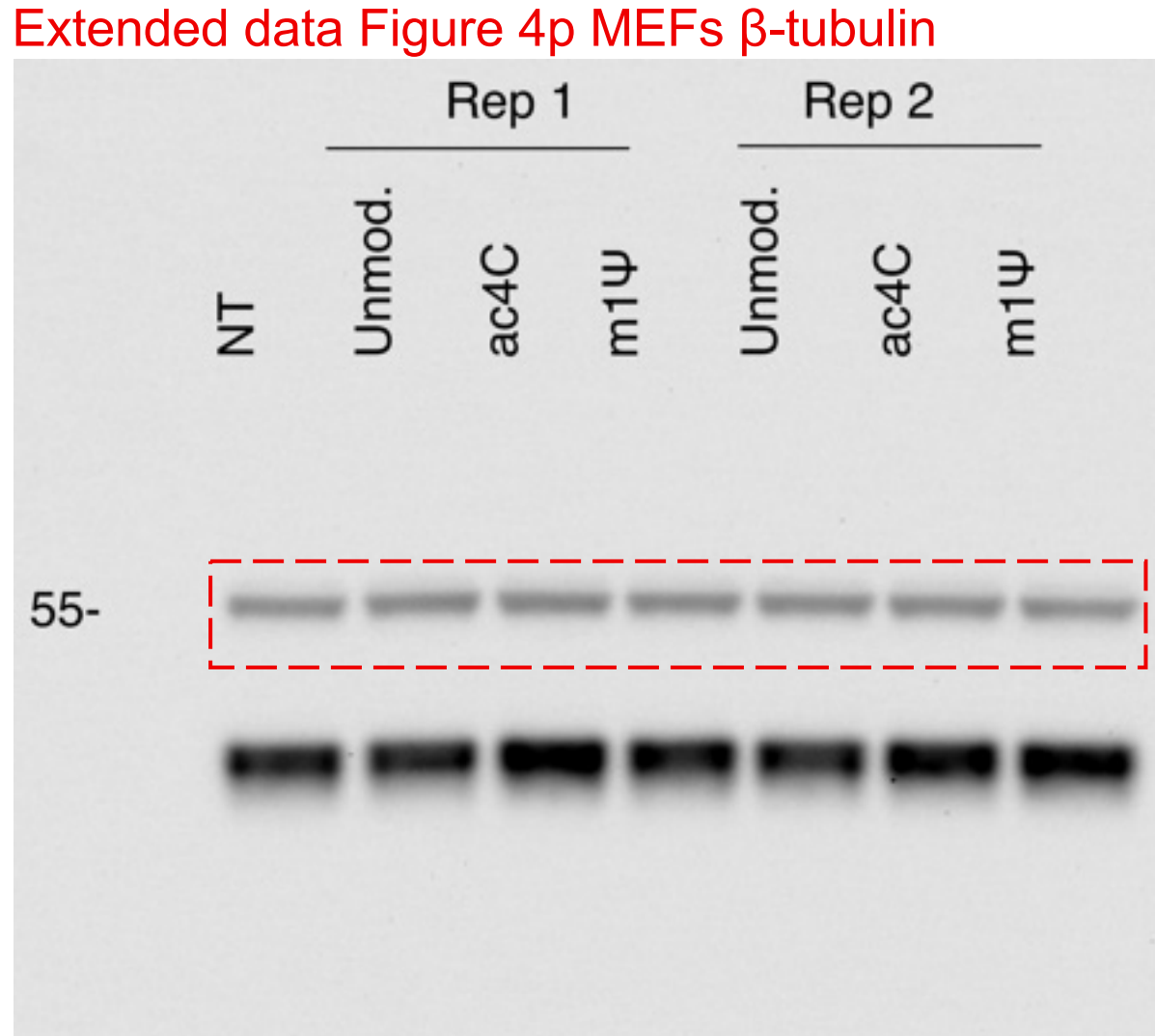

Extended Data Figure 5a Denaturing gel *SunTag* mRNA

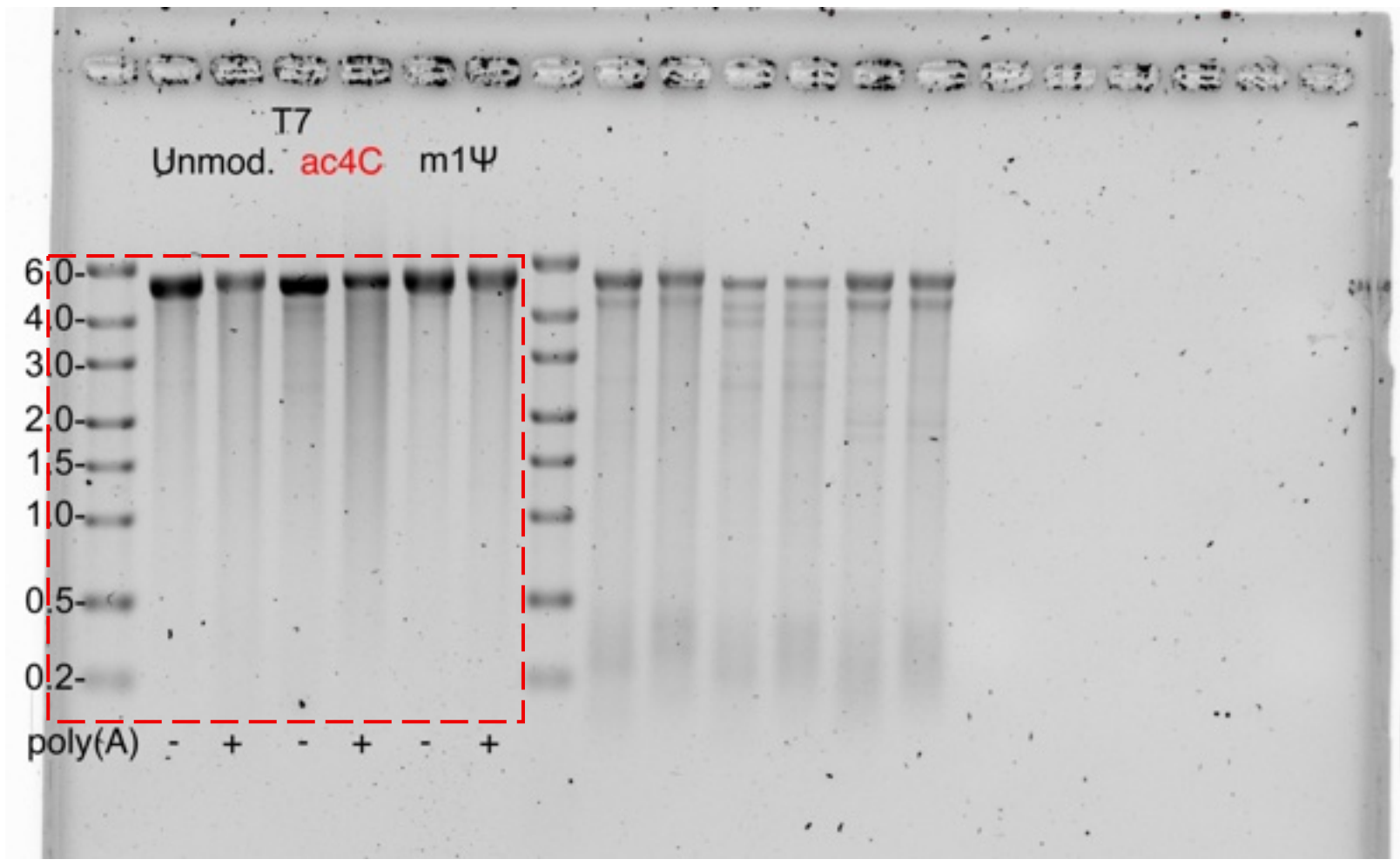

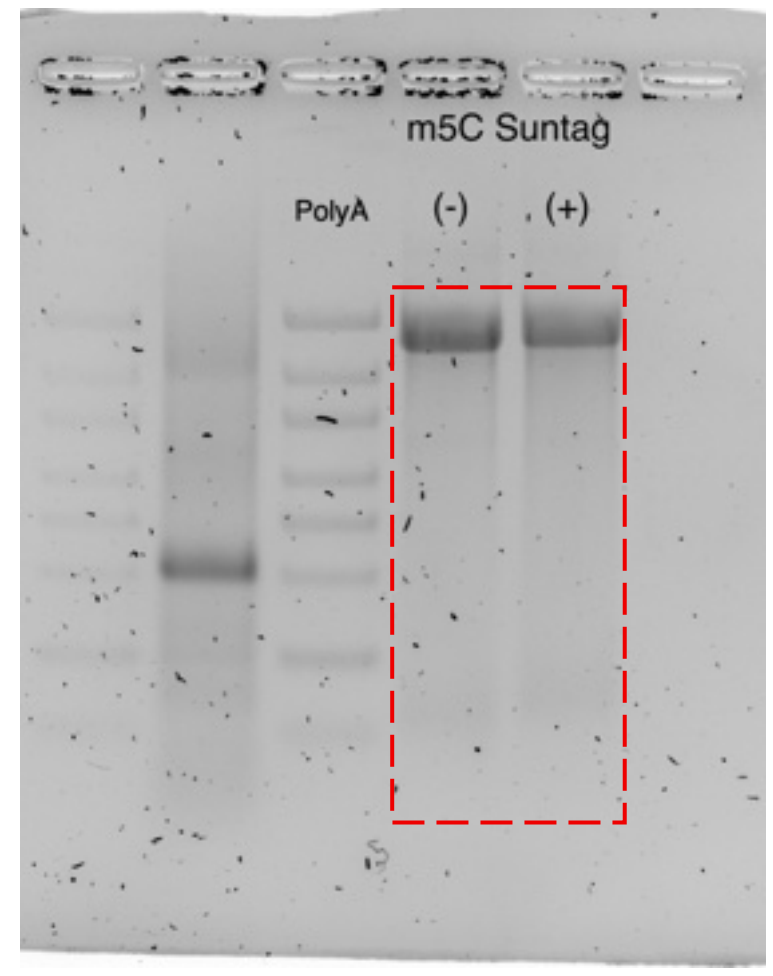

Extended Data Figure 5f U-2 OS p-eIF2-α

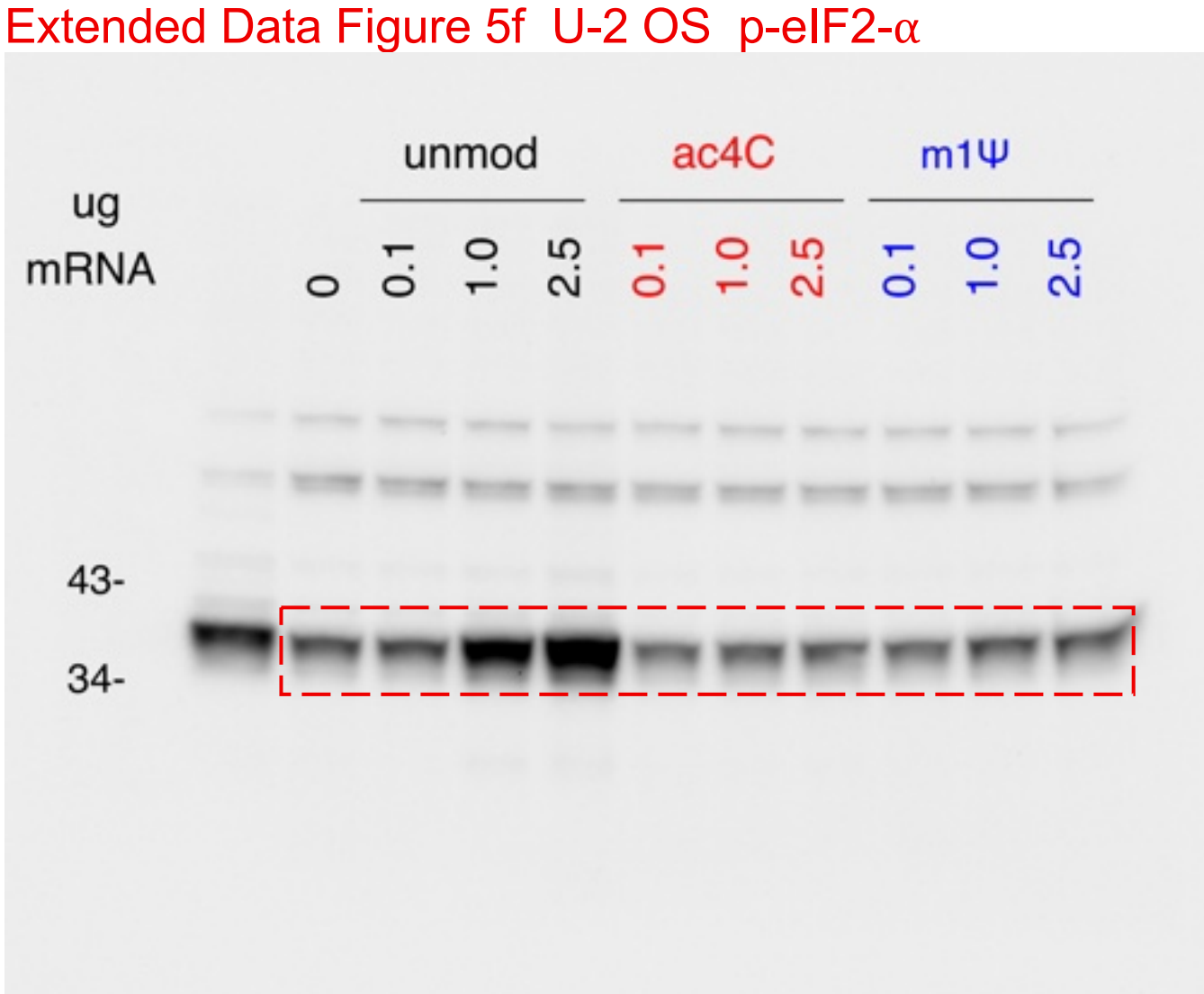

Extended Data Figure 5f U-2 OS eIF2-α

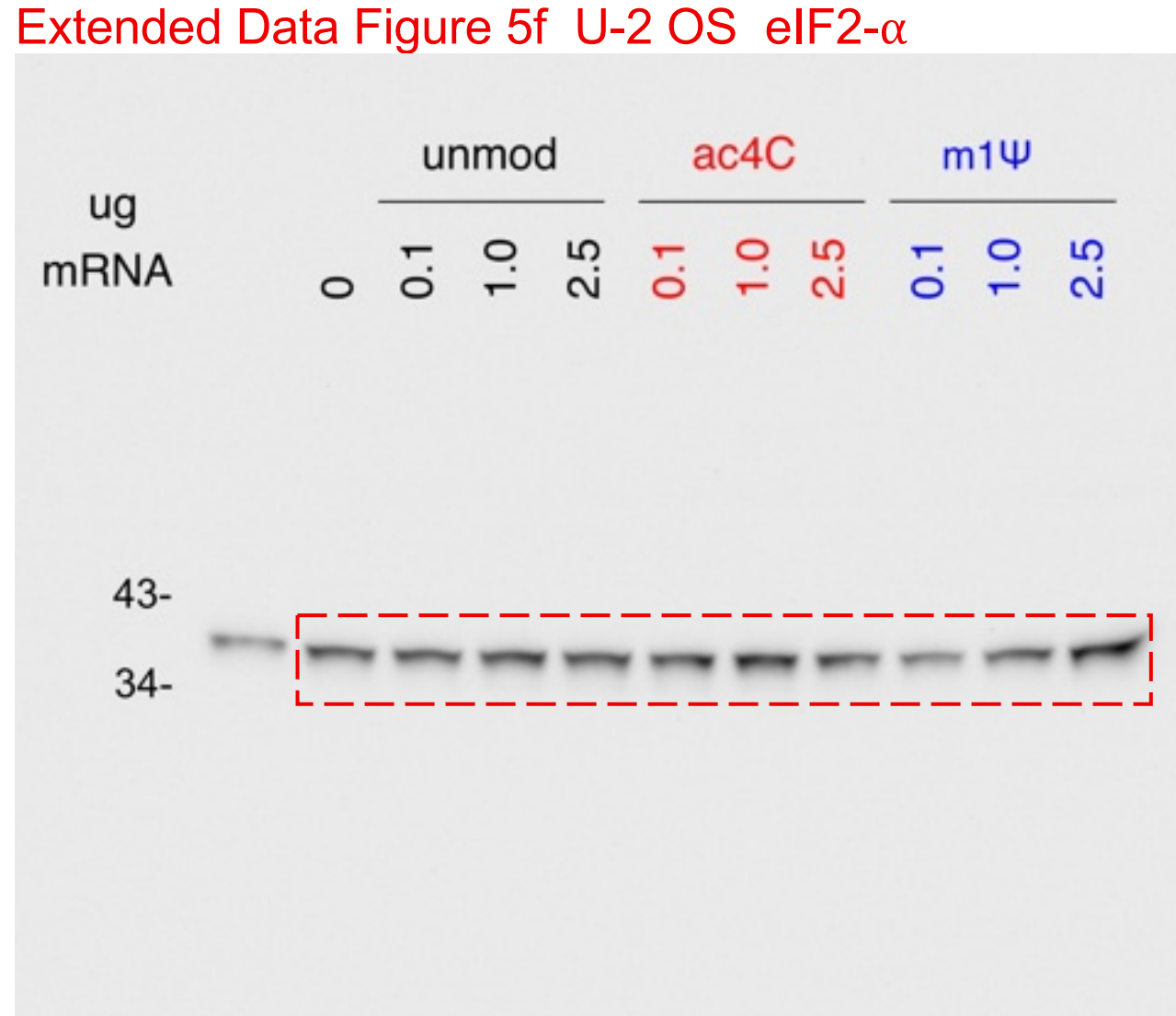

Extended Data Figure 5f U-2 OS p-eIF2-α

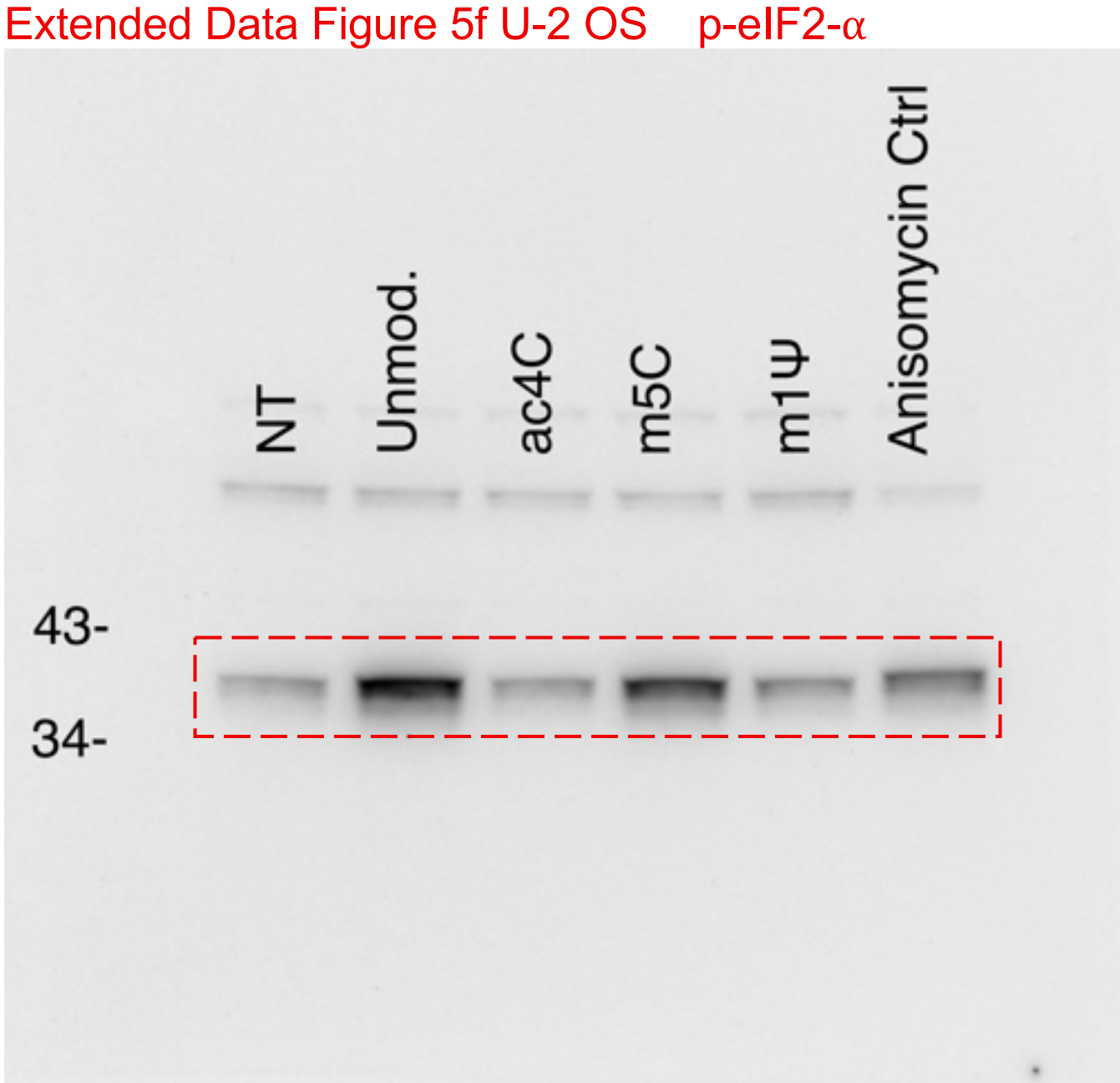

Extended Data Figure 5f U-2 OS eIF2-α

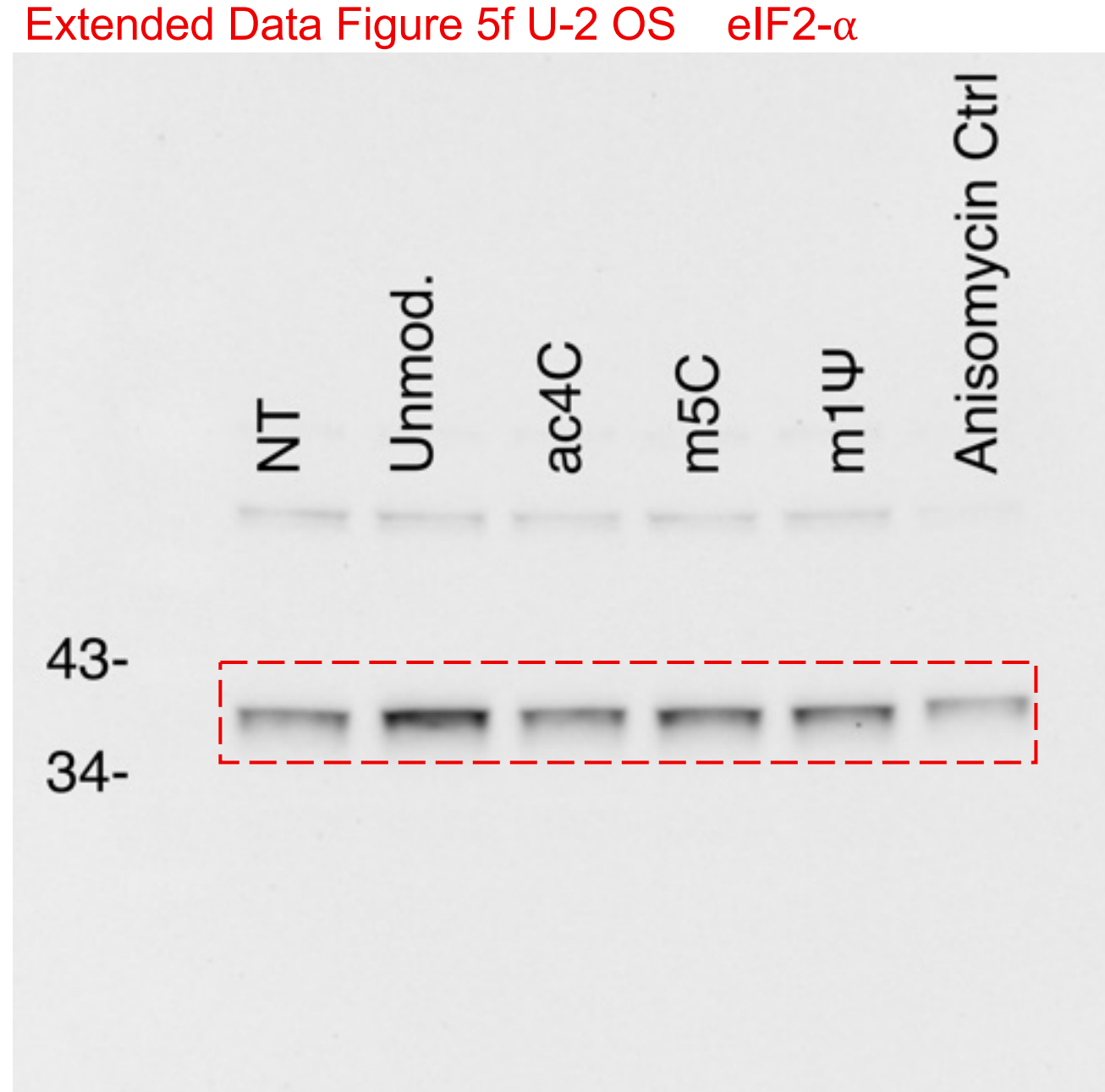

Extended Data Figure 5f U-2 OS β-tubulin

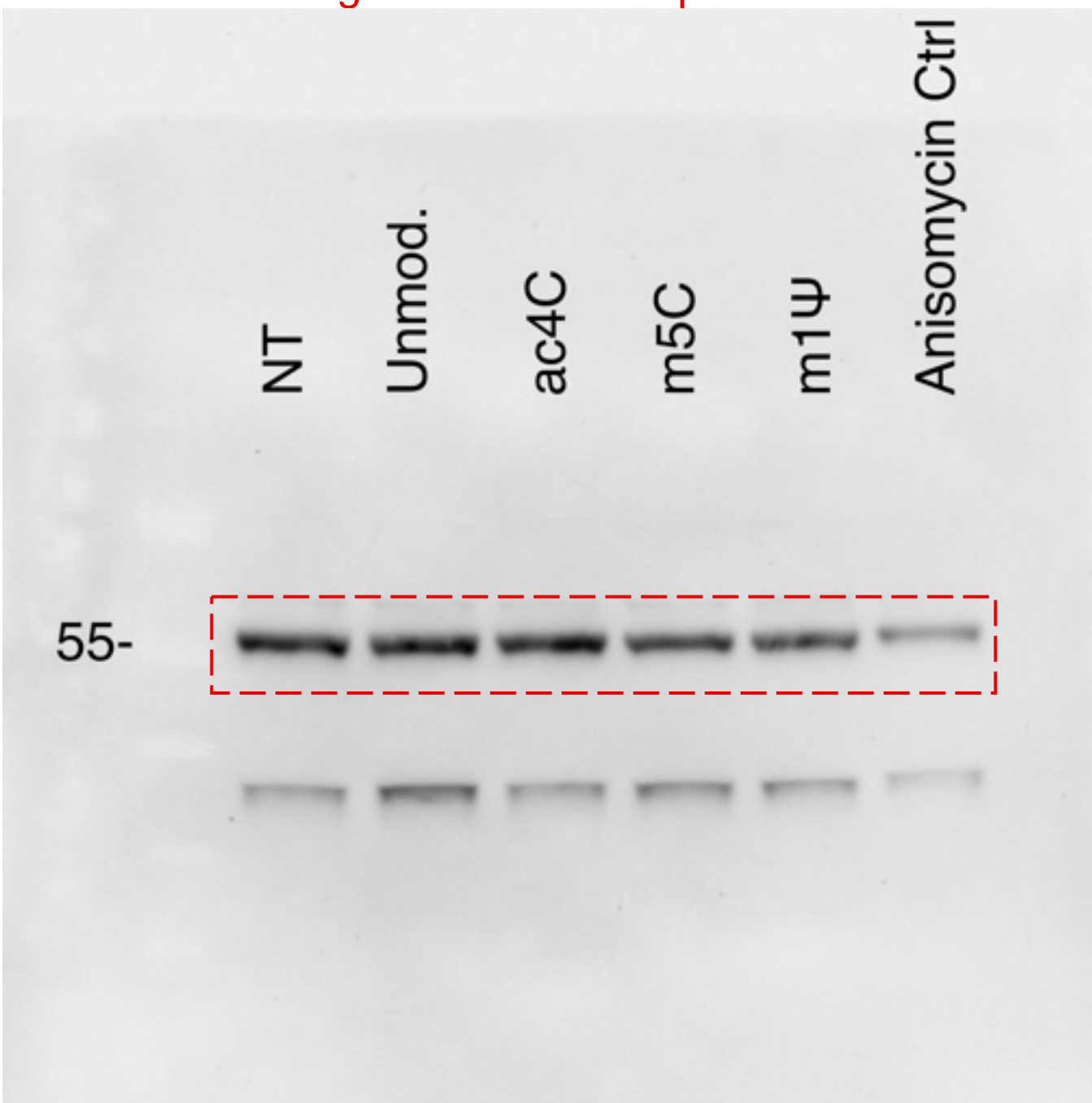

Extended Data Figure 6d U-2 OS ZNF598 KD – ZNF598

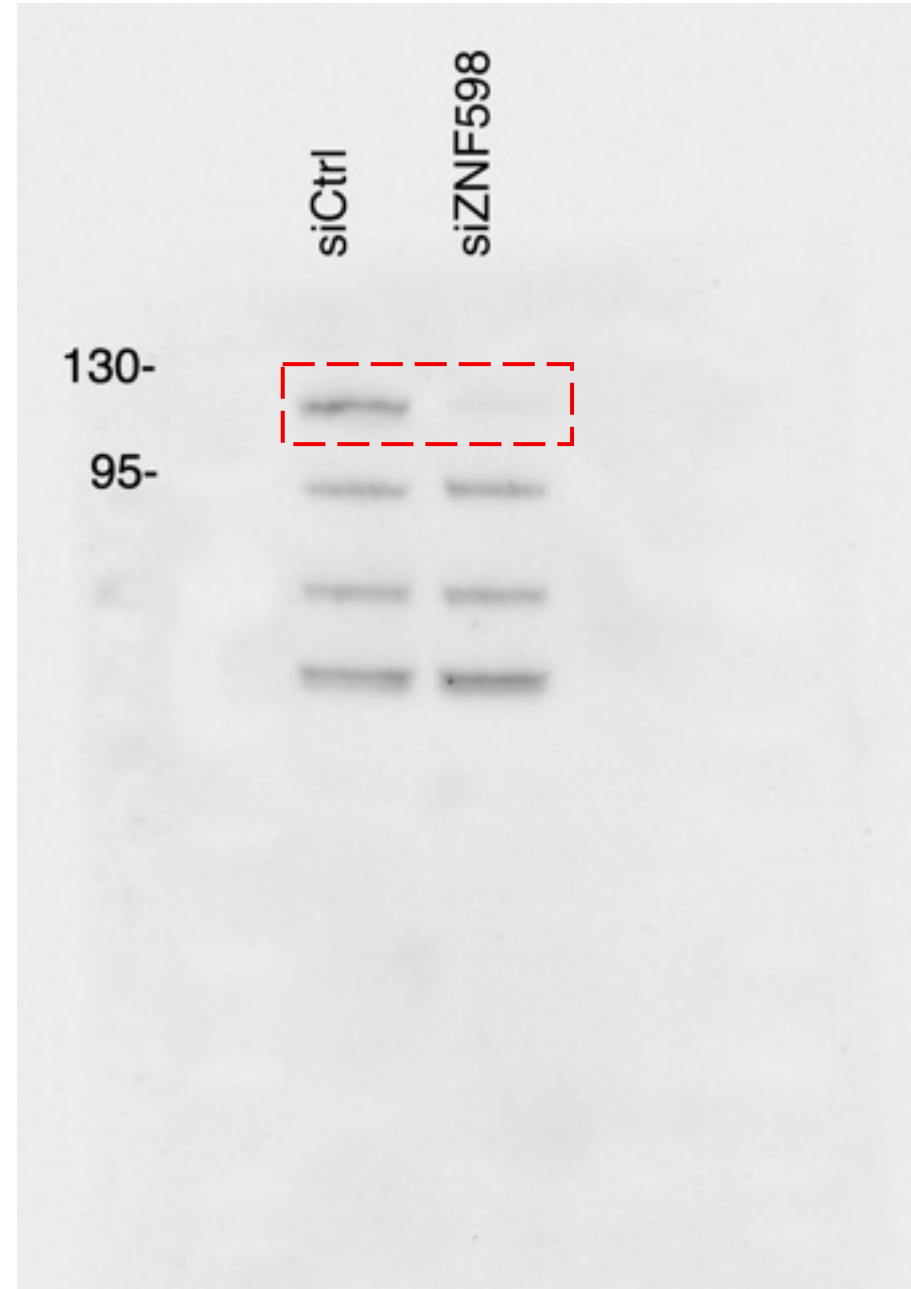

Extended Data Figure 6d U-2 OS ZNF598 KD - GAPDH

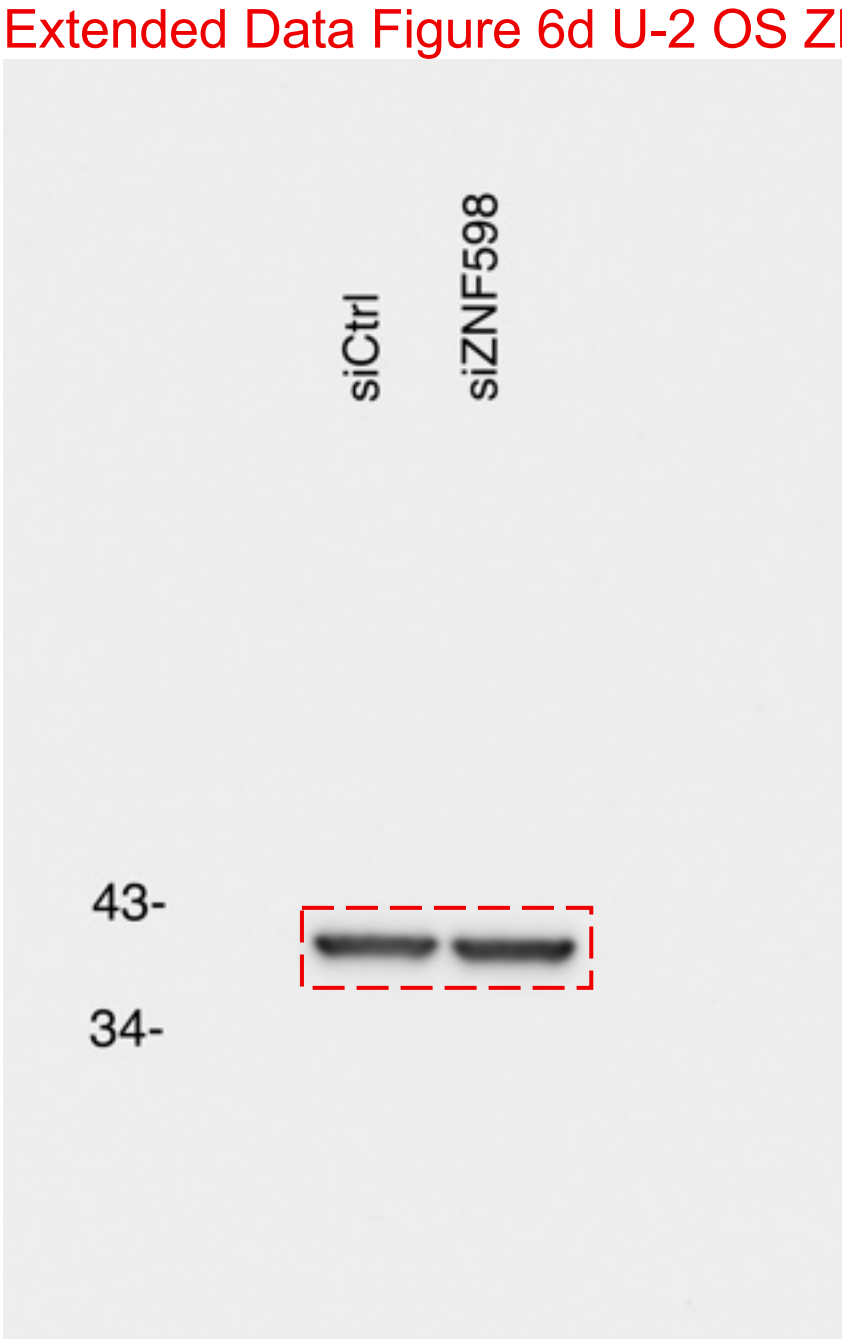

Extended Data Figure 6e U-2 OS p38-P

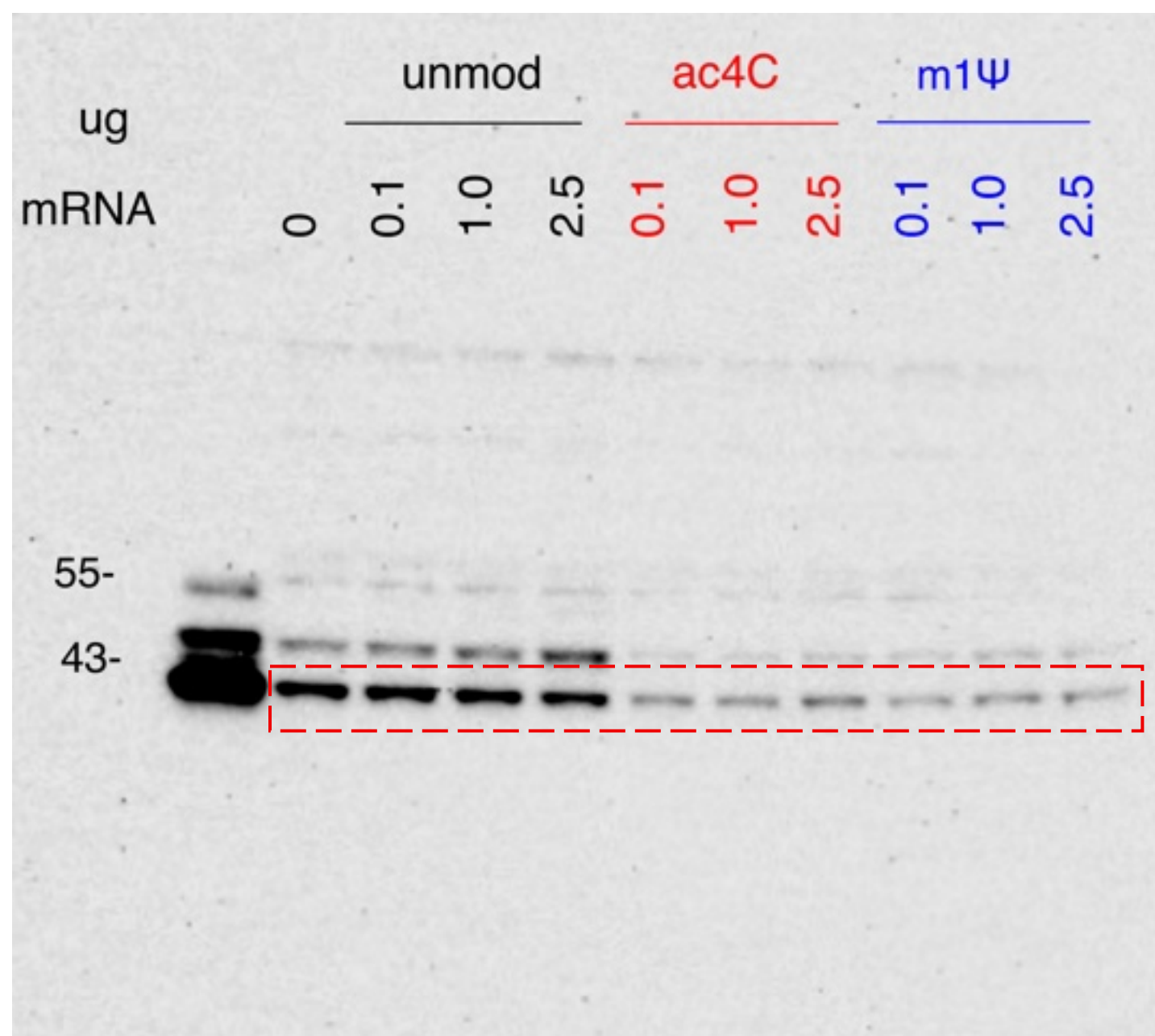

Extended Data Figure 6e U-2 OS p38

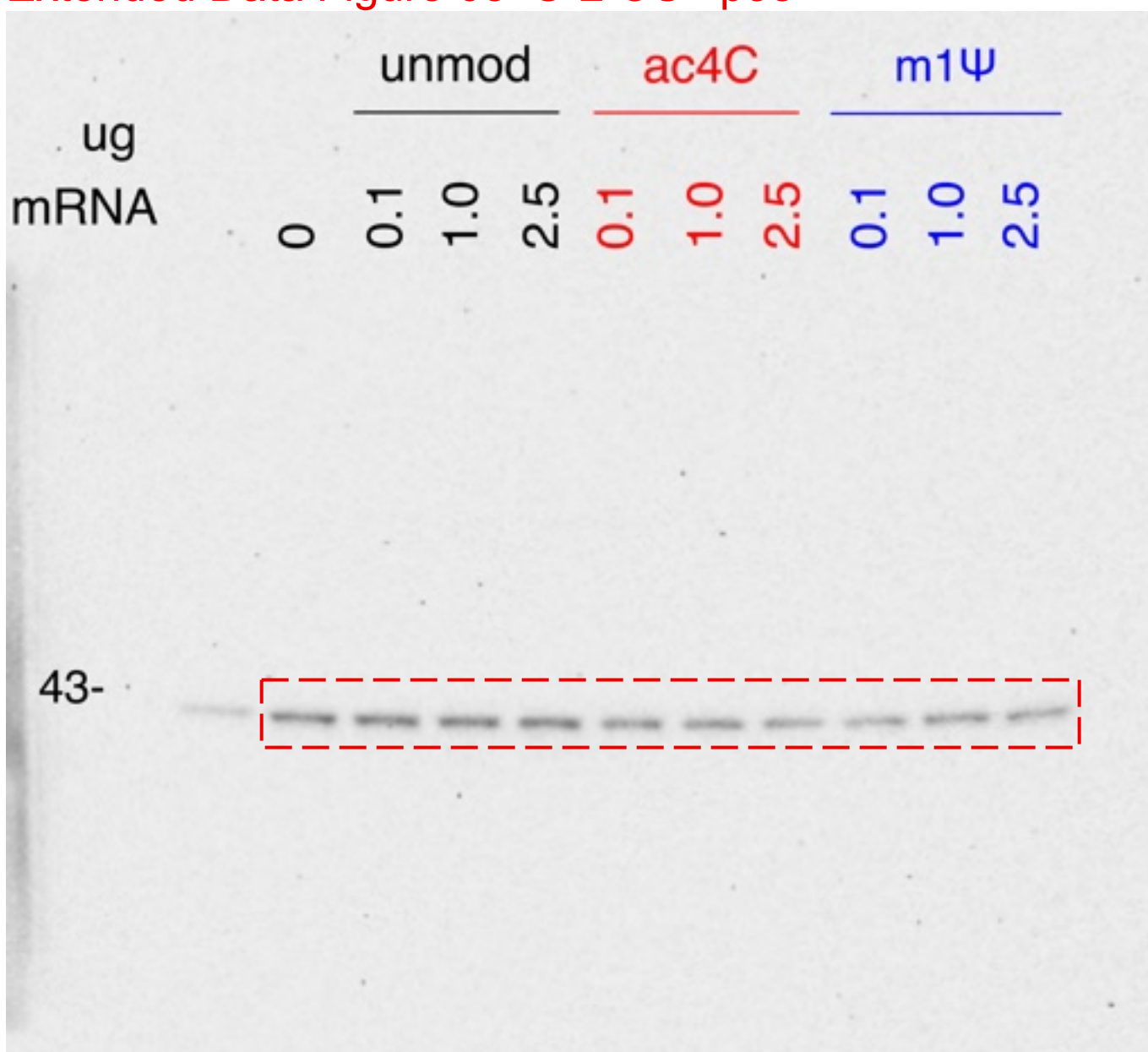

Extended Data Figure 6e U-2 OS JNK-P

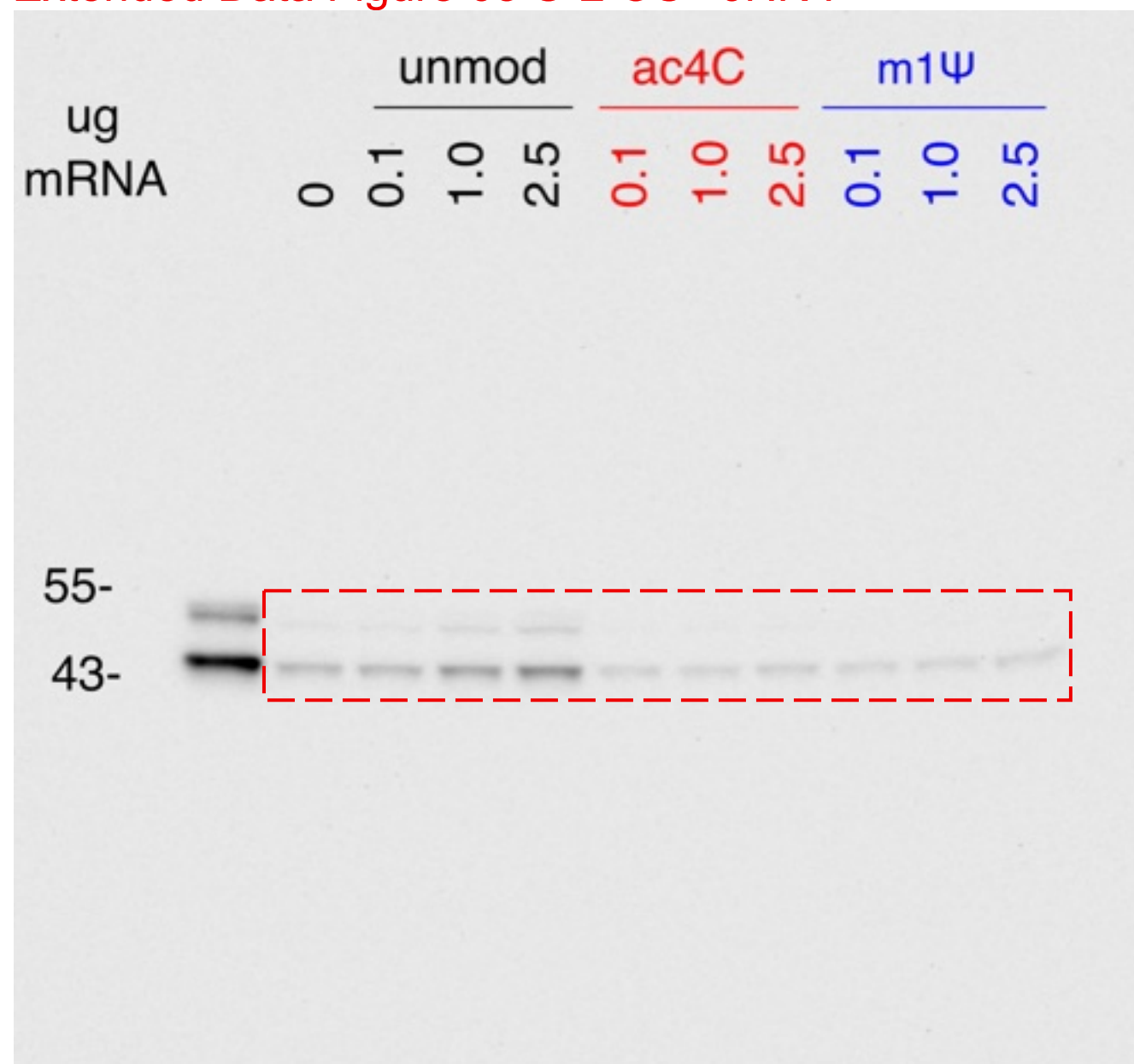

Extended Data Figure 6e U-2 OS JNK

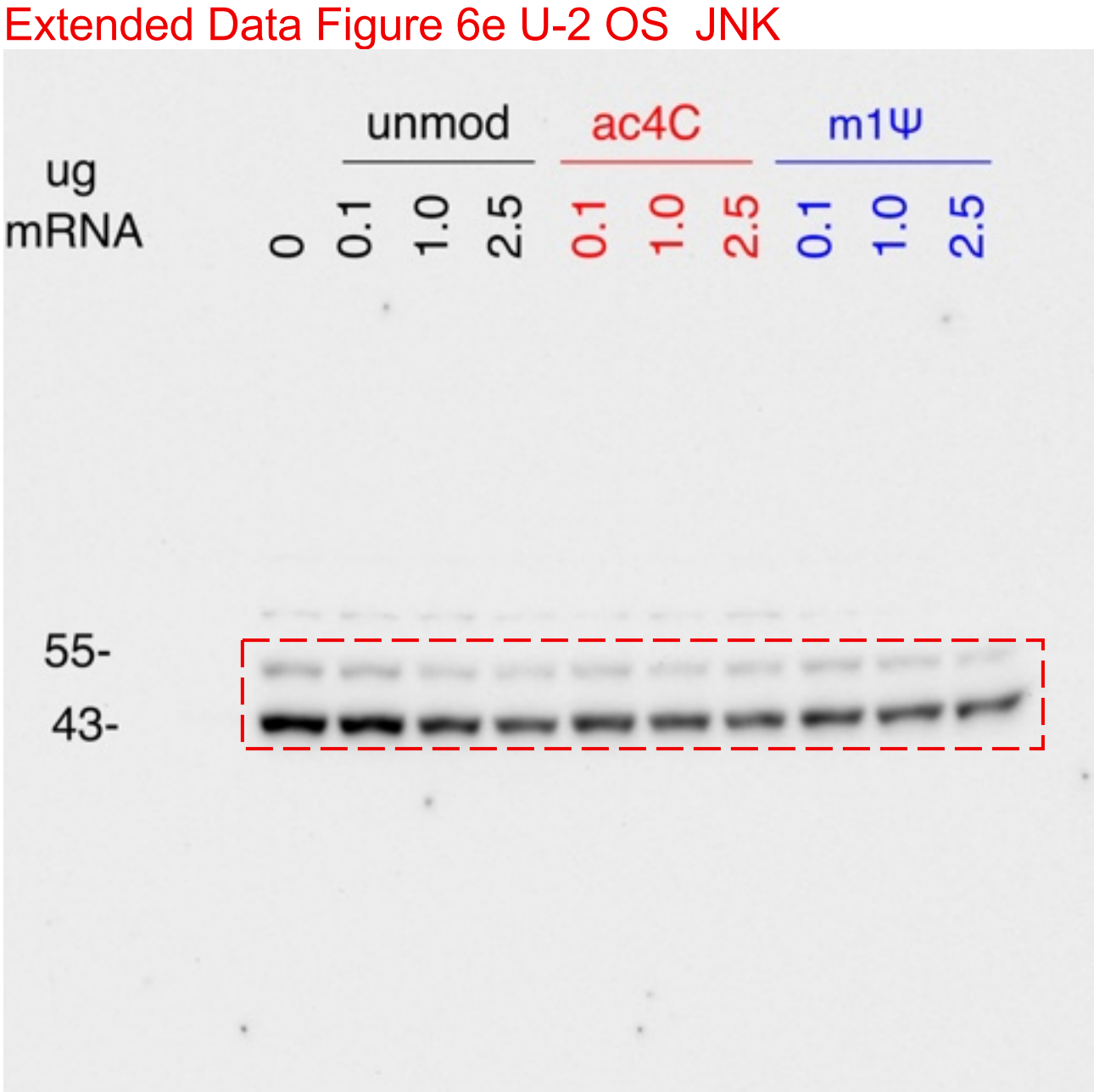

Extended Data Figure 6e U-2 OS LMNB1

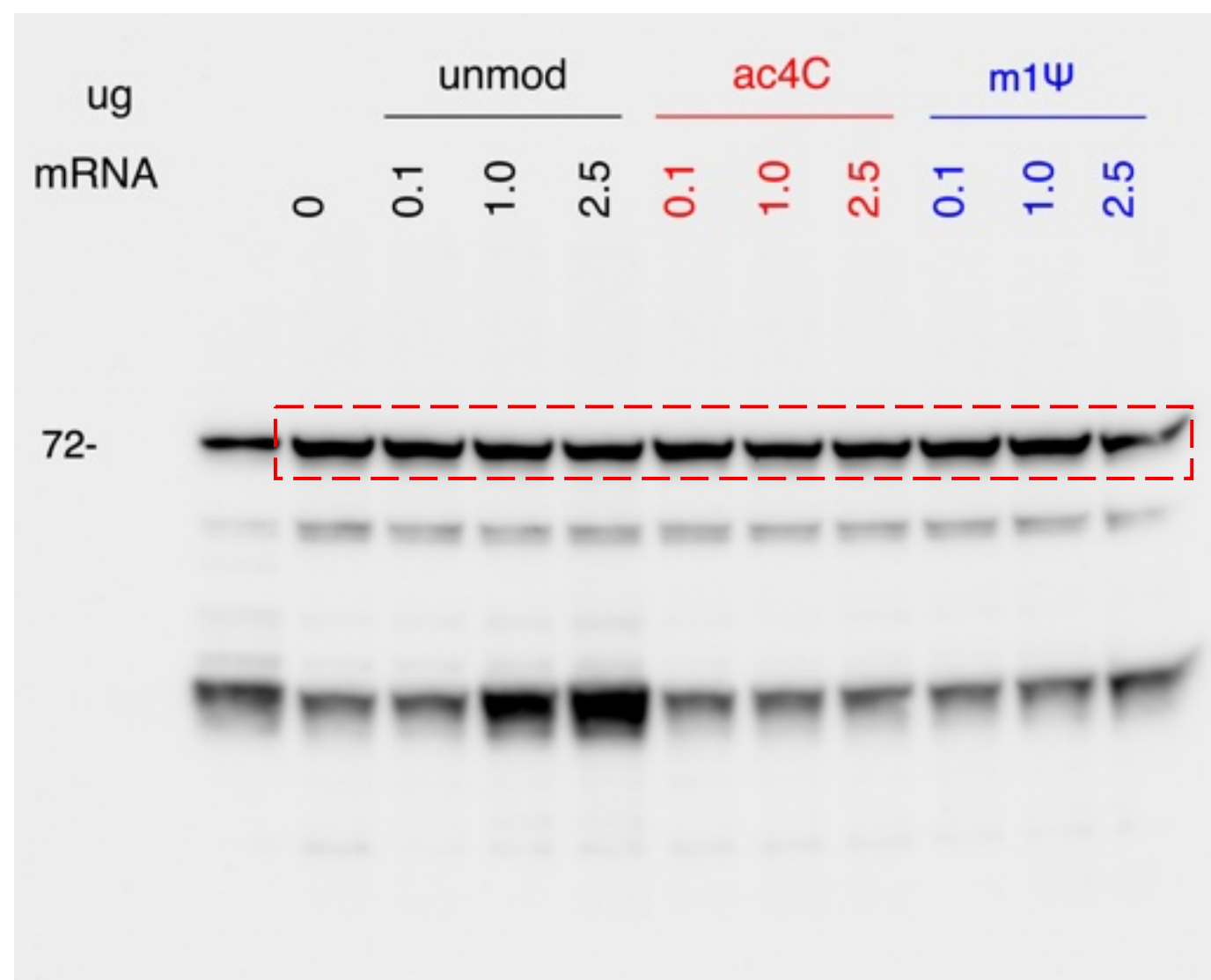

Extended Data Figure 6f p38-P

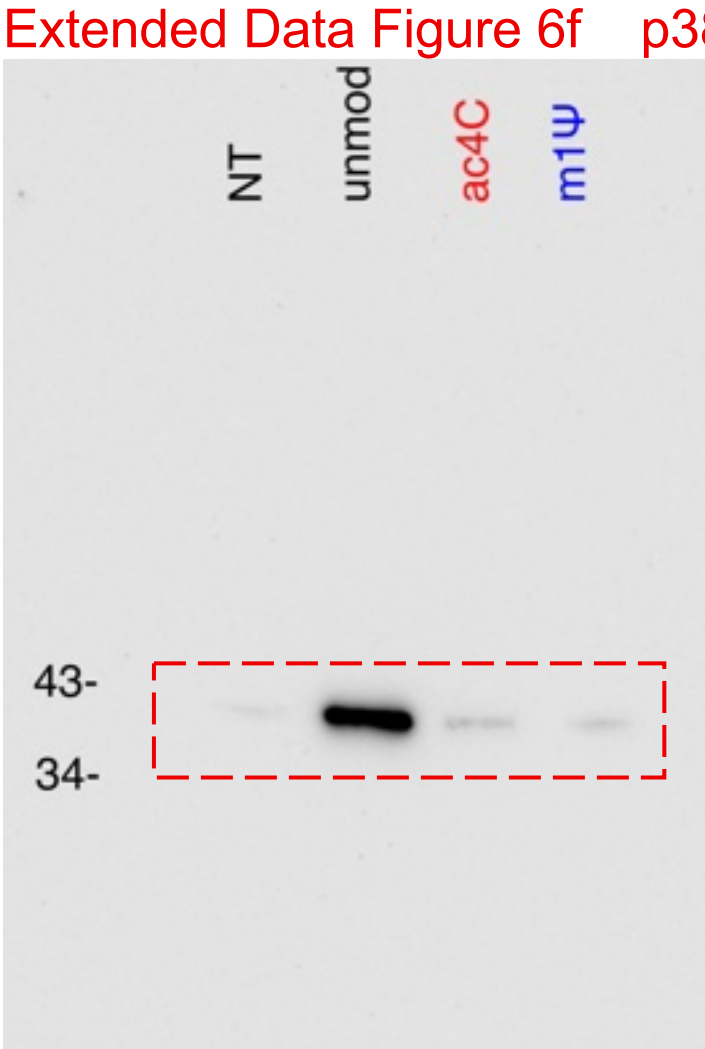

Extended Data Figure 6f p38-P

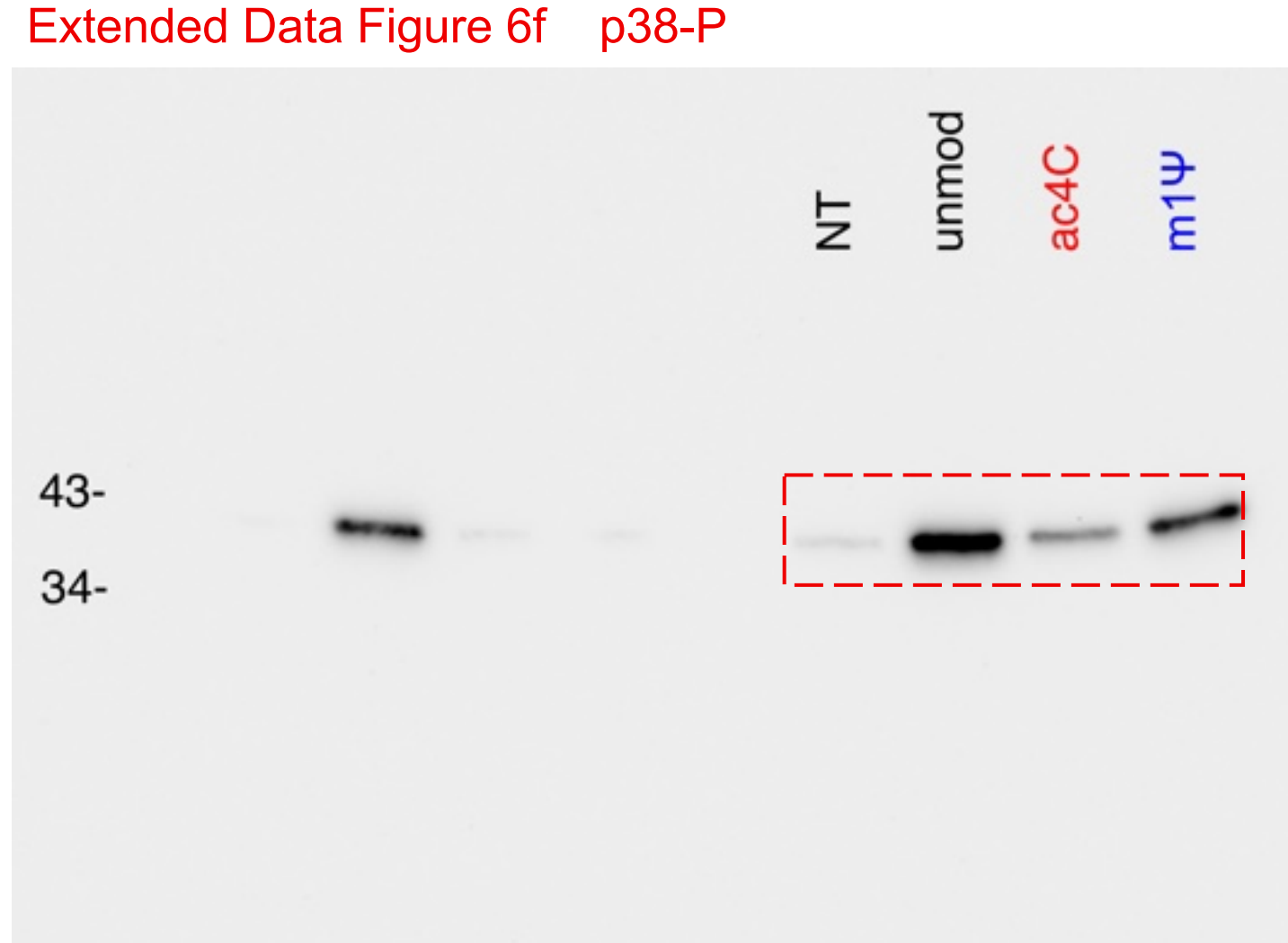

Extended Data Figure 6f p38

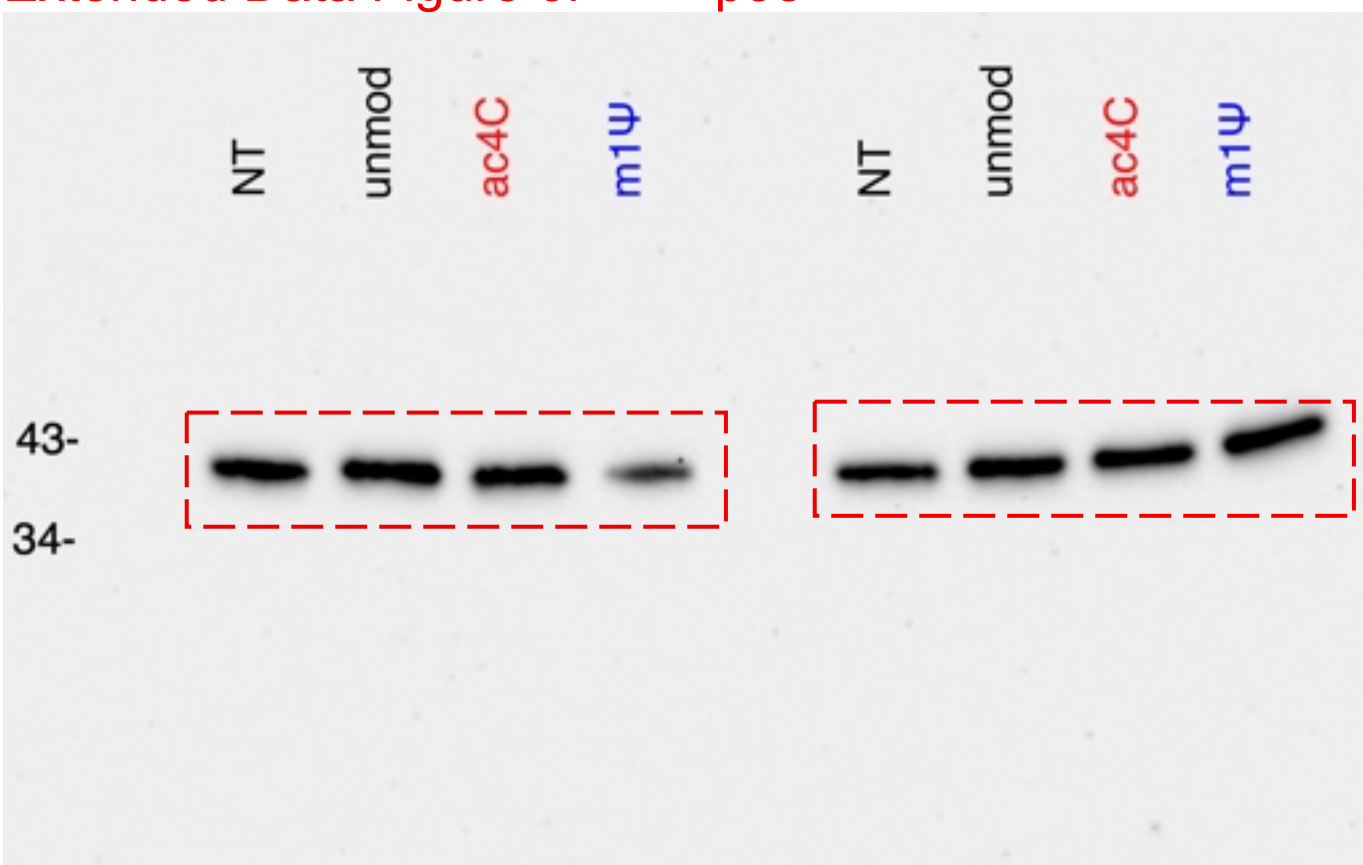

Extended Data Figure 6f JNK-P

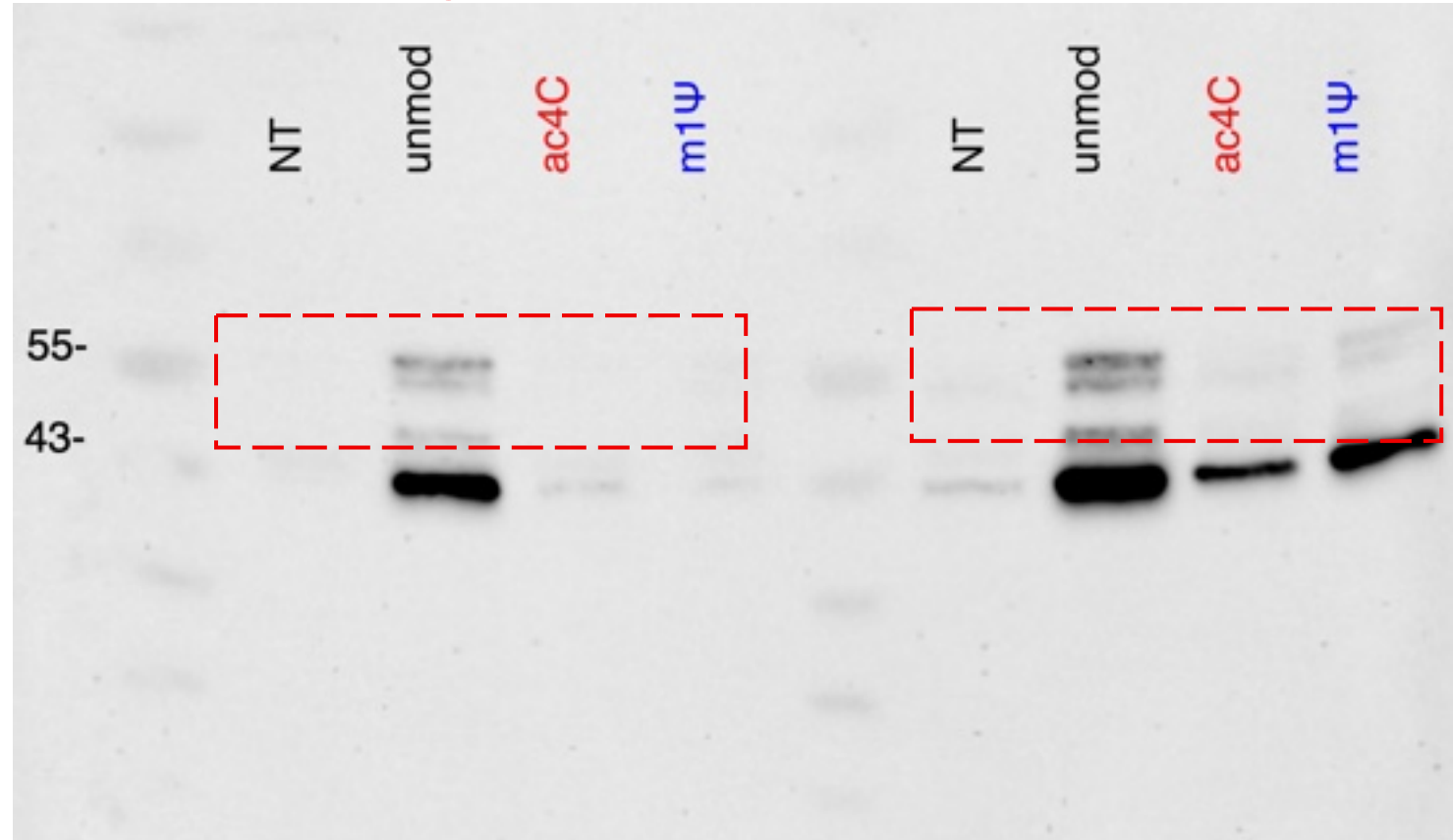

Extended Data Figure 6f JNK

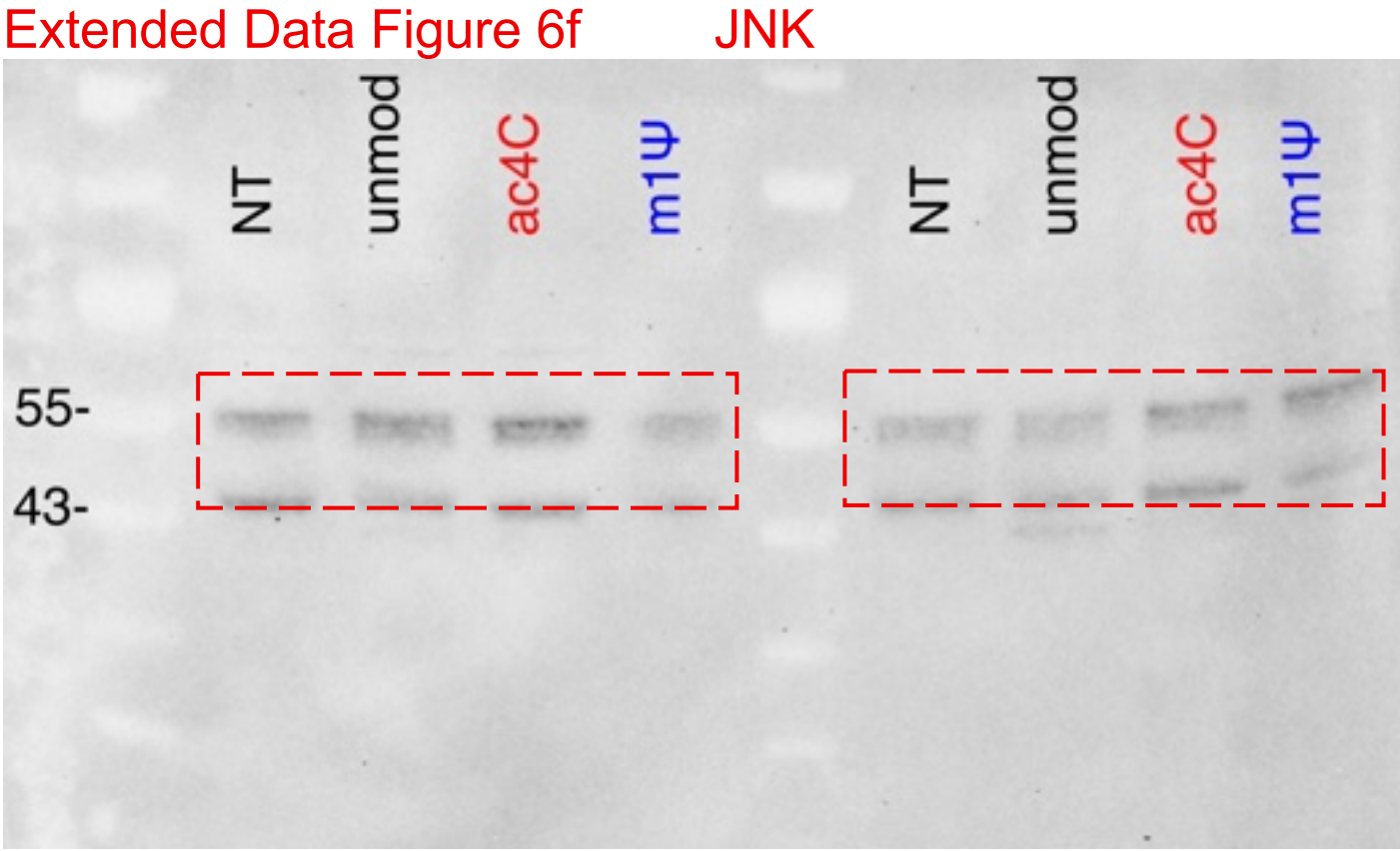

Extended Data Figure 6f GAPDH

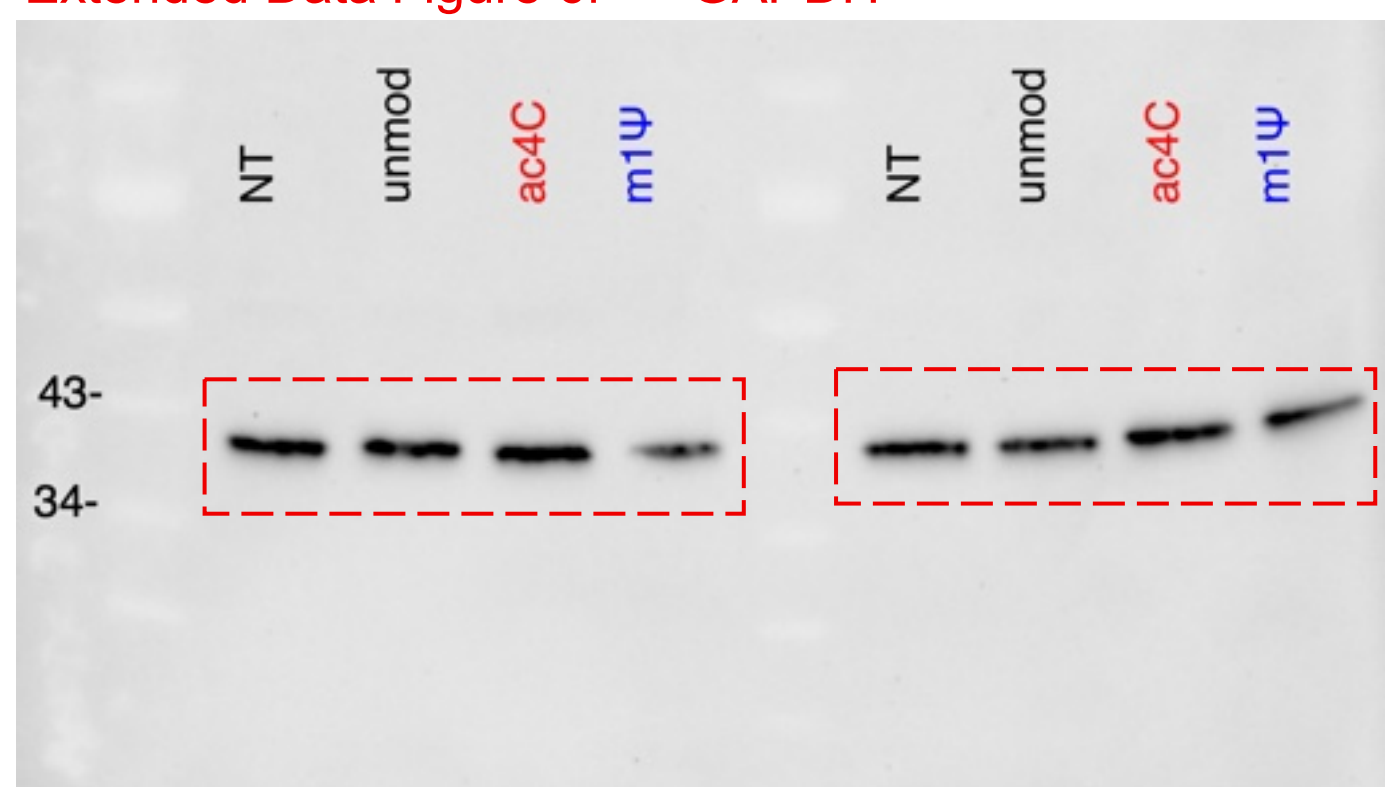

Fig.5b ^{35}S-Met labeled FLuc RRL

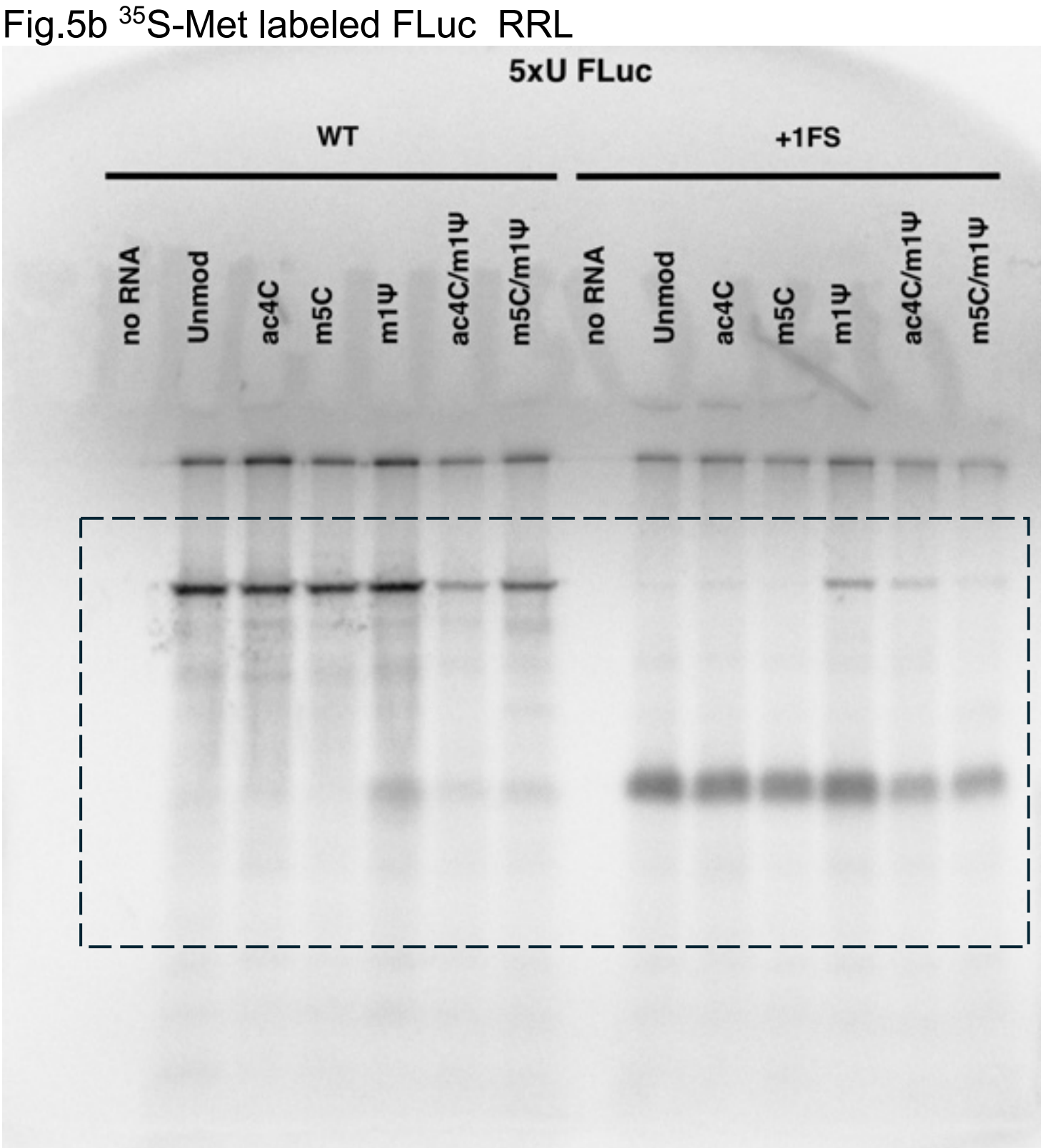

Fig.5b ^{35}S-Met labeled FLuc RRL

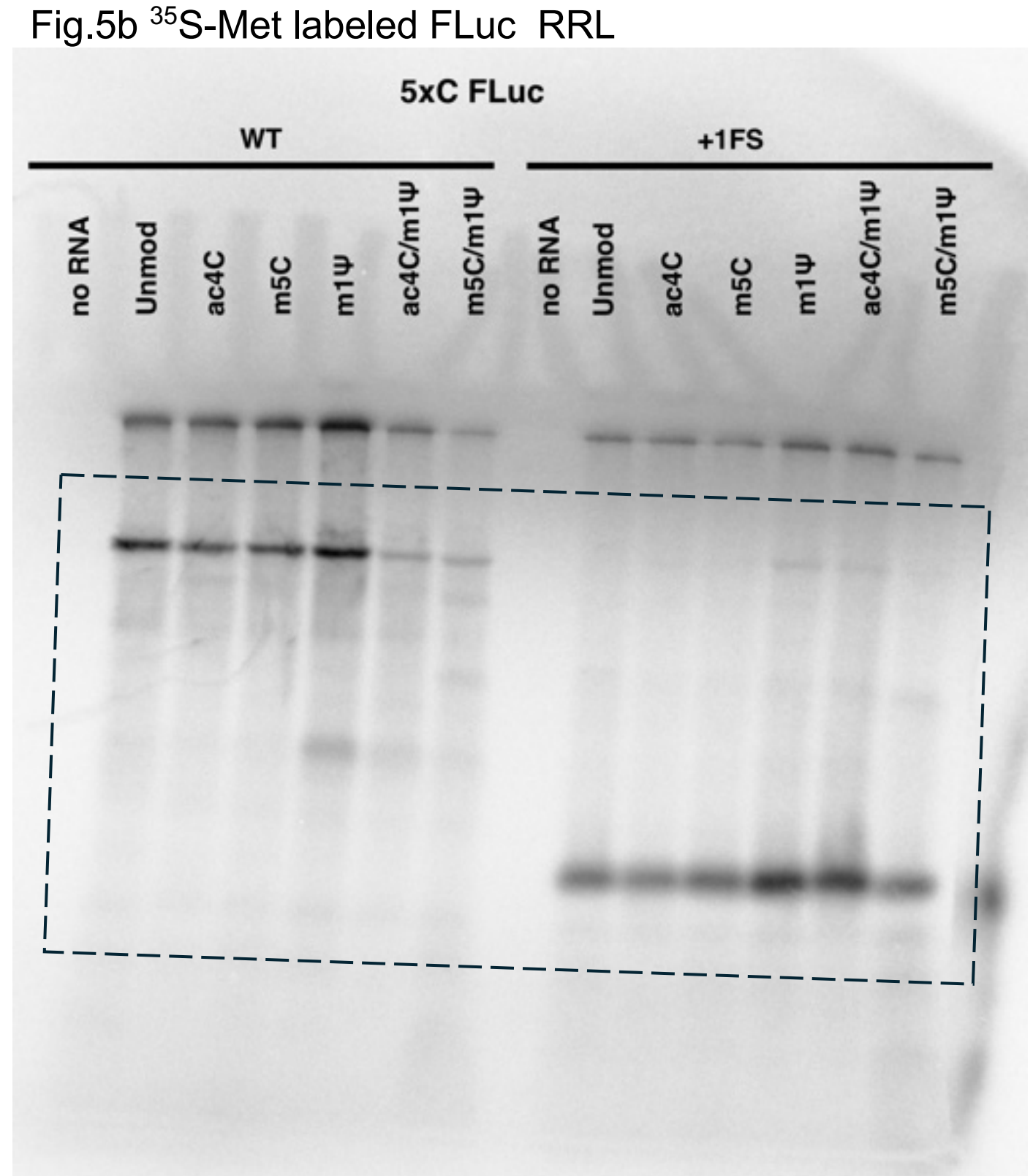

Fig.5f 5xU FLuc Flag Extended Data 7e

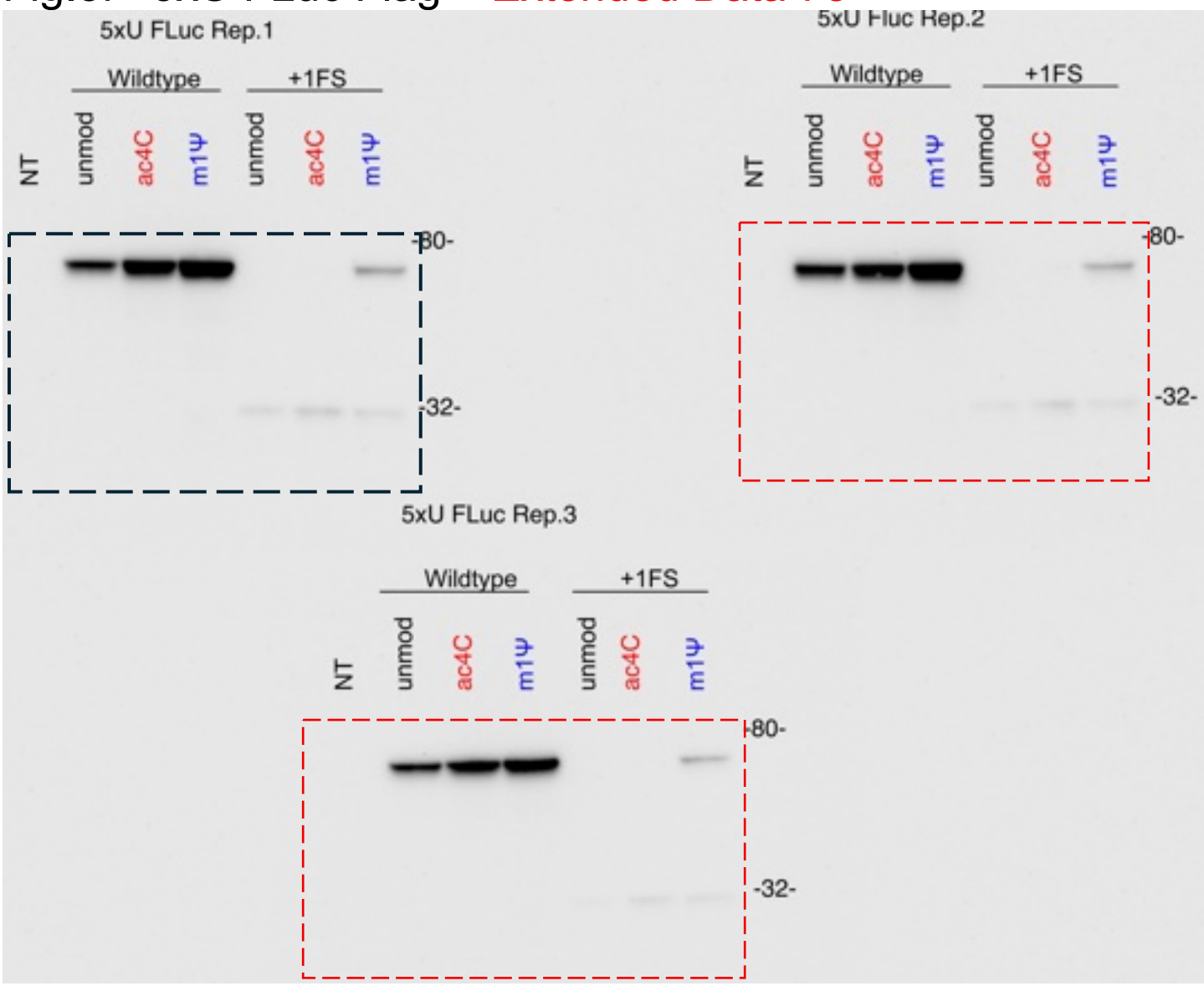

Fig.5f 5xU FLuc GAPDH Extended Data 7e

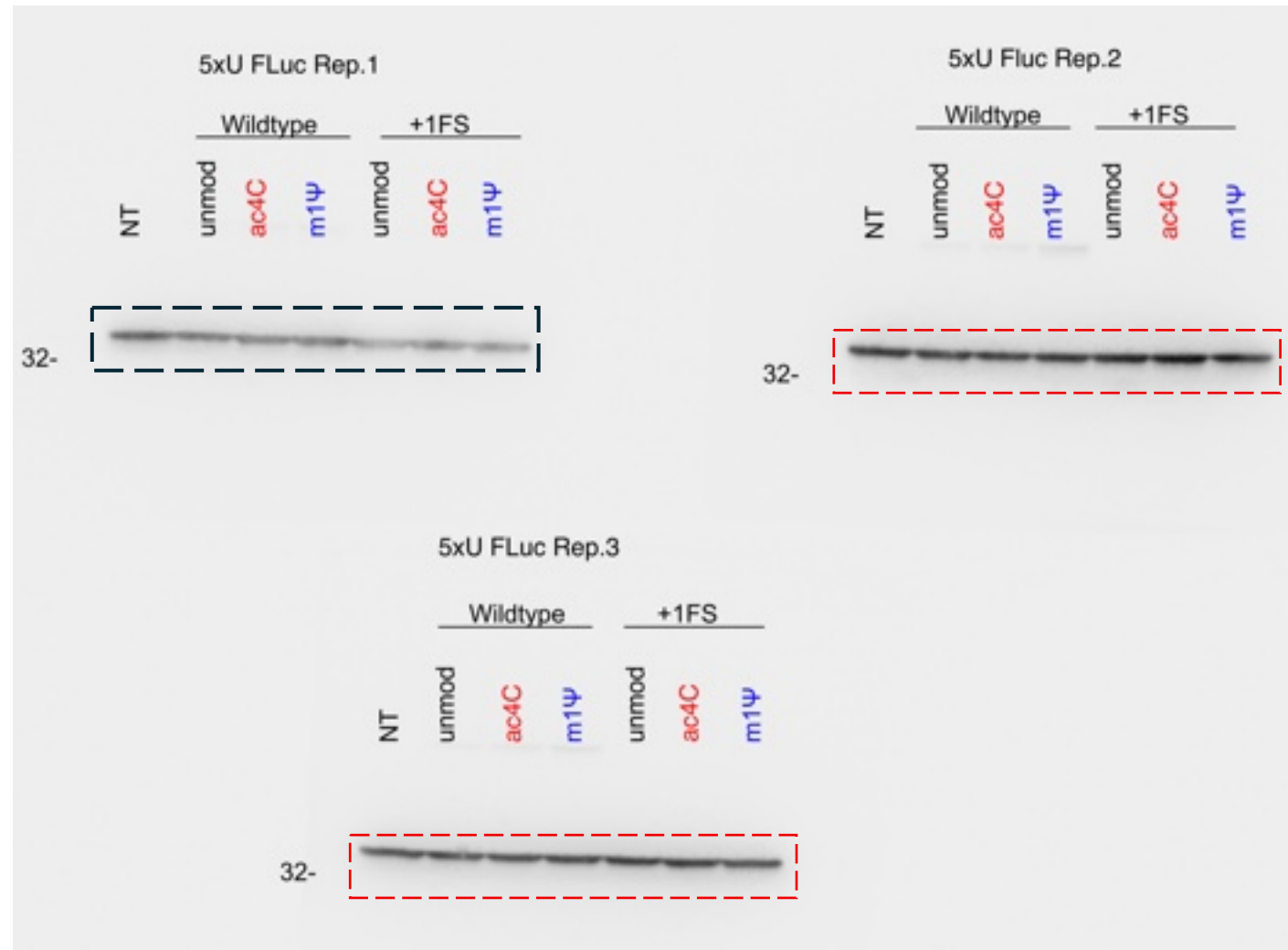

Fig.5f 5xC FLuc Flag

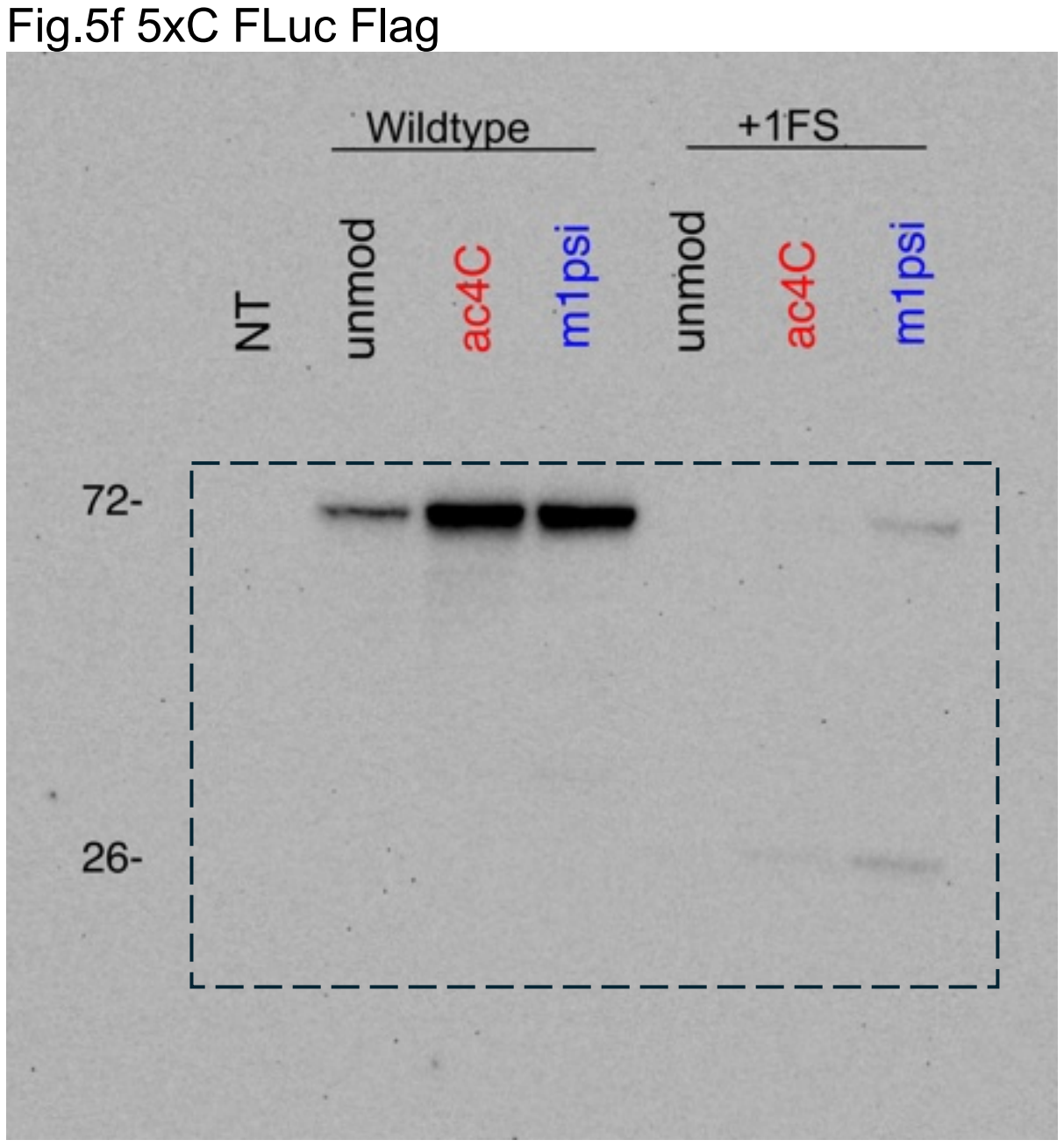

Fig.5f 5xC FLuc GAPDH

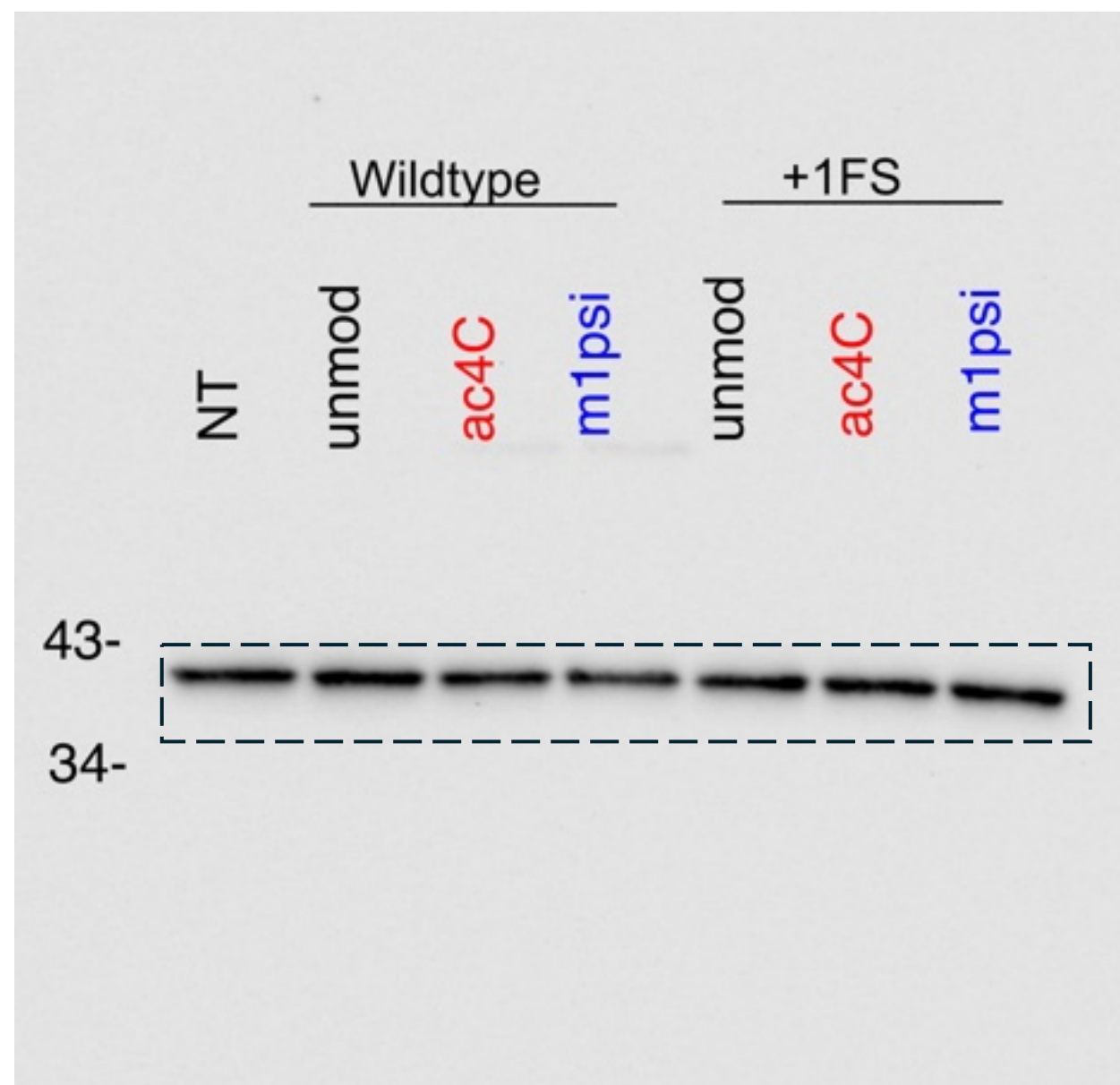

Extended Data Figure 7a

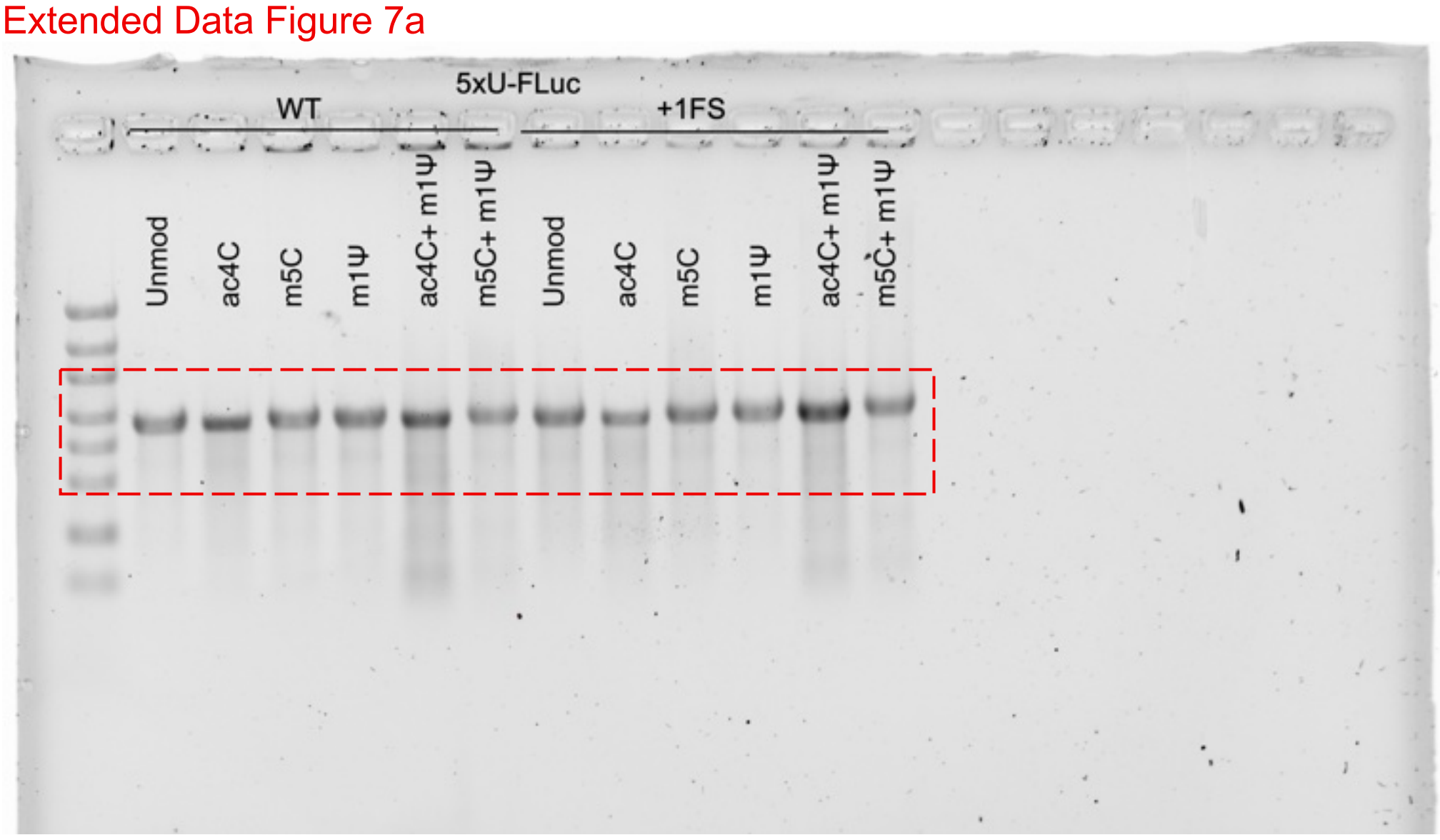

Extended Data Figure 7a

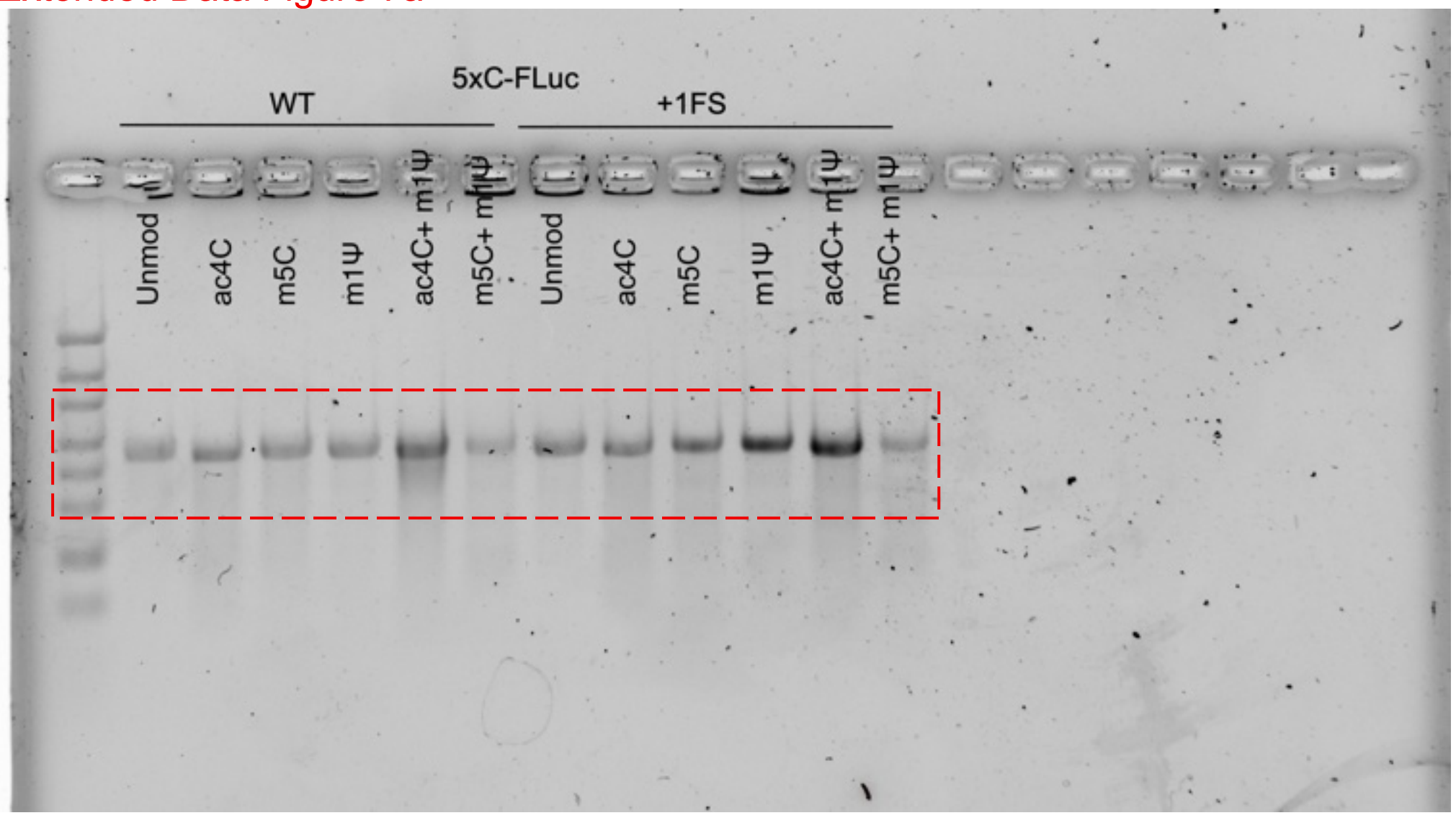

Extended Data Figure 7b ^{35}S-Met labeled FLuc RRL

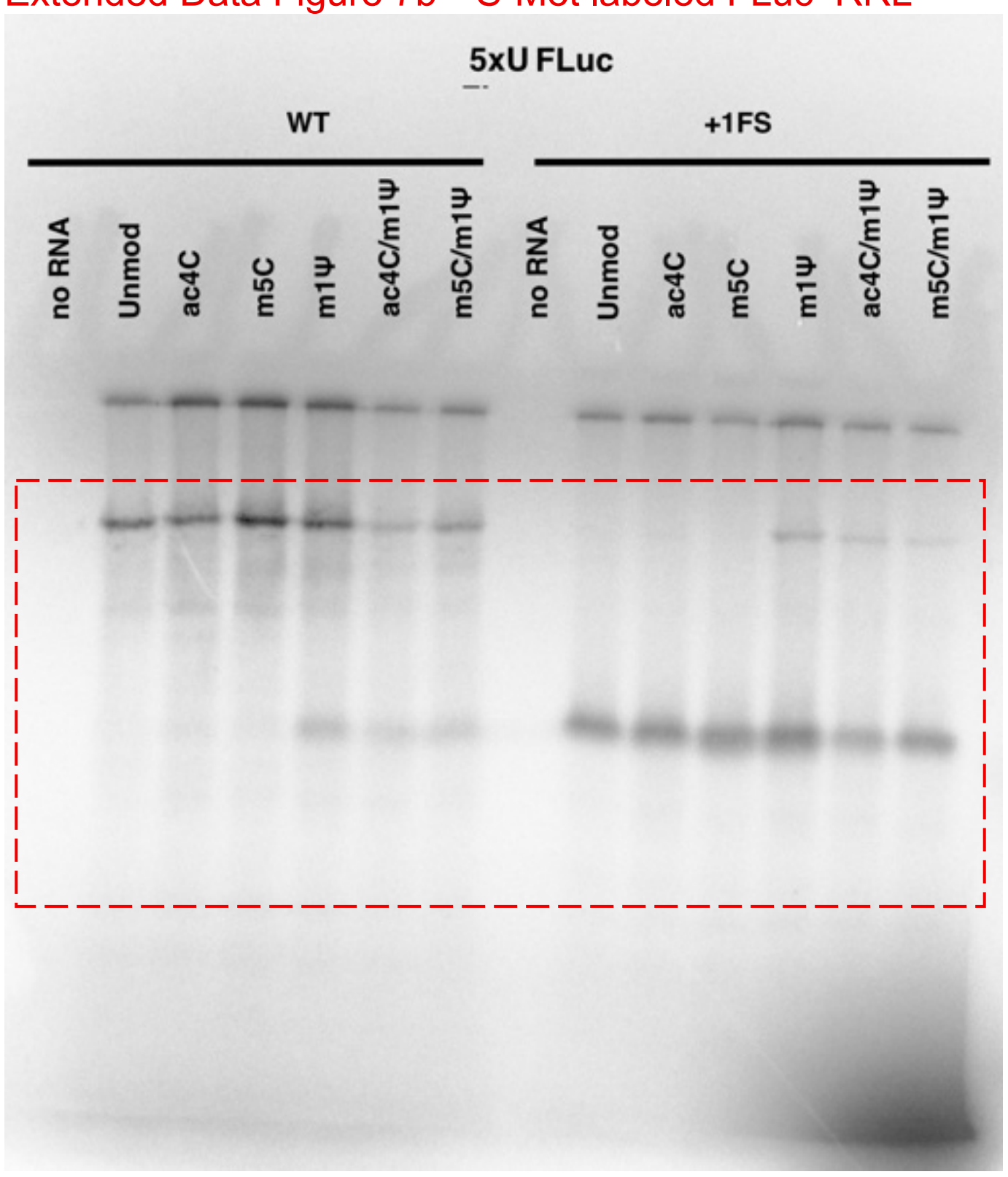

Extended Data Figure 7b ^{35}S-Met labeled FLuc RRL

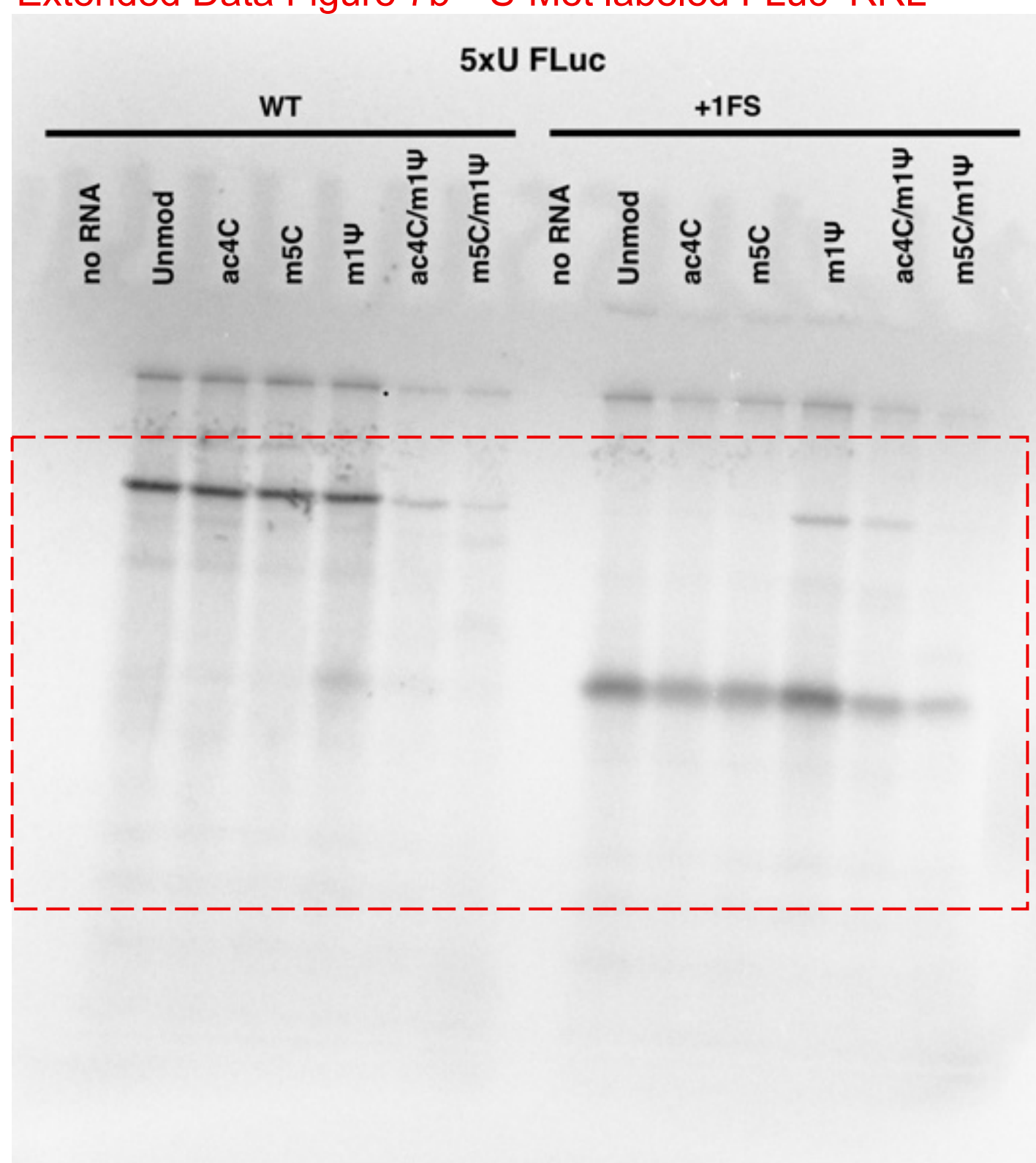

Extended Data Figure 7b ^{35}S-Met labeled FLuc RRL

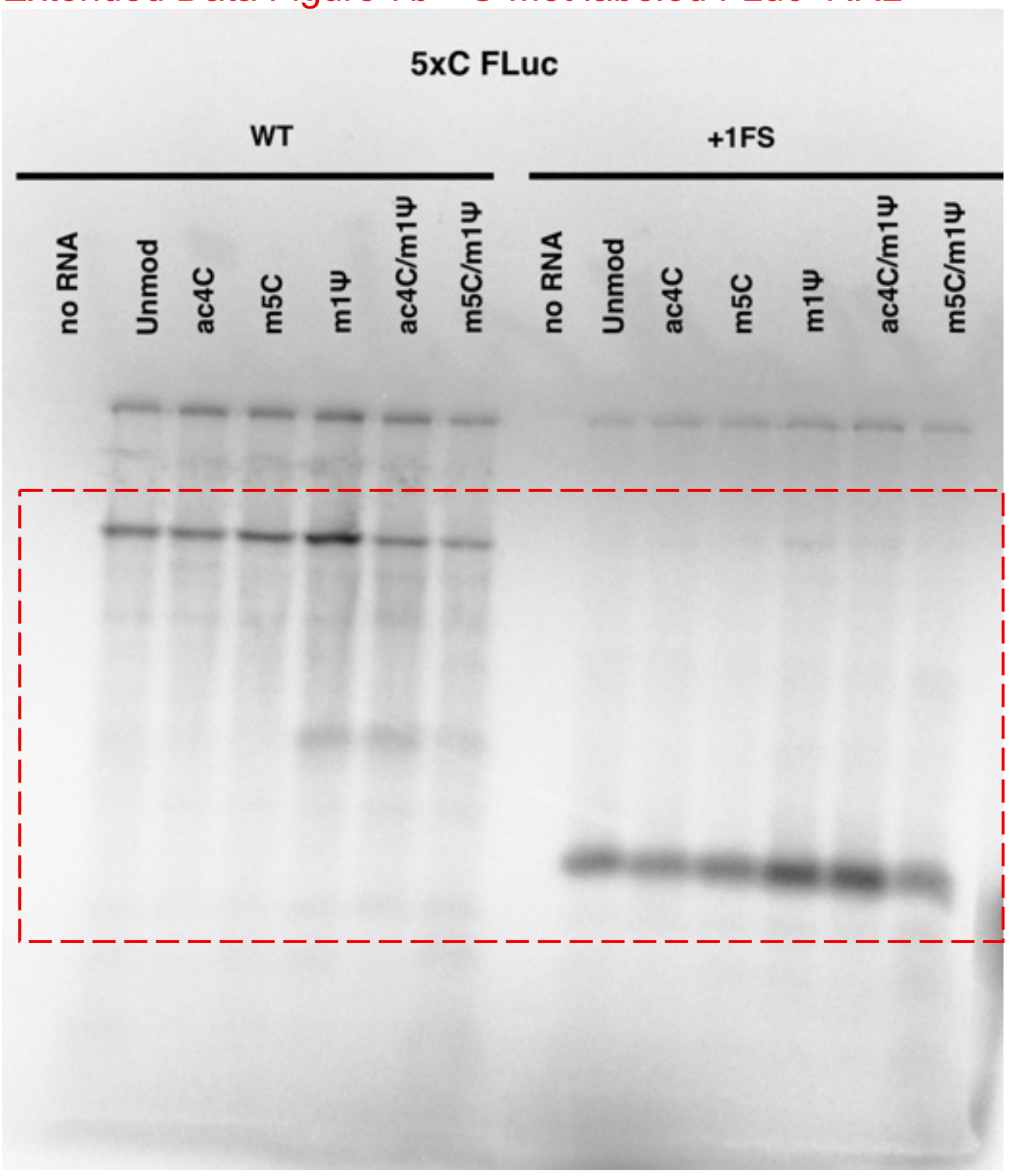

Extended Data Figure 7b ^{35}S-Met labeled FLuc RRL

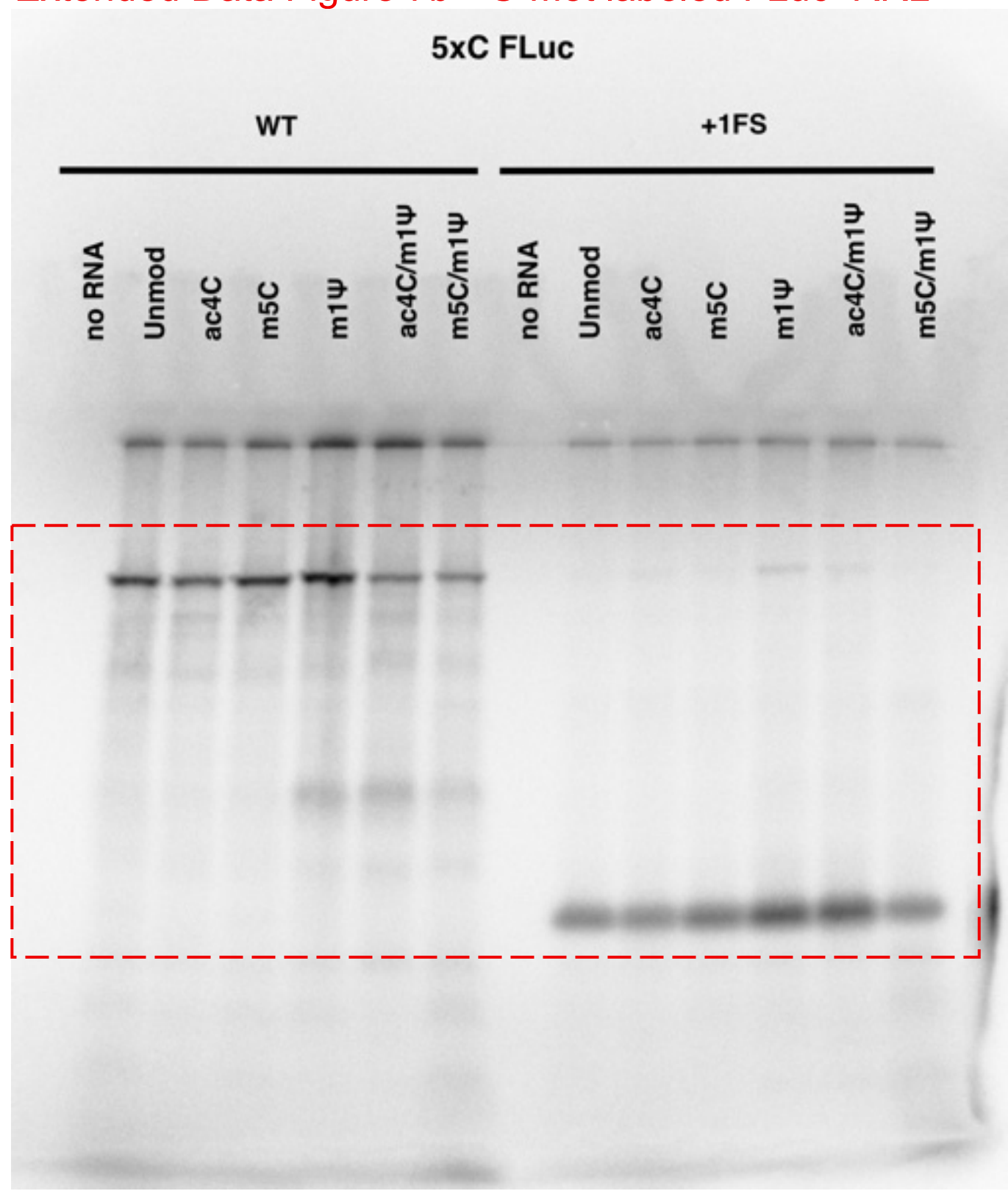

Extended Data Figure 7e 5xC FLuc Flag

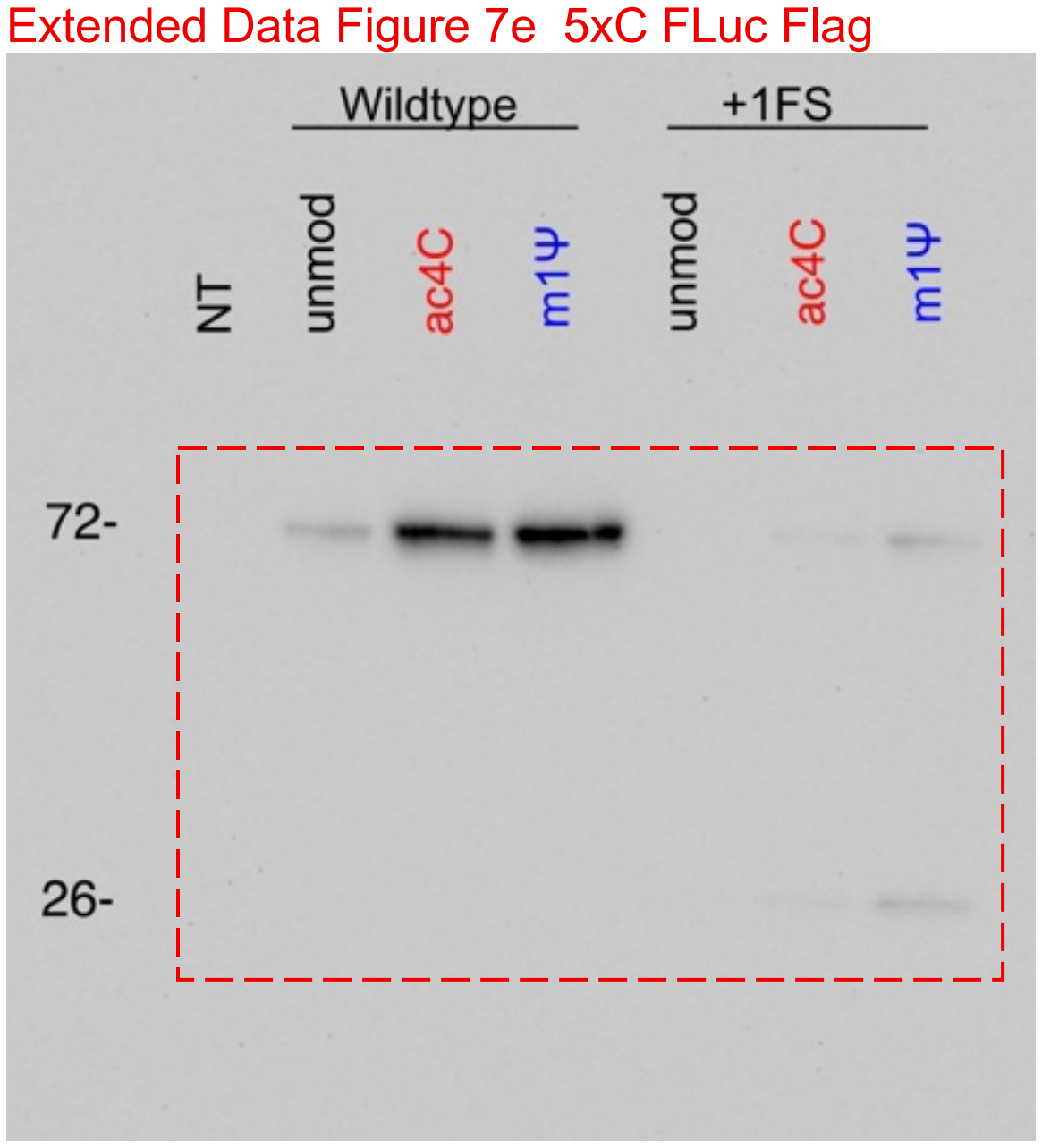

Extended Data Figure 7e 5xC FLuc Flag

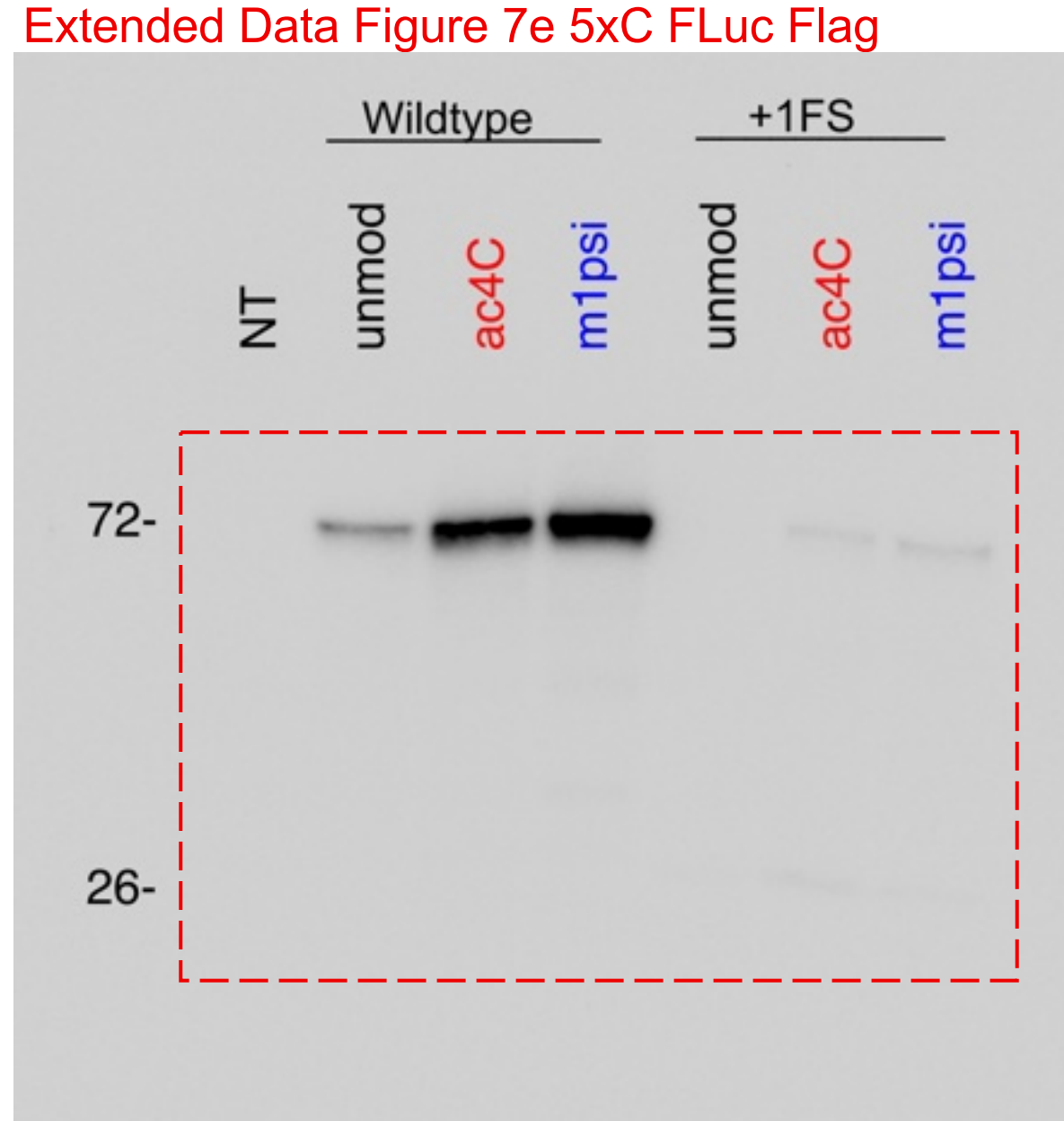

Extended Data Figure 7e 5xC FLuc GAPDH

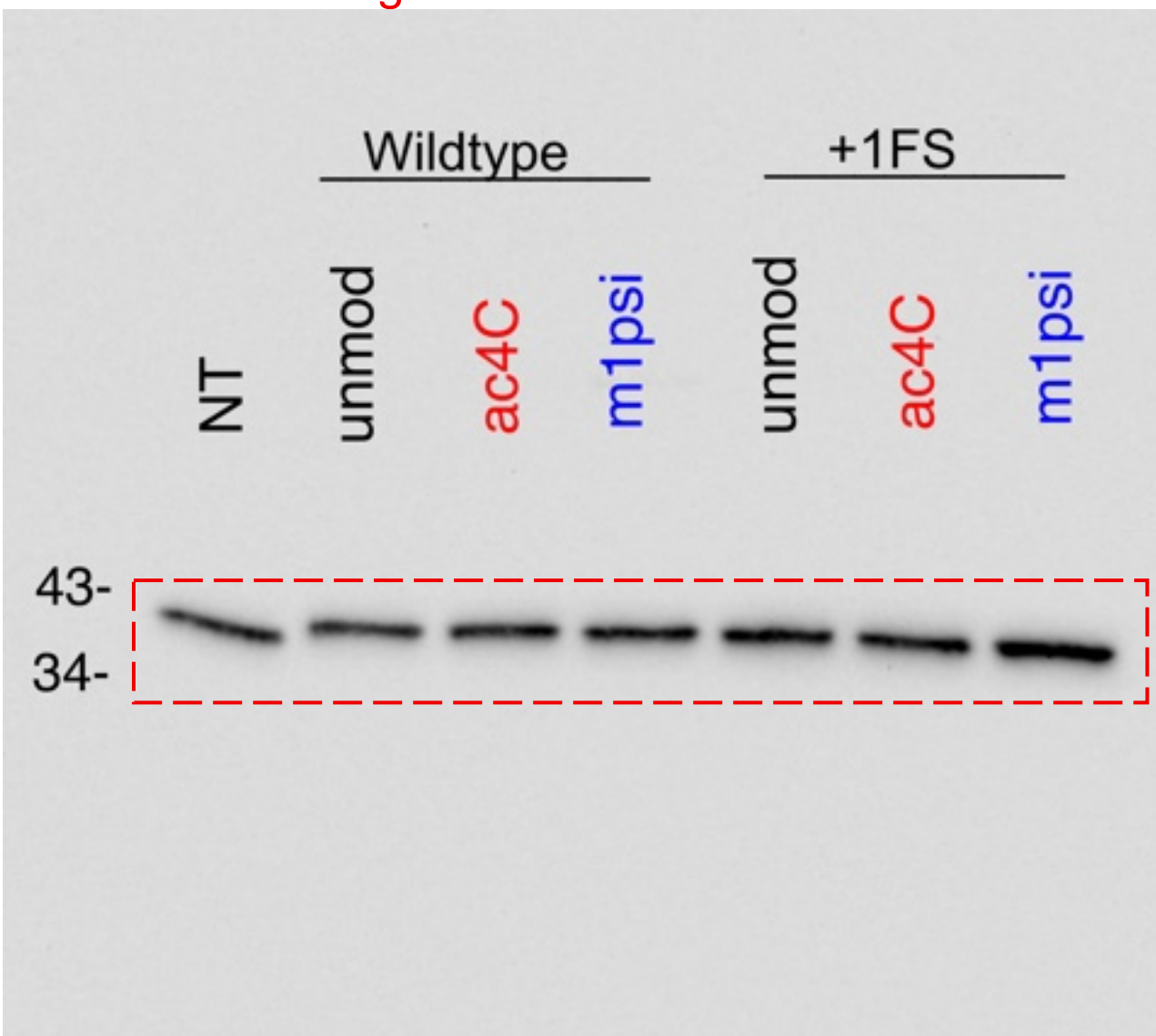

Extended Data Figure 7e 5xC FLuc GAPDH

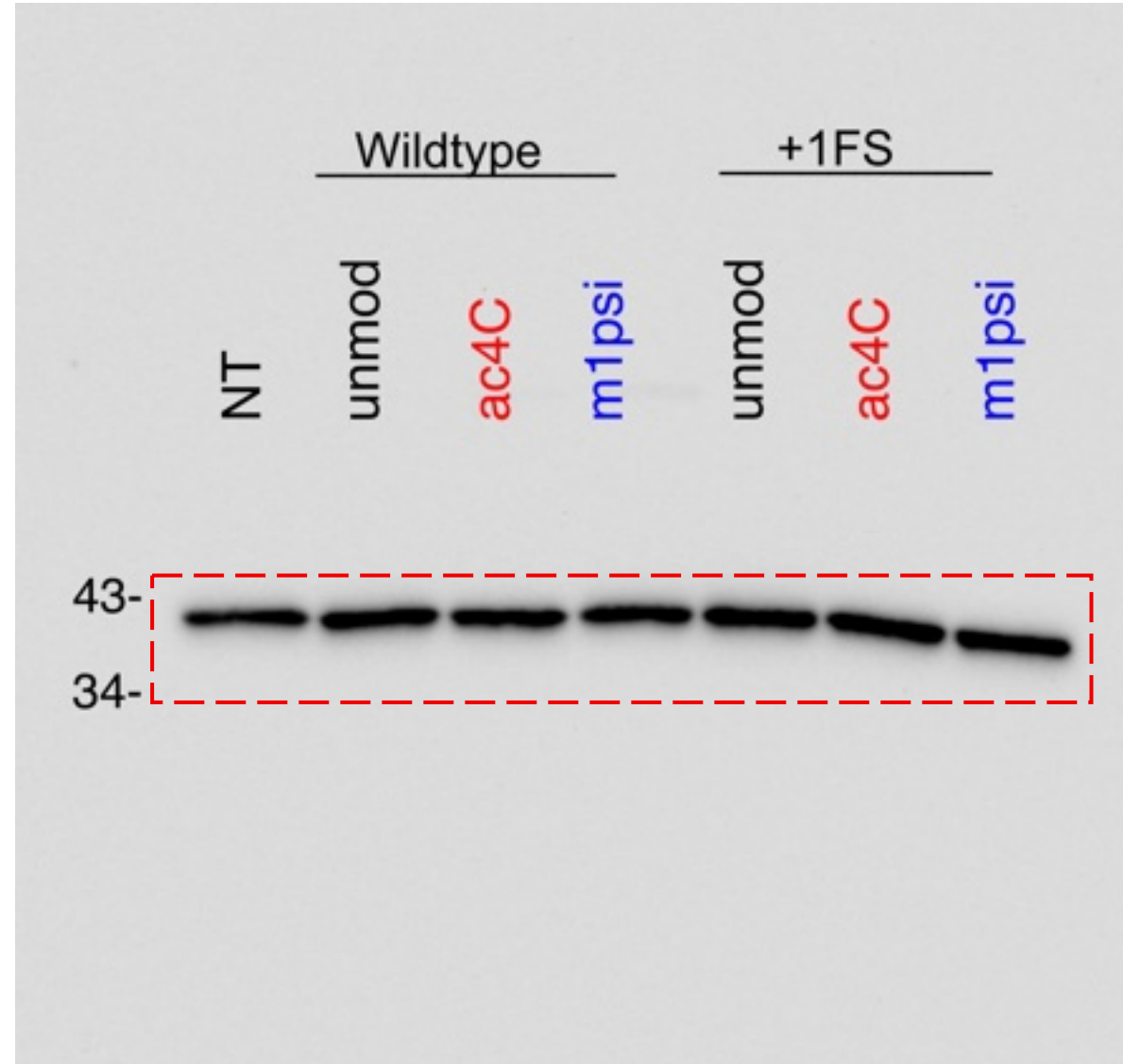

Extended Data Figure 7f 5xU FLuc Flag

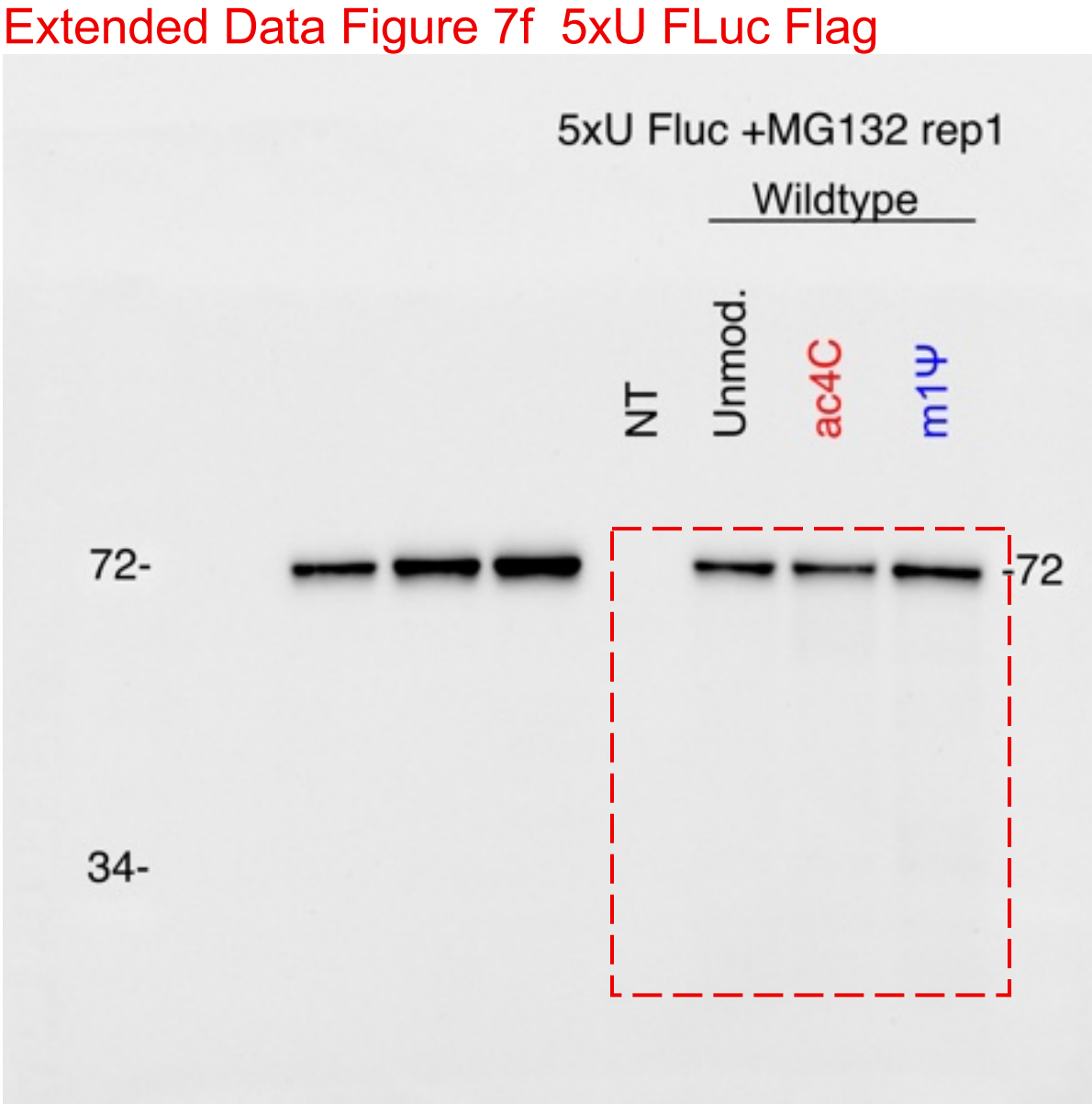

Extended Data Figure 7f 5xU FLuc Flag

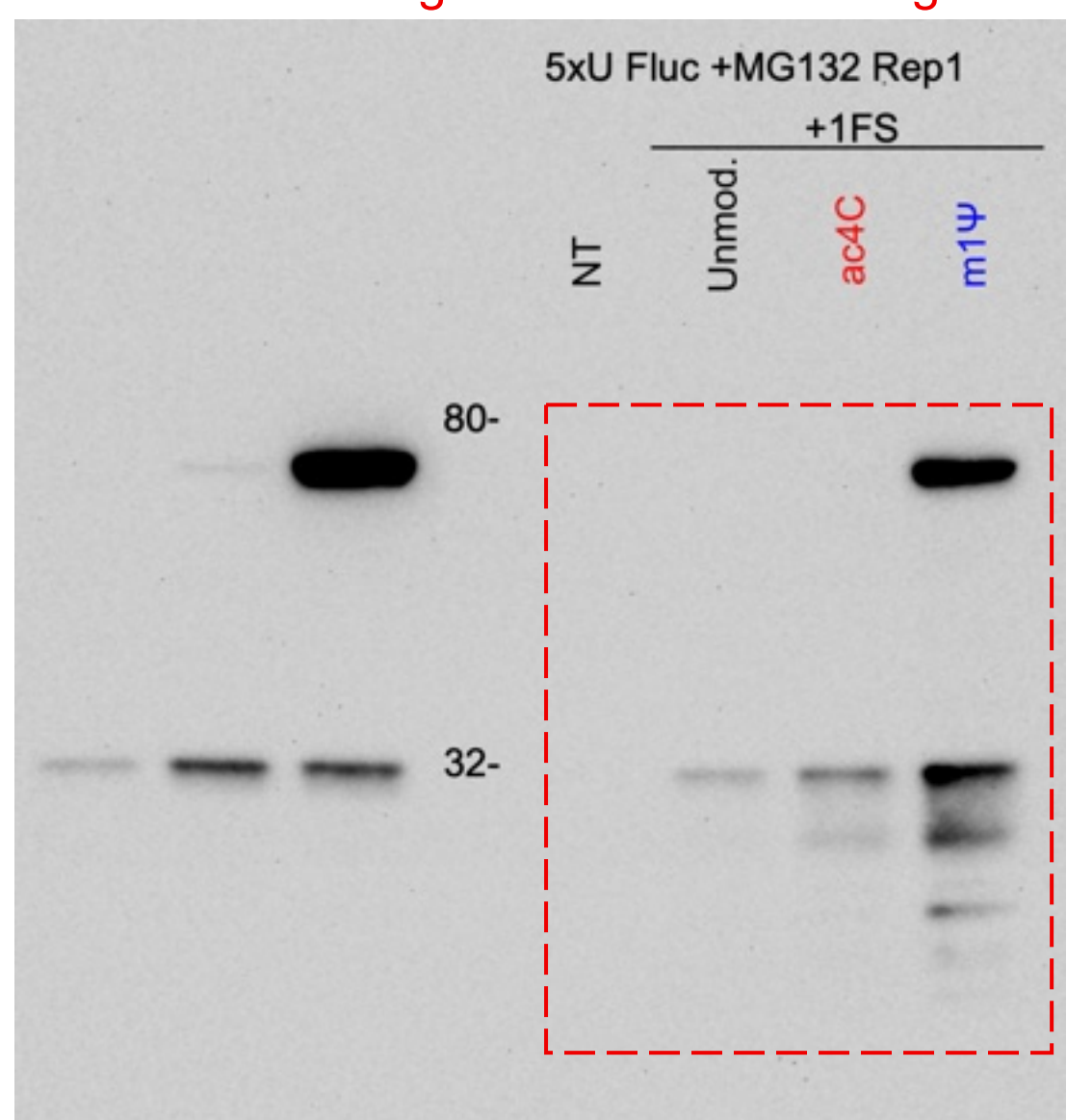

Extended Data Figure 7f 5xU FLuc GAPDH

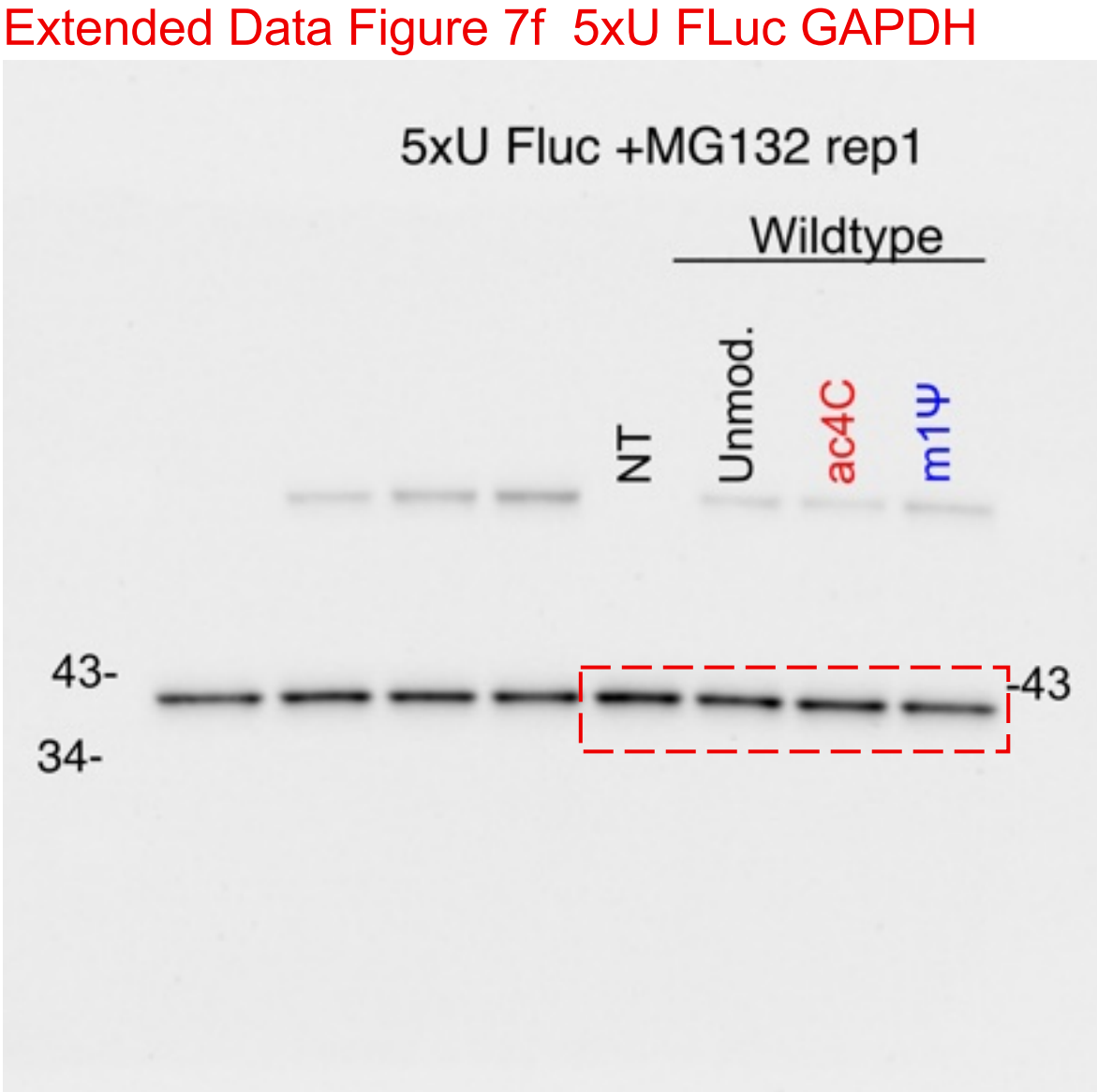

Extended Data Figure 7f 5xU FLuc GAPDH

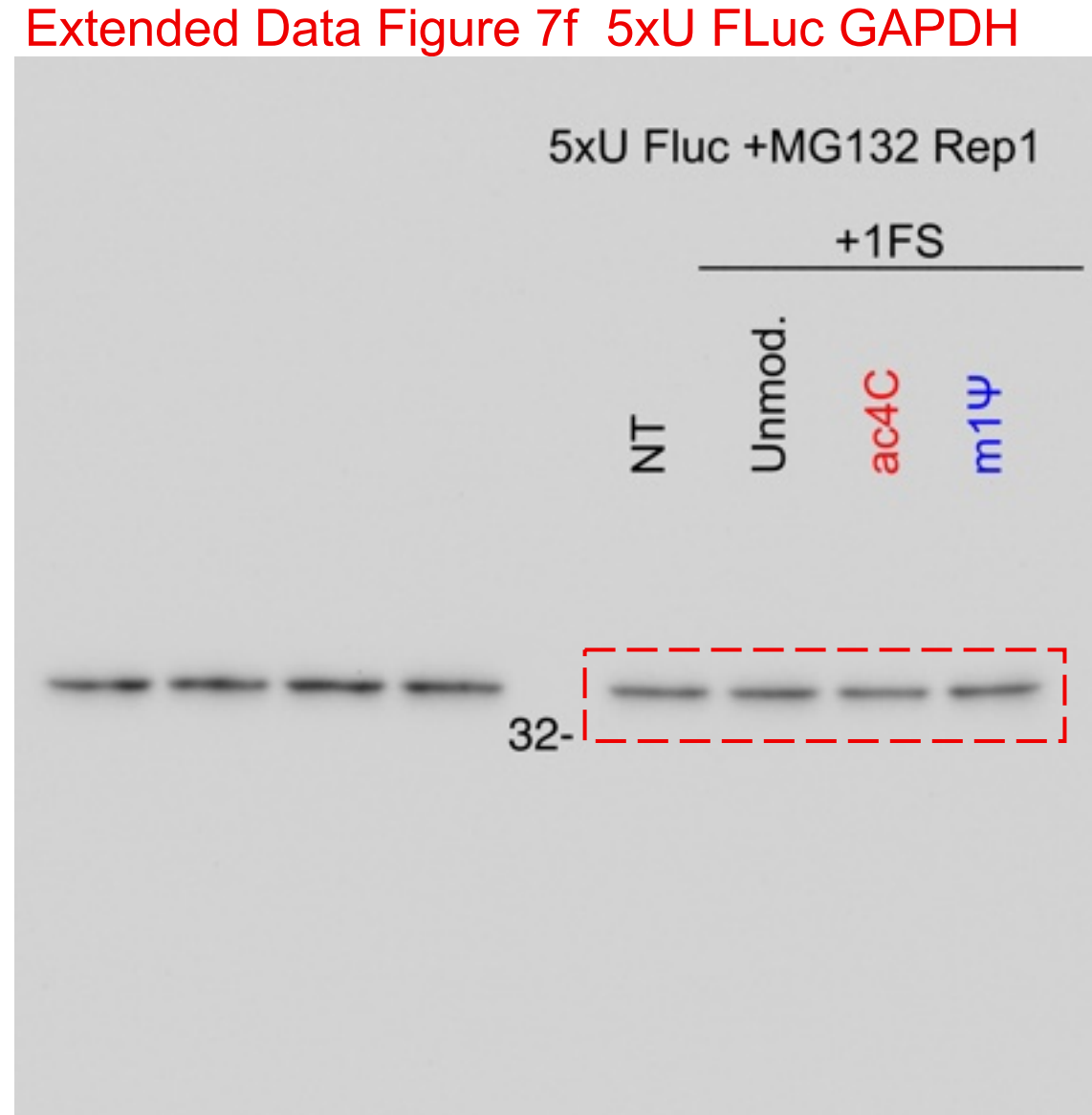

Extended Data Figure 7f 5xC FLuc Flag

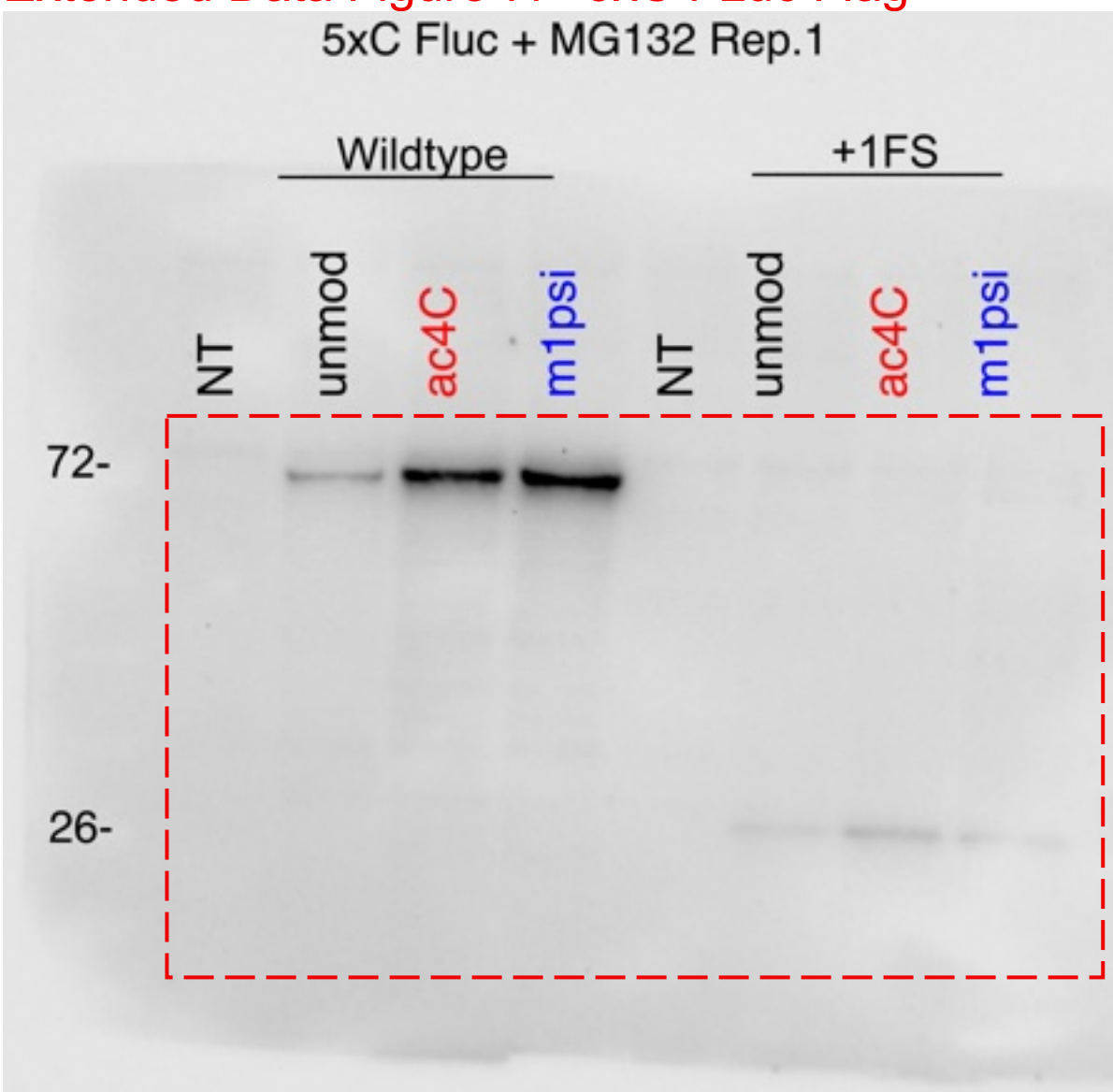

Extended Data Figure 7f 5xC FLuc GAPDH

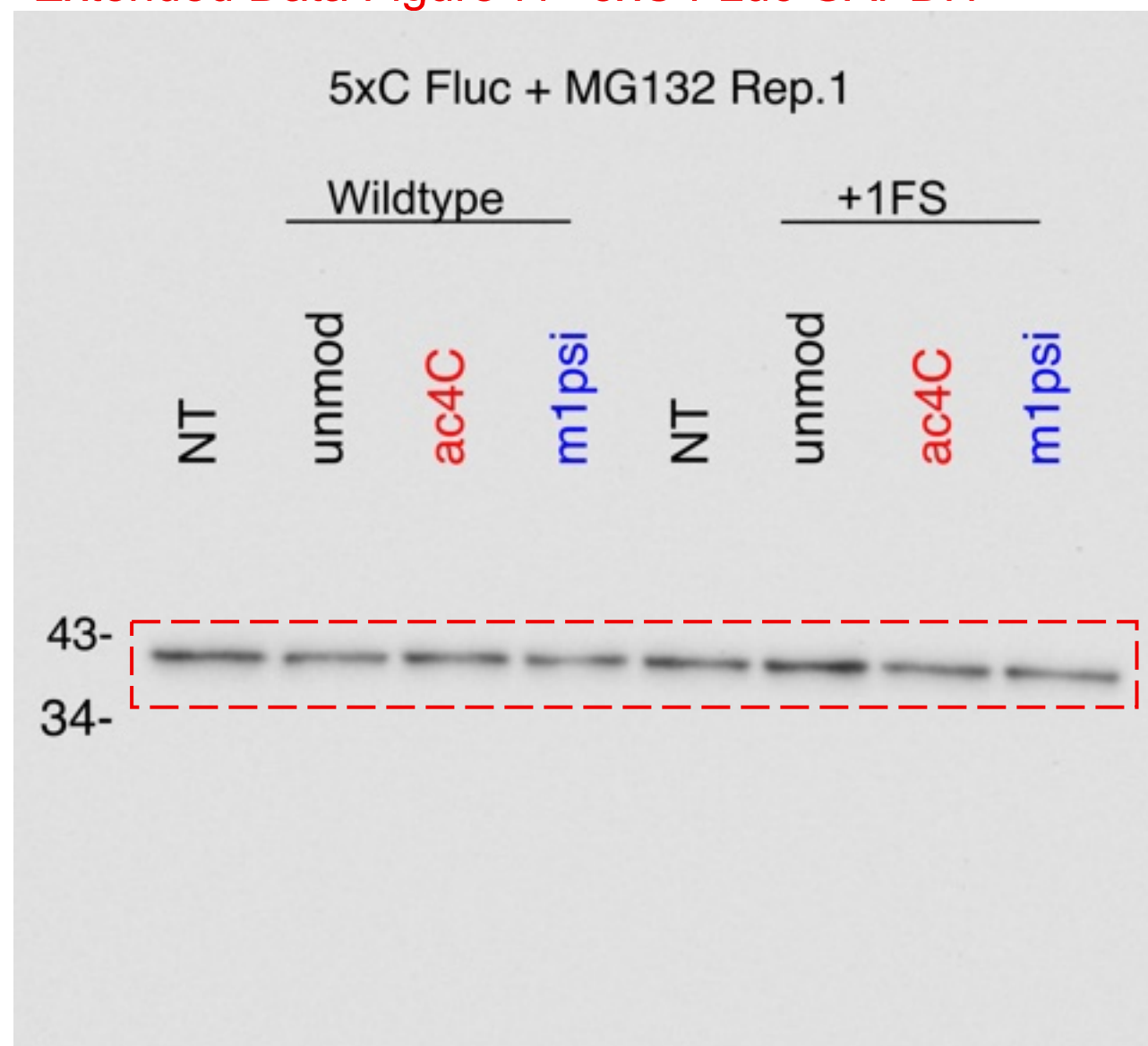

Extended Data Figure 7g 5xU FLuc Flag not shown

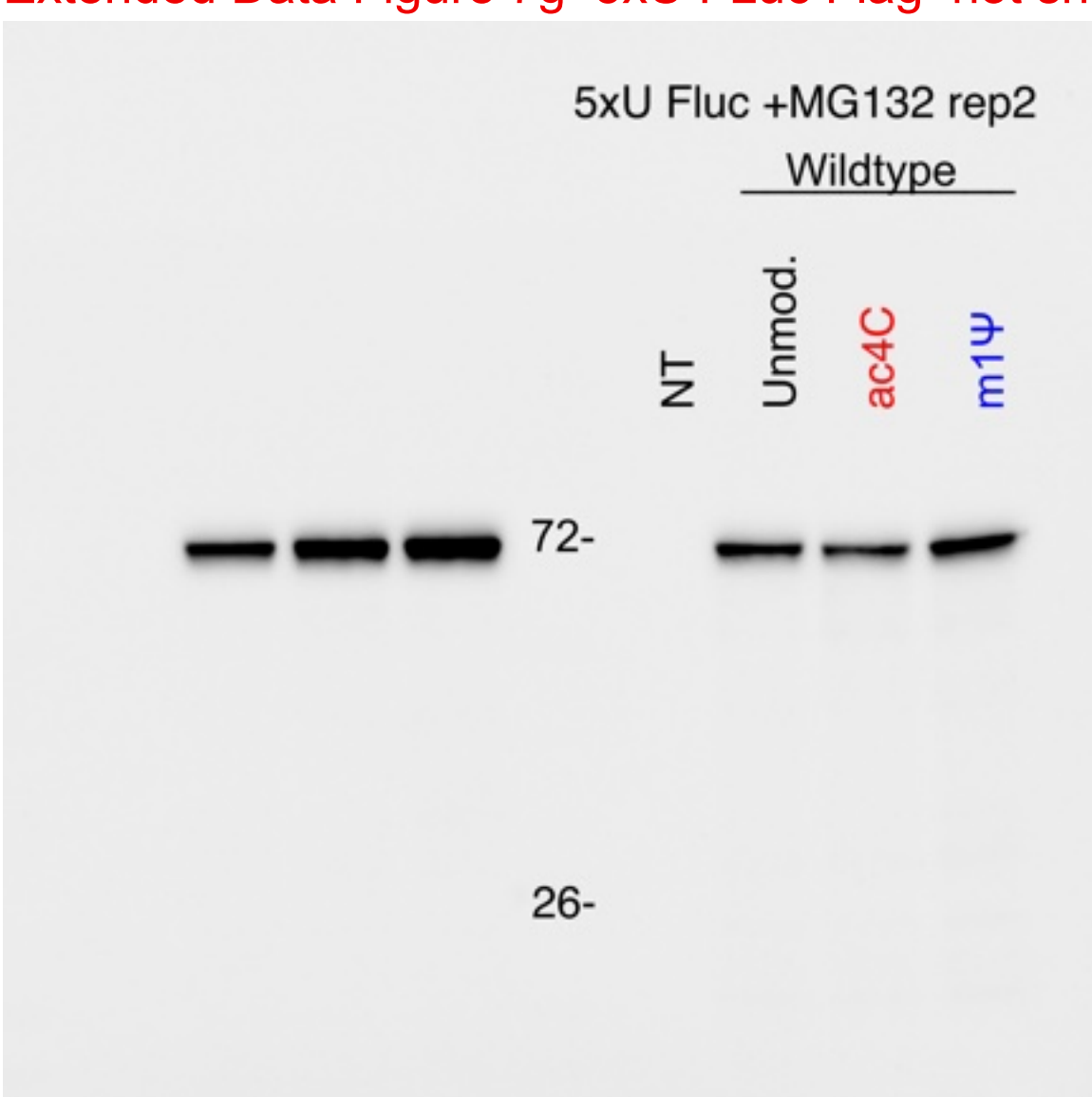

Extended Data Figure 7g 5xU FLuc Flag not shown

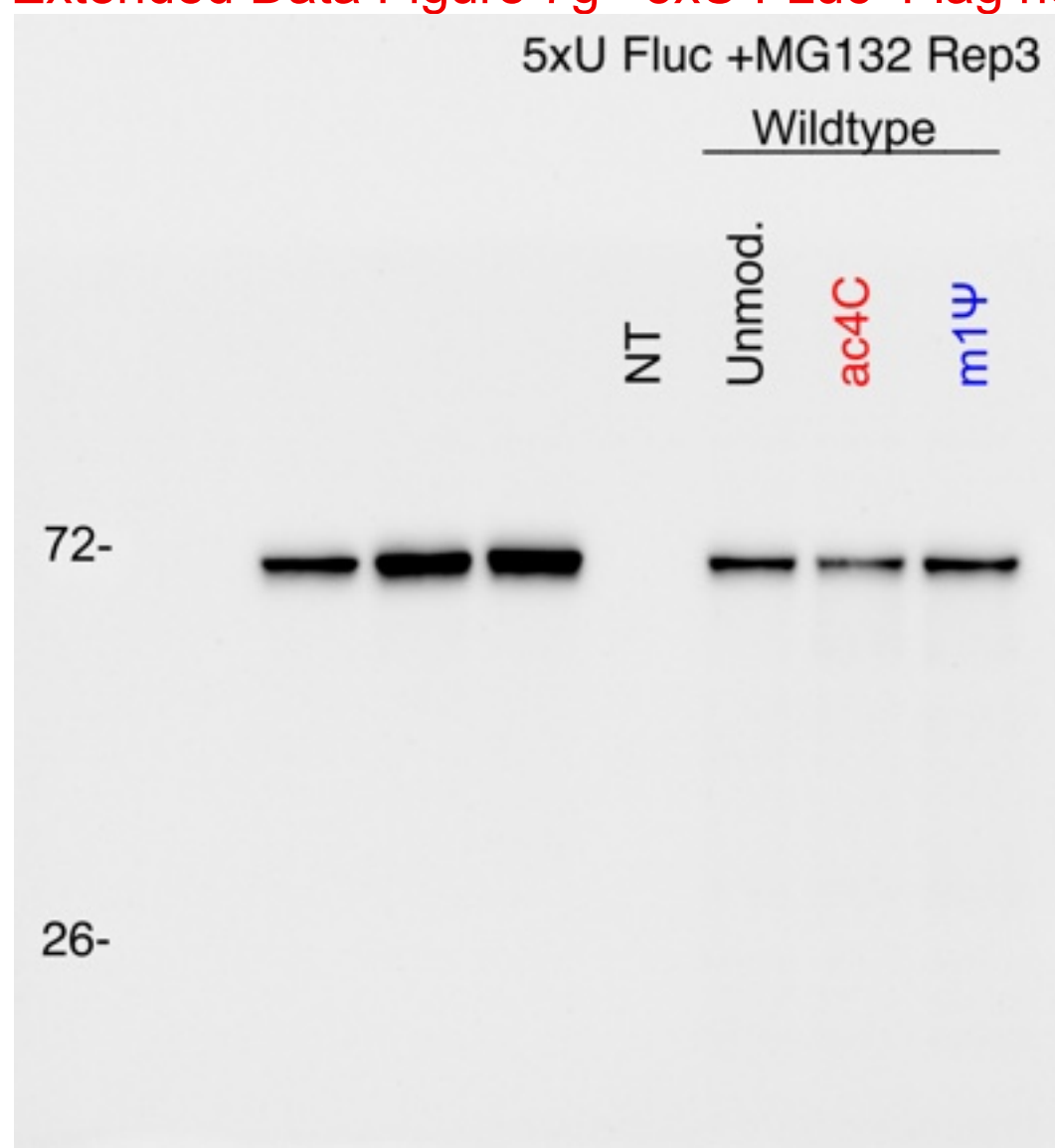

Extended Data Figure 7fg 5xU FLuc Flag not shown

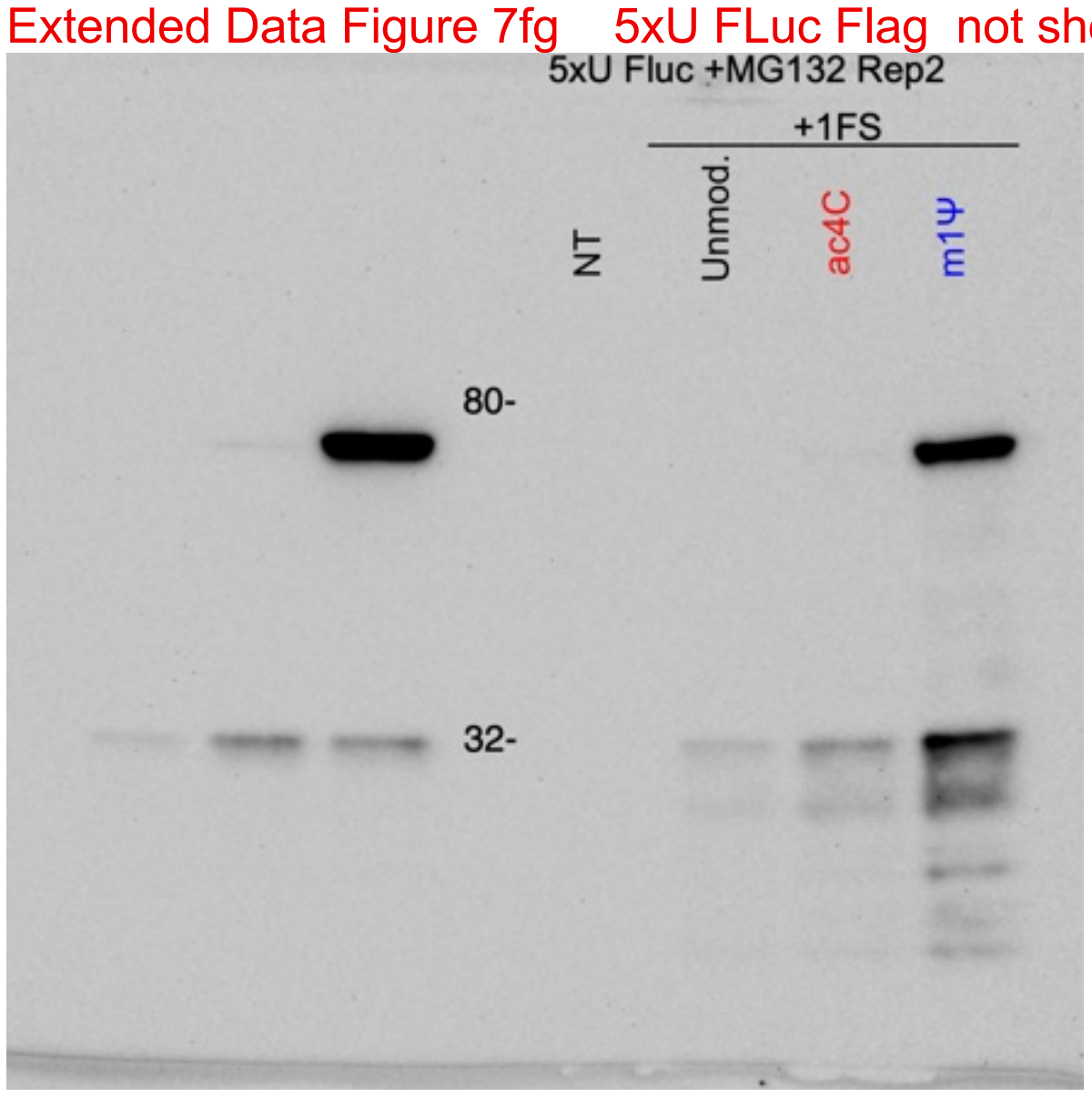

Extended Data Figure 7fg 5xU FLuc Flag not shown

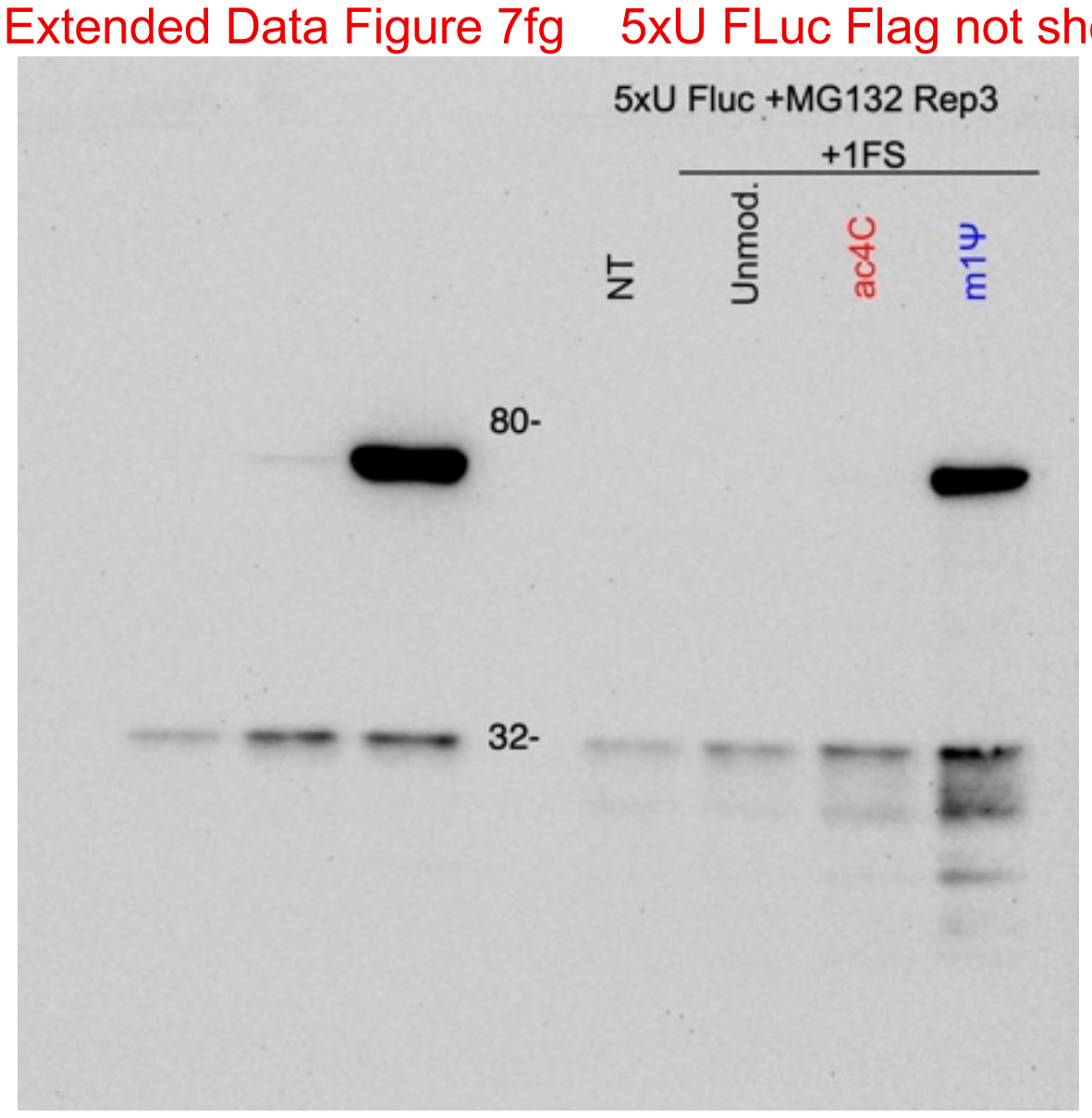

Extended Data Figure 7g GAPDH not shown

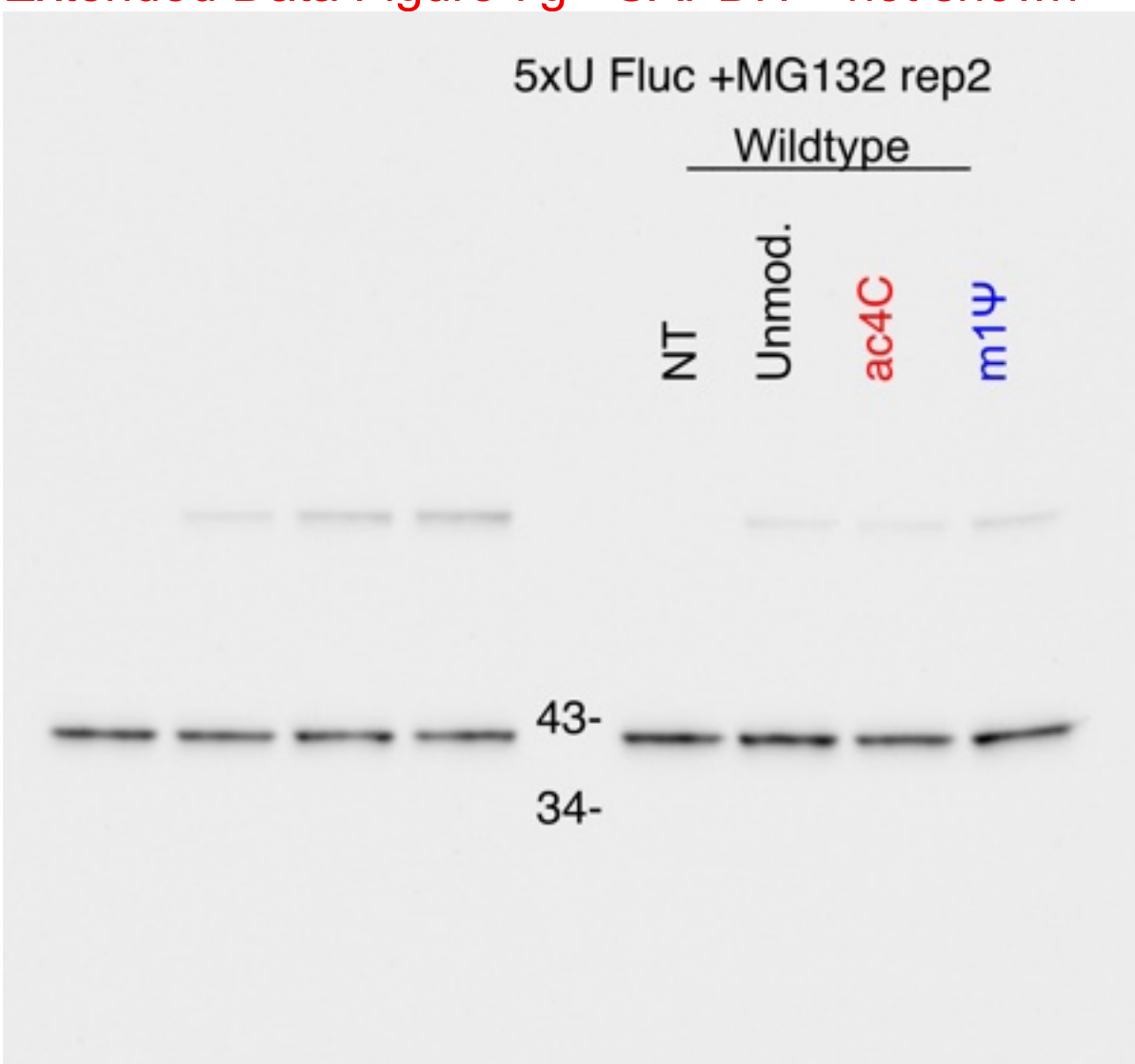

Extended Data Figure 7g GAPDH not shown

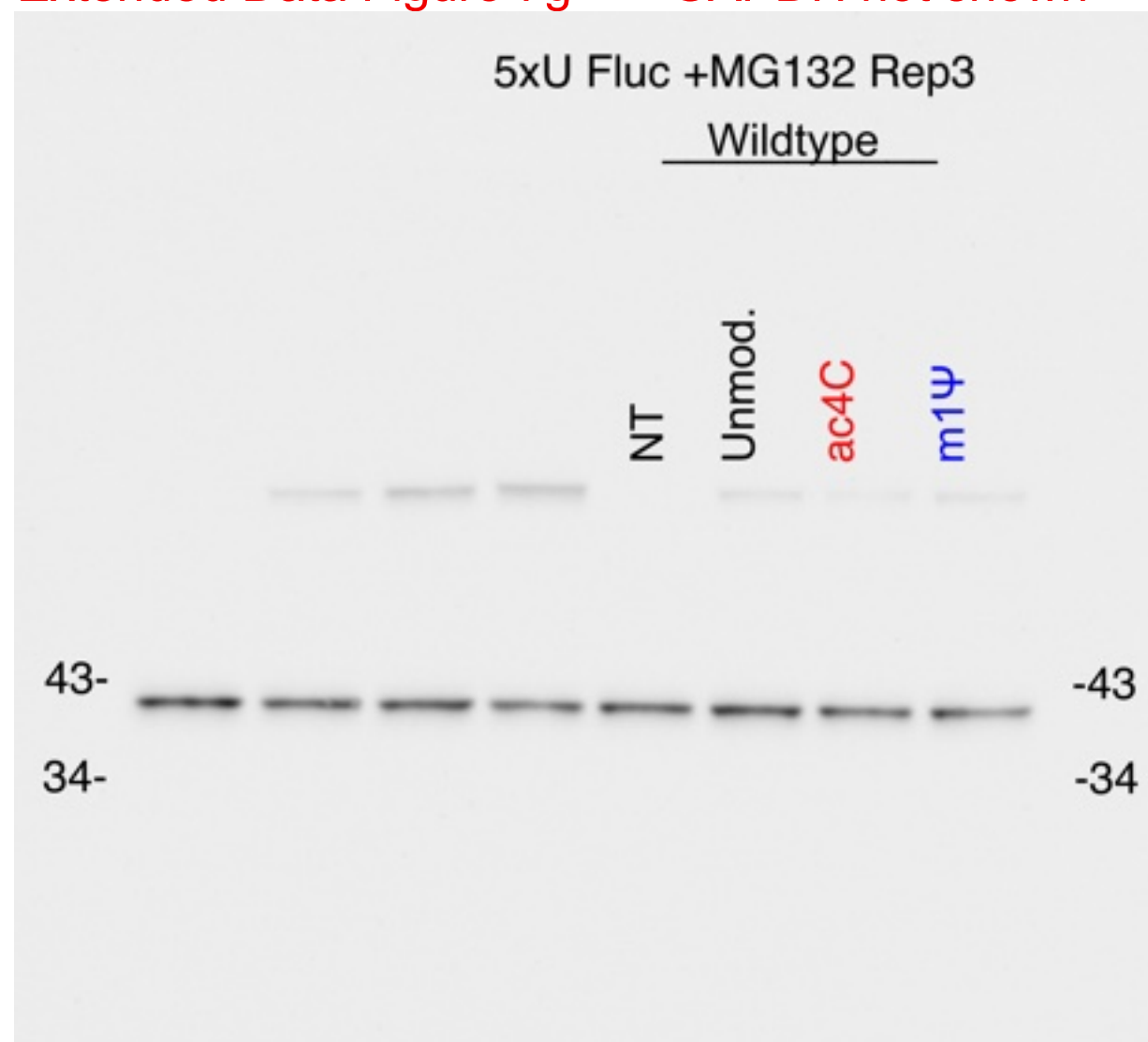

Extended Data Figure 7g 5xU FLuc GAPDH not shown

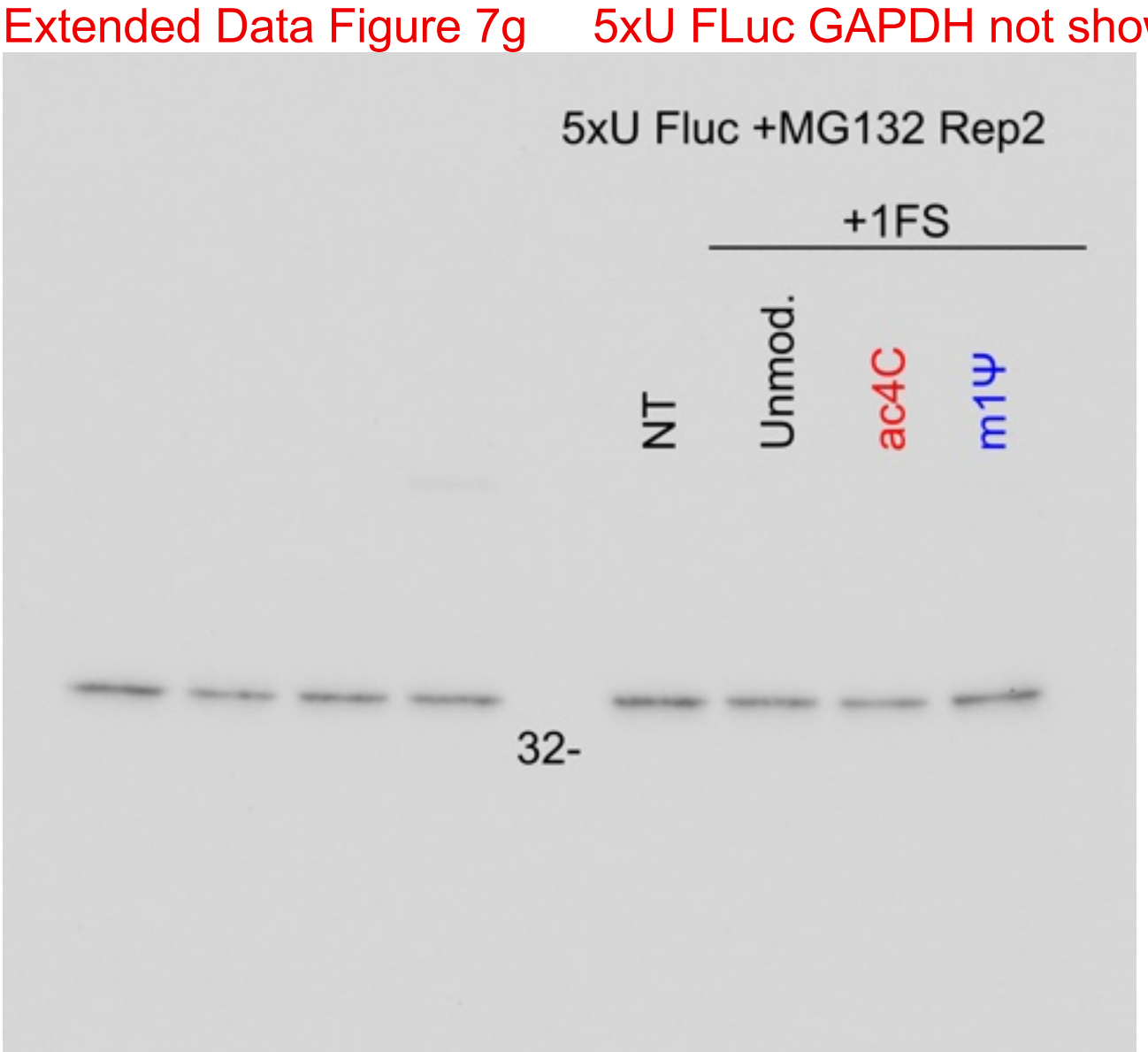

Extended Data 7g 5xU FLuc GAPDH not shown

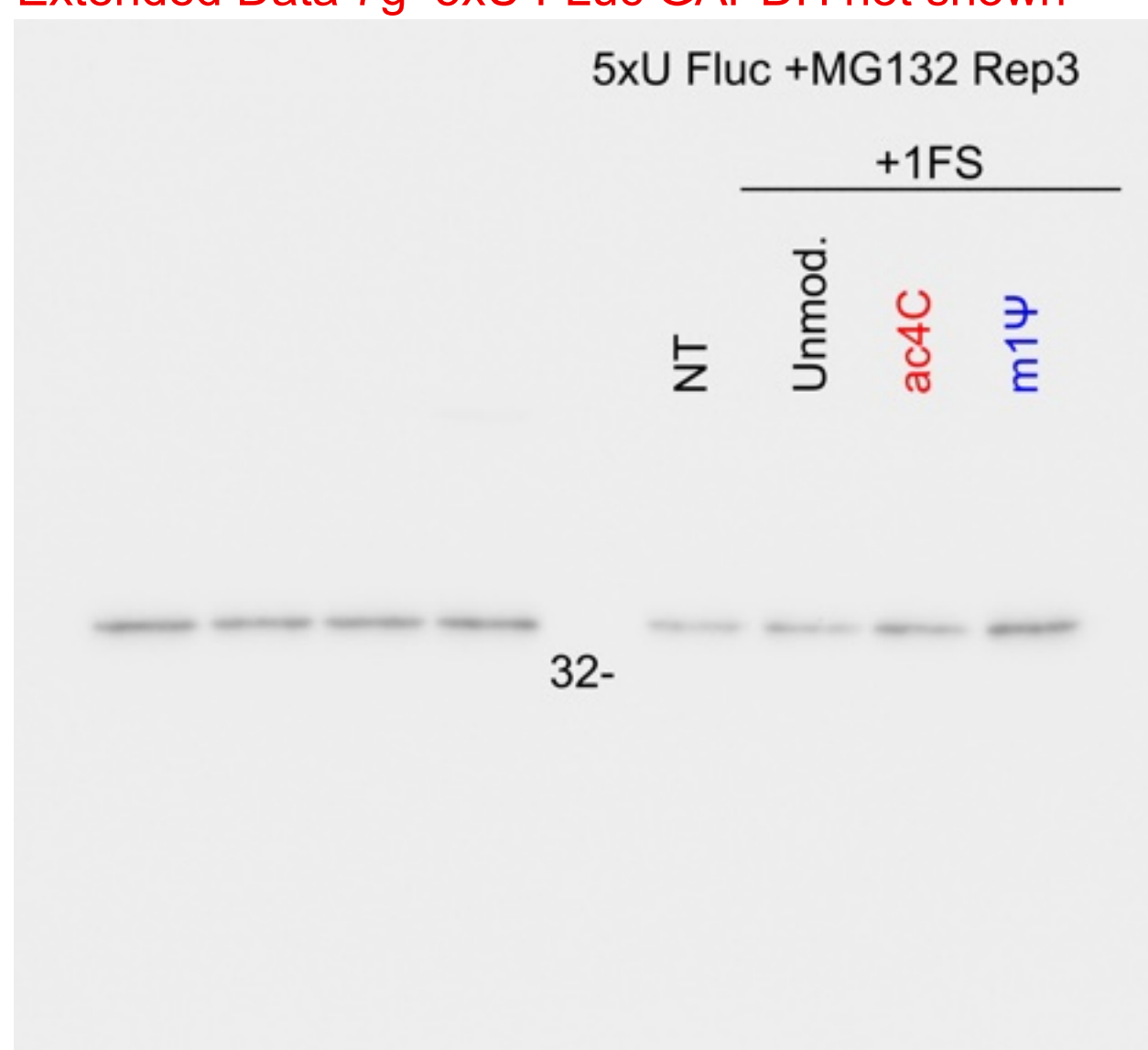

Extended Data Figure 7g 5xC FLuc Flag not shown

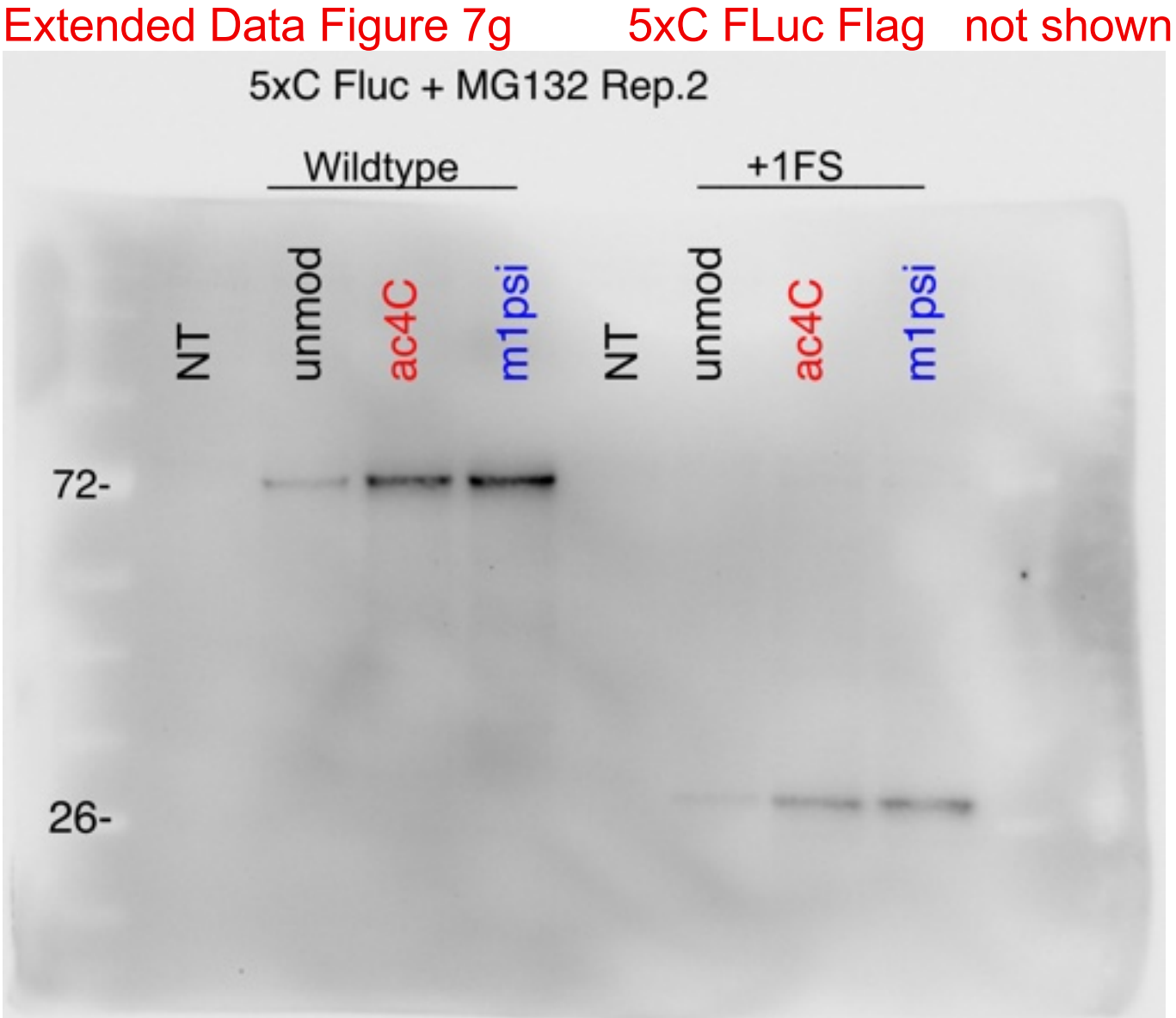

Extended Data Figure 7g 5xC FLuc Flag Not shown

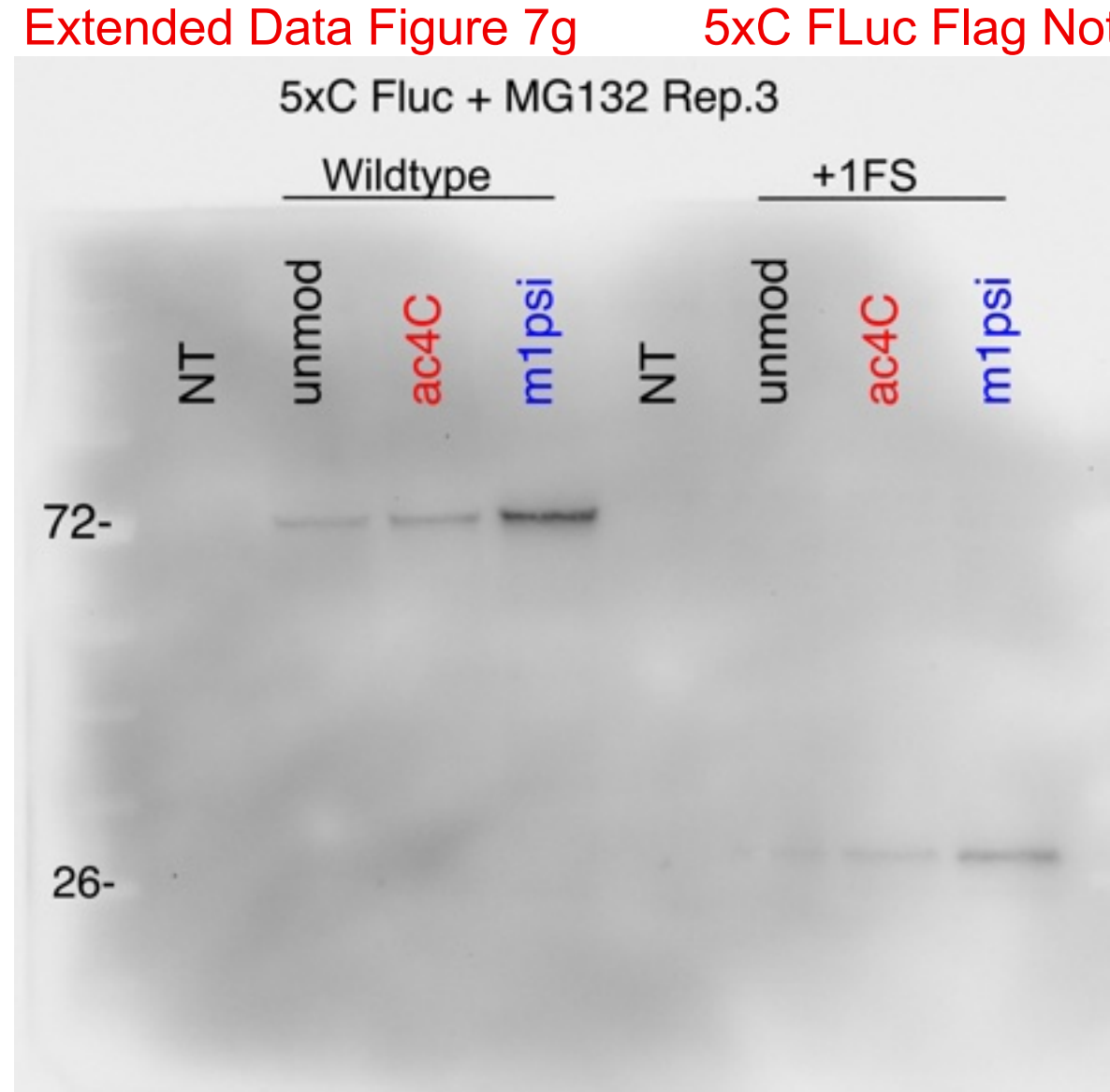

Extended Data Figure 7g GAPDH not shown

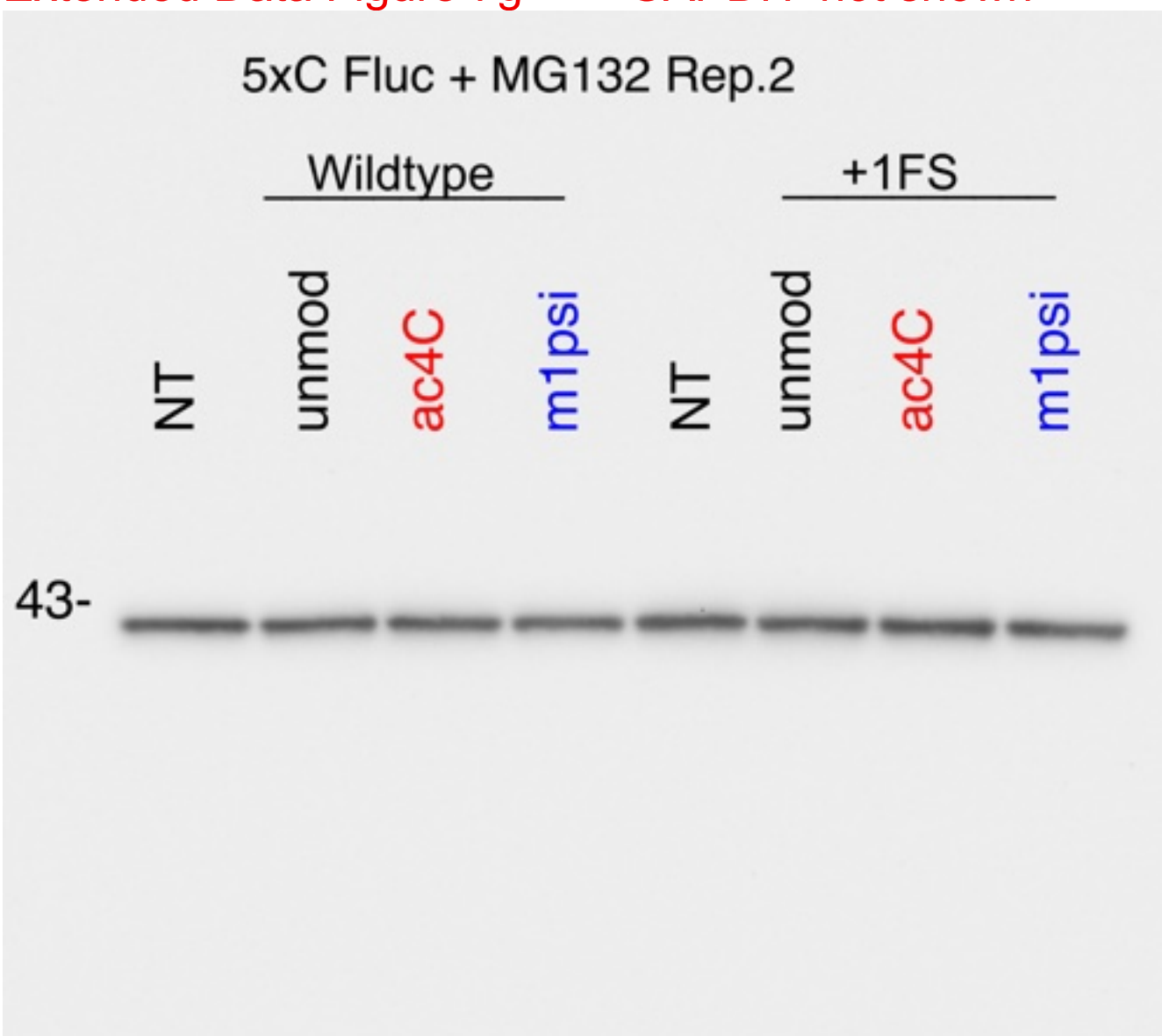

Extended Data Figure 7f GAPDH Not shown

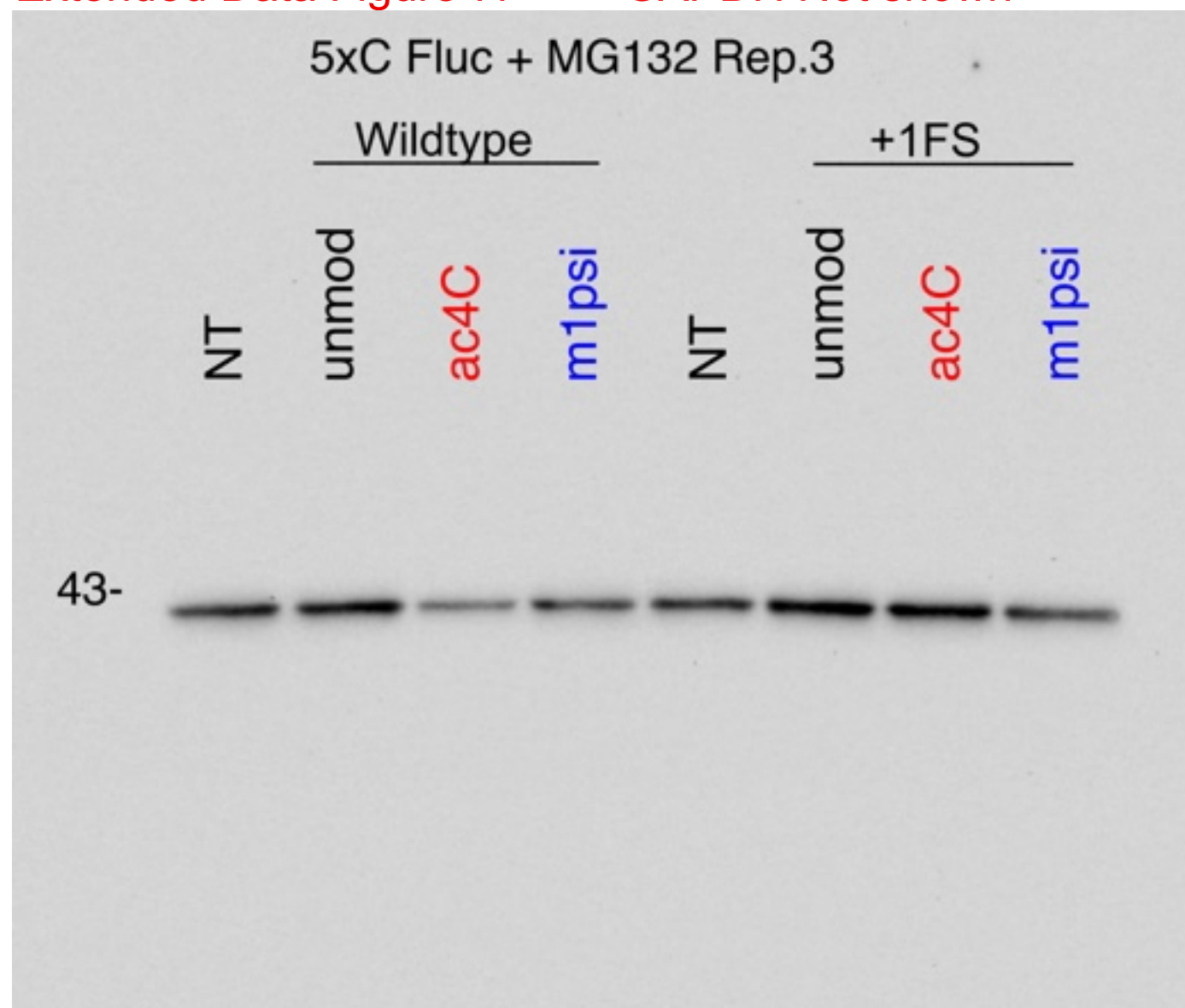

Extended Data Figure 8d WT-5xU FLuc Flag

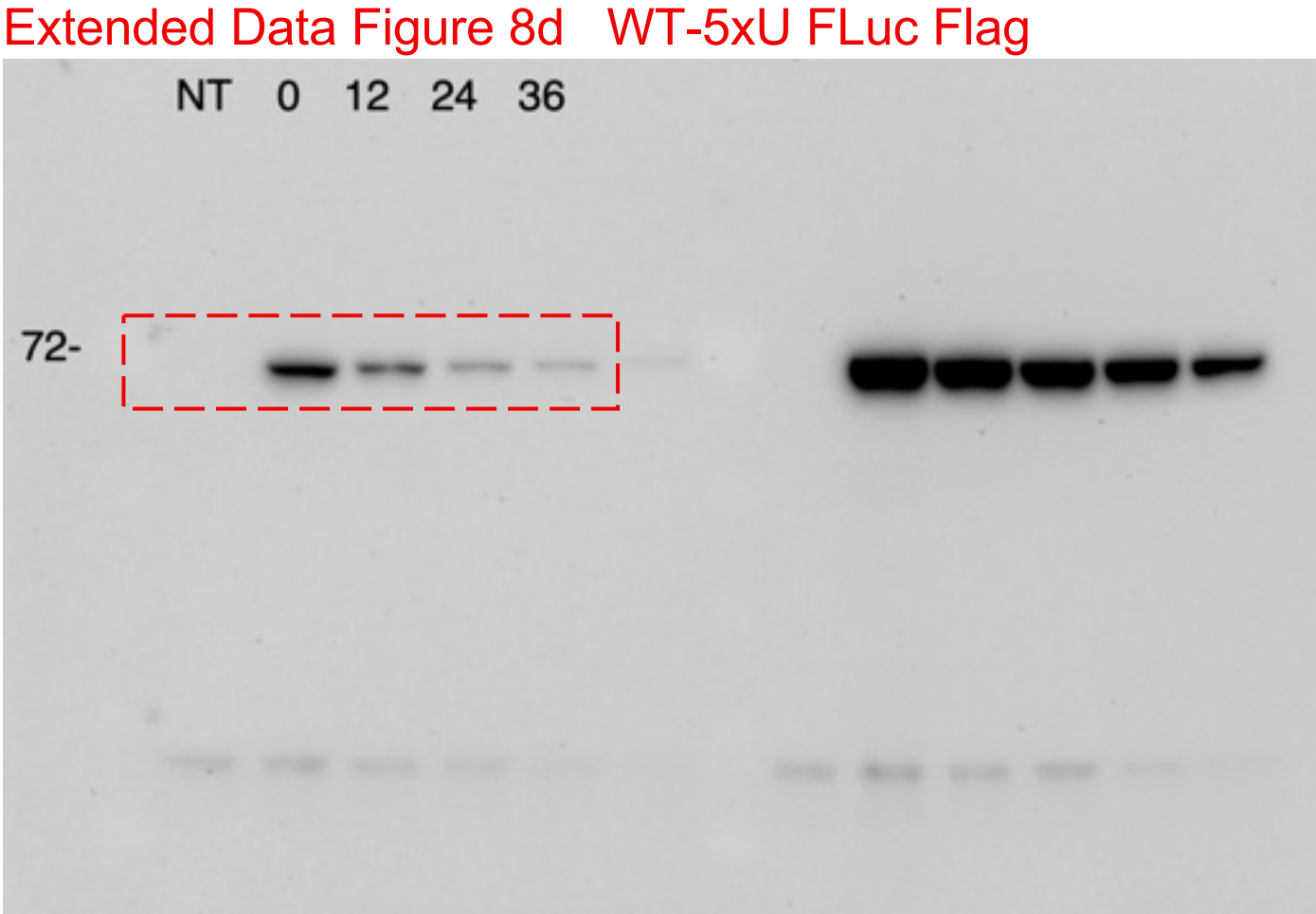

Extended Data Figure 8d WT-5xU FLuc Flag

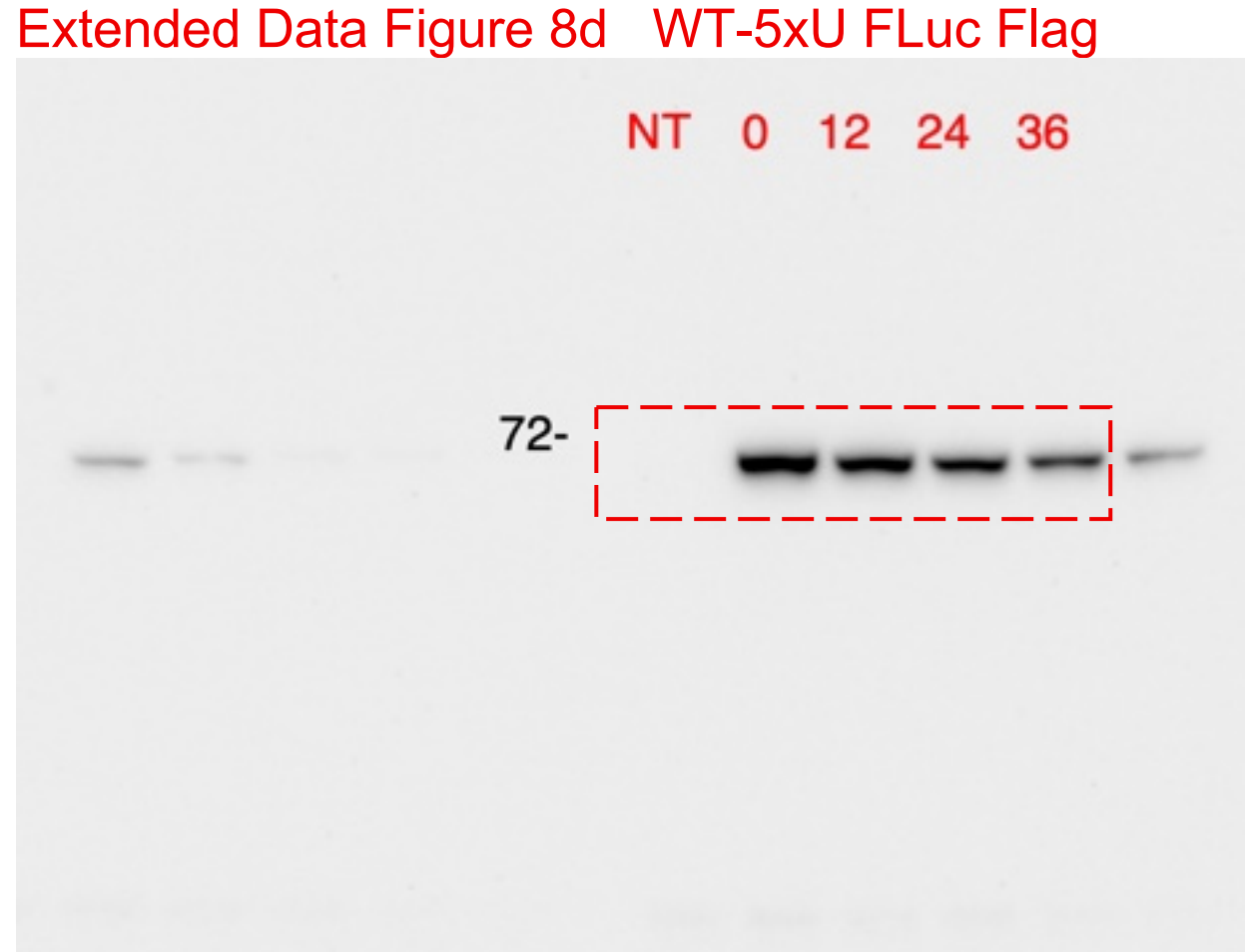

Extended Data Figure 8d WT-5xU FLuc Flag

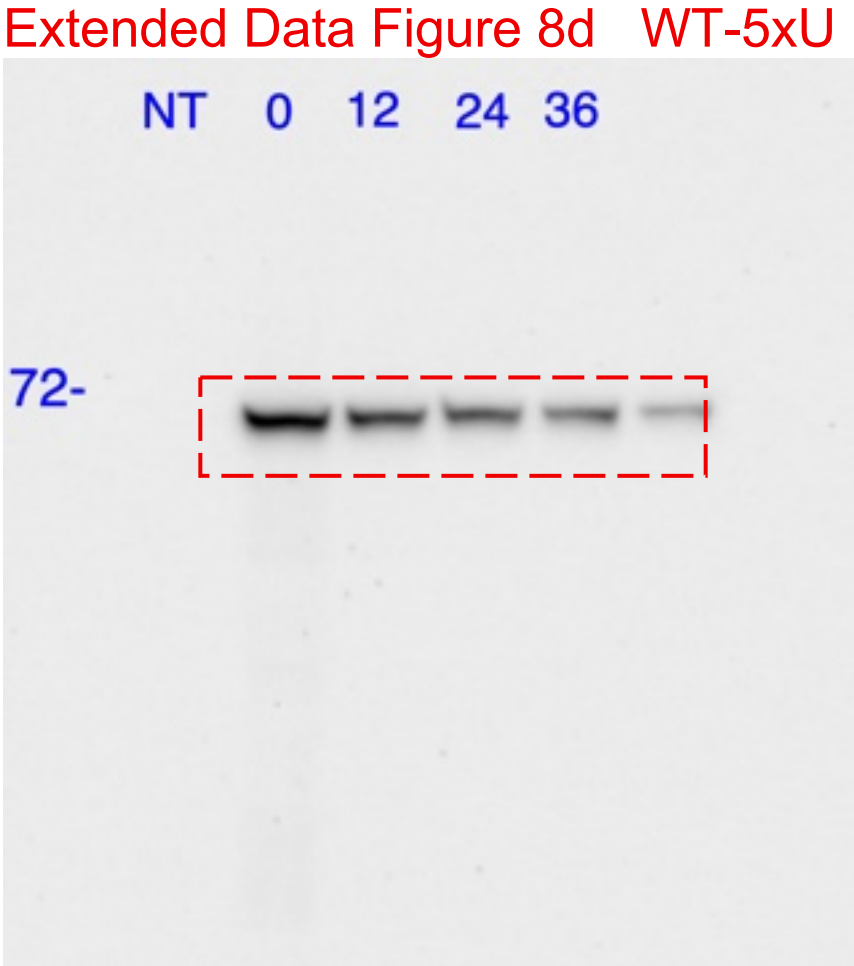

Extended Data Figure 8d WT-5xU GAPDH

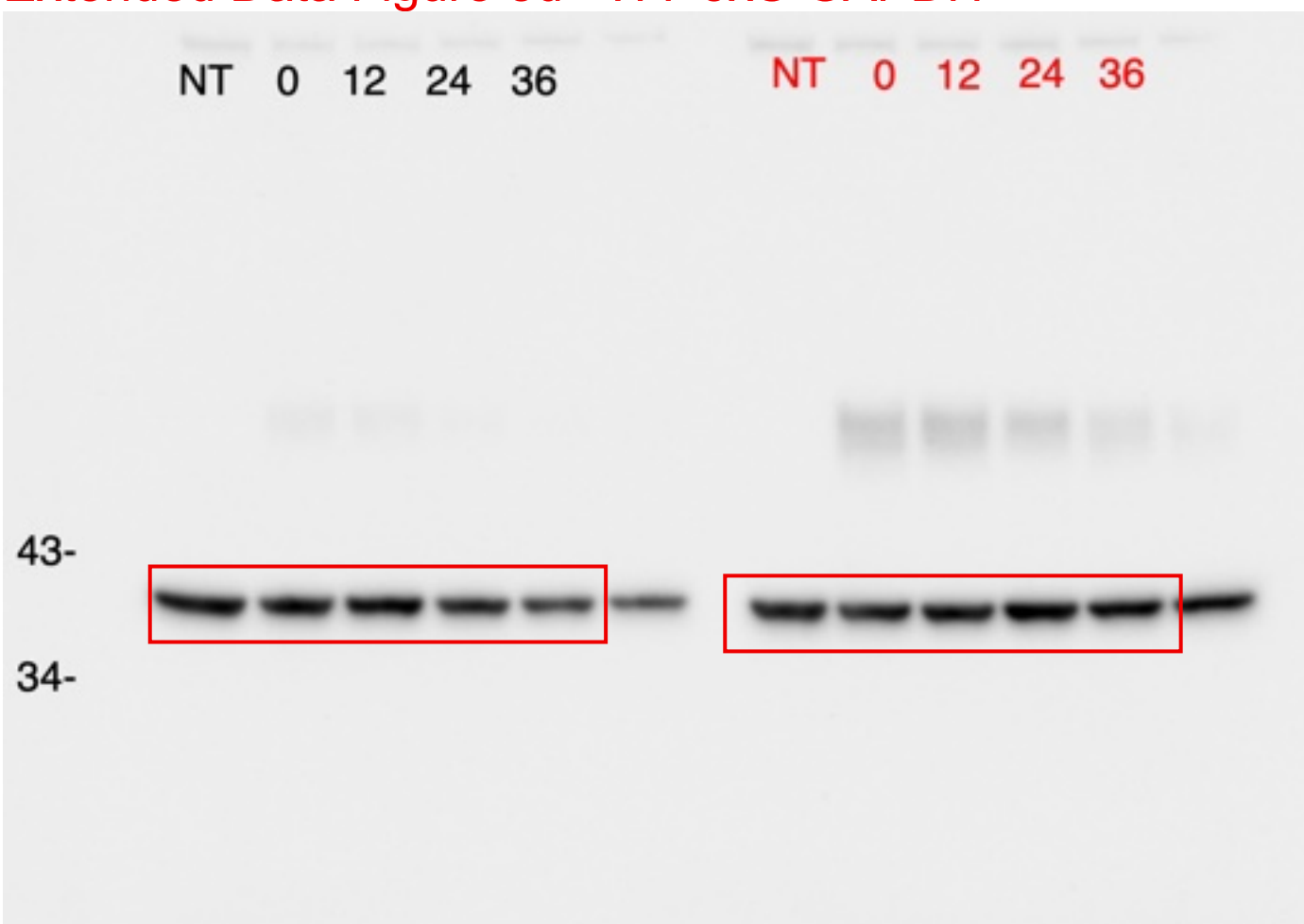

Extended Data Figure 8d WT-5xU GAPDH

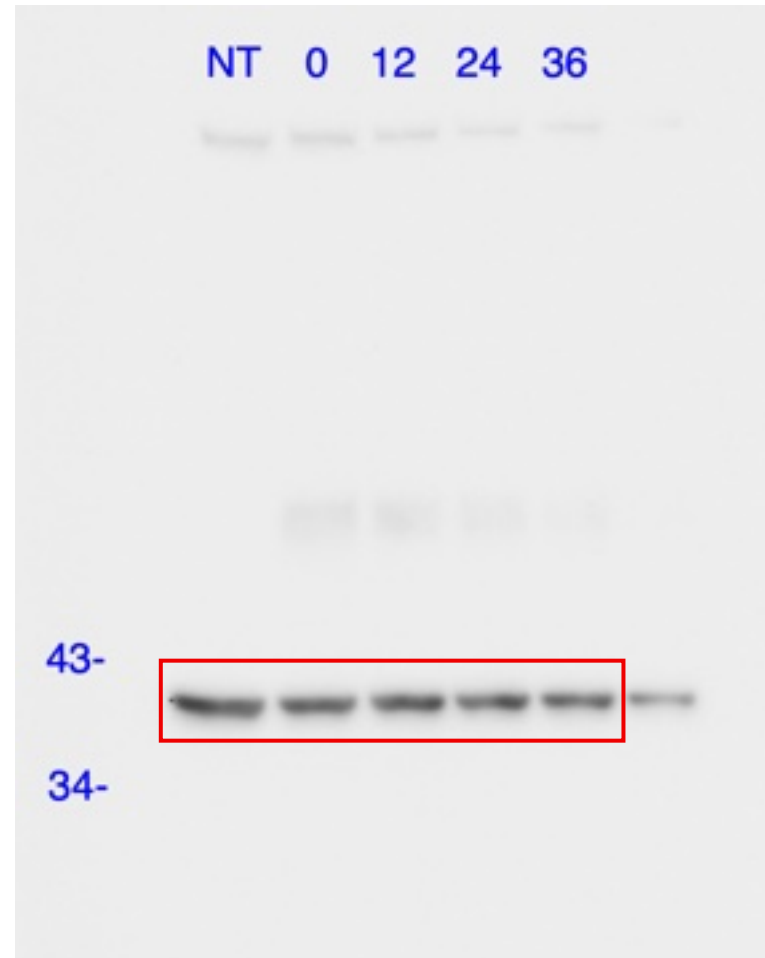

Extended Data Figure 8d CD25
Extended Data Figure 8e 1x rep not shown

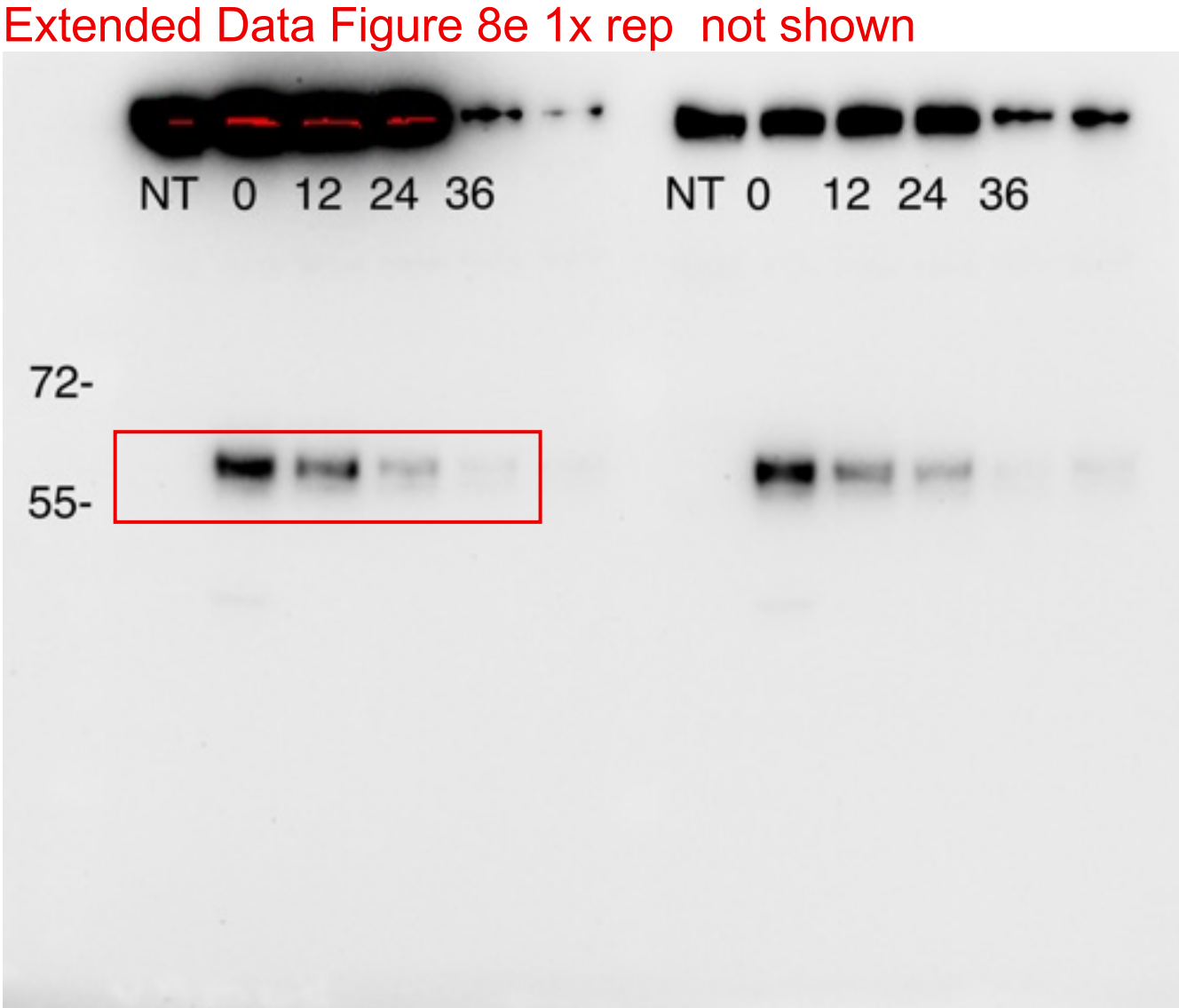

Extended Data Figure 8d CD25
Extended Data Figure 8e 1x rep not shown

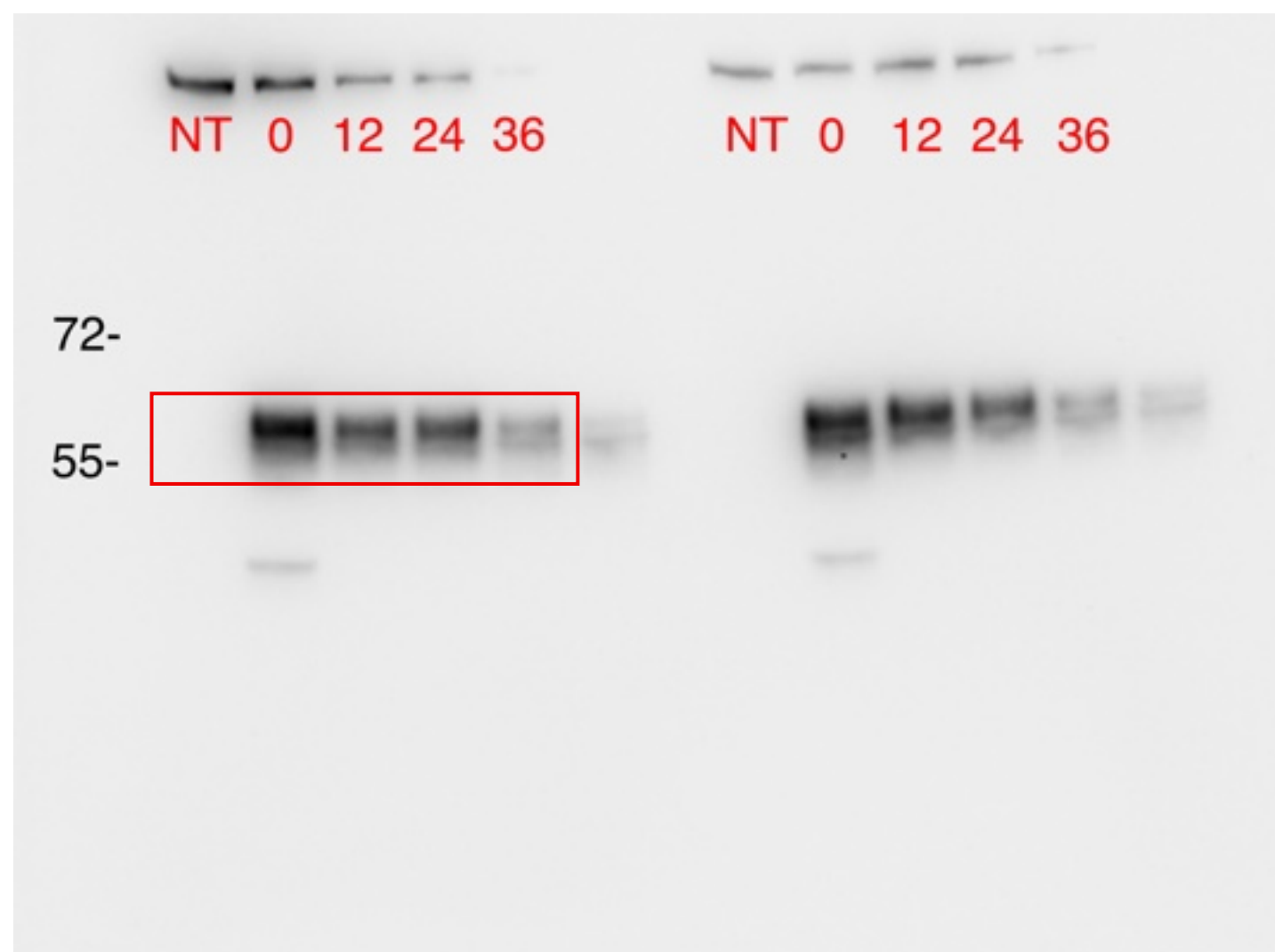

Extended Data Figure 8d CD25
Extended Data Figure 8e 1x rep not shown

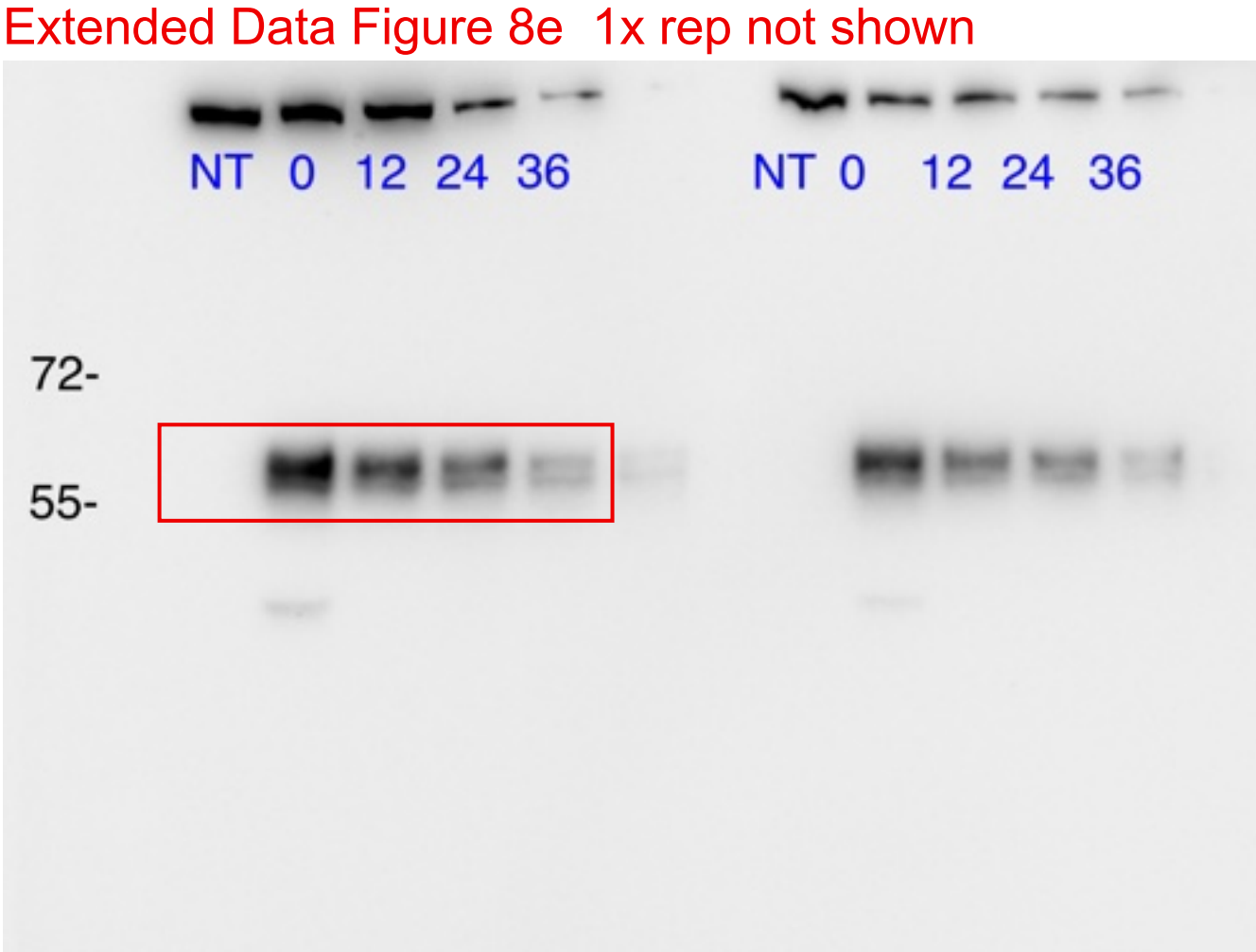

Extended Data Figure 8d GAPDH
Extended Data Figure 8e 1x rep not shown

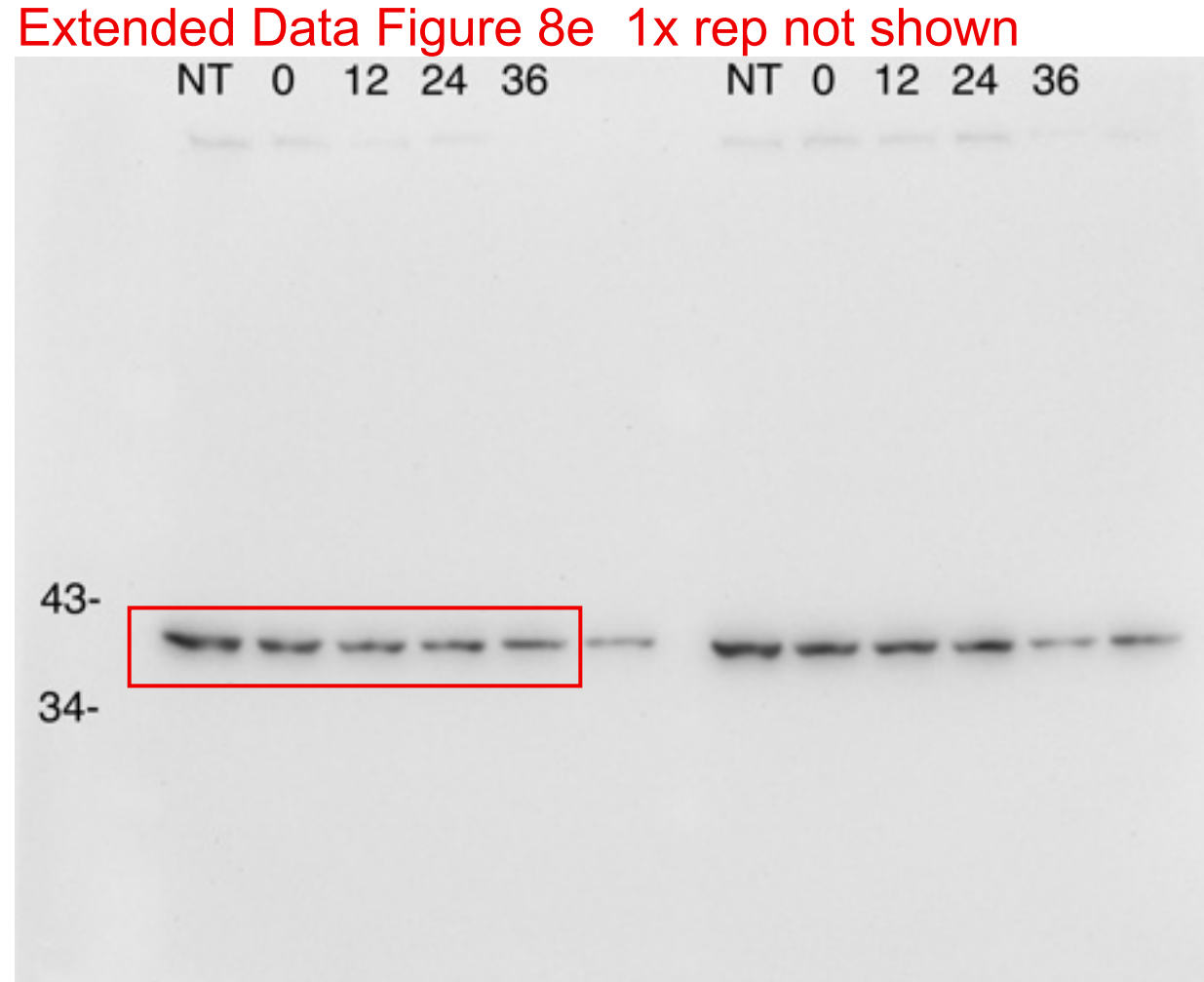

Extended Data Figure 8d GAPDH
Extended Data Figure 8e 1x rep not shown

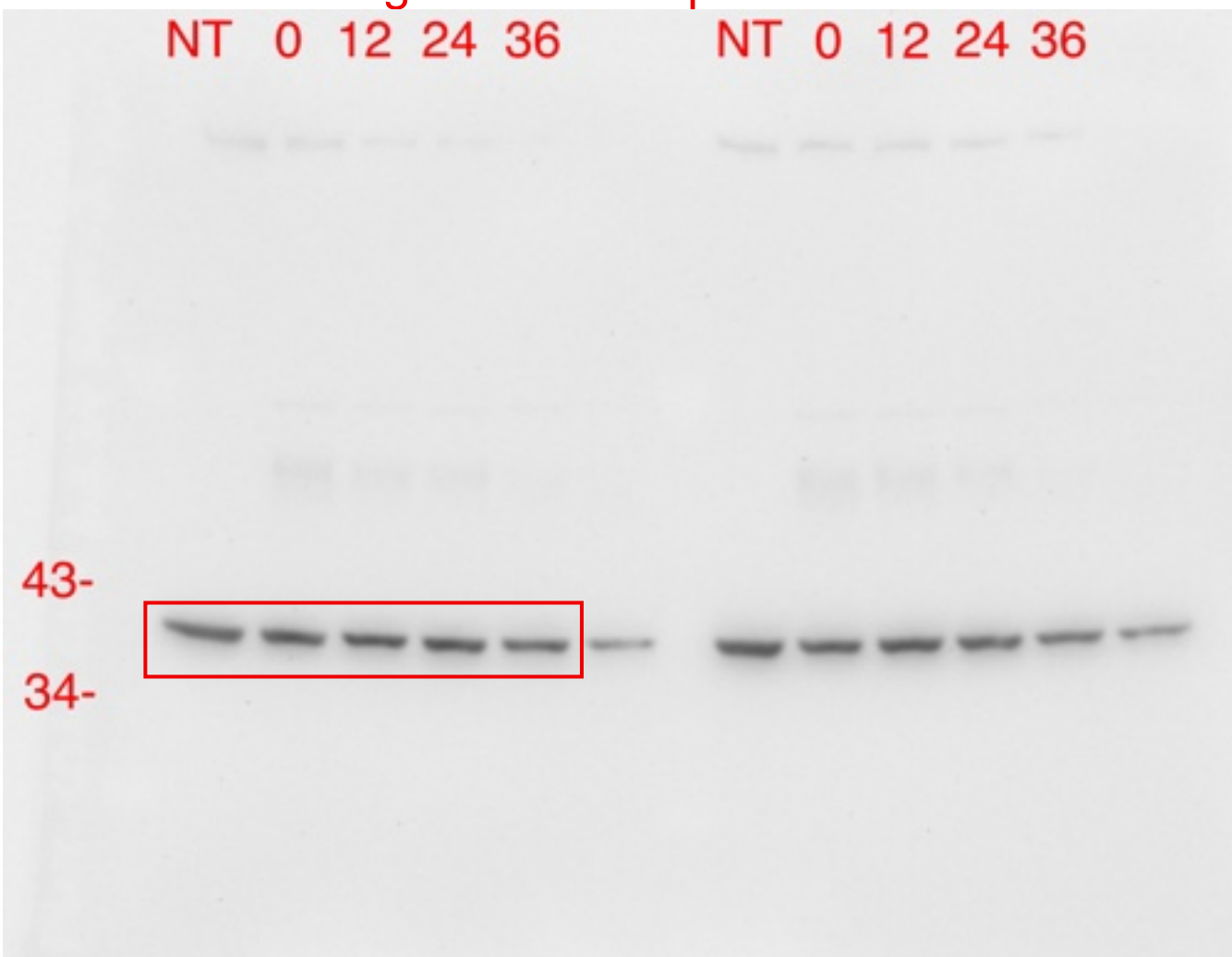

Extended Data Figure 8d GAPDH
Extended Data Figure 8e 1x rep not shown

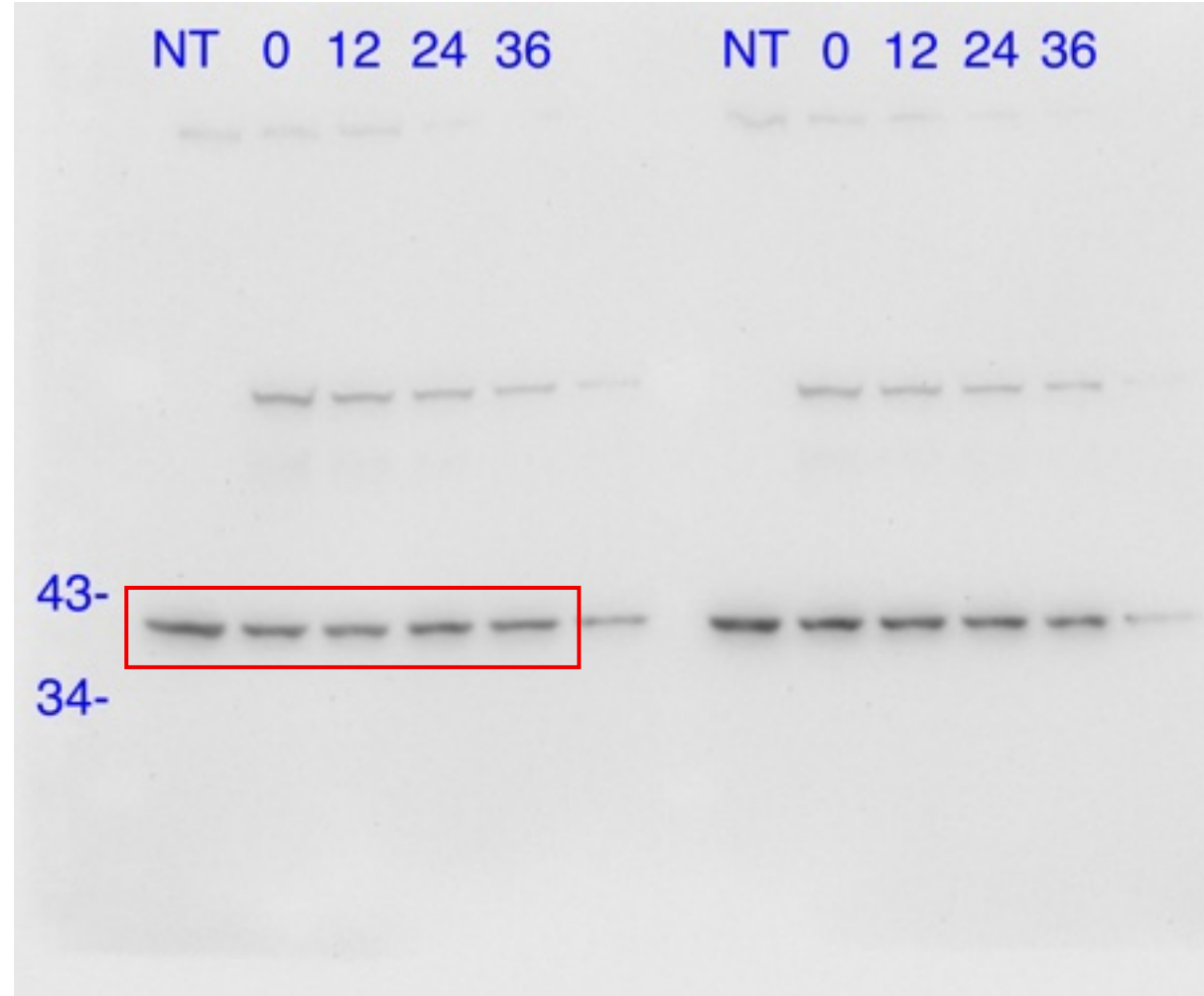

Extended Data Figure 8e   WT-5xU FLuc Flag
(2x rep not shown)

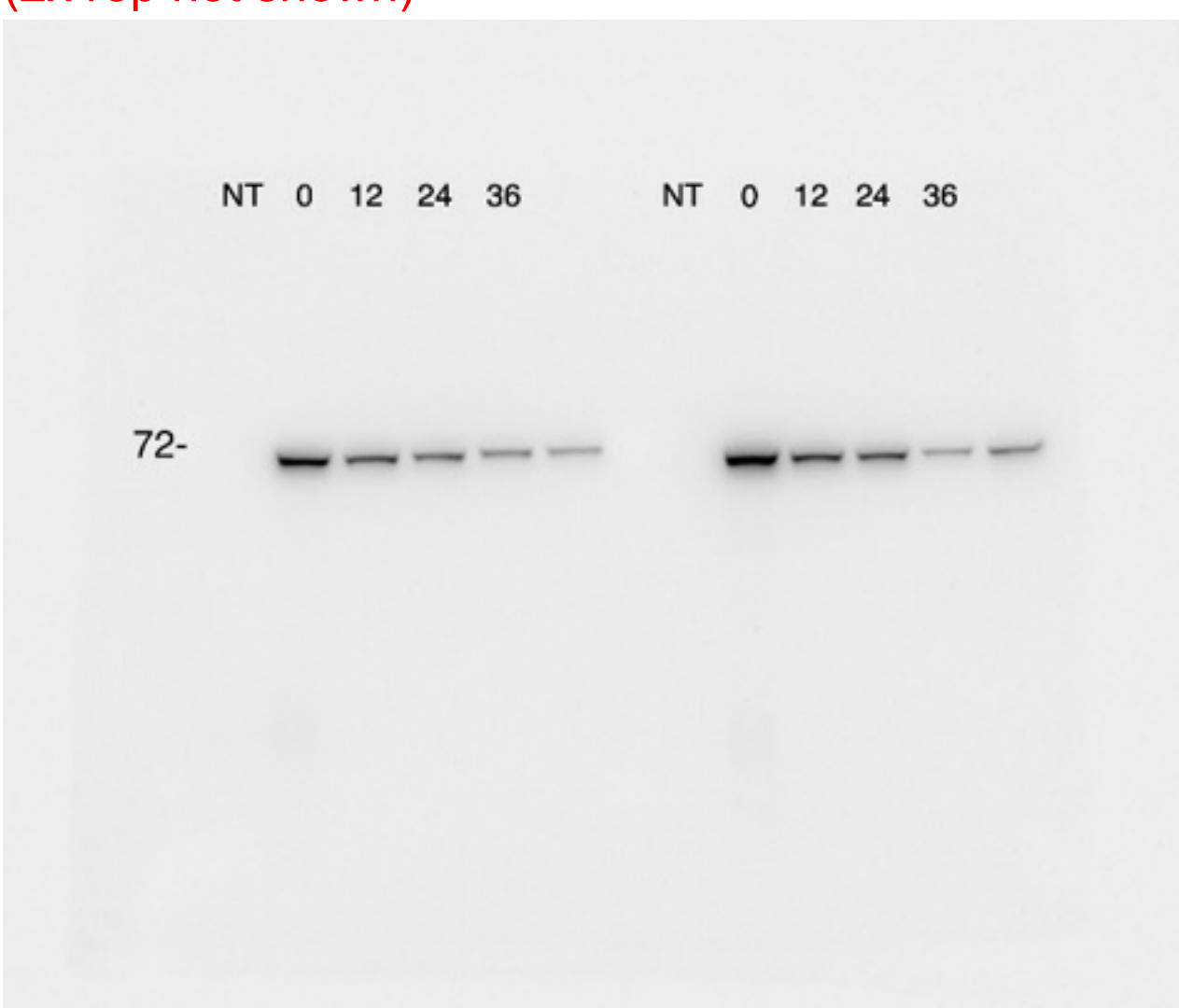

Extended Data Figure 8e   WT-5xU FLuc Flag
(2x rep not shown)

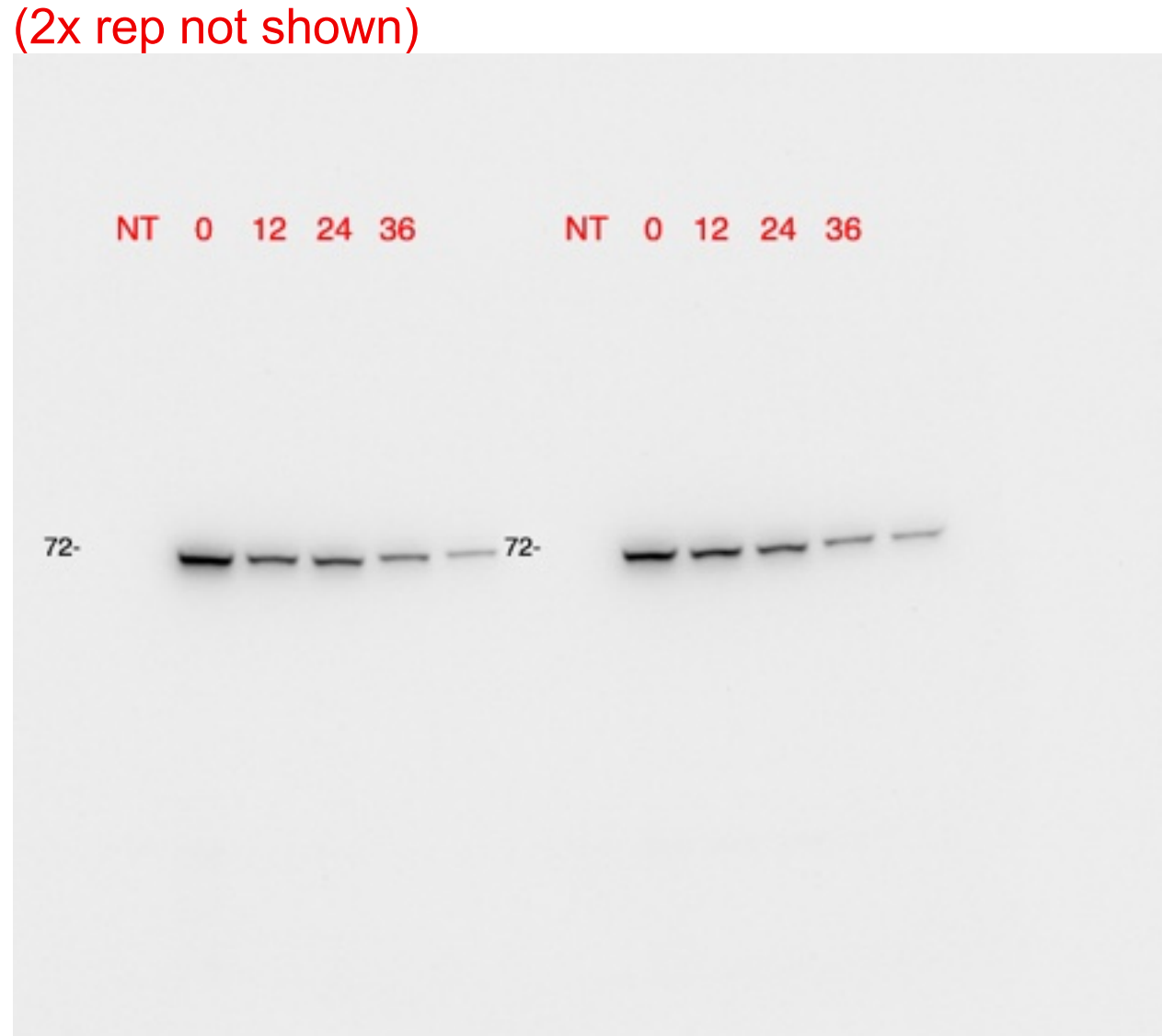

Extended Data Figure 8e   WT-5xU FLuc Flag
(2x rep not shown)

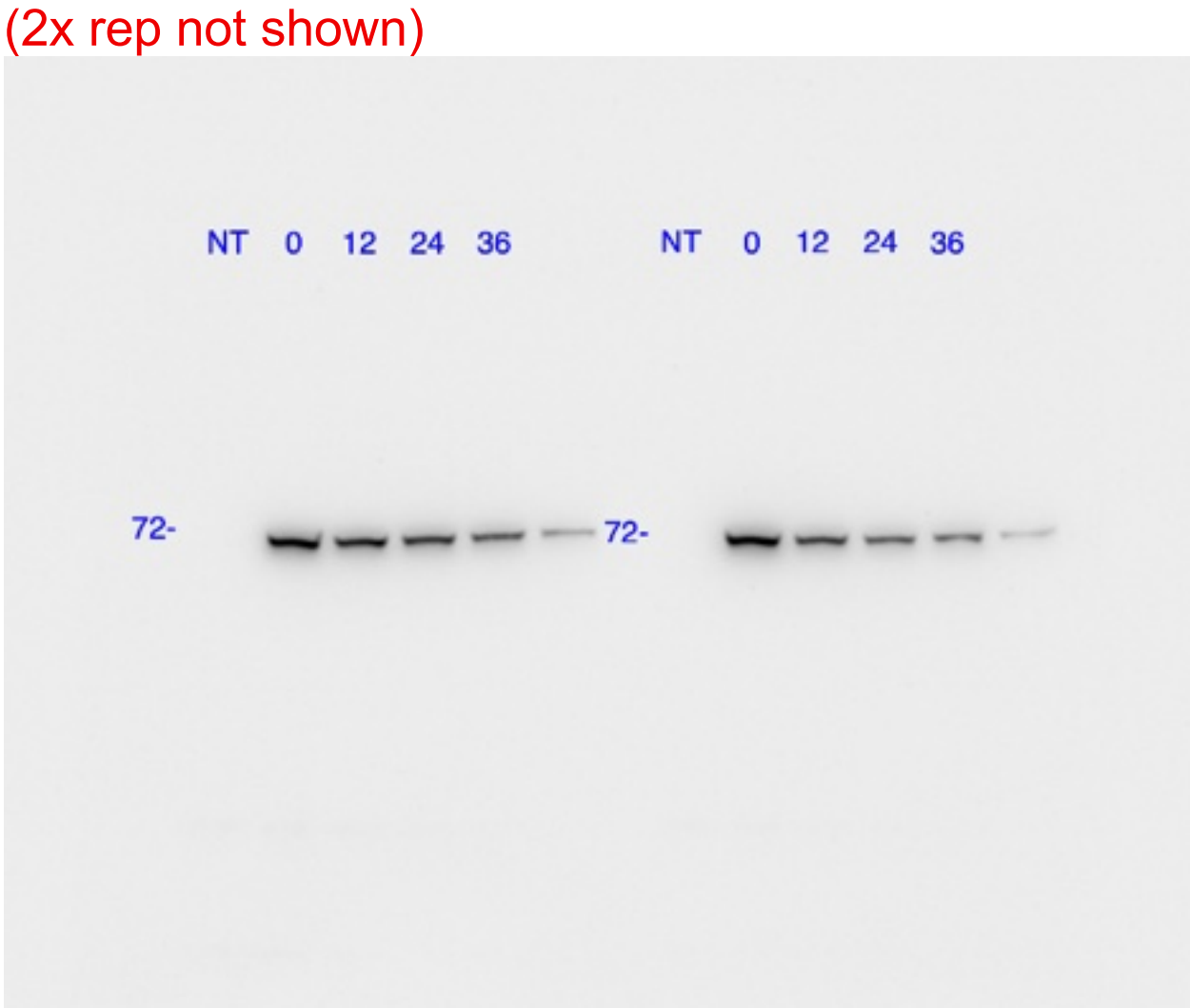

Extended Data Figure 8e   CD25
(1x rep not shown)

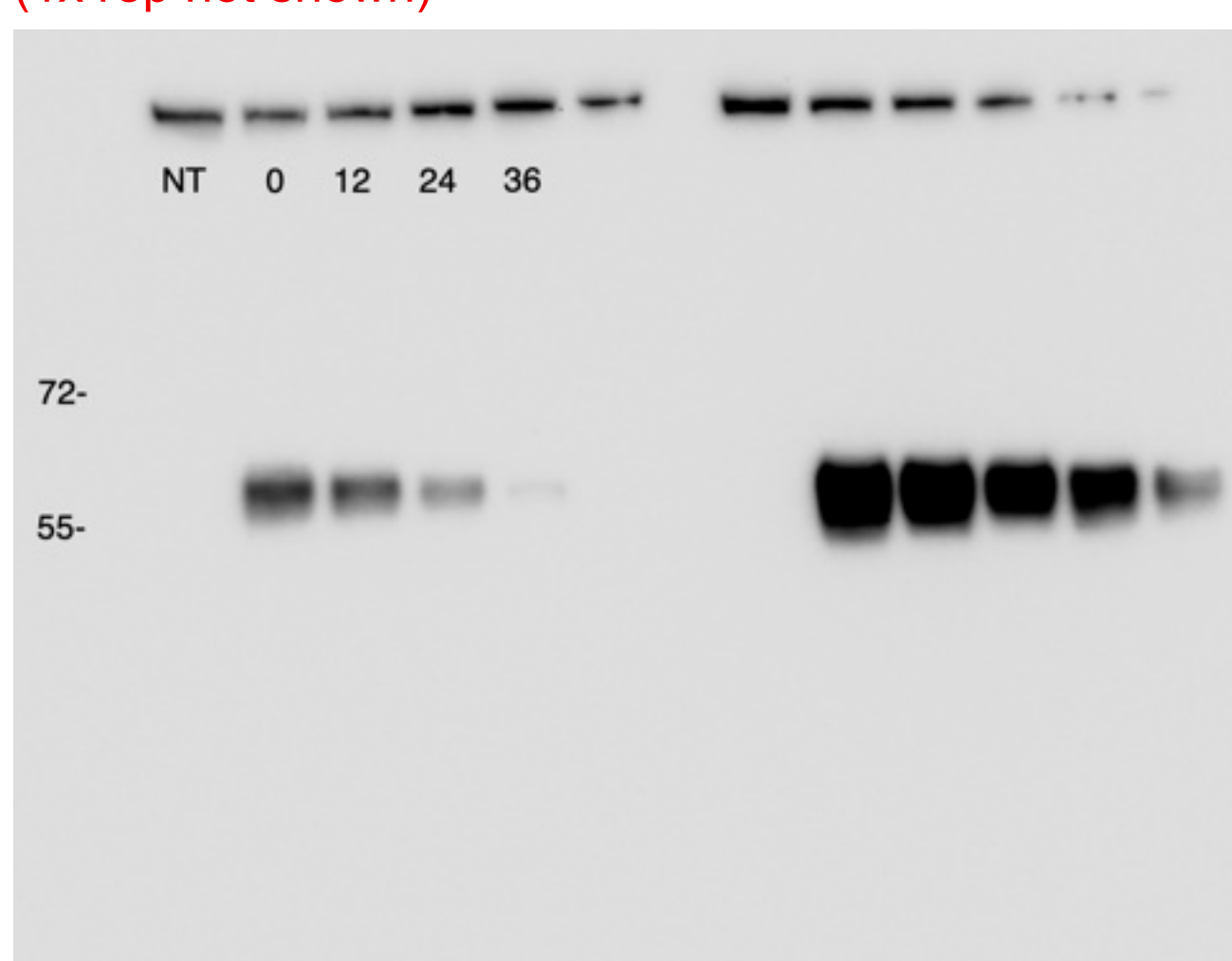

Extended Data Figure 8e CD25
(1x rep not shown)

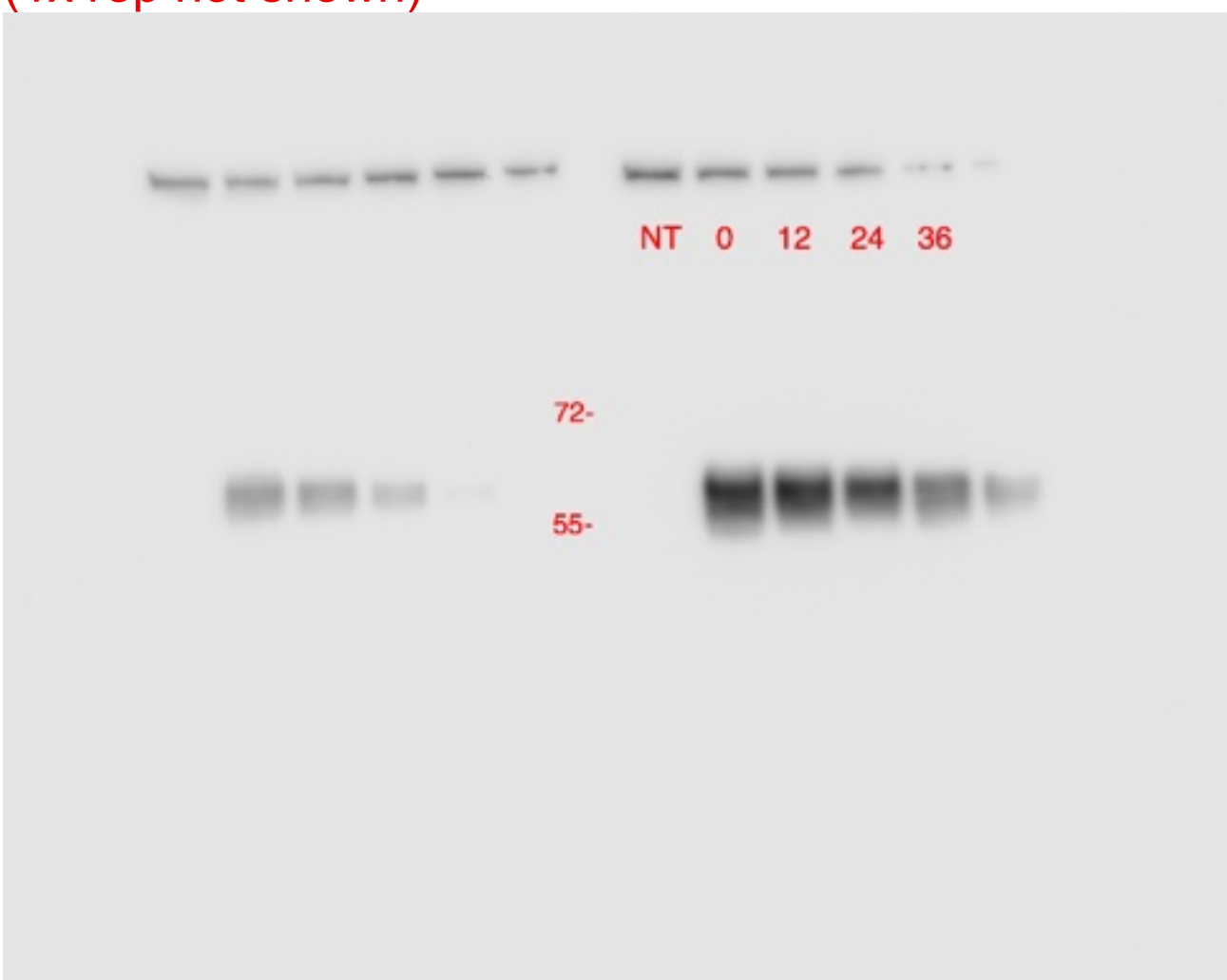

Extended Data Figure 8e CD25
(1x rep not shown)

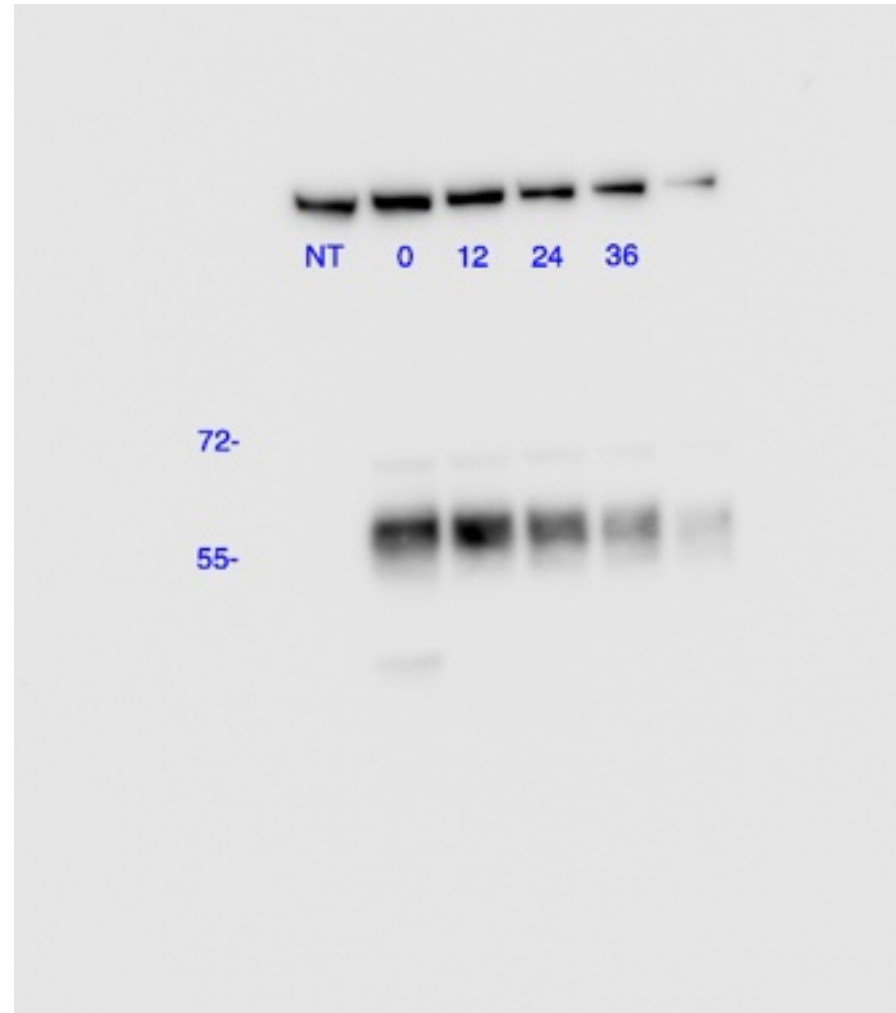

Extended Data Figure 2j,2m
Representative flow cytometry gating strategy to analyze CD25 mRNA expression post transfection

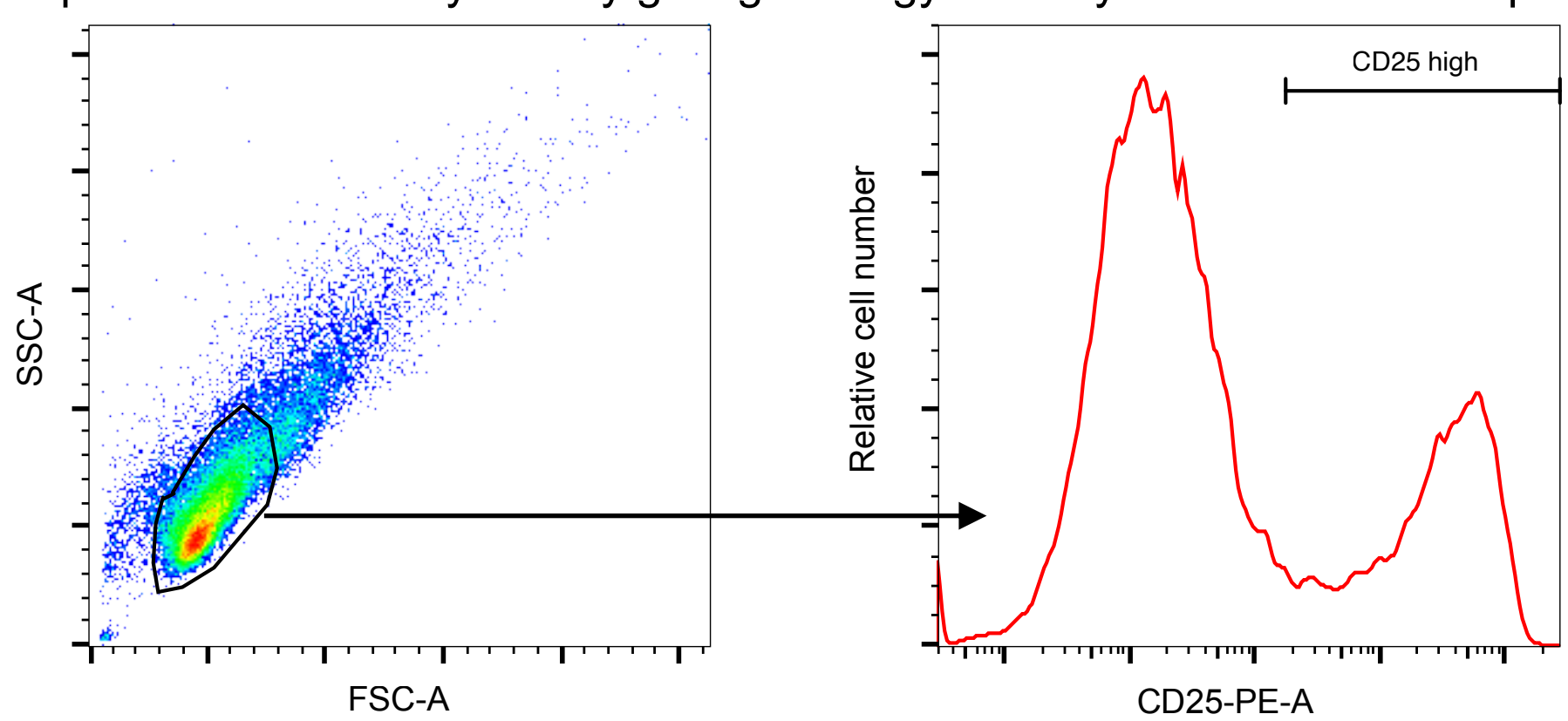

Extended Data Figure 3a
Representative flow cytometry gating strategy to analyze CD14 and CD11c expression in THP1 and PMA-stimulated THP1-derived M0 macrophages.

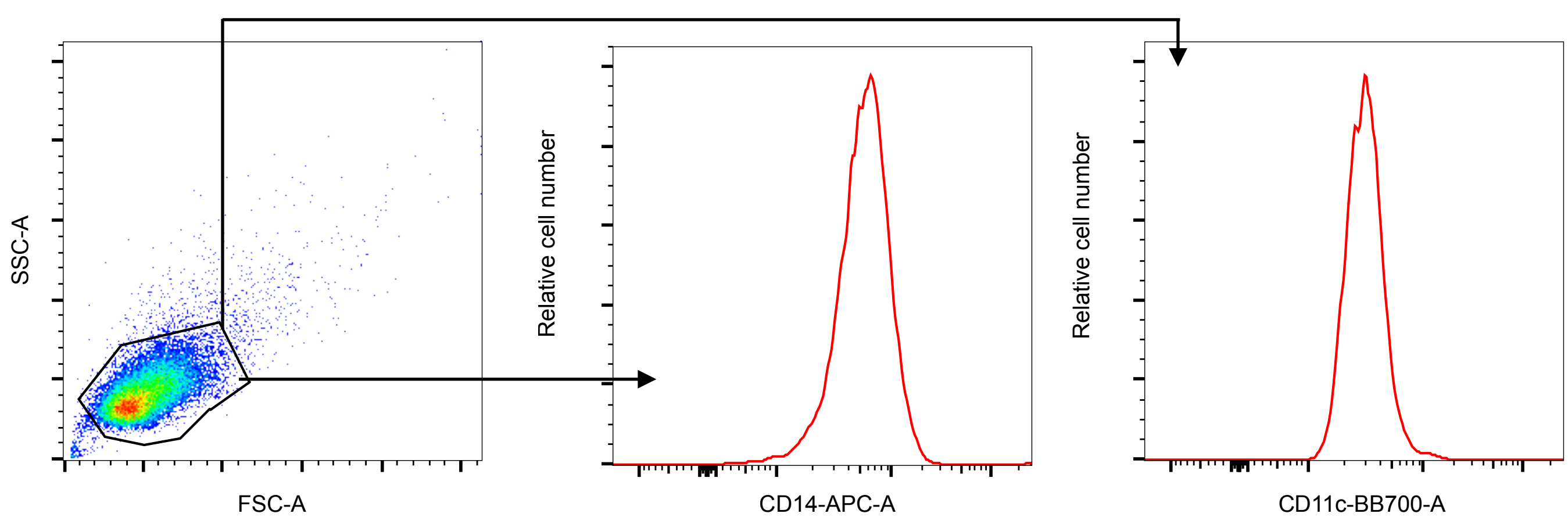

Extended Data Figure 3e, 4a, 6a

Representative flow cytometry gating strategy to analyze CD209 DC-SIGN expression in primary human monocyte-derived dendritic cells (MoDCs)

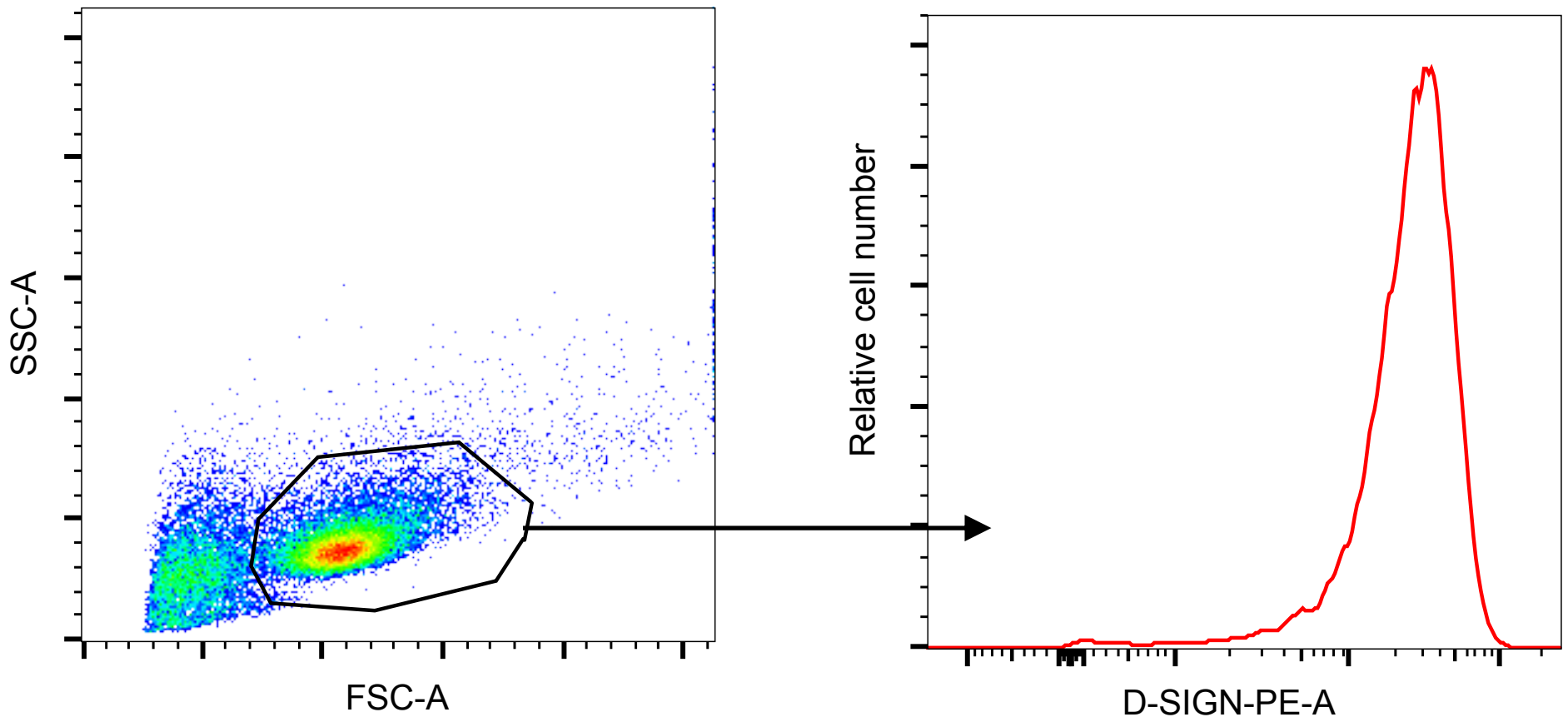

Supplement: Supplementary file 1 [file 41586_2026_10729_MOESM1_ESM.pdf]
