# Supplementary material for: N4-Acetylcytidine enhances synthetic mRNA translation yield and fidelity
Source: Nature. 2026 Jul 1;656(8129):1034–43. doi: 10.1038/s41586-026-10729-8 (PMC13518219; doi:10.1038/s41586-026-10729-8)
Supplement: Supplementary file 2 [file 41586_2026_10729_MOESM2_ESM.pdf]

nature portfolio

Corresponding author(s): Oberdoerffer, Wu

Last updated by author(s): May 18, 2026

Reporting Summary

Nature Portfolio wishes to improve the reproducibility of the work that we publish. This form provides structure for consistency and transparency in reporting. For further information on Nature Portfolio policies, see our Editorial Policies and the Editorial Policy Checklist.

Statistics

For all statistical analyses, confirm that the following items are present in the figure legend, table legend, main text, or Methods section.

n/a	Confirmed	
☐	☒	The exact sample size (*n*) for each experimental group/condition, given as a discrete number and unit of measurement
☐	☒	A statement on whether measurements were taken from distinct samples or whether the same sample was measured repeatedly
☐	☒	The statistical test(s) used AND whether they are one- or two-sided<br>*Only common tests should be described solely by name; describe more complex techniques in the Methods section.*
☐	☒	A description of all covariates tested
☐	☒	A description of any assumptions or corrections, such as tests of normality and adjustment for multiple comparisons
☐	☒	A full description of the statistical parameters including central tendency (e.g. means) or other basic estimates (e.g. regression coefficient) AND variation (e.g. standard deviation) or associated estimates of uncertainty (e.g. confidence intervals)
☐	☒	For null hypothesis testing, the test statistic (e.g. *F*, *t*, *r*) with confidence intervals, effect sizes, degrees of freedom and *P* value noted<br>*Give P values as exact values whenever suitable.*
☒	☐	For Bayesian analysis, information on the choice of priors and Markov chain Monte Carlo settings
☒	☐	For hierarchical and complex designs, identification of the appropriate level for tests and full reporting of outcomes
☒	☐	Estimates of effect sizes (e.g. Cohen's *d*, Pearson's *r*), indicating how they were calculated

Our web collection on statistics for biologists contains articles on many of the points above.

Software and code

Policy information about availability of computer code

Data collection	Nikon Elements software was utilized for image collection.
Data analysis	FastQC (v0.12.1), TrimGalore (v0.6.11), Bowtie2 (v2.5.3), Samtools (v1.23) and Qualimap (v2.2.1) were used for RNA-seq analysis. UMI-tools (v1.1.5), Bowtie (v.1.3.1) and riboWaltz (v2.0) was used for Ribo-Seq. Single-molecule FISH data were analyzed using MATLAB (R2021a), FISH-Quant (v3) and uLocalize (Custom code for analysis of live-cell imaging data is available at: https://github.com/binwulab/uLocalize.git). Analysis of live-cell imaging was done using u-track (v2), visualization and processing of the images was done using ImageJ (v1.53t).

For manuscripts utilizing custom algorithms or software that are central to the research but not yet described in published literature, software must be made available to editors and reviewers. We strongly encourage code deposition in a community repository (e.g. GitHub). See the Nature Portfolio guidelines for submitting code & software for further information.

Data

Policy information about availability of data

All manuscripts must include a data availability statement. This statement should provide the following information, where applicable:

- Accession codes, unique identifiers, or web links for publicly available datasets
- A description of any restrictions on data availability
- For clinical datasets or third party data, please ensure that the statement adheres to our policy

RNA-Seq data are available under GSE301223. Ribo-Seq data are available under PRJNA1280991.

Research involving human participants, their data, or biological material

Policy information about studies with human participants or human data. See also policy information about sex, gender (identity/presentation), and sexual orientation and race, ethnicity and racism.

Reporting on sex and gender	Primary cell experiments utilized human peripheral blood from de-identified human subjects. Identifiers such as sex and race are not available.
Reporting on race, ethnicity, or other socially relevant groupings	Primary cell experiments utilized human peripheral blood from de-identified human subjects. Identifiers such as sex and race are not available.
Population characteristics	Primary cell experiments utilized peripheral blood from de-identified human subjects. Identifiers such as sex and race are not available.
Recruitment	Human peripheral blood was obtained from de-identified healthy donors through the NIH Clinical Center, Department of Transfusion Medicine, Research Blood Donor Program. Participants provided informed consent prior to participation.
Ethics oversight	The Research Blood Donor Program is under NIH Institutional Review Board – approved protocol IRB# 99CC0168. All research was performed in accordance with all relevant guidelines and regulations.

Note that full information on the approval of the study protocol must also be provided in the manuscript.

Field-specific reporting

Please select the one below that is the best fit for your research. If you are not sure, read the appropriate sections before making your selection.

☒ Life sciences ☐ Behavioural & social sciences ☐ Ecological, evolutionary & environmental sciences

For a reference copy of the document with all sections, see nature.com/documents/nr-reporting-summary-flat.pdf

Life sciences study design

All studies must disclose on these points even when the disclosure is negative.

Sample size	For all cell culture experiments, a minimum of three biological replicates were used to adhere to standards in the field. For mouse experiments, sample sizes were selected based on prior published work (Zhu, Y., Ma, J., Shen, R. et al. Screening for lipid nanoparticles that modulate the immune activity of helper T cells towards enhanced antitumour activity. Nat. Biomed. Eng 8, 544–560 (2024). https://doi.org/10.1038/s41551-023-01131-0), where comparable group sizes were sufficient to detect biologically meaningful differences.
Data exclusions	No data were excluded.
Replication	All experiments were performed on multiple days under multiple conditions with independent biological replicates. All reported data are robust and reproducible. The in vivo IVIS experiments were independently replicated twice with multiple mice used in each experiment, and the datasets were pooled for analysis.
Randomization	The majority of experiments were performed in human cell lines or with de-identified human donors. Animal experiments exclusively utilized female mice. Mice were randomly allocated into experimental groups, while the body weight (17 to 28 grams) and age (6 to 8 weeks) were maintained within the selected ranges.
Blinding	For cell culture, data collection was not performed blindly because the experiments involved conditions with objectively measured outcomes and predefined treatment groups. In vivo mouse experiments and data collection were performed blinded to group allocation. Investigators remained blinded during data analysis whenever feasible.

Reporting for specific materials, systems and methods

We require information from authors about some types of materials, experimental systems and methods used in many studies. Here, indicate whether each material, system or method listed is relevant to your study. If you are not sure if a list item applies to your research, read the appropriate section before selecting a response.

Materials & experimental systems

n/a	Involved in the study
☐	☒ Antibodies
☐	☒ Eukaryotic cell lines
☒	☐ Palaeontology and archaeology
☐	☒ Animals and other organisms
☒	☐ Clinical data
☒	☐ Dual use research of concern
☒	☐ Plants

Methods

n/a	Involved in the study
☒	☐ ChIP-seq
☐	☒ Flow cytometry
☒	☐ MRI-based neuroimaging

Antibodies

Antibodies used

Rabbit anti-eIF2α (1:1,000, Cell Signaling Technology, Cat. #: 9722), rabbit anti-phospho-eIF2α ((S51), [E90], 1:1,000, abcam, Cat. #: ab32157), mouse anti-NanoLuc ([965808], 1:500, R&D, Cat. #: MAB10026), rabbit anti-TNFα ([EPR22598-212], 1:1,000, abcam, Cat. #: ab255275), mouse anti-β-tubulin ([D3U1W], 1:1,000, Cell Signaling Technology, Cat. #: 86298), rabbit anti-CD25 (([SP176], 1:300, abcam, Cat. #: ab231441), mouse anti-FLAG ([M2], 1:1,000, Sigma-Aldrich, Cat. #: F1804), mouse anti-JNK ([1A12E1], 1:1,000, Proteintech, Cat. #:66210-1-Ig), rabbit anti-phospho-SAPK/JNK ((Thr183/Tyr185) [81E11], 1:1,000, Cell Signaling Technology, Cat. #: 4668S), mouse anti-GAPDH ([6C5], Santa Cruz Biotechnology, Cat. #: sc-32233), rabbit anti-phospho-p38 MAPK ((Thr180/Tyr182), 1:1,000, Cell Signaling Technology, Cat. #: 9211S), rabbit anti-p38 MAPK (1:1,000, Cell Signaling Technology, Cat. #: 9212S), rabbit anti-TLR8 (1:1,000, Thermo Fisher Scientific, PA5-102413), rabbit anti-TLR7 ([EPR2088(2)], 1:1,000, abcam, Cat. #: ab124928), rabbit anti-lamin B1 ([EPR8985(B)], 1:1,000, abcam, Cat. #: ab133741), rabbit anti-ZNF598 ([5H5L17], 1:1,000, Thermo Fisher, Cat. #: 703601), mouse anti-PKR ([1441CT628.33.40], 1:1,000, Thermo Fisher Scientific, Cat#: M5-37667), rabbit anti-phospho-PKR/EIF2AK2-T446 ([ARC0293], 1:1,000, ABclonal, Cat#: AP1134), PE-conjugated anti-Hu/NHP CD25 ([CD25-4E3], 1:100, eBioscience, Cat. #: 12-0257-42), APC-conjugated anti-Hu CD14 ([61D3], 1:100, eBioscience, Cat. #: 17-0149-42), PerCP/Cyanine5.5-conjugated anti-human CD11c ([3.9], 1:100, Biolegend, Cat. #: 301624), PE-conjugated anti-CD209 ([9E9A8], 1:100, Biolegend, Cat. #: 330105), anti GFP antibody Chicken IgY (1:5,000, Aves Labs, Cat. #: GFP-1010), Goat anti-Chicken IgY (H+L) Secondary Antibody, Alexa Fluor 488 (1:1,000, Cat. #: A-11039), and mouse anti-FLAG ([M2], 1:200, Sigma-Aldrich, Cat. #: F3165)

Validation

All antibodies are commercially available and validated as follows according to the manufacturer's websites:
Rabbit polyclonal anti-eIF2α (Cell Signaling Technology, Cat. #: 9722) detects endogenous levels of total eIF2α in human, mouse, rat and monkey. It is validated for WB.
Rabbit monoclonal anti-phospho eIF2α ((phospho S51) [E90], 1:1,000, abcam, Cat. #: ab32157) detects S51-phosphorylation on EIF2S1 in human, mouse, neurospora crassa, and rat by WB, IHC-P, Dot Blot.
Mouse monoclonal anti-Nanoluc ([965808], 1:500, R&D, Cat. #: MAB10026) antibody detects NanoLuc Luciferase (NLuc) in direct ELISAs and WB. NanoLuc is a registered trademarks of Promega Corporation.
Rabbit monoclonal anti-TNFα ([EPR22598-212], abcam, Cat. #: ab255275) antibody detects TNF alpha in human by FC (Intra), WB, IP.
Mouse monoclonal anti-β-tubulin ([D3U1W], Cell Signaling Technology, Cat. #: 86298), antibody detects endogenous levels of total β-tubulin protein in human, mouse, rat, hamster and monkey by WB, IHC-P, IF.
Rabbit monoclonal anti-CD25 ([SP176], 1:300, abcam, Cat. #: ab231441) detects IL-2 Receptor alpha (CD25) in human and mouse by WB, IHC-P, ICC/IF.
Mouse monoclonal anti-FLAG ([M2], 1:1,000, Sigma-Aldrich, Cat. #: F1804) antibody detects the FLAG peptide sequence at the N-terminus, Met-N-terminus, C-terminus, and internal sites of the fusion protein by WB, IP, IHC, IF/ICC.
Mouse monoclonal anti-GAPDH ([6C5], Santa Cruz Biotechnology, Cat. #: sc-32233), antibody detects GAPDH protein in human, mouse, rabbit and rat by WB, IP, IF.
Mouse monoclonal anti-JNK ([1A12E1], 1:1,000, Proteintech, Cat. #:66210-1-Ig) antibody detects JNK protein in human, mouse and rat by WB, IHC, IF/ICC ,FC (Intra), ELISA.
Rabbit monoclonal anti-phospho-SAPK/JNK ((Thr183/Tyr185) [81E11] 1:1,000, Cell Signaling Technology, Cat. #: 4668S), antibody detects endogenous levels of p46 and p54 SAPK/JNK when phosphorylated at Thr183 and Tyr185. This antibody will also react with SAPK/JNK singly phosphorylated at Tyr185. This antibody may cross-react with phosphorylated p44/42 or p38 MAP kinases in human, mouse, rat, D. melanogaster, and S. cerevisiae by WB, IP, IHC.
Rabbit polyclonal anti-phospho-p38 MAPK ((Thr180/Tyr182), 1:1,000, Cell Signaling Technology, Cat. #: 9211S) antibody detects endogenous levels of p38 MAPK only when activated by phosphorylation at threonine 180 and tyrosine 182. This antibody does not cross-react with the phosphorylated forms of either p42/44 MAPK or SAPK/JNK. This antibody will also react with p38 singly phosphorylated at Thr180 and singly phosphorylated at Tyr182 in human, mouse, rat, monkey, D. melanogaster, pig, S. cerevisiae by WB, IP and ICC/IF.
Rabbit polyclonal anti-p38 MAPK (1:1,000, Cell Signaling Technology, Cat. #: 9212S) antibody detects endogenous levels of total p38α, -β or -γ MAPK protein. This antibody does not recognize p38δ, JNK/SAPK or p44/42 MAPK in human, mouse, rat, monkey, guinea pig by WB, IHC-P and ICC/IF.
Rabbit polyclonal anti-TLR8 (1:1,000, Thermo Fisher Scientific, PA5-102413) antibody detects endogenous levels of total TLR8 in human, mouse, rat by WB, IHC(-P) and FC according to the manufacturer's website.
Rabbit recombinant monoclonal anti-TLR7 ([EPR2088(2)], 1:1,000, abcam, Cat. #: ab124928) antibody detects endogenous levels of TLR7 in by IHC-P, WB.
Rabbit monoclonal anti-lamin B1 ([EPR8985(B)], 1:1,000, abcam, Cat. #: ab133741) - Nuclear Envelope Marker antibody detects Lamin B1 in human, mouse, rat by WB, IP, IHC-P, ICC/IF according to the manufacturer's website.
Rabbit recombinant monoclonal anti-ZNF598 ([5H5L17], 1:1,000, Thermo Fisher, Cat. #: 703601) detects endogenous levels of ZNF598 in human by WB and ICC/IF.
Mouse monoclonal anti-PKR ([1441CT628.33.40], 1:1,000, Thermo Fisher Scientific, Cat#: M5-37667) detects endogenous levels of PKR in human by WB and ICC/IF.
Rabbit monoclonal anti-phospho-PKR/EIF2AK2-T446 ([ARC0293], 1:1,000, ABclonal, Cat. #: AP1134) antibody detects endogenous levels of phospho-PKR/EIF2AK2-T446. Species reactivity: human, mouse, pig. It is validated for WB and ELISA.

Mouse monoclonal PE-conjugated anti-Hu/NHP CD25 antibody ([CD25-4E3], eBioscience, Cat. #: 12-0257-42) reacts to human CD25, which is also known as the low-affinity interleukin (IL)-2 receptor alpha, in human and non-human primates by FC.
Mouse monoclonal APC-conjugated anti-HuCD14 antibody ([61D3], eBioscience, Cat. #: 17-0149-42) reacts with human CD14, a 53-55 kDa GPI-linked glycoprotein in human by FC.
Mouse monoclonal PerCP/Cyanine5.5-conjugated anti-human CD11c antibody ([3.9], Biolegend, Cat. #: 301624 preferentially binds the activated form of CD11c, is specific for the I domain of CD11c, and is able to partially block the binding of CD11c and ICAM-4. 3.9 binding is divalent cation dependent in human, cynomolgus, rhesus by FC according to the manufacturer's website.
Mouse monoclonal PE-conjugated anti-CD209 (DC-SIGN) antibody ([9E9A8], Biolegend, Cat. #: 330105) reacts with the extracellular domain of DC-SIGN in human by FC.
Chicken polyclonal anti-GFP antibody (1:1,000, Aves Labs, Cat. #: GFP-1020) detects Green Fluorescent Protein (GFP) and related variants including eGFP, YFP, CFP and others by ELISA, IHC, ICC, and WB according to the manufacturer's website.
Alexa-488-goat polyclonal anti-chicken secondary antibody (IgY (H+L), Thermo Fisher Scientific, Cat. #: A-11039) detects immunoglobulin Y in chicken by IHC, ICC/IF, FC.
Mouse monoclonal anti-FLAG ([M2], 1:200, Sigma-Aldrich, Cat. #: F3165) antibody detects the FLAG peptide sequence at the N-terminus, Met-N-terminus, C-terminus, and internal sites of the fusion protein by WB, IP, ChIP, IHC, IF/ICC, ELISA, FC, EM.

Eukaryotic cell lines

Policy information about cell lines and Sex and Gender in Research

Cell line source(s)	HeLa cells were obtained from ATCC, Cat. #: CCL-2. THP-1 cells were obtained from ATCC, Cat. #: TIB-202. U-2 OS cells were obtained from ATCC, Cat. #: HTB-96.
Authentication	None of the cell lines used were authenticated.
Mycoplasma contamination	All cell lines were tested and negative for mycoplasma.
Commonly misidentified lines (See ICLAC register)	Commonly misidentified cell lines were used in this study.

Animals and other research organisms

Policy information about studies involving animals; ARRIVE guidelines recommended for reporting animal research, and Sex and Gender in Research

Laboratory animals	Female BALB/c mice (6–8 weeks) were obtained from the Jackson Laboratory and randomly grouped. Mice were housed under standard specific pathogen-free (SPF) conditions with a 12-hour light/12-hour dark cycle and had ad libitum access to food and water.
Wild animals	No wild animals were used in this study.
Reporting on sex	All animals used in the study were female.
Field-collected samples	No field-collected samples were used in this study.
Ethics oversight	All animal procedures were performed with ethical compliance and approval by the Johns Hopkins Institutional Animal Care and Use Committee (protocol #MO23E31).

Note that full information on the approval of the study protocol must also be provided in the manuscript.

Plants

Seed stocks	N/A
Novel plant genotypes	N/A
Authentication	N/A

Flow Cytometry

Plots

Confirm that:

☒ The axis labels state the marker and fluorochrome used (e.g. CD4-FITC).

☒ The axis scales are clearly visible. Include numbers along axes only for bottom left plot of group (a 'group' is an analysis of identical markers).

☒ All plots are contour plots with outliers or pseudocolor plots.

☒ A numerical value for number of cells or percentage (with statistics) is provided.

Methodology

Sample preparation	0.1-1x10^6 cells were stained in 100 µL staining buffer (1% FBS in PBS) for 30 mins at room temperature in the dark, washed twice with PBS and resuspended in 1% paraformaldehyde containing staining buffer.
Instrument	Flow cytometry acquisition was performed on BD FACSymphony A5.
Software	Data was examined using FACSDiva software (v9.3.1, BD Bioscience), data was further analyzed using FlowJo (v10.8.1, BD).
Cell population abundance	No sorting was performed. The entire live population was analyzed.
Gating strategy	Primary gating was performed based on SSC/FSC, followed by analysis of the live population.

☒ Tick this box to confirm that a figure exemplifying the gating strategy is provided in the Supplementary Information.
